# Supplementary material for: Lineage-level divergence of copepod glycerol transporters and the emergence of isoform-specific trafficking regulation
Source: Commun Biol. 2021 May 31;4:643. doi: 10.1038/s42003-021-01921-9 (PMC8167128; doi:10.1038/s42003-021-01921-9)
Supplement: Supplementary file 2 — Supplementary information [file 42003_2021_1921_MOESM2_ESM.pdf]

Lineage-level divergence of copepod glycerol transporters and the emergence of isoform-specific trafficking regulation

Marc Catalán-García, François Chauvigné, Jon Anders Stavang, Frank Nilsen, Joan Cerdà, Roderick Nigel Finn

Supplementary Information

Page 2-3: **Figure S1.** Molecular phylogeny of mandibulatan glycerol transporters.

Page 4: **Figure S2.** Antibody specificity against *L. salmonis* Glps

Page 5-6: **Figure S3.** Enterocyte localization of Glp1_v2 in female *L. salmonis*, and control sections probed with preadsorbed antibodies

Page 7: **Figure S4.** Regulation of *L. salmonis* Glp intracellullar trafficking in *X. laevis* oocytes

Page 8: **Figure S5.** Identification of PKC and PKA phosphorylation sites in *L. salmonis* Glps

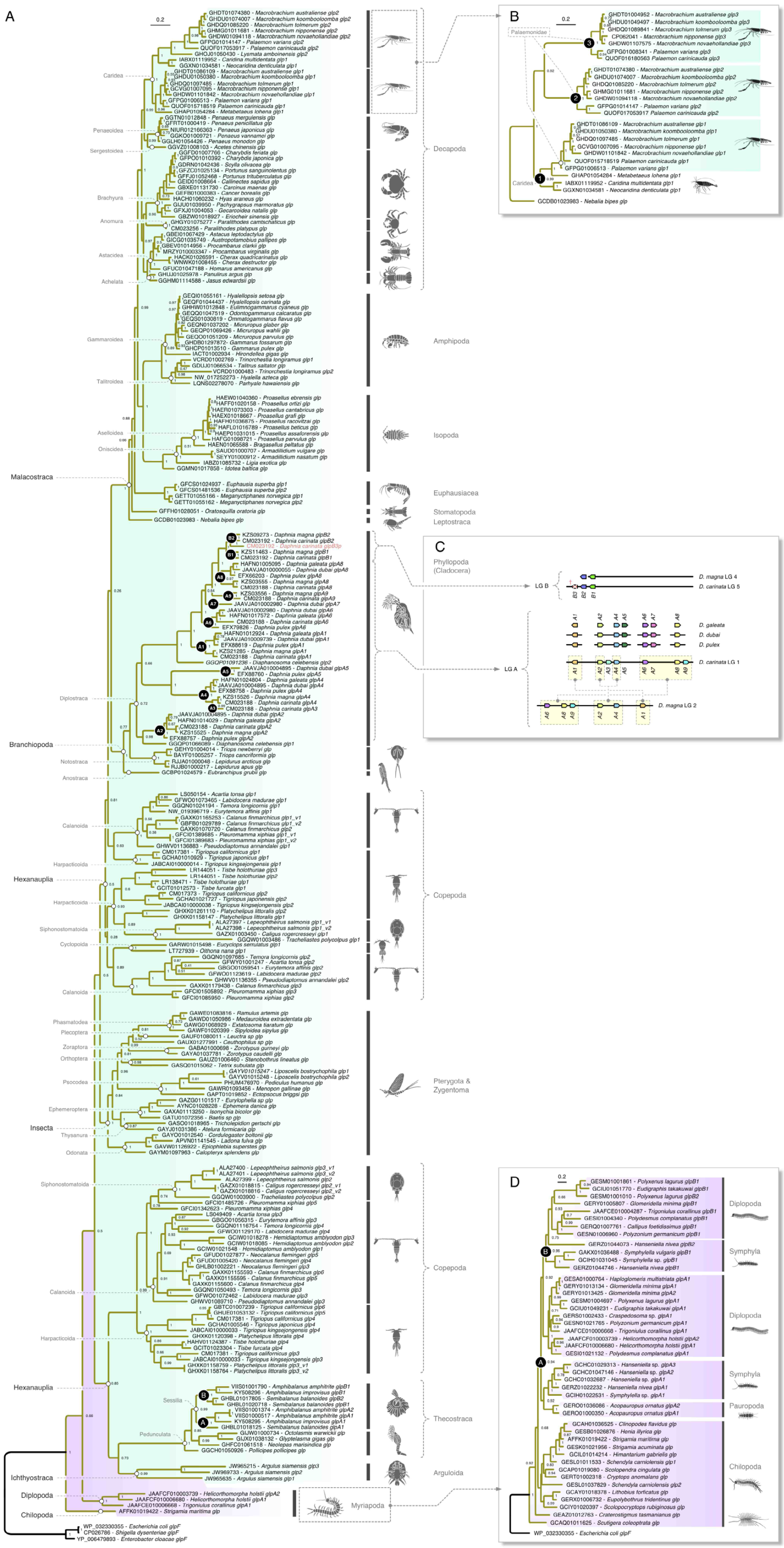
A
B
C
D
Decapoda
Amphipoda
Isopoda
Euphausiacea
Stomatopoda
Leptostraca
Phyllopoda (Cladocera)
Copepoda
Pterygota & Zygentoma
Thecostraca
Arguloida
Myriapoda
Diplopoda
Symphyla
Pauropoda
Chilopoda
Malacostraca
Branchiopoda
Hexanauplia
Insecta
Ichthyostraca
Palaemonidae
Caridea
LG B
LG A
D. magna LG 4
D. carinata LG 5
D. carinata LG 1
D. magna LG 2
D. galeata
D. dubai
D. pulex

Supplementary Figure S1. Molecular phylogeny of mandibulatan glycerol transporters. (A) Bayesian majority rule consensus tree rooted with eubacterial *glpF*. The tree is inferred from 15 million MCMC generations (nucmodel = 4by4, nst = 2, rates = gamma) of 224,094 aligned nucleotide sites of 249 mandibulaten CDS partitioned by codon. Support values shown at each node are Bayesian posterior probabilities. (B) Mid-point rooted Bayesian majority rule consensus tree of caridean shrimp and prawn *glp* CDS inferred from 1 million MCMC generations (nucmodel = 4by4, nst = 2, rates = gamma) of 24,118 nucleotide sites partitioned by codon. (C) Syntentic relationships of of *Daphnidae glp* genes. (D) Bayesian majority rule consensus tree of 44 myriapod *glp* CDS rooted with eubacterial *glpF*. The tree is inferred inferred from 1 million MCMC generations (nucmodel = 4by4, nst = 2, rates = gamma) of 35,723 nucleotide sites partitioned by codon. Bayesian posterior probabilities are annotated at each node. Accession numbers are listed for each CDS and scale bars indicate the expected rate of substitutions per site.

Supplementary Figure S2.

Antibody specificity against *L. salmonis* Glps. (S2A-S2O): Uncropped Western blots of Fig. 2A-J in main text.

Fig S2A (Fig 2A Glp1_v1)

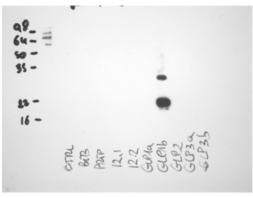

Fig S2B (Fig 2B Glp1_v2)

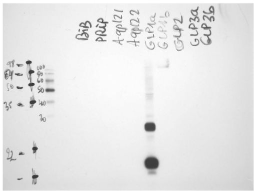

Fig S2C (Fig 2C Glp2)

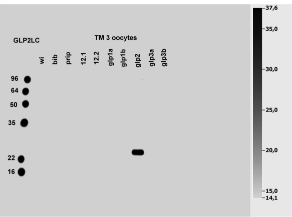

Fig S2D (Fig 2D Glp3_v1)

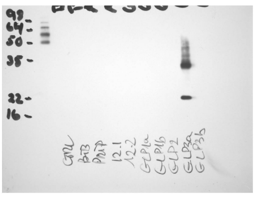

Fig S2E (Fig 2E Glp3_v2)

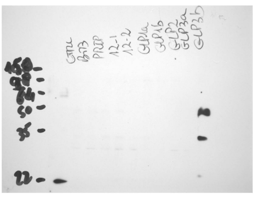

Fig S2F (Fig 2F Glp1_v1)

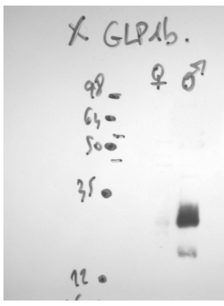

Fig S2G (Fig 2F Glp1_v1 preadsorbed)

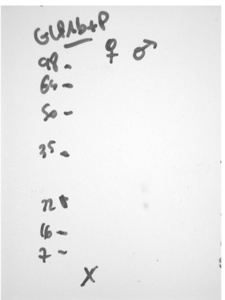

Fig S2H (Fig 2G Glp1_v2)

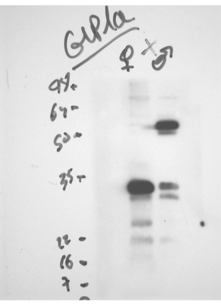

Fig S2I (Fig 2G Glp1_v2 preadsorbed)

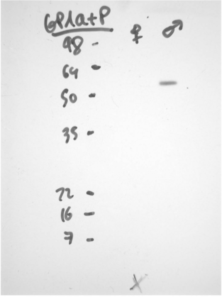

Fig S2J (Fig 2H Glp2)

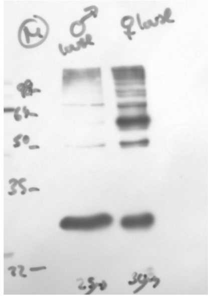

Fig S2K (Fig 2H Glp2 preadsorbed)

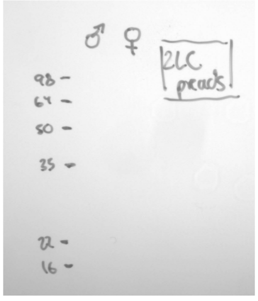

Fig S2L (Fig 2I Glp3_v1)

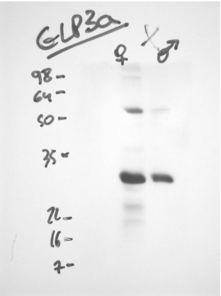

Fig S2M (Fig 2I Glp3_v1 preadsorbed)

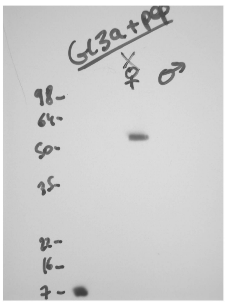

Fig S2N (Fig 2J Glp3_v2)

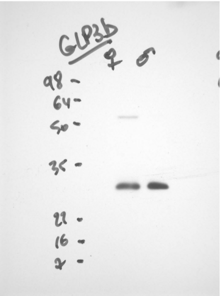

Fig S2O (Fig 2J Glp3_v2 preadsorbed)

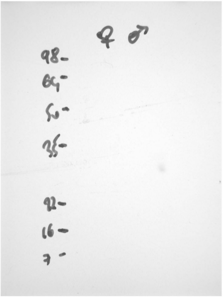

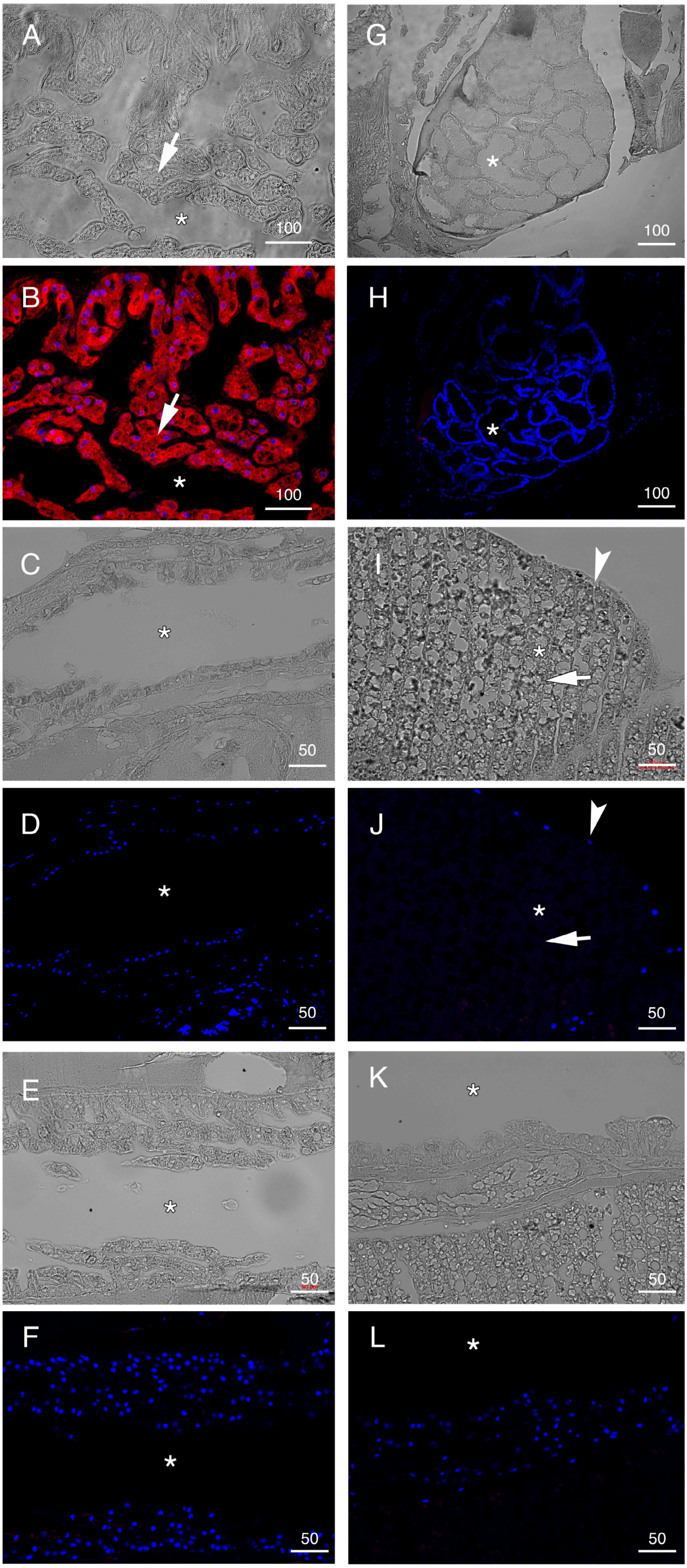

A
100
B
100
C
50
D
50
E
50
F
50
G
100
H
100
I
50
J
50
K
50
L
50

Supplementary Figure S3. Enterocyte localization of Glp1_v2 in female *L. salmonis*, and control sections probed with preadsorbed antibodies. Representative bright field (A) and immunofluorescence (B) microscopy images of Glp1_v2 localization in female enterocytes (arrows). Representative bright field (C, E, G, I and K) and immunofluorescence (D, F, H, J and L) microscopy images of male intestines (C, D) and female intestines (E, F), ovaries (G, H) and immature egg strings (I, J) probed with preadsorbed Glp1_v2 antiserum, and female intestines (K, L) probed with preadsorbed Glp3_v1 antiserum. Sections were counterstained with DAPI (blue). Asterisks indicate the intestinal lumen (A, B, C, D, E, F, I and J) or oocyte (G, H, I and J). Arrows in I and J indicate the oolemma and arrowheads in I and J indicate the epithelia lining the immature egg strings. Scale bars are in µm.

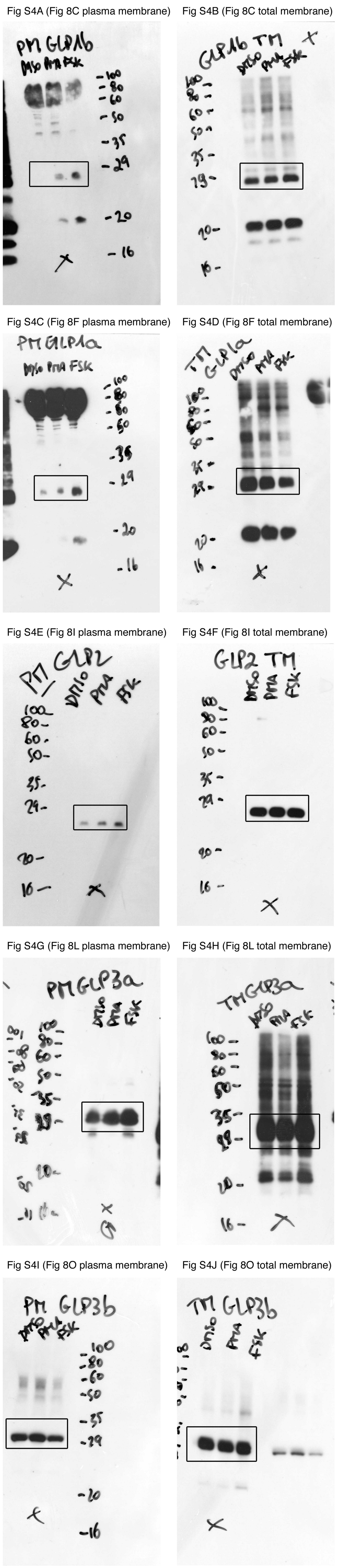

Supplementary Figure S4. Regulation of *L. salmonis* Glp intracellullar trafficking in *X. laevis* oocytes. (S4A-S4J): Uncropped Western blots of Fig. 8C-D and Fig. 8I, L and O in main text. Boxes illustrate regions shown in main text.

Fig S5A (Fig 10D: Glp1_v1)

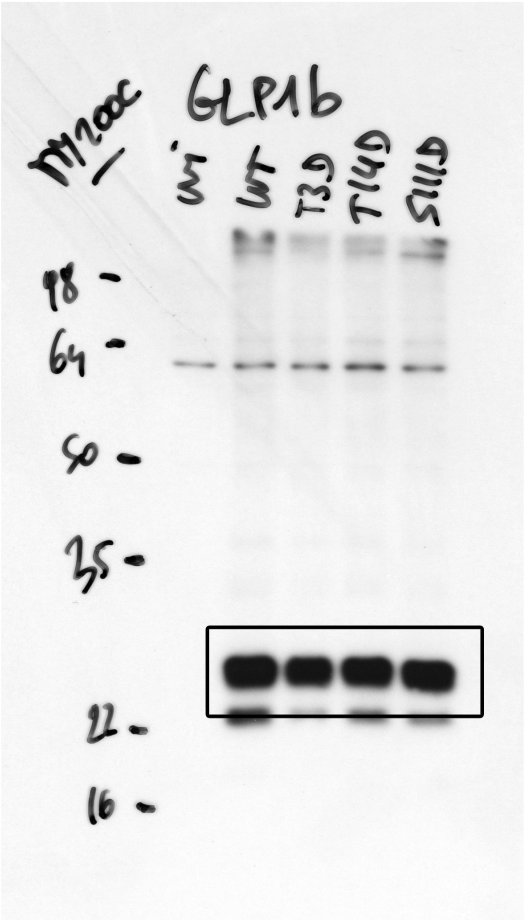

Fig S5B (Fig 10D: Glp1_v2)

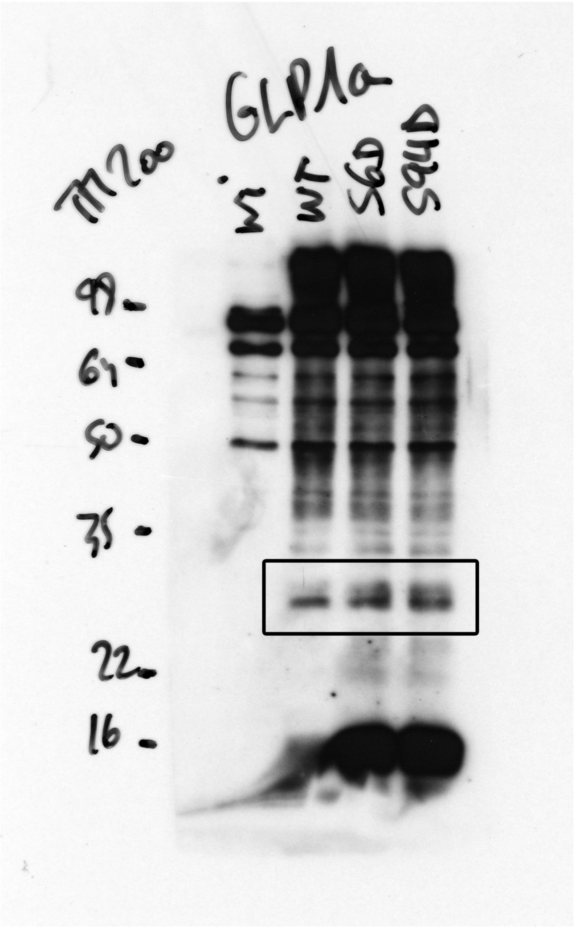

Fig S5C (Fig 10G: Glp2)

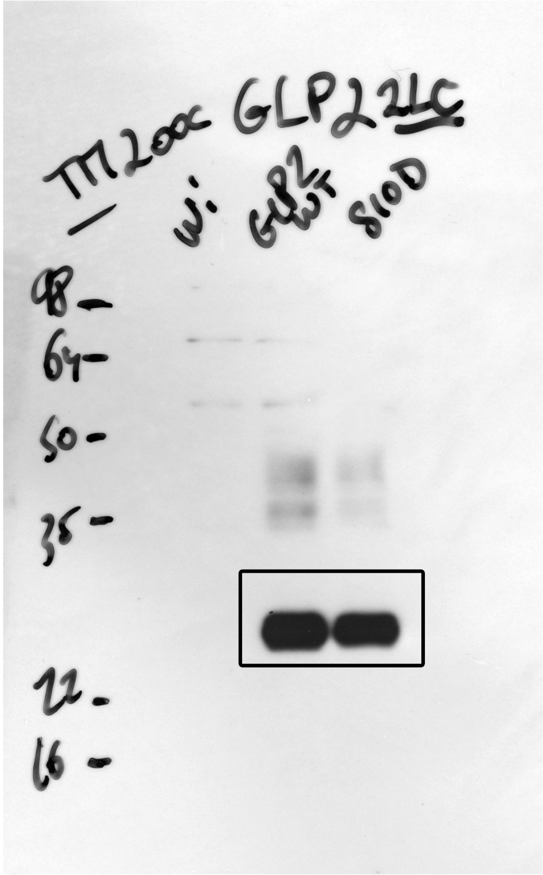

Fig S5D (Fig 10G: Glp3_v1)

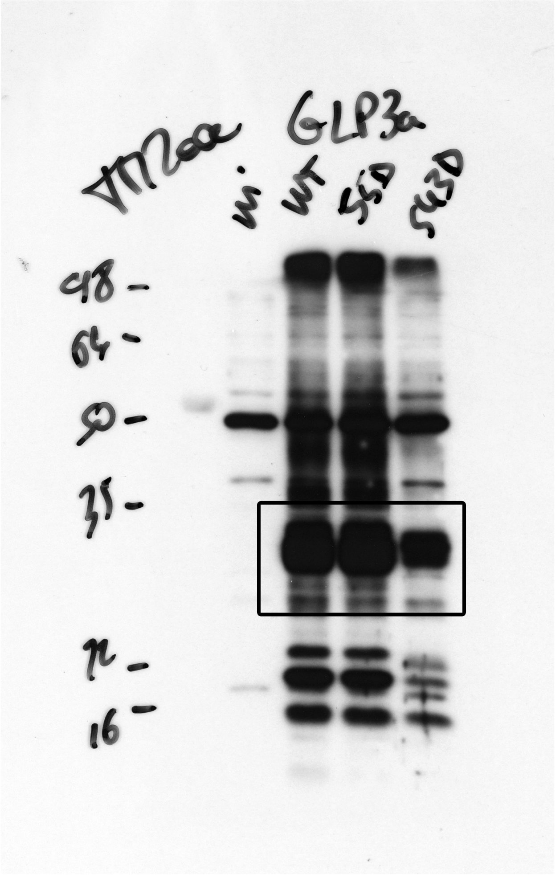

Supplementary Figure S5. Identification of PKC and PKA phosphorylation sites in *L. salmonis* Glps. (S5A-S5D): Uncropped Western blots of Fig. 8C-D and Fig. 8I, L and O in main text. Boxes illustrate regions shown in main text.
