# Supplementary material for: Lineage-level divergence of copepod glycerol transporters and the emergence of isoform-specific trafficking regulation
Source: Commun Biol. 2021 May 31;4:643. doi: 10.1038/s42003-021-01921-9 (PMC8167128; doi:10.1038/s42003-021-01921-9)
Supplement: Supplementary file 3 — Description of Additional Supplementary Files [file 42003_2021_1921_MOESM3_ESM.pdf]

Description of Additional Supplementary Files

File name: Supplementary Data 1

Description:

Supplementary File S1: Alignment for Fig. 1B

Supplementary File S2. Alignment for Fig. 1C

Supplementary File S3. Alignment for Fig. S1B

Supplementary File S4. Alignment for Fig. S1C

File name: Supplementary Data 2

Description: Original data for Figures 8, 9 and 10.
