# Supplementary material for: Lineage-level divergence of copepod glycerol transporters and the emergence of isoform-specific trafficking regulation
Source: Commun Biol. 2021 May 31;4:643. doi: 10.1038/s42003-021-01921-9 (PMC8167128; doi:10.1038/s42003-021-01921-9)
Supplement: Supplementary file 4 — Supplementary data 1 [file 42003_2021_1921_MOESM4_ESM.pdf]

Lineage-level divergence of copepod glycerol transporters and the emergence of isoform-specific trafficking regulation

Marc Catalán-García, François Chauvigné, Jon Anders Stavang, Frank Nilsen, Joan Cerdà, Roderick Nigel Finn

Supplementary data 1

Page 2: **File S1:** Alignment for Fig. 1B

Page 63: **File S2.** Alignment for Fig. 1C

Page 86: **File S3.** Alignment for Fig. S1B

Page 93: **File S4.** Alignment for Fig. S1C

File S1: Alignment for Fig. 1B

>GHDT01074380_Macrobrachium_australiense_Glp2
AAAATCATGGATTATAACCTTCGGTCTAAGTTAAAA---ATCAAAAACGTTTTGGTTCGC
GATTTTCTCTCCGAGTTTCTTGGAACTTTCATTTTAATACTCTTTGGAAACGCATCAGTT
GCCCAGATGGTCTTGACGAACAAA---------------GTCGGGAACGACGTCTTCGCC
GTCAACTGGGCATGGGGCATAGCCGTCATGTTGGCCATAATGACTAGCGGAGGGATCTCG
GGCGGGCACATCAATCCGGCGGTGACCTTGGCATTCGCCATTTGGGACAAACTG---CCC
TGGGTCAAAGTACCCGTATATATTTTGGGTCAATATGCTGGGGCCTTCTTTGCCTCTTGC
CTCGTTTACGTTGTTTATTTTGATGCTCTTCATCATTATGAA---CCATCC---------
AAG------------------------------ACTTTGGCAACGGCTGGTATTTGGGCA
ACGTACCCGGGCACCATGACAAACAATAACGGCACTATA------ATCTCTGGATTCCTG
TCTTTCGCCAATGGGTTTGGAGATCAGGTCCTTGGCACGGCTGCCCTCTTGGTCTGCGTG
TGCGCCATCTCAGACGCCAGGAACATGGAGGTGCCCAGACACCTAATTCCTCTCTCCATC
GGATTACTCGTCTCCAACATGGGTAACTGTTTCGGCTTCAATTGCGGATGTGCTCTTAAT
CCCGCCAGGGACCTCGCTCCTAGGTTCTTCACGCTGATTGCAGGGTGGGGGGACGACCCT
TTCACGGCCAGTACTCGGGAGGGCATACCTTGGTGGTGGGTGCCCATTGTTGCACCACAC
TTAGGCGCCATTTTGGGAGTAGGCATCTACGTATTGTTG---ATCGAAATGCATCACCCT
GATCTGGAAGACCAGTCTGAGAGCAGAGTCATC------
>GHDU01074007_Macrobrachium_koombooloomba_Glp2
AAAATCATGAATGACAATGTTCGGTCTAGGTTAAAA---ATTAAAAACGTTTTGGTTCGC
GATTTTCTCTCCGAGTTTCTTGGAACTTTCATTTTTATACTTTTTGGAAACGCATCAGTT
GCCCAGATGGTCTTGACGAACAAA---------------GTCGGGAACGACGTCTTCGCC
GTCAACTGGGCATGGGGCATAGCCGTCATGTTGGCCATAATGATTAGCGGAGGGATCTCG
GGCGGGCACATCAACCCGGCGGTGACCTTGGCATTCGCCATTTGGGACAAACTG---CCT
TGGGTCAAAGTACCCGTATATCTTTTGGGTCAATATGCTGGGGCCTTCTTTGCCTCTTGC
CTTGTTTACGTTGTTTATTTTGATGCTCTTCATCATTACGAA---CCATCC---------
AAG------------------------------ACTCTGGCAACGGCGGGTATTTGGGCA
ACGTACCCGGGCACCATGACAAACAATAACGGCACTATA------GTCTCTGGATTCCTG
TCTTTCGCCAATGGGTTTGGAGATCAGGTCCTTGGCATGGCTGCCCTCTTGGTCTGCGTG
TGCGCCATCACAGACGCCAGGAACATGGAGGTGCCCAGACACCTAATTCCTCTCTCCATC
GGATTACTCGTCACCAACATGGGTAACTGTTTCGGCTTCAATTGCGGATGTGCTCTTAAT
CCCGCCAGGGACCTCGCTCCTAGGTTCTTCACGCTGATTGCAGGGTGGGGGGACGATCCT
TTCACGGCCAGTACTCGGGAGGGCATACCTTGGTGGTGGGTGCCCATTGTTGCTCCACAC
TTAGGTGCCATTTTGGGAGTGGGCATCTACGTATTGTTG---ATCGAAATGCATCACCCT
GATCTGGAAGACCAGTCTGAAAGCAAAGTCATC------
>GHDQ01085220_Macrobrachium_tolmerum_Glp2
AAAATCATGAAATATGATCTTCGGTCTAAGTTAAAA---ATTAAAAACGTTTTGGCTCGC
GATTTTCTCGCCGAGTTTCTAGGAACTTTCATCTTAATACTCTTTGGAAATGCATCAATT
GCTCAAATGGTTTTGACGAACAAA---------------GTCGGGGGCGACGTCTTCGCC
GTCAACTGGGCATGGGGCATAGCCGTCATGTTGGCCATACTGACTTGCGGAGGAATTTCG
GGTGGGCACATCAACCCCGCGGTGACCTTGGCATTCGCCATTTGGGACAAACTG---CCC
TGGGTCAAAGTACCCGTTTATCTTTTGGGTCAATATGCTGGGGCATTCGTCGCCTCTTGC
CTTGTGTATGTTGTTTATTTTGATGCTCTTCATCATTATGAA---CCATCC---------
AGG------------------------------AGTCTGGCAACGGCGGGTATCTGGGCA
ACGTACCCGGGCAGTATGACAAACAGTAACGGCACGATA------GTCTCTGGATTCCTT
TCTTTCGCCAATGGGTTTGGAGATCAGGTCCTTGGTACGGCTGCCCTCTTGGTCTGCGTG
TGCGCCATCGTAGACGCCAGGAACATGGAGGTGCCAAAGCACCTAATCCCTCTCTCCGTC
GGATTACTCGTTGTCAACATGGGGAACTGTTTCGGCTTCAATTGCGGATGTGCTCTTAAT
CCCGCCAGGGACCTCGCTCCTAGGTTCTTCACGCTGATTGCAGGATGGGGGGACGATCCT
TTCACGGCCAGCACTCGGGAGGGCATACCTTGGTGGTGGGTGCCCATAGTTGCTCCACAC
ATAGGCGCCATTTTGGGAGTGGGCATCTACGTCTTGTTC---ATTGAAATGCATCACCCT
GATCTGGAAGACCAGTCTGGAAGCAAAGTCATC------
>GHMG01011681_Macrobrachium_nipponense_Glp2
AGAATCAAGGAATATAATCTTCGGTCAAAGTTGAAG---ATAAAAAACGCTTTGGTTCGC
GATTTTATCGCCGAGTTTCTTGGAACTTTCGTCCTAATACTCTTTGGAAATGCGTCCATT
GCCCAGATGGTCTTGACGAACAAA---------------GTCGGGGGCGACGTCTTCGCC
GTCAACTGGGCATGGGGCATCGCCGTCATGTTGGGTATATTGACTAGTGGAGGGATCTCG
GGCGGGCACATCAACCCGGCAGTGACCTTGGCATTCGCCATTTGGGACAGACTG---CCC
TGGTTCAAAGTACCCGTTTATCTTCTGGGTCAATATGCTGGGGCTTTCGTTGCCTCTTGC
CTTGTTTATGCTGTTTATTTTGATGCTCTTCATCATTACGAG---CCATCC---------
AGG------------------------------AGTCTGGCAACGGCTGGTATCTGGGCA
ACGTACCCGGGCAGTATGACGAACAACAACGGCACGATA------GTCTCCGGATTCCTT
TCTTTCGCCAATGGGTTTGGAGATCAGGTGCTGGGTACGGCTACCCTCTTGTTCTGCGTG
TGTGCCATCACAGACTCCAGGAACATGGAGGTGCCCAAGCACCTGATCCCTCTCTCCATC
GGATTGCTTGTCTTCAACATGGGGAACTGCTTTGGCTTCAACTGCGGATGCGCTCTCAGT
CCCGCCAGGGACCTCGCTCCGAGGCTCTTCACGCTGATTGCTGGATGGGGGGAGGACCCC
TTCACGGCCAGTACCCGGGAGGGGATACCGTGGTGGTGGGTGCCCGTAGTCGCTCCACAC
ATAGGCGCCATTTTGGGAGTGGGCATTTATGTATTGTTC---ATCGAAATGCATCACCCT

GACCTGGAAGACCAATCTGAAAGCCAGGTCACC------
>GHDW01094118_Macrobrachium_novaehollandiae_Glp2
AAAATCAGGGAATATAATCTTCGGTCAAAGATGAAG---ATAAAAAACGCTTTGTTTCGC
GATTTTCTCGCCGAGTTTCTTGGAACTTTCATCTTAATACTCTTTGGAAATGCATCCGTT
GCCCAGATGGTCTTGACGAACAAA---------------GTCGGGGGCGACGTCTTCGCC
GTCAATTGGGCATGGGGCATCGCCGTCATGTTGGGTATACTGACTAGCGGAGGGATTTCG
GAGGGGCACATCAACCCGGCGGTGACCTTGGCATTCGCCATCTGGGACAGACTG---CCC
TGGTTCAAAGTACCCGTTTATCTTCTGGGTCAATATGCTGGGGCCTTCGTTGCCTCTTGC
CTTGTTTATGCTGTTTATTTGATGCTCTTCATCATTACGAG---TCATCC---------
AAG------------------------------AGTCTGGCGACGGCGGGTATCTGGGCA
ACGTACCCGGGCAGCATGACGAACAACAACGGCACGATC------GTCTCTGGATTCCTT
TCTTTCGCCAACGGGTTCGGAGATCAGGTCCTGGGTACGGCTACCCTCTTGTTCTGCGTG
TGCGCCATCATAGACTCCAGGAACATGGGTTTACCCCGCCACATGATCCCTCTCTCCATC
GGATTACTTGTTTTCAACATGGGGAACTGCTTCGGCTTCAACTGCGGATGCGCCCTCAGC
CCCGCCAGGGACCTCGCTCCGAGGTTCTTCACGCTGATTGCAGGGTGGGGAGACGACCCC
TTCACGGCCAGTACCCGGGAGGGCATACCGTGGTGGTGGGTGCCCGTAGTCGCTCCACAC
ATAGGTGCCATTTTGGGAGTGGGCATCTACGTATTGTTC---ATCGAAATGCATCATCCC
GACCTGGAAGACCAGTCTGAAAGTCAGGCCATC------
>GFPG01014147_Palaemon_varians_Glp2
------ATGGAATCCAATCTTCGTGCAAAGTTGAAA---GTTGAAAATGTTTTGGTTCGA
GATTTCCTCGCGGAATTTCTTGGGACTTTTGTCTTGATACTGTTTGGAAATGCCTCCGTC
GCTCAAATGGTTCTGACAAACAAA---------------ATCGGAGGTGACGTATTTGCC
GTCAACTGGGCTTGGGGTATTGCTGTCATGCTGGCTATATTCACGAGTGGAGGGGTATCA
GGAGGGCACATCAACCCGGCCGTGACCTTGGCCTTCGCCGTTTGGGATAAGCTC---CCC
TGGGTCAAAGTGCCTGTGTATTTTCTGGGTCAATATGCCGGGGCATTTGTGGCCTCTTGT
TTGGTTTACGTTGTGTATTTTGACGCCCTTCATCATTATGAG---TCAACT---------
AGG------------------------------AGTCTATCGACGGCGGGTATTTGGGCA
ACGTACCCAGGTAGTATGACCAATATCAATGGCACCGTT------GTCACCGGATTCCTC
TCTGGCGCCAACGGCTTTGGGGACCAGGTCCTCGCAACGGCTGCCCTCATGATCTGCGTC
TGCGCCATCACGGACGCCAGGAACATGGAGTTGCCCAAATACCTGATCCCTCTCTCCGTC
GGTCTGCTGGTCTTCAACATGGGCAGCTGCTTCGGGTTCAACTGCGGATGTGCCCTCAAT
CCTGCCAGGGATCTCGCCCCTAGGATCTTCACGCTCTTGGCAGGATGGGGCGACGCCCCT
TTCACGGCCAGCACTCGAGAGGGTATGCCATGGTGGTGGGTGCCAATCGTAGCTCCTCAC
ATAGGGGCCATTTTCGGAGTGGGCGTTTACGTATTGTTC---ATTGAACTTCATCATCCT
GACCTTCAAGAAGATTCTCAAAGTGAAATGATT------
>QUOF017053917_Palaemon_carinicauda_Glp2
---------------------------AGGATGAAA---ATCGAAAAGGTTTCCGTCAAA
GAATTTCTTAGTGAATTTCTTGGCACATTCATTTTGGTGCTGTTTGGAAATGCATCCGTT
GCCCAGATGGTTTTGACCAACAAA---------------GTCGGAGGAGACGTGTTTGCC
GTTAACTTGGCATGGGGCATCGCGGTCATGCTGGCTATAATGACGAGCGGAGGGGTATCG
GGTGGGCATATCAACCCAGCCGTGACCTTGGCCTTCGCCGTTTGGGGTAAACTA---CCC
TGGGTCAAAGTGCCCGTGTATTTTCTGGGGCAATATGCTGGGGCATTTGTCGCTTCGTGT
TTGGTCTACGTTGTATAT---GACGCTCTTCATCACTACGAG---CCAGCC---------
AGG------------------------------AGTCTTGCCACAGCGGGCATCTGGGCA
ACGTACCCAGGAACTATGTCCAATATCAATGGCACTGTT------GTCAACGGATTCCTT
TCTGGCGTCAATGGGTTTGGGGACCAGGTAGTCGGCACTATGCTACTTCTGATCTGCGTG
TGTGCCATTACGGACACCCGGAACGTTGGCGTACCTCAGTATCTTGTGCCTCTTTATGTG
GGATTTGCCGTTCTGAACATCAACAACTGCTTTGGACTGAACTGTTCTTGCGCCATCAAC
CCTGCCAGAGATTTTGCCCCGAGGCTCTTCACCTTCATCGCAGGATGGGGCACAGACCCC
TTCGCGGCCAGCACACGCGAGGGTATGCCGTGGTGGTGGGTACCAATCGTAGCCCCACAC
ATAGGGGCCATTCTGGGAGTGGGTATTTACGTACTATTC---ATCGAACTCCATCACCCA
GACCTTCGAGGAGAGTCTCAGAGTGAAGTGATC------
>GHOJ01050430_Lysmata_amboinensis_Glp1

------------------TATATGGCAGGCCAATACGTCGGGGCATTCGTGGCATCATGC
GTGGTCTACGCTGTTTATATCAATGCTCTAGACCACTTCGAA---GCGACT---------
AAG------------------------------AGCATCGCGACGGCGGGCATCTGGGCG
ACATACCCTGGCACCATGACAAGTGCCAATGGCACAGTC------ATATCTGATTTCCTA
TCTAATACCAACGGCCTCGTAGATCAGGTGATCGGCACA---------------------

------------------------AACTGCTTCGGATTCAATTGTGGCTGTGCAATCAAT
CCAGCCAGGGACTTAGCGCCTAGGATCTTCACATATGTTGCAGGGTGGGGAGATGTCCCC
TTTGGTGCCAGTACCCGAGAGGGCATAGCGTGGTGGTGGGTGCCTATAGTAGGTCCTCAT
ATAGGCGCAGTTTTGGGAGTTGGCGTTTACGTCGTATGC---GTCGAATTACATCATCAC

GACGTCGAT------------------------------
>IABX01119952_Caridina_multidentata_Glp1

---------------------------------------CTATTCGGTGATGCTTCCGTA
GCCCAAAGTGTCTTGACTAACAAT---------------GCCAATGGAGATTTTTTCGCT
GTCAACTGGGGCTGGGCATTGGCGGTCGTATTGGCTGTTCTGGTCAGCGGTGGGGTCAGC
GGAGGTCATGTCAACCCTGCCGTGACCTTGGCTATGGCCATCTGGGGCAAACAT---CCT
TGGGTCAAAGTGCCAGTCTATATGGTAGCCCAGTACGTCGGAGCATTTTTCGCGTCCTGT
CTCGTGTATGGGGTCTATATAGATGCCCTGAACGCCTTCGAG---CCCTAC---------
AGG------------------------------AGCATGGCAACAGCGGGTATCTGGGCC
ACATACCCCGGAACGATGACCAACGTCAATGGAACTGCC------GTCTACAACTTCCTC
TCCAGCGGTAATGGTTTCGCTGATCAGATCGTTGGCACAGCCGTCCTTTTAATCTGCGTC
TGTGCCATAACCGACCCTCGTAACATGGAAGTCCCCAAGTATCTTGTTCCCCTCTTCGTC
GGCTTCACCGTTCTCAACATTGGCATCTGCTTTGGATTTAACTGTGGCTATGCCATTAAC
CCAGCCAGGGATCTATCTCCTAGACTCTTCACACTCATTGCAGGATGGGGAGAGGCTACT
TTCCAAGCCAGTACACTGGAGGGCATAGCATGGTGGTGGGTGCCACTAGTCGGACCACAC
ATCGGTGCCATCCTGGGTGTCGCAATATACCTTGTCTTG---ATCGAACTACATCACCCT
GAAGCCAAGGACTTTGATTTACCCCAG------------
>GGXN01034581_Neocaridina_denticulata_Glp1
AATAAAATCATGGAAGCCCTACAGTCCAGAGTTCGA---GTCAAGAACGTTTTGGTGAGA
GAATTTCTCGCCGAGTTCTTTGGAACTTTCATCTTAGTGCTTTTCGGCGATGCCTCCGTA
GCCCAGAGTGTCTTGACCAACAAT---------------GCCAACGGTGACTTCTTCTCT
GTTAACTGGGGCTGGGGACTGGCAGTCGTCTTGGCAGTGCTGGTCAGCGGCGGGGTCAGC
GGAGGTCATGTCAATCCCGCCGTGACCTTGGCTATGGCCGTCTGGGGCAAGCAT---CCT
TGGGTCAAAGTGCCTGTCTATATGGTTGCGCAGTACGTGGGAGCATTCTTCGCTTCCTGT
ATTGTGTATGGTATATATATAGATGCCCTGAATGCCTACGAA---CCCTAC---------
CGT------------------------------AGTATGGCAACGGCAGGCATCTGGTCG
ACATACCCTGGTACAATGACCAACATCAATGGCACCGCC------GTCTACAATTTCCTC
TCCAGTGGCAATGGATTCGCAGATCAGGTTGTAGGTACAGCTGTCCTCTTGATCTGCGTG
TGTGCCATAACCGACCGTCGTAACATGGAGGTTCCCAAGTATCTTATTCCTCTCTTCGTT
GGCTTCACTGTCCTCAACATTGGCATCTGCTTTGGATTCAACTGTGGTTATGCCATTAAC
CCAGCCAGAGACTTGTCCCCTAGACTCTTTACGCTTATTGCAGGATGGGGAGAGGCTACT
TTCCAAGCCAGTACACTCGAGGGCTTTGCATGGTGGTGGGTGCCAATTGTCGCACCACAC
ATCGGTGCCATCCTGGGTGTGGCAATATACCTCGTCCTG---ATCGAACTGCATCATCCT
GAAGCTAAGGAGTTTGACTTACCCCAGATACAC------
>GHDT01086109_Macrobrachium_australiense_Glp1
AACAAAGTCGTGGAAACTCTTCAGTCGAAGATCCGA---CTCAGGAACGTCGTCGTCAGG
GAGTTCCTGGCCGAGTTCCTTGGCACGTTCATTCTCGTGCTCTTCGGGGACGCATCGGTG
GCACAAAGCGTCCTGACGAACAAA---------------GCCAATGGAGATTTCTTCGCC
ATCAACTGGGGATGGGGCCTGGCTGTTGTCTTAGGCGTCCTCGTAAGTGGAGGGGTGAGC
GGAGGTCACATCAATCCAGCCGTGACCTTAGCCATGGCCATCTGGGGGAAGCAC---CCT
TGGGCAAAAGTGCCCGTTTATATGGTGGCACAGTATCTGGGGGCGTTCTTCGCGTCGTGT
ATGGTCTATGCTGTCTATCTCAATGCCCTGGACGCCTTCGAG---CCAGAG---------
AAA------------------------------AGTTTGGCAACAGCTGGAATCTGGGCA
ACGTACCCAGGAGCTATGACTAATATCAATGGCACCGTT------ATCACAGATTTCTTG
TCCAGTGGAAATGGCTTGGCTGATCAGGTTGTGGGCACCATGCTGCTGCTGATCTGCGTC
TGCGCCATCACAGACTCTAGGAACATGGAGGTGCCCAAGTACTTGATTCCTCTCTTCGTC
GGCTTCACCGTCGTGAACATCGGCATCTGCTTCGGCTTCAACTGCGGCTACGCCATCAAC
CCCGCCAGGGACCTGGCACCCAGGCTCTTCACCCTCATTGCCGGATGGGGAGGCGATACG
TTCAAAGCGAGCACCTTCGAGGGAATTGTCTGGTGGTGGGTGCCCATTGTTGGCCCACAC
ATCGGAGCGGTGTTGGGCGTGGCGATATACCTGGCGCTG---ATCGAACTCCATCACCCG
GAGTCACAGGAGTTCTCCCTTCCACAGATCCAC------
>GHDU01050380_Macrobrachium_koombooloomba_Glp1
AACAAAGTCGTGGAAACTCTTCAGTCGAAGATTCGA---CTCAGGAACGTCGTCGTCAGG
GAGTTCCTGGCCGAGTTCCTTGGCACGTTCATTCTCGTGCTCTTTGGGGACGCATCGGTG
GCACAAAGCGTTCTGACGAACAAA---------------GCCAATGGAGATTTCTTCGCT
ATCAACTGGGGATGGGGCCTGGCTGTTGTCTTAGGCGTCCTCGTAAGTGGAGGGGTGAGC
GGAGGTCACATCAATCCAGCCGTGACCTTAGCCATGGCCATCTGGGGGAAGCAC---CCT
TGGGCGAAAGTGCCCGTTTATATGGTGGCACAGTATCTGGGGGCGTTCTTCGCGTCGTGT
ATGGTCTATGCTGTCTATCTCAATGCCCTGGACGCCTTCGAG---CCAGAG---------
AAA------------------------------AGTTTGGCAACAGCTGGAATCTGGGCA
ACGTACCCAGGAGCTATGACTAATATCAATGGCACCGTT------ATCACAGATTTCTTG
TCCAGTGGAAATGGCTTGGCTGATCAGGTTGTGGGCACCATGCTGCTGCTGATCTGCGTC
TGTGCCATCACAGACTCTAGGAACATGGAGGTGCCCAAGTACTTGATTCCTCTTTTCGTC
GGCTTCACCGTCGTGAACATCGGCATCTGCTTCGGCTTCAACTGCGGCTACGCCATCAAC
CCCGCCAGGGACCTGGCACCCAGACTCTTCACCCTCATTGCCGGATGGGGAGGCGATACG
TTCAAAGCGAGCACCTTCGAGGGAATTGTCTGGTGGTGGGTGCCCATTGTTGGCCCACAC
ATTGGAGCGGTGTTGGGCGTGGCGATATACCTGGCGCTG---ATCGAACTCCACCACCCG

GAGTCACAGGAGTTCTCCCTTCCACAGATCCAC------
>GHDQ01097485_Macrobrachium_tolmerum_Glp1
AACAAAGTCGTGGAAACTCTCCAGTCGAAGATTCGA---CTCAGGAACGTCGTCGTCAGG
GAGTTCCTGGCCGAGTTCCTTGGCACGTTCATTCTCGTGCTCTTTGGGGACGCATCTGTG
GCACAAAGCGTCCTGACGAACAAA---------------GCCAATGGAGATTTCTTCGCC
ATCAACTGGGGATGGGGCTTTGCTGTTGTCTTAGGCGTCCTCGTGAGTGGAGGGGTGAGC
GGAGGTCACATCAATCCAGCTGTGACCTTAGCCATGGCCATCTGGGGGAAGCAC---CCT
TGGGCGAAAGTGCCCGTTTATATGGTGGCACAGTATCTGGGGGCGTTCTTCGCGTCGTGT
ATGGTCTATGCTGTCTATCTCAATGCCCTGAACGCCTTCGAG---CCAGAG---------
AAA------------------------------AGTTTGGCAACAGCTGGAATCTGGGCA
ACGTACCCAGGAGCTATGACTAATATCAATGGCACCGTT------ATCACAGATTTCTTG
TCCAGTGGAAATGGCTTGGCTGATCAGGTTGTGGGCACTATGCTTCTGCTGATCTGCGTC
TGCGCCATCACAGACTCGAGGAACATGGAGGTGCCCAAGTACTTGATCCCTCTCTTCGTC
GGCTTCACCGTCGTGAACATCGGCATCTGCTTCGGCTTCAACTGCGGCTACGCCATCAAC
CCGGCCAGGGACCTGGCACCCAGACTCTTCACCCTCATTGCCGGATGGGGAGGCGATACG
TTCCAAGCGAGCACCTTCGAGGGAATTGTCTGGTGGTGGGTGCCCATCGTTGGCCCTCAC
ATCGGAGCGGTGTTGGGCGTGGCGATATACCTGGCGCTG---ATCGAACTCCACCACCCG
GAGTCACAGGAGTTCTCCCTTCCACAGATCCAC------
>GCVG01007095_Macrobrachium_nipponense_Glp1
AACAAAGTCGTGGAAACTCTTCAGTCGAAGATTCGA---CTGAGGAACGTCGTCGTCAGG
GAGTTCCTGGCCGAGTTCCTTGGCACGTTCATTCTCGTGCTCTTTGGGGACGCATCGGTG
GCACAAAGCGTCCTGACGAACAAA---------------GCCAACGGAGATTTCTTCGCC
ATCAACTGGGGATGGGGCCTGGCCGTTGTCTTAGGAGTCCTCGTCAGTGGAGGGGTGAGC
GGAGGTCACATCAACCCAGCTGTGACCTTAGCCATGGCCATCTGGGGGAAGCAC---CCG
TGGGCGAAAGTTCCCGTTTATATGGTGGCACAGTATCTGGGGGCGTTCTTCGCGTCATGT
ATGGTCTATGCTGTCTATCTCAATGCCCTGGACGCCTTCGAG---CCACAG---------
AAA------------------------------AGTTTGGCAACGGCTGGAATCTGGGCA
ACGTACCCAGGAGCGATGACTAATGTCAATGGCACCGTT------ATCACAGACTTCCTG
TCCAGTGGGAATGGTTTAGCTGATCAGGTTGTGGGCACCATGCTGCTACTGATCTGCGTC
TGCGCCATCACCGACTCTAGGAACATGGAGGTGCCCAAGTACTTGATTCCTCTCTTCGTC
GGCTTCACCGTCGTGAACATCGGCATCTGCTTCGGCTTCAACTGCGGTTACGCCATTAAC
CCCGCCAGGGACCTGGCGCCCAGACTCTTCACCCTCATTGCCGGATGGGGAGGCGACACC
TTCAAAGCTAGCACCTTCGAGGGAATTGTGTGGTGGTGGGTGCCCATCGTTGGCCCACAC
ATCGGAGCGGTGTTGGGCGTGGCGATATACCTGGCGCTG---GTCGAACTCCACCACCCG
GAGGCCCAAGAGTTCGCCCTTCCTCAGATCCAC------
>GHDW01101842_Macrobrachium_novaehollandiae_Glp1
AACAAAGTCGTAGAAGCTCTTCAGTCGAAAATTCGA---CTGAGGAACGTCGTCGTCAGG
GAGTTCCTGGCCGAGTTCCTTGGCACGTTCATTCTCGTGCTCTTTGGAGACGCCTCGGTT
GCACAAAGCGTCCTGACGAACAAT---------------GCCAAAGCAGATTTCTTCGCC
ATCAACTGGGGATGGGGTCTGGCCGTTGTCTTAGGAGTCCTCGTCAGTGGAGGGGTGAGC
GGAGGTCACATCAATCCAGCAGTGACCTTAGCCATGGCCATCTGGGGGAAGCAC---CCA
TGGGCGAAAGTTCCCGTTTATATGGTGGCGCAGTATCTGGGGGCGTTCTTCGCGTCGTGT
ATGGTCTATGCCGTCTATCTCAATGCCCTGGACGCCTTCGAT---TCAGGG---------
AAA------------------------------ACTTTGGCGACAGCTGGAATCTGGGCA
ACGTACCCAGGAGAGATGGTTAACGCCAATGAGACCGTT------ATCAAAAATTTCCTG
TCTAGTGGGAATGGTTTGGCTGATCAGGTTGTGGGCACAATGCTGCTACTGATCTGCGTC
TGCGCCATCACAGACTCTAGGAACATGGAGGTGCCCAAGTACTTGGTCCCTCTCTTCGTC
GGCTTCACCGTCGTGAACATCGGCATCTGTTTTGGTTTCAACTGCGGCTACGCCATCAAC
CCCGCCAGGGACCTGGCCCCTAGACTCTTCACCCTCATTGCCGGATGGGGAAGCGATACG
TTCACAGCGAGCACCTCCGATGGAATTGCCTGGTGGTGGGTGCCCATTGTTGGCCCACAC
ATCGGAGCTGTGTTGGGCGTGGCGATATACCTGGCGCTG---GTCGAGCTCCACCACCCG
GAGTCCCAGGAGTTCGCCCTTCCTCAGATCCAC------
>GFPG01006513_Palaemon_varians_Glp1
AACAAAGTCGTGGAGACCCTCCAGTCGAAGATTCGC---CTCAGGAACGTCATCGTCAGG
GAGTTCCTGGCTGAGTTCCTTGGCACGTTCATCCTCGTGCTCTTTGGAGACGCATCAGTC
GCGCAAAGCGTCTTGACCAACAAA---------------GCCAATGGGGACTTCTTCGCC
ATCAACTGGGGATGGGGTTTGCGGTCGTCCTCGGCGTCCTCGTAAGCGGAGGGGTCAGC
GGAGGTCACATCAATCCAGCCGTGACCTTAGCCATGGCCATCTGGGGCAAGCAT---CCC
TGGGCAAAAGTGCCTGTTTATATGGTGGCACAGTACCTGGGGGCGTTCTTCGCGTCTTGT
ATGGTCTATGCAGTTTATCTCAATGCCCTGGACGCCTTCGAG---CCAACG---------
AGA------------------------------AGTTTGGCAACAGCTGGAATCTGGGCA
ACGTACCCAGGGGCGATGACCAATGTCAATGGGACAGTG------CTCACTGATTTCCTG
TCCAGTGGCAATGGCTTGGCTGATCAGGTGGTAGGCACAATGTTGCTGCTGATCTGCGTC
TGCGCCATCACCGACCCCCGGAACATGGAGGTGCCCAAGTACCTGGTTCCTCTCTTCGTC
GGCTTCGCCGTCGTGAACATTGGCATCTGCTTCGGCTTCAACTGCGGCTACGCCATTAAC
CCGGCCAGGGACCTCGCCCCCAGACTCTTCACTCTCATTGCTGGATGGGGAAGCAGCACA
TTCACAGCGAGCACCTTCGAGGGCATTGTGTGGTGGTGGGTGCCCATCGTGGGCCCACAC
ATAGGGGCCATTTTGGGCGTGGCCATATACCTGGCTCTG---ATCGAACTCCACCACCCG

GAGTCGCAGGAGTTCTCCCTTCCACAGATCCAC------
>QUOF015718519_Palaemon_carinicauda_Glp1
------------------------------ATTCGA---CTCAGGAACGTTGTCGTCAGG
GAGTTCCTGGCCGAGTTCCTTGGGACGTTCATCCTCGTGCTCTTCGGAGACGCCTCAGTA
GCGCAAAGCGTCCTGACCAACAAA---------------GCCAATGGGGATTTCTTCGCC
ATCAACTGGGGATGGGGCTTTGCCGTTGTCTTAGGTGTCCTTGTGAGCGGAGGGGTCAGC
GGAGGTCACATCAATCCAGCCGTGACCTTAGCCATGGCCATCTGGGGCAAGCAT---CCC
TGGGCAAAAGTGCCCGTTTATATGGTGGCGCAGTACCTGGGGGCCTTCTTCGCGTCGTGC
ATGGTCTATGCAGTTTATCTCGATGCCCTGGACGCCTTCGAG---CCAACG---------
CGA------------------------------AGTATGGCAACAGCGGGAATCTGGGCT
ACGTACCCGGGGGCGATGACCAATGCCAATGGACCGTG------CTCACAGATTTCCTG
TCGAGTGGGAATGGATTGGCTGATCAG---GTAGGCACAATGCTGCTTCTAATCTGCGTC
TGCGCCATCACCGACCCAAGGAACATGGAGGTGCCCAAGTACCTAGTCCCTCTCTTCGTC
GGCTTCGCCGTCGTGAACATCGGCATCTGCTTCGGTTTCAACTGCGGTTACGCCATCAAC
CCAGCCAGGGATCTTGCGCCCAGACTGTTTACGCTCATTGCCGGGTGGGGAAGCAGCACA
TTCACGGCGAGCACCTTCGAGGGCATTGTATGGTGGTGGGTGCCCATCGTGGGCCCACAC
ATAGGCGCCATCTTGGGCGTGGCGATCTACCTGGCCCTG---ATCGAACTGCATCACCCG
GAGTCACAGGAGTTCTCCCTTCCGCAAATCCAC------
>GHAP01054284_Metabetaeus_lohena_Glp1
AACAAAATAGTTGAGACTTTGCAGTCCAAGTTGCGC---CTGAAAAACGCCATCGTCAGG
GAATTTTTAGCTGAATTCCTTGGAACGTTTATACTAGTGCTCTTTGGTGACGCCTCTGTA
GCTCAAAGCGTCTTGACCAACAAG---------------GCCAATGGTGACTTTTTTGCC
ATCAACTGGGGCTGGGGCCTGGCTGTCGTCCTCGGGGTTCTCATTAGCGGAGGGGTCAGC
GGAGGGCACATCAATCCTGCCGTGACCTTAGCTATGGCCGTCTGGGGCAAACAC---CCT
TGGGTGAAAGTCCCCATCTACATGGTAGCCCAGTACCTAGGGGCTTTCTTAGCATCTTGT
ATGGTCTATGCTGTATATCTCAATGCCTTAAACACGTTTGAG---CCCGCC---------
CGA------------------------------AGTTTGGCCACAGCTGGTATCTGGGCT
ACATACCCGGGCGCTATGACCAATGTTAACGGAACAGTC------GTCACAAATTTCCTC
TCCAGCGGCAATGGTCTGGCTGATCAAATTGTAGGAACAATGCTGCTCCTGATTTGTGTG
TGCGCCATCACTGACCCACGAAACATGGAAGTGCCCAAGTACCTCATCCCTCTCTTTGTC
GGATTCACTGTCATTAATATCGGTATCTGCTTTGGATTCAACTGTGGCTACGCCATTAAC
CCCGCCAGGGATCTTGGTCCCAGGCTCTTCACTCTTATTGCTGGATGGGGTGGACAAACT
TTCACAGCCAGTACCTTTGAAGGCATCGTTTGGTGGTGGGTACCTATTGTAGGCCCCCAC
ATTGGAGCCATACTGGGAGTTGCCATCTACTTAGCGCTG---GTTGAACTCCACCATCCA
GAGGCGCAAGATTTTTCTCTACCACAACTCCAC------
>GGTN01012848_Penaeus_merguiensis_Glp

------------GGCATGGGCGATCAGGTGGTGGGCACGATGCTGCTGCTGATCTGCGTC
TGCGCCATCACGGATCCGCGCAACATGGAGGTCCCTAAGGCCCTGATTCCTCTCTTCGTG
GGCTTCACCGTCCTCAACATCGGCGTGTGCTTCGGCTTCAACTGCGGCTACGCTCTCAAC
CCCGCCAGGGATCTCGCCCCGCGCTTCTTCACGCTCATCGCGGGCTGGGGCGAGCAGACC
TTCACCGCCGCCACCTTCGAAGGCATCGCCTGGTGGTGGGTGCCCCTCCTGGCCCCCCAC
ATTGGCGCTGTGATAGGCGTGGGCGTGTACCTCCTGCTG---GTCGAGCTGCACCACCCA
GAGGCCCAGGAGTTCTCCCTCCCACACGTCCAC------
>GFRT01000419_Penaeus_penicillatus_Glp
AACAAGGTGGTCGAGATGCTGCAGTCCAGATTGAGG---CTCAGGAACCCGATGTTTCGA
GAATTTCTGGCGGAATTTCTCGGCACCTTCATCCTGGTGATCTTCGGCGACGGTTCCGTA
GCGCAGAAGGTCCTGACCAACAAC---------------GCCAACGGGGACTTCTTCTCG
GTCAACTGGGGCTGGGGAGTGGCCGTGACCTTGGGCGTGCTCGTGTCAGGCGGGGTCAGC
GGAGGTCACATCAACCCCGCCGTGACCGTGGCCATGGCTGTGTGGGGCAAGCAT---CCC
TGGGTCAAAGTGCCTGTGTATGTTATTGCCCAGTACCTCGGAGCATTCCTGGCGTCGTCG
GTGCTCTACGCAGTTTACTTGAACGCCTTGGACGCCTTCGAG---GCGGAG---------
CGC------------------------------ACCCTGGCGACGGCGGGCATCTTCGCC
ACGTACCCCGGGGCGATGACAGCCGCCAACGGACGGTC------GTGACGGACTTCCTG
TCGAATGGCAACGGCATGGGCGATCAGGTGGTGGGCACGATGCTGCTGCTGATCTGCGTC
TGCGCCATCACGGACCCGCGCAACATGGAGGTTCCTAAGGCCCTGATTCCTCTCTTCGTG
GGCTTCACCGTCCTCAACATCGGCGTGTGCTTCGGCTTCAACTGCGGCTATGCTATCAAC
CCCGCCAGGGATCTCGCCCCGCGCTTCTTCACGCTCATCGCGGGCTGGGGCGAGCAGACC
TTCACCGCCGCCACCTTCGAGGGCATCGCCTGGTGGTGGGTGCCTCTCCTGGCCCCCCAC
ATCGGCGCTGTGCTCGGCGTGGGCGTGTACCTCCTCCTG---GTCGAGCTGCACCACCCA

GAGGCCCAGGAGTTCTCCCTCCCACACGTCCAC------
>GGLH01054426_Penaeus_monodon_Glp
AACAAGGTGATGGAGACGCTGCAGTCCAAAATGCGG---CTCAGGAACCCCATGTTTCGG
GAATTCTTGGCGGAATTTCTCGGCACCTTCATTCTGGTGATCTTCGGCGACGCCTCCGTA
GCGCAGAAGGTCCTGACCAATGAC---------------GCCAACGGGGACTTCTTCTCG
GTCAACTGGGGCTGGGGAGTGGCCGTGACCTTGGGCGTGCTCGTGTCGGGCGGGGTCAGC
GGAGGTCACATCAACCCCGCCGTGACCGTGGCCATGGCTGTGTGGGGCAAGCAT---CCC
TGGGTCAAAGTGCCTGTGTATGTTATTGCCCAGTACCTCGGGGCATTCCTGGCGTCGTCG
GTGCTCTACGCAGTTTACTTGAACGCCCTGGACGCCTTCGAG---GCGGAG---------
CGC------------------------------ACCCTGGCGACGGCGGGCATCTTCGCC
ACGTACCCCGGGGCGATGACGGCCGCCAACGGGACCGTC------TTGACGGACTTCCTG
TCGAACGGCAACGGCATGGGCGATCAGGTGGTGGGCACGATGCTGCTGCTGATCTGCGTG
TGCGCCATCACGGACCCCCGCAACATGGAGGTTCCTAAGGCCCTGATTCCTCTCTTCGTG
GGCTTCACCGTCCTCAACATCGGCGTGTGCTTCGGCTTCAACTGCGGCTATGCTATCAAC
CCTGCCAGGGATCTCGCCCCGCGCTTCTTCACTCTCATCGCGGGCTGGGGCGAGCAGACC
TTCACCGCCGCCACCATCGACGGCATCGCCTGGTGGTGGGTGCCCCTCGTGGCTCCTCAC
ATCGGCGCTGTGATGGGCGTGGGCGTGTACCTTCTTCTG---GTCGAACTGCACCACCCA
GAGGCCCAGGAGTTCTCCCTGCCACACGTCCAC------
>NIUR012166363_Penaeus_japonicus_Glp
AATAAAGTGGTCGAAACGCTTCAATCCAAAATGAGG---CTTCGGAACCCCATGTTTCGA
GAATTCTTGGCGGAATTTCTCGGCACATTCATTCTGGTGCTCTTCGGCGACGCATCAGTA
GCGCAGAAGGTCCTGACCAACGAT---------------GCCAATGGGGACTTCTTCTCG
GTCAACTGGGGTTGGGGCATAGCCGTGACCTTGGGCGTGCTCGTATCTGGTGGGGTCAGC
GGAGGTCACATCAACCCCGCCGTGACCGTGGCCATGGCTGTGTGGGGCAAGCAT---CCC
TGGGTCAAGGTGCCCGTGTATGTTATTGCCCAGTACCTTGGAGGGTTCTTGGCATCGGCG
GTGCTCTACGGAGTTTACTTGGACGCCCTGGACGCGTACGAG---GCCGAG---------
CGG------------------------------ACGCTGGCGACGGCGGGCATCTTCGCC
ACGTACCCCGGGGCGATGACGGCGGCCAACGGGACCCTC------GTGACGGAGTTCCTC
TCGAATGGCAACGGCATGGGGGATCAG---------------------------------

---AGCGCTGCCACCATCGACGGGGTCGCCTGGTGGTGGGTGCCCGTCGTGGCCCCCCAC
ATCGGCGCTGTGATGGGCGTGGGCATCTATCTTCTCCTT---GTGGAGCTCCACCACCCC
GAGGCCCAGGAGTTCTCCCTCCCGCACGTCCAT------
>GGKO01009721_Penaeus_vannamei_Glp
AACAAAGTGGTCGAAATGCTTCAGTCCAAGCTGAGG---CTCCGGAACCCCATGTTTCGA
GAATTCTTGGCGGAATTTCTCGGCACTTTTATCTTGGTGATCTTCGGCGACGCCTCCGTA
GCGCAAAAGGTCCTGACCAAAGAT---------------GCCAACGGTGACTTCTTCTCG
GTCAACTGGGGCTGGGGAGTAGCCGTGACCCTGGGGGTGCTCGTGTCAGGCGGGGTCAGC
GGGGGTCACATCAACCCCGCCGTGACCGTTGCCATGGCTGTGTGGGGCAAGCAT---CCC
TGGGTCAAAGTGCCTGTGTATGTTATTGCCCAGTACCTCGGGGCGTTCATGGCGTCGTCG
ATGGTCTACGCAGTTTACTTGAACGCCCTGGACGCCTTCGAG---CCGGAG---------
CGC------------------------------AGCCTGGCGACGGCGGGCATCTTCGCC
ACGTACCCCGGGGCAATGACGGCCTCCAACGGGACCCTC------GTGACGGACTTCCTG
TCGAATGGCAACGGGATGGGAGACCAGGTGGTGGGTACGATGCTGCTGCTGATCTGCGTC
TGCGCCATCACGGACCCCCGCAACATGGAGGTTCCTAAGGCCCTGATTCCGCTCTTCGTG
GGCTTCACCGTCCTCAACATCGGCGTGTGCTTCGGCTTCAACTGCGGCTACGCTATCAAC
CCCGCCAGGGATCTCGCCCCCCGCTTCTTCACGCTCATTGCGGGATGGGGCGAGCAGACC
TTCACGGCTGGCACCTTCGACGGCATCGCCTGGTGGTGGGTGCCCCTCGTCGGCCCCCAC
ATCGGCGCTGTGATGGGCGTGGGCATATACCTTCTCCTG---ATCGAGCTGCACCACCCA
GAGGCCCAGGAGTTCTCCCTCCCGCACGTCCAC------
>GGVZ01008103_Acetes_chinensis_Glp
---------------ATGCTGCAGCCAAAACTCCGT---GTGAGGAACACCATCATCAGG
GAGTTCATGGCGGAGTTCCTTGGCACCTTTATCCTAGTGATCTTCGGCGACGCGTCAGTG
GCCCAGATGGTCCTGACCAACAAG---------------GGCTCCGGCGACTTCTTCTCC
GTCAACTGGGGCTGGGGCGTCGGCGTCACTCTGGCGGTACTCGTCAGCGGCGGCGTCAGC
GGCGGACACGTCAACCCCGCAGTCACACTGGCCATGGCCGTGTGGGGCAAGCAC---CCG
TGGGTCAAGGTGCCGGTATACATGGTGGCTCAGTACCTGGGTGGTCTAATGGGGGCCGCC
ATGGTGTATGCTGTATACCTCAACGCCCTGGACGCCTTTGAG---CCCACG---------
CGT------------------------------ACCCTGAGCACCGCGGGCATCTTCGCC
ACGTACCCAGGCTCGATGGTCAACGTCAACGGCACCACC------ATTGACAACTTCCTA
TACAACGGCAACGGCTTCGGTGACCAGATTGTGGGCACGATGTTGCTGTTGATCTGTGTG
TGCGCCATCACCGACCCCCGCAACATGGAAGTCCCCAAGGGTCTGGTCCCCTTCCTGGTG
GGTCTGACGGTCCTCAACATCGGGATCTGTTTCGGGTTCAACTGTGGGTACGCTCTGAAC
CCCGCCCGGGACTTGGCGCCCAGGTTCTTCACGCTCATCGCTGGCTGGGGCGATCAAACC
TTCTCGGCGGGCACGGTGGACGGTGTGTCGTGGTGGTGGGTGCCCCTGGTGGCGCCGCAC
ATCGGGGCGGTGCTGGGCGTGTCGGTGTACTGCCTGCTG---GTGGAGCTGCACCACCCA

GAGGCCCAGGACTTCTCCCTCCCGCAGATGCAG------
>GGFD01007766_Charybdis_feriata_Glp
GCCAAGTTAGTCGAGGCGGTTCAAACCAAACTGAAG---CTTCGGAACACAATGGCCAGG
GAATTTTTGGCTGAATTTCTTGGAACCTTCGTCCTAATAGCGTTCGGGACAGCCAGTGTG
GCCCAGAGTGTGCTAACCAACAAA---------------GCCAACGGGGACTTCTTCTCC
ATCAACTGGGGCTGGGGCATGGGTGTGACACTCGGGGTTTTGGTGAGCGGCGGGGTAAGC
GGAGGACACATCAACCCTGCAGTCACCGTGGCCATGGCGGTCTGGGGCAAACAC---CCG
TGGCAGAAGGTCCCAGTGTACATTGTGGCGCAGTACCTCGGGGCCTTCATTGCCTCGGCA
GTGGTCTACGGCGTGTATCTGAACGCCCTTGACGGCTTCGAA---GCGGAG---------
CGC------------------------------ACCCTGGCCACTGCGGGCATCTGGGCC
ACCTACCCGGGAGCTATCACAACAAGGAACGGCACTGTC------ATTACCGACTACCTG
TCCAGCGGGAACGGAATGAGCGACCAGGTGGTGGGCACGATGCTGCTGCTGCTGTGCGTG
TGTGCCATCACTGACGCACGCAACATGGAGGTGCCCAAGGGTTTGGTTCCCTTGCTAGTG
GGTCTCACCGTCCTCAACATTGGTGTGTGCTTCGGCTTCAACTGCGGCTATGCTATCAAC
CCAGCACGTGACTTGGGGCCCAGGATCTTCACCCTCATTGCTGGCTGGGGGAGCGCCACC
TTCAGCGCTGCCACCGTGGAGGGCATCGCATGGTGGTGGGTGCCAGTGGTGGGGCCTCAC
ATCGGCGCTATCCTAGGGGTCACCCTGTACGAGGTGTTC---GTGGCCCTTCACCACCCA
GACACGCAGGAGTTCACTCTACCGCAGGTCACC------
>GFPO01010392_Charybdis_japonica_Glp
GCCAAGTTAGTCGAGGCGGTTCAAACCAAACTGAAG---CTTCGGAACACAATGGCCAGG
GAATTTTTGGCTGAATTTCTTGGAACCTTCGTCCTAATAGCTTTCGGGACAGCCAGTGTG
GCCCAGAGTGTGCTAACCAACAAA---------------GCCAACGGGGACTTCTTCTCC
ATCAACTGGGGCTGGGGCATGGGTGTGACACTCGGGGTTCTGGTGAGCGGCGGGGTAAGT
GGAGGACACATCAACCCTGCAGTCACCGTGGCCATGGCGGTCTGGGGCAAACAT---CCG
TGGCAGAAGGTCCCAGTGTACATTGTGGCGCAGTACCTCGGGGCCTTCATCGCCTCGACA
GTGGTCTACGGCGTGTATCTGAACGCCCTTGACGGCTTCGAG---GCGGAG---------
CGC------------------------------ACCCTGGCCACTGCGGGCATCTGGGCC
ACCTACCCGGGAGCCATCACAACAAGGAACGGCACTGTC------ATTACCGACTACCTG
TCCAGCGGGAACGGAATGAGCGACCAGGTGGTGGGCACGATGCTGCTGCTGCTGTGCGTG
TGTGCCATCACTGACGCACGCAACATGGAGGTGCCCAAGGGTTTGGTCCCGTTGCTAGTG
GGTCTCACCGTCCTCAACATTGGTGTGTGCTTCGGCTTCAACTGCGGCTATGCTATCAAC
CCAGCACGTGACTTGGGGCCCAGGATTTTCACCCTCATTGCTGGCTGGGGGAGCGCCACC
TTCAGCGCTGCCACCGTGGAGGGCATCGCATGGTGGTGGGTGCCAGTGGTGGGACCTCAC
ATCGGCGCTATCGTGGGGGTCACTCTGTACGAGGTGTTC---GTGGCCCTTCACCACCCA
GACACGCAGGAGTTCACTCTACCGCAGGTCACC------
>GDRN01042436_Scylla_olivacea_Glp
GCCAAGCTGGTCGAGGTGGTTCAAAGCAAACTGAAG---CTTCGGAACACAATGGCCAGG
GAATTTTTGGCTGAATTTCTTGGAACCTTCATCCTAATAGCGTTCGGGACAGCCAGCGTG
GCCCAGAGTGTGCTAACCAACAAA---------------GCCAACGGGGACTTCTTCTCC
ATCAACTGGGGCTGGGGCATGGGTGTCACACTCGGGGTCCTGGTGAGTGGCGGGGTGAGC
GGGGGACATATCAACCCTGCAGTCACCGTGGCCATGGCGGTCTGGGGCAAACAC---CCG
TGGCAGAAGGTCCCACTGTACATTGTGGGGCAGTACCTCGGGGCCTTTATTGCCTCGGCA
GTGGTCTACGGCGTGTATCTGAACGCCCTTGACGGCTTCGAG---GCGGAG---------
CGC------------------------------ACTCTGGCCACGGCGGGCATCTGGGCC
ACCTACCCGGGCGCCATCACGACAAAGAACGGCACTGTC------ATTACCGACTACCTG
CCCAGCGGG---
------------------------------------AAGGGCCTGGTTCCCCTGCTAGTG
GGTCTTACTGTCCTCAACATCGGCGTGTGCTTCGGCTTCAACTGCGGCTATGCTATCAAC
CCAGCACGTGACTTGGCGCCCAGGATCTTCACCCTCATTGCTGGGTGGGGCAGCGACACC
TTCAGCGCTGCCACCGTGGAGGGCATCGCGTGGTGGTGGGTGCCAGTGGTGGGGCCTCAC
ATCGGCGCTATCCTGGGGGTCACCCTGTACGAGGTGTTC---GTGGCCCTTCACCACCCA
GAGACGCAGGAGTTCACCCTACCGCAGGTCACC------
>GFZC01025134_Portunus_sanguinolentus_Glp

---------------------------------------CTGGTGAGCGGCGGGGTGAGC
GGGGGACACATCAACCCTGCTGTCACCGTGGCCATGGCGATCTGGGGGAAGCAC---CCG
TGGCAGAAGGTTCCAGTGTATATTGTTGGGCAGTACCTCGGAGCCTTCATCGCTTCAGCA
CTGGTCTACGGCGTGTATCTAAACGCCCTTGACGGTTTTGAG---TCTGAG---------
CGC------------------------------ACCCTGGCCACGGCGGGCATCTGGGCC
ACCTACCCGGGCGCCATCACAGCAAGGAACGGCACTGTT------ATTACCGACTACCTC
TCCAGCGGGAACGGAATGGGTGACCAGGTGGTGGGCACGATGTTGCTGTTGCTGTGCGTG
TGTGCCATCACTGACACACGCAACATGGAGGTGCCCAAGGGTCTGGTTCCTCTGCTTGTG
GGCCTGACTGTCCTCAACATCGGCGTGTGCTTCGGCTTCAACTGCGGCTACGCTATCAAC
CCAGCACGTGACTTGGCGCCCAGGATCTTCACCCTCATTGCTGGGTGGGGCAGCGACACC
TTCAGCGCTGCCACCGTGGAGGGCCTGGCGTGGTGGTGGGTGCCGGTGGTGGGGCCTCAC
ATCGGCGCTATCCTGGGCGTCACTTTGTATGAGGTGTTC---GTGGCCCTTCACCACCCG

GAGACGCAGGACTTCACGCTTCCGCAAGTCACC------
>GFFJ01052468_Portunus_trituberculatus_Glp
GCCAAGCTGGTCGAGGCTGTTCAAAGCAAACTGAAG---CTTCGGAACACAATGGCCAGA
GAATTTTTGGCCGAATTTCTTGGAACCTTCGTCTTAATAGCGTTCGGGACAGCCAGTGTG
GCCCAAAGCGTGCTGACCAATAAA---------------GCCAATGGGGACTTCTTCTCA
ATCAACTGGGGATGGGGCATGGGCGTCACACTGGGGGTCCTGGTGAGCGGCGGGGTGAGC
GGGGGACACATCAACCCTGCAGTTACCGTAGCAATGGCGATCTGGGGGAAGCAC---CCG
TGGCAGAAGGTTCCAGTGTATATTGTTGCGCAGTATCTCGGAGCTTTCATCGCCTCAGCA
TTGGTCTACGGCGTGTATCTGAACGCCCTTGACGGATTTGAG---GCGGAG---------
CGC------------------------------ACCCTGGCCACGGCGGGCATCTGGGCC
ACCTACCCCGGCGCCATCACGGCAAGGAACGGCACTGTT------ATTACCGACTACCTC
TCCAGCGGGAACGGAATGGGCGACCAGGTGGTGGGCACGATGCTGCTGCTGCTGTGCGTG
TGTGCCATCACTGACGCACGCAACATGGAGGTGCCCAAGGGTCTGGTTCCTCTGCTTGTG
GGTCTGACTGTCCTCAACATCGGCGTGTGCTTCGGCTTCAACTGCGGCTACGCTATCAAC
CCAGCACGTGACTTGGCGCCCAGGATCTTCACCCTCATTGCTGGGTGGGGCAGCGACACC
TTCAGCGCTGCCACCGTGGAGGGCCTGGCGTGGTGGTGGGTGCCGGTGGTGGGGCCTCAC
ATCGGCGCTATCCTGGGCGTCACTTTGTATGAGGTGTTC---GTGGCCCTTCACCACCCG
GAGACGCAGGACTTCACCCTTCCGCAAGTCACC------
>GEID01008664_Callinectes_sapidus_Glp
GCCAAGCTAGTCGAAGCAGTTCAAAGCAAACTGAAG---CTTCGAAACACAGCGGCCAGA
GAGTTTTTGGCTGAGTTTCTTGGAACCTTCGTCCTAATAGCATTTGGAACCGCCAGTGTG
GCCCAGAGTGTGCTGACCAACAAA---------------GCCAATGGTGACTTCTTCTCC
ATCAACTGGGGC--
--TGGGGGAAACAC---CCG
TGGCAAAAGGTCCCAGTGTATATGGTGGGGCAGTACCTCGGAGCTTTCATCGCCTCGGCA
GTGGTCTACGGCGTGTATCTGGACGCCCTTGATGGCTTCGAA---GCTGAG---------
CGC------------------------------ACCCTGGCCACAGCGGGTATCTGGGCC
ACCTATCCCGGCGCCATCACC---------------------------------------

------TCAGTCCTCAACATCGGCGTGTGCTTCGGCTTCAACTGCGGCTATGCTGTCAAC
CCGGCCCGTGACTTGGCGCCCAGGATCTTCACCCTCATTGCTGGATGGGGCAGCGACACC
TTCAGTGCCGCTATCGTGGAGGGCCTGGCGTGGTGGTGGGTGCCGGTGGTGGGGCCTCAC
ATCGGCGCTATCCTGGGGGTCACCCTGTACGAGGTGTTC---GTGGCCCTTCACCACCCT
GAGACACAGGAGTTCACCCTACCGCAGGTCACC------
>GBXE01131730_Carcinus_maenas_Glp
GCAAAACTAGTGGAAGCGGTTCAAACTAAACTGAAG---CTTCGAAACACAATGGCCAGG
GAATTTTTGGCTGAATTTCTTGGAACCTTCGTCTTAATAGCCTTCGGAACCGCCAGCGTC
GCCCAGAGTGTCCTGACCAACAAG---------------GCTAATGGGGACTTCTTCGCC
ATTAACTGGGGCTGGGGTATGGGGGTGACGCTGGGGGTGCTGGTGTGCGGGGGGGTGAGC
GGGGGACACATCAACCCTGCCGTCACCGTGGCTATGGCGGTCTGGGGGAAGCAC---CCG
TGGCAAAAGGTCCCCGTGTATATGATTGCCCAGTACCTCGGGGCTTTTGTGGCCTCCGGG
GTGGTCTACGGTGTTTACTTGAACGCCCTGGACGGCTTCGAG---GGAGAG---------
AGG------------------------------ACGCTGGCCACGGCGGGCATCTGGGCC
ACCTACCCAGGCACTATCACAGCGAGGAACGGCACTGTC------ATTAGCGACTACCTC
TCCAGCGGCAACGGGATGGCTGACCAGGTGGTGGGGACAATGTTGCTGCTGCTGTGCGTG
TGTGCCATCACCGACACACGCAACATGGAGGTGCCCAAGGGTCTGGTGCCGCTCCTCGTG
GGTCTGACCGTGCTCAACATCGGGGTGTGTTTCGGCTTCAACTGCGGCTATGCTATCAAC
CCTGCTCGTGACTTGGCGCCCAGGATCTTTACACTCGTCGCTGGATGGGGTAGTGACACG
TTCAGCGCTGCCACCGTGGAGGGGATAGCGTGGTGGTGGGTGCCAGTGGTGGGCCCTCAC
ATCGGTGCCATCCTGGGGGTCACTCTGTACGAGGTATTT---GTGGCACTCCACCATCCT
GAGGCACAAGAATTCACACTACCCCAAGTCAGC------
>GEFB01000383_Cancer_borealis_Glp
GCAAAACTGGTCGAAGCAGTTCAGGGCAAAATGAAG---CTTCGGAACACCATGGCGAGG
GAGTTTTTGGCTGAATTTCTTGGAACTTTCGTCCTGATCGCCTTCGGAACAGCCAGTGTC
GCGCAGGTCGTCCTAACCAACAAG---------------GGAAATGGTGACTTCTTCTCC
ATCAACTGGGGCTGGGGGATGGCTGTCATGCTGGGGGTGCTAGTGAGCGGAGGGGTGAGC
GGGGGACACATCAACCCAGCTGTCACCGTGGCTATGGCTGTCTGGGGGAAGCAC---CCG
TGGAGTAAGGTCCCCGTGTATATGGTTGCACAGTACCTAGGAGCTTTCATTGGTTCAGCT
GTGGTCTACGGCGTTTACCTAAACGCCCTGGACGGCTACGAG---GCAGAG---------
CGC------------------------------ACCCTGGTCACGGCGGGCATCTGGGCC
ACCTACCCAGGCACCGTCACAACACGCAACGGCACACTC------ATTAACGACTACCTG
TCCAACGGCAACGGCCTAGGCGACCAGGTGGTGGGAACAATGCTGTTGCTGCTGTGCGTG
TGTGCCATCACTGACACACGCAACATGGAGGTGCCCAAGGGCCTCGTGCCGCTGCTGGTC
GGCCTCACTGTACTCAACATTGGCGTGTGTTTCGGCTTCAACTGCGGCTACGCCATCAAC
CCTGCACGTGACCTGGCGCCGAGACTCTTCACCTTGATTGCAGGATGGGGCGGCGACACC
TTTACTGCTGCCACCAGTGAGGGCGTGGTGTGGTGGTGGGTGCCAGTGGTCGGGCCTCAC
ATCGGTGCCATTATTGGAGTCACTCTCTATGAGGTGTTC---ATCGCCCTCCACCACCCC

```
GATGCGCAGGAGTTTACTCTACCGCAGGTCACT------
>HACH01060232_Hyas_araneus_Glp
------------------------------------------------------------
------------------------------------------------------------
------------------------------------------------------------
------------------------------------------------------------
------------------------------------------------------------
------------------------------------------------------------
------------------------------------------------------------
------------------------------------------------------------
------------------------------------------------------------
------------------------------------------------------------
---------ACGGACACGCGCAACATGGAGGTGCCCAAGGGCCTGGTGCCGCTCCTTGTG
GGCCTGTCCGTGCTCAACATCGGCGTGTGCTTCGGCTTCAACTGCGGCTACGCCATCAAC
CCGGCGCGCGACCTGGCCCCGAGGATCTTTACGCTGATAGCCGGGTGGGGCAGCGATACA
TTCAGCGCGGCTACTATTGAGGGCGTGGCGTGGTGGTGGGTGCCTGTGGTGGGGCCTCAC
ATCGGTGCCGTGTTGGGGGTGACCATCTACGAGGTGTTT---GTGGGACTCCATCACCCC
GAAGCCAAGGAGTTCACGCTGCCACAAGTCGCC------
>GIJU01039950_Pachygrapsus_marmoratus_Glp
GCAAAACTAGTTGAAGCAGTTCAGAGCAAGATGAAA---CTCCGAAACCCCCTCGTGAGA
GAGTTTCTGGCAGAGTTTCTCGGGACTTTCATCCTCATCACATTTGGCACAGCATCAGTA
GCCCAGAGCGTCCTCACCATGAAA---------------GCCAATGGGGACTTTTTCTCC
ATCAATTGGGGCTGGGGGATGGGAGTGACTCTTGGGGTACTTGTGAGCGGCGGCATAAGT
GGGGGACACATCAACCCAGCTGTCACTGTGGCCATGGCGGTCTGGGGCAAGCAT---CCA
TGGAACAAGGTCCCTATTTATATGGTTGGCCAGTACCTCGGTGCTTTCCTCGCCTCTGCT
ATGGTGTACGGAGTCTACTTGAACGCCCTGGACAACTTCGAA---GGTGAG---------
AGG------------------------------AGCCTGGCCACGGCGGGTATCTGGGCC
ACCTACCCCGGCGCCATCATCAACATCAACGGGACTGTC------GTTACTGACTACCTG
TCCAGCGGCAACGGCTTGGGAGACCAGGTCGTGGGAACGATGCTGCTGCTGATCTGTGTG
TGCGCCATCACGGACACACGCAACATGGAGGTCCCCAAGGCCCTCATCCCTCTCTTTGTT
GGACTCACTGTGCTCAACATCGGCGTGTGTTTCGGGTTCAACTGCGGCTACGCCATCAAC
CCAGCCCGTGACCTTGCCCCGAGAATCTTTACTTTGATTGGTGGATGGGGAAGTGAAACG
TTCAATGCCGCCACCAACGAGGGCGTGGCGTGGTGGTGGGTGCCGGTGGTGGGGCCTCAC
ATCGGTGCCGTGCTGGGAGTCACCATCTACGAGGTGCTG---GTGGGCCTGCACCACCCA
GAGGCACAGGAGTTCACGCTGCCCCAGGTCACG------
>GFXJ01004053_Gecarcoidea_natalis_Glp
GCGAAACTAGTTGAAGCAGTACAGAGCAAAATGAAA---CTTCGAAACACCCTTGTGAGA
GAATGCCTGGCGGAGTTTCTCGGGACCTTCATCCTCGTGGCGTTCGGGACAGCATCCGTA
GCTCAGAGCGTTCTCACAATGAAA---------------GCCAACGGAGACTTCTTCTCT
ATCAACTGGGGCTGGGGGATGGGCGTGACTATGGGGGTACTGGCGAGCGGGGGCATAAGT
GGAGGGCACATCAACCCAGCTGTCACCGTGGCCATGGCTATCTGGGGCAAGCAT---CCA
TGGAATAAAGTCCCAGTTTATATGGTTGGCCAATATCTGGGAGCCTTCATTGCCTCTGCT
GTGGTGTATGGAGTCTACCTGAACGCATTGGACAGCTTCGAG---GGGGAG---------
AGG------------------------------AGCCTTGCCACCGCGGGAATCTGGGCC
ACTTATCCCGGCGCCATCACCAACATCAACGGGACTCTC------GTTACTGACTACCTG
TCCAGCGGCAACGGCTTTGGAGATCAGGTGGTGGGCACGATGCTACTGCTCATCTGTGTG
TGCGCCATCACGGACACACGCAATATGGAGGTTCCCAAGGCCCTCATCCCTCTCTTCGTG
GGGCTCACAGTGCTCAACATCGGCGTGTGTTTCGGGTTCAACTGCGGCTATGCGATCAAC
CCAGCCCGTGACCTTGCCCCGAGGATCTTTACGCTGATTGGTGGATGGGGAAGTGACACG
TTCAGCGCCGCCACCAATGAGGGCGTGGCGTGGTGGTGGGTGCCAGTGGTGGGGCCTCAC
ATTGGTGCCGTCCTGGGGGTCACCATCTACGAGGTGTTT---GTGGGCCTTCACCACCCC
GAGGCACAGGAGTTCACGCTGCCGCAGGTCACG------
>GBZW01018927_Eriocheir_sinensis_Glp
GCGAAGCTTGTCGAAGCCGTCCAGAGCAAGATGAAA---CTTCGGAACTCGCTCCTACGA
GAGTTTTTGGCGGAGCTACTGGGAACCTTCATTCTCCTGGCCTTCGGGACAGCCTCAGTG
GCCCAGTGCATCCTCACTAACAAA---------------GCTAACGGGGACTTCTTCTCT
ATCAACTGGGGATGGGGCATTGGCGTGACGCTGGGGGTGCTGGTGAGCGGGGGCATCAGC
GGGGGGCACATCAACCCAGCTGTCTCCGTGTCCCTAGCGGTCTGGGGTAAATTT---CCC
TGGAATAAGGTCCCGATCTATATGATTGGCCAGTACATCGGGGCCTTCCTTGCCTCTGCT
ATGGTCTACGGGGTGTACCTGAACGCATTGGACAGCTTCGAA---GGGGAC---------
CGA------------------------------ACATTGGCCACTGCTGGGATCTGGGCA
ACCTATCCTGGCTCAATCACCAACGTTAACGGAACGGTG------ATGACTGACTACCTG
TCCAGCGGTAATGGTTTGGGAGATCAGGTGGTAGGAACGATGCTGCTGCTAATCTGCGTG
TGTGCCATCACGGACCCACGTAACATGGAGGTCCCCAAGGCTCTGATCCCGCTCTTCGTC
GGGTTCACGGTCATCAACATCGGTGTCTGCTTCGGCTTCAACTGCGGCTATGCGATCAAC
CCAGCCCGAGACCTTGCCCCGAGGATCTTCACTCTCGTTGGTGGATGGGGAGCGATACG
TTTAGTGCCACAACCAACGATGGCGTGGCATGGTGGTGGGTGCCAGTGGTCGGCCCTCAC
ATCGGCGCCATCCTGGGAGTCACCGTCTACGAGGTCTTT---ATCGGACTGCACCACCCT
```

GAGGCCCAAGAGTTCACGCTGCCGCAAGTCACG------
>GHGY01075277_Paralithodes_camtschaticus_Glp
CAGACACTGATAGAGGTTATTCAGGAGCGTGTGAGG---TTGAGATGGTCCATGGCTAGG
GAGTTCTTAGCAGAGTTCCTGGGTACCTTCATCCTCATACTGTTCGGTAATGCATCGGTG
GCCCAGAGCGTACTGACCAACAGT---------------GCCAATGGTGATTTCTTCTCC
ATCAACTGGGGGTGGGGCATGGCAGTGACCCTGGGTGTGTTGGTCAGTGGGGGGATCAGT
GGAGGTCACATCAACCCGGCCGTGACCCTGGCCATGGCCATCTGGGGCAAACAT---CCC
TGGGTCAAGGTGCCGATCTACATGGTGGCGCAGTACCTCGGAGCCTTCCTCGCCTCGGCT
GTACTCTACGGGGTCTACCTTAACGCCCTTGACGCCTTTGAC---CCCGAG---------
CGG------------------------------ACCATACTGACGGCGGGTATCTGGGCC
ACCTACCCTGGGGCCGTCCACAACGCCACCACGGGCACC------GGAGTTCCCTTCCTC
TCCAGCGGCAACGGAATGGGCGATCAGATTGTGGGAACGATGGTGTTGCTGATCGCCGTG
TGCGCCATCACTGACCGCAAGAACATGGAGGTTCCCAAGGCCATGATCCCCCTGCTGGTG
GGTCTAACAGTCCTCAACATCGGGATCTGCTTCGGGTTCAACTGTGGGTACGCCATCAAC
CCCGCCCGAGACCTGGCGCCGCGTTTCTTCACCATGTTGGCCGGCTGGGGAAGCATGCCG
TTCACGGCCACCACAGTTGACGGTGTGGCATGGTGGTGGGTGCCAGTGGTGGGACCCCAC
ATCGGAGCCATCCTGGGAGTCGCCATCTACGTCCTCTGT---ATCGAGCTACACCACCAC
AAGGAGGAAGAGTACCCCCTCCCCCAGATCCAA------
>CM023256_Paralithodes_platypus_Glp
CAGACACTGATAGAGGTTATTCAGGAGCGTGTGAGG---TTGAGATGGTCCATGGCTAGG
GAGTTCTTAGCAGAGTTCCTGGGTACCTTCATCCTCATACTGTTCGGTAATGCATCGGTG
GCCCAGAGCGTACTGACCAACAGT---------------GCCAATGGTGATTTCTTCTCC
ATCAACTGGGGGTGGGGCATGGCAGTGACCCTGGGTGTGTTGGTCAGTGGGGGGATCAGT
GGAGGTCACATCAACCCGGCCGTGACCCTGGCCATGGCCATCTGGGGCAAACAT---CCC
TGGGTCAAGGTGCCGATCTACATGGTGGCGCAGTACCTCGGAGCCTTCCTCGCCTCGGCT
GTACTCTACGGGGTCTACCTTGACGCCCTTGACGCCTTTGAC---CCCGAG---------
CGG------------------------------ACCATACTGACGGCGGGTATCTGGGCC
ACCTACCCTGGGGCCGTCCACAACGCCACCACGGGCACC------GGAGTTCCCTTCCTC
TCCAGCGGCAACGGAATGGGCGATCAGATTGTGGGAACGATGGTGTTGCTGATCGCCGTG
TGCGCCATCACTGACCGCAAGAACATGGAGGTTCCCAAGGCCATGATCCCCCTGCTGGTG
GGTCTAACAGTCCTCAACATCGGGATCTGCTTCGGGTTCAACTGTGGGTACGCCATCAAC
CCCGCCCGAGACCTGGCGCCGCGTTTCTTCACCATGTTGGCCGGCTGGGGAAGCATGCCG
TTCACGGCCACCACAGTTGACGGTGTGGCGTGGTGGTGGGTGCCAGTGGTGGGACCCCAC
ATCGGAGCCATCCTGGGAGTCGCCATCTACGTCCTCTGT---ATCGAGCTACACCACCAC
AAGGAGGAAGAGTACCCCCTCCCCCAGATCCAA------
>GBEI01067429_Astacus_leptodactylus_Glp
AACAAACTCATGAAGGCGATGCAGTCCAAGCTGAGG---CTGCGGAACGTTATTATCAGA
GAGTTCTTAGCAGAGTTCTTGGGCACCTTCATCCTGATCGTGTTTGGGAACGCGTCGGTG
GCCCAAACCGTCCTCACCAGCAGC---------------GCAAACGGCGATTTCTTCTCA
ATCAACTGGGGGTGGGGCATTGGGGTGACCCTAGCGGTGGTGGTGAGCGGCGGGGTGAGC
GGGGGTCACGTCAACCCAGCTGTGACCCTGGCCATGGCAGTGTGGGGCAAGCAC---CCC
TGGTGTAAGGTGCCTGTGTATATGGTGGCTCAGTACCTCGGGGCCTTCCTCGCCTCGGCC
CTCGTCTACGCTGTCTATCTCAATGCACTGGACGCGTTCGAT---CCCGAA---------
CGC------------------------------AGCATGGCAACGGCCGGGATCTGGGCC
ACCTACCCAGGGGCCATCACCAACATCAACGGAACCATG------GTCAATGACTACCTC
TCCAACGGTAATGGCTTCGGCGACCAGGTGATGGGGAGCATGCTGCTGCTAATATGCGTG
TGTGCCATCACTGACCCTCGCAACATGGAGGTGCCCAAGTACATGGTGCCGCTCCTCGTC
GGCTTCGTCGTCCTCAACATTGGCATCTGCTTTGGCTTCAACTGCGGCTACGCCATCAAC
CCCGCCCGGGACCTGGCCCCCAGGCTCTTCACTCTCCTCGCTGGCTGGGGAGAGTCCCCC
TTCACGGCGATGACCAGCGAGGGCCTGGCATGGTGGTGGGTGCCGGTAGTGGCGCCTCAT
ATCGGAGCCATCCTGGGCGTCATGGTCTACCTTTTCTTC---ATCGAGCTACACCACCCG
GAGGCTCAGGAGTTCGTCCTGCCACAGGTCCAG------
>GICG01035749_Austropotamobius_pallipes_Glp1

---------------------------------ATGGCAGTGTGGGGCAAGCAC---CCC
TGGTGCAAGGTGCCTGTGTATATGGTGGCTCAGTACCTCGGGGCCTTCCTCGCCTCGGGA
CTCGTCTACGCTGTCTATCTCAATGCACTGGACGCGTTCGAG---CCCGAA---------
CGC------------------------------AGCATGGCAACGGCCGGGATATGGGCC
ACCTACCCAGGGACCATCACCAACATCAACGGAACCATG------GTCAATGACTACCTC
TCCAACGGTAATGGCTTCGGCGACCAGGTGATGGGGAGCATGCTGCTGCTAATATGCGTG
TGTGCCATCACTGACCCTCGCAACATGGAGGTGCCCAAGTACATGGTGCCGCTCCTCGTC
GGCTTCGTCGTCCTCAACATTGGCATCTGCTTTGGCTTCAACTGCGGCTACGCCATTAAC
CCCGCCCGGGACCTG---

>GBEV01014956_Procambarus_clarkii_Glp
AACAAACTGATGGAGGCGATGCAGTCCAAGCTGAGG---CTGAGGAACGTCATCATCAGA
GAGTTCCTAGCAGAGTTCCTGGGCACTTTCATCCTGATCGTGTTCGGGAACGCGTCTGTG
GCTCAGTATGTGCTGACCAACAAG---------------GAAGGCGGAGACTTCTTCTCC
ATCAACTGGGGGTGGGGCTTGGCGGTGACCCTGGCGGTACTGGTGAGCGGCGGGGTCAGC
GGAGGTCACGTCAACCCGGCCGTGACCCTGGCCATGGCAGTGTGGGGCAAGCAC---CCC
TGGTGTAAGGTGCCCGTCTACATGGTGGCCCAGTATCTGGGGGCCTTCCTCGCCTCCTCC
CTCGTCTACGCCGTCTACCTCAACGCTCTGGACGCCTACGAG---CCGGAG---------
CGG------------------------------ACCATGGCCACGGCGGGCATCTGGGCC
ACCTACCCGGGGGTCATCACCAACATCAACGGCACCGTT------GTCACTGACTACCTC
TCCAACGGTAATGGCTTCGGCGATCAGGTGATGGGGACGATGCTGCTGCTGATATGCGTG
TGTGCCATCACCGACCCCCGCAACATGGAGGTGCCCAAGTATATGGTGCCGCTGCTCGTC
GGCTTCACCGTCCTCAACATCGGCATCTGCTTCGGCTTCAACTGTGGCTACGCCATCAAC
CCCGCCCGAGACCTGGCGCCCAGACTCTTCACCCTCCTCGCTGGCTGGGGGGATGACCCC
TTCACGGCGGCCACCAGCGAGGGTCTGGCCTGGTGGTGGGTTCCTGTGGTCGCGCCTCAC
ATCGGAGCCATCCTGGGCGTCATGATCTACCTCTTCTTC---ATCGAACTTCATCACCCA
GAAGCCCAAGAGTTCGTCCTGCCGCAGATCCAG------
>MRZY010003347_Procambarus_virginalis_Glp
AACAAACTGATGGAGGCGATGCAGTCCAAGCTGAGG---CTGAGGAACGTCATCATCAGA
GAGTTCCTAGCAGAGTTCCTGGGCACCTTCATCCTGATCGTG------------------

---------GTCAACCCGGCCGTGACCCTGGCCATGGCAGTGTGGGGCAAGCAT---CCC
TGGTGTAAGGTGCCCGTCTACATGGTGGCCCAGTATCTGGGGGCATTCCTCGCCTCCTCC
CTCGTCTACGCCGTCTACCTGAGT------------------------------------

------CCGGGTGTCATCACCAACATCAACGGCACCGTT------GTCTCTGACTACCTC
TCCAACGGTAATGGCTTCGGCGACCAGGTGATGGGGACGATGCTGCTGCTGATATGCGTG
TGTGCCATCACCGACCCCCGCAACATGGAGGTGCCCAAGTATATGGTGCCGCTGCTCGTC
GGCTTCACCGTCCTCAACATCGGCATCTGCTTCGGCTTCAACTGTGGCTACGCCATCAAC
CCCGCCCGAGACCTGGCGCCCAGACTCTTCACCCTCCTCGCTGGTTGGGGGGATGACCCC
TTCACG--

>HACK01026591_Cherax_quadricarinatus_Glp
AACAAGTTTCTGGAGGCGTTGCAATCTAAACTCCGG---CTGAGAAACGTCATAATCCGA
GAGTTTCTCGCAGAGTTCCTGGGCACCTTCATCTTGATCGTTTTCGGGAACGCTGCGGTA
GCACAGGCTGTGCTCACCAACAAT---------------GTCAAGGGTAGCTTCTTCGCC
ATCAACTGGGGATACGGCTTGGCAGTGACCCTGGCTGTGCTGGTGAGCGGCGGGGTCAGC
GGAGGTCACGTTAACCCAGCGGTGACCCTAGCCATGGCCGTGTGGGGTAAACAC---CCC
TGGAGTAAAGTACCTGTTTACATGGTCGCCCAGTACTTAGGAGCCCTGCTAGCCTCCCTT
GCTGTCTTCGCTGTCTACATAAACGCCCTGGACGCATTTGAG---CCGCAT---------
CGC------------------------------AGCTTGGCTACCGCTGGGATCTGGGCC
ACCTACCCCGGTTCCATGACCAACATCAACGGCACTCTT------GTCACCGACTTTCTG
TCCAACGGCAACGGCTTCGGTGATCAGGTTCTCGGCACAATGTTGCTGCTGATCTGCGTG
TGTGCCATCACCGACCCCAGGAACATGGAGGTGCCAAAGTATATGGTGCCTCTCCTAGTC
GGCTTCGTTGTCCTTAACATCGGTATCTGCTTTGGCTTCAACAGCGGCTACGCTATCAAC
CCCGCCAGGGACCTGGCACCAAGACTCTTCACTATCCTTGCTGGCTGGGGAGAGACTCCC
TTCACGGCAGCAACTAGCGAGGGTTTGGCGTGGTGGTGGGTGCCTGTGGTAGCCCCACAC
ATCGGTGCTGTCCTGGGAGTGATGGTCTATCTCTTCCTC---GTCGAGCTTCATCATCCA
GAGTCCCAGGAATTCGCTCTGCCACAGGTGTTG------
>WNWK01008455_Cherax_destructor_Glp
AACAAGTTTCTGGAGACGTTACAATCTAAACTCCGG---CTGAGAAACGTCATAATCCGA
GAGTTTCTCGCAGAGTTCCTGGGCACCTTCATCTTGATCGTTTTCGGGAACGCTGCGGTA
GCACAGGCTGTGCTCACCAACAAT---------------GTCAAGGGTAGCTTCTTCTCC
ATCAACTGGGGATACGGCTTGGCAGTGACCCTGGCTGTGCTGGTGAGCGGCGGGGTCAGC
GGAGGTCACGTTAACCCAGCGGTGACCCTAGCCATGGCCGTGTGGGGCAAACAC---CCC
TGGAGTAAAGTACCTGTTTACATGGTCGCCCAGTACTTAGGAGCCCTGTTAGCCTCCCTT
GTTGTCTTCGCTGTCTACATAGATGCTCTGGACGCCTTTGAG---CCGCAT---------
CGC------------------------------AGCTTGGCTACCGCTGGGATCTGGGCC
ACCTACCCAGGCTCCATGACCAACATCAACGGCACTCTT------GTCACCGACTTTCTG
TCCTACGGCAACGGCTTCGGTGATCAGGTTCTCGGCACGATGTTGCTGCTGATCTGCGTG
TGTGCCATCACCGACCCCAAGAACATGGAGGTGCCAAAGTACATGGTGCCTCTCCTAGTC
GGCTTCGTTGTCCTCAACATCGGCATCTGCTTTGGCTTCAACAGCGGCTACGCAATCAAC
CCCGCCAGGGACTTGGCACCCAGACTCTTCACTATCCTTGCTGGCTGGGGAGAGACGCCC
TTCACGGCAGCAACTAGCGAGGGTCTGGCGTGGTGGTGGGTGCCTGTGGTAGCCCCACAC
ATCGGTGCTGTCCTGGGAGTGATGGTCTATCTCTTCCTC---ATCGAGCTTCATCATCCA

GAGTCCCAGGAATTTGCTCTGCCGCAGGTGTTG------
>GFUC01047188_Homarus_americanus_Glp
AACAAGTTTCTGGAGGCGATGCAGTCCAAGCTGAGG---TTGAGGAACGTTATGTTCAGG
GAGTTCCTGGCAGAGTTCCTGGGTACCTTCATCCTAATTCTGTTCGGGGATGCGTCGGTG
GCTCAGAGTGTGTTGACCAACAAG---------------GCAAATGGAGACTTCTTCTCC
ATCAACTGGGGTTGGGGCCTGGCGGTGACCCTGGCCGTCCTGGTGAGTGGTGGAGTCAGT
GGAGGTCACGTCAACCCGGCGGTCACCCTGGCCATGGCTGTATGGGGGAAGCAC---CCG
TGGGGGAAGGTGCCAATCTACATGGTGGCGCAGTATATGGGGGCCCTCCTAGCCTCCGCC
CTCCTCTACGGCGTCTACCTCAATGCACTTGACGGGTTCGAC---CCAGAG---------
CGG------------------------------ACCATGGCCACGGCTGGCATATGGGCT
ACCTACCCAGGGGCCATC---AACGTCAACGGTACTGTC------GTCAATGACTTCCTT
TCCAACGGCAATGGCTTCGGCGACCAGGTGGTTGGCACTATGTTGCTGCTGGTGTGTGTG
TGTGCCATCACTGACCCCCGCAACATGGAGGTGCCCAAGTACCTGGTGCCACTGTTCGTC
GGTTTCACCGTCCTCAACATCGGCATCTGCTTCGGCTTCAACTGCGGCTACGCTATTAAT
CCCGCCCGAGACCTGGCACCTAGACTCTTTACTTTACTGGCTGGCTGGGGGGAGACACCC
TTCACAGCGGTGACCATCGAGGGGCTCGCCTGGTGGTGGGTGCCGGTAGTGGCGCCTCAC
ATCGGTGCGATTCTGGGCGTGATGGTCTACATCTTCTTC---ATCGAGCTCCACCACCCA
GAGGCCCAAGAGTTCGTCCTTCCGCAAGTTCAA------
>GHUJ01025978_Panulirus_argus_Glp
AACAAGCTGCTGGAGATGATGCAGTCCAAGCTTAGG---CTGAAGAACCAAATGGGCCGG
GAGTTCCTGGCGGAACTGCTGGGCACCTTCATCCTAATCCTGTTTGGCGACGCATCTGTG
GCTCAGAGCGTTCTTACCAACAAG---------------GCCAACGGCGACTTCTTCTCC
ATCAACTGGGGCTGGGGTCTGGCCGTGACCCTGGCCGTGCTGGTGAGCGGCGGGGTCAGC
GGAGGTCACGTGAACCCGGCGGTGACCCTGGCCATGGCCATCTGGGGCAAGCAC---CCG
TGGGCCAAGGTGCCCGTGTACATGGTGGCCCAGTACCTGGGGGCCTTCCTGGCCTCGGCC
CTCCTCTACAGCGTCTACCTCAACGCCTTGGATGGGTTCGAA---CCCGAG---------
AGG------------------------------ACCCTGGCCACGGCAGGCATCTGGGCC
ACCTACCCGGGCGCCATCACCAACGTCAACGGCACCGTC------GTCTCCGACTTCCTC
TCCAGCGGCAACGGCTTCGGCGACCAGGTGGTTGGCACGATGTTGCTGCTCATCTGCGTG
TGCGCCATCACCGACGGGCGGAACATGGAGGTGCCCAAGTACCTGGTGCCGCTCCTGGTG
GGCTTCACCGTCCTCAACATTGGCATCTGCTTCGGCTTCAACTGCGGCTACGCCATCAAC
CCGGCCCGAGACCTGGCTCCTAGGCTGTTTACCCTCATCGCTGGCTGGGGAGAGGCGCCC
TTCACGGCGACGACCAAGGACGGCCTGGCGTGGTGGTGGGTGCCTCTGGTGGGGCCACAC
ATCGGCGCCGTTCTGGGGGTCATGATCTACCTCTTCCTC---ATCGAGCTGCACCACCCG
GAGGCCCAGGAGTTCGCCCTGCCGCAGGTCCAC------
>GGHM01114588_Jasus_edwardsii_Glp
AACAAGCTGGTGGAAACAATGCAGTCCAAGCTGAGG---CTGAAGAACGTCATGGCCCGG
GAGTTCCTGGCCGAGCTGCTGGGCACCTTCATCTTGGTGCTCTTTGGTGACGCGTCGGTA
GCCCAGAGCGTGCTCACCAACAAG---------------GCCAATGGTGACTTCTTCTCC
ATCAACTGGGGCTGGGGCCTGGCCGTGACCCTGGCCGTCCTGGTGAGCGGTGGGGTCAGC
GGTGGCCACGTGAACCCGGCGGTGACCCTGGCCATGGCCGTGTGGGGGAAGCAC---CCG
TGGGCCAAGGTGCCGGTCTACATGGTGGCCCAGTACCTCGGGGCCTTCCTGGCCTCGGCC
CTACTCTACGGTGTATACCTCAATGCTTTGGACGAGTTCGAG---GCAGAG---------
CGG------------------------------ACCATGGCCACGGCTGGCATCTGGGCC
ACCTACCCCGGCGTCATCACCAACGTCAACGGCACCGCT------GTTACTGACTTCCTG
TCCAGTGGCAACGGTTTTAGTGACCAGGTGGTGGGGACGATGCTCCTGCTCATCTGCGTC
TGCGCCATCACGGACCCCCGCAACATGGAGGTGCCCAAGTACCTGGTGCCCCTCCTGGTG
GGCTTCACCGTCCTCAACATCGGCGTCTGCTTCGGCTTCAACTGCGGCTACGCCATCAAC
CCGGCCCGAGACCTGGCCCCTCGGCTTTTCACACTGATCGCTGGCTGGGGCGAGGCGCCT
TTCACGGCAATGACCAAGGACGGGCTAGCGTGGTGGTGGGTCCCGCTGGTGGGGCCCCAC
ATCGGGGCCATCATAGGCGTCATGGTCTACCTCTTCCTC---ATCGAACTGCACCACCCG
GAGGCCCAGGAGTTCGCCCTACCGCAAGTACAC------
>GFCS01024937_Euphausia_superba_Glp1
AACTATATCACTGGAACCTTCCCCAAG---AGCCGC---ATCGGCATCCCAGTCATCAAG
GAGTTCCTGGCAGAGTTCCTTGGTACATTCGTGCTTGTGATGTTTGGTGATGGTTCCGTA
GCACAAAGCGTCCTCACAGGTCAA---------------GCCAATGGAGATGTCTTCTCC
ATCAACTGGGGCTGGGCTGTGGCTGTCATGTTGGCTGTGTTGGTCTCTGGAGGAGTGTCG
GGAGGTCACGTAAATCCAGCAGTAAGTTTGGCGATGGCTGTTTGGGGCAAATTC---CCT
TGGGCCAAGGTACCAGTTTATATGGTGGCACAGTACCTCGGGTCCTTTTGTGCCAGTGCG
TTGCTGTATGGAGTATATCTCAATGCTCTTGATAACTATGAC---CCCGGC---------
CGC------------------------------ACTGATCTGACTGCCGGCATCTGGGCC
ACCTACCCCGGTGTGGCAACACTACTCAATGGCACCCTA---GAAGCTACTGCCTTCCTA
TCAGCTGGAAATGGACTCAGTGATCAGATTGTTGGCACCATGCTTCTACTGATTTGTGTG
TGTGCTATCACAGATCCAAGAAATATGCAAGTCCCCAAGGCTCTCGTTCCACTATTTATT
GGATTCACGGTACTGAACATTGGTATCTGCTTTGGGTTTAACTGCGGCTACGCCATCAAC
CCAGCTCGAGATCTCGCTCCAAGAATGTTCACTCTCATCGCTGGTTGGGGTGACCATCCA
TTTACAGCTATGACCATTGACAGTGTGGCATGGTGGTGGATTCCAATCATTGGCCCACAC
ATAGGCGCTATTCTTGGTGTTTTTATTTACTTGATCTTC---ATAGAACTTCACCATACG

GAGGCACAAGAGTTCAACCTTCCACAAGTTCAA------
>GFCS01481536_Euphausia_superba_Glp2
-----------------------AAGTTGGTACAG---ATCAAGAACCCCCTCTACAAA
GAGTTCCTCGCAGAATTCCTAGGGATGTTTATACTTATGATGTTTGGTGATGGTTCCGTA
GCACAAAGCGTCCTCACAGGTCAA---------------GCCAATGGAGATGTCTTCTCC
ATCAACTGGGGCTGGGCTGTGGCTGTCATGTTGGCTGTGTTGGTCTCTGGAGGAGTGTCG
GGAGGTCACGTAAATCCAGCAGTAAGTTTGGCGATGGCTGTTTGGGGCAAATTC---CCT
TGGGCCAAGGTACCAGTTTATATGGTGGCACAGTACCTCGGGTCCTTTTGTGCCAGTGCG
TTGCTGTATGGAGTATATCTCAATGCTCTTGATAACTATGAC---CCCGGC---------
CGC------------------------------ACTGATCTGACTGCCGGCATCTGGGCC
ACCTACCCCGGTGTGGCAACACTACTCAATGGCACCCTA---GAAGCTACTGCCTTCCTA
TCAGCTGGAAATGGACTCAGTGATCAGATTGTTGGCACCATGCTTCTACTGATTTGTGTG
TGTGCTATCACAGATCCAAGAAATATGCAAGTCCCCAAGGCTCTCGTTCCACTATTTATT
GGATTCACGGTACTGAACATTGGTATCTGCTTTGGGTTTAACTGCGGCTACGCCATCAAC
CCAGCTCGAGATCTCGCTCCAAGAATGTTCACTCTCATCGCTGGTTGGGGTGACCATCCA
TTTACAGCTATGACCATTGACAGTGTGGCATGGTGGTGGATTCCAATCATTGGCCCACAC
ATAGGCGCTATTCTTGGTGTTTTTATTTACTTGATCTTC---ATAGAACTTCACCATACG
GAGGCACAAGAGTTCAACCTTCCACAAGTTCAA------
>GETT01055166_Meganyctiphanes_norvegica_Glp1
AACTACATTACAAGGAATTTCCCGAAG---AGCCGC---CTGAAAAATTGGTACGTCAAG
GAGTTCCTCGCCGAGATGCTCGGCACATTCGTGCTTGTGATGTTTGGTGATGGTTCAGTG
GCACAAAGCGTTCTCACAGGTCAA---------------GCTAATGGAGACGTCTTCTCA
ATCAACTGGGGCTGGGCTGTGGCTGTCATGCTGGCTGTGTTGGTATCTGGAGGGGTATCA
GGAGGTCACGTAAATCCTGCCGTGAGTCTTGCCATGGCAGTATGGGGCAAGTTT---CCC
TGGGCAAAGGTTCCAATCTACATGGTGGCACAGTACCTTGGGGCCTTCTTCGCGAGTGTC
TTGCTGTACGGAGTATATCTCAATGCCCTCGATAACTTCGAC---CCTGGC---------
CGC------------------------------ACAGATCTAACTGCTGGCATCTGGGCC
ACATACCCTGGTGTGGCA---CTACTCAATGGAACCAAT---GAATCTACAGCCTTCCTC
TCAGCTGGAAATGGCATGAGTGATCAGATTGTGGGCACCATGTTGTTACTGATTTGTGTG
TGTGCCATCACTGATCCAAGGAATATGCAAGTTCCCAAGGCTCTCGTTCCTTTCTTCGTT
GGAATGACGGTTCTGAATATCGGCATCTGCTTTGGATTTAACTGTGGTTATGCCATCAAC
CCCGCTCGAGATCTCGGTCCAAGAATATTCACCCTCATCGCTGGATGGGGCGACCACCCA
TTCACTGCCATGACCGTTGACAGTGTAGCATGGTGGTGGATCCCAATCATTGGGCCACAC
ATTGGGGCTATCATTGGAGTTTTTGTTTACCTGGTCTTC---ATAGAGCTACATCACACA
GAGGCAGCGGAGTTCAATCTACCACAAGTTCAG------
>GETT01055162_Meganyctiphanes_norvegica_Glp2
ACACTTCTGTTGGAGTATGTTCGAAAGCTGATGCAC---ATAAAGAACCCCCTCATCAAA
GAATTTCTAGCAGAGTTCCTAGGAACATTTATACTAATGATGTTTGGTGATGGTTCAGTG
GCACAAAGCGTTCTCACAGGTCAA---------------GCTAATGGAGACGTCTTCTCA
ATCAACTGGGGCTGGGCTGTGGCTGTCATGCTGGCTGTGTTGGTATCTGGAGGGGTGTCA
GGAGGTCACGTAAATCCTGCCGTGAGTCTCGCCATGGCAGTATGGGGCAAGTTT---CCC
TGGGCAAAGGTTCCAATCTACATGGTGGCACAGTACCTTGGGGCCTTCTTCGCGAGTGTC
TTGCTGTACGGAGTATATCTCAATGCCCTCGATAACTTCGAC---CCTGGC---------
CGC------------------------------ACAGATCTAACTGCTGGCATCTGGGCC
ACATACCCTGGTGTGGCA---CTACTCAATGGAACCAAT---GAATCTACAGCCTTCCTC
TCAGCTGGAAATGGCATGAGTGATCAGATTGTGGGCACCATGTTGTTACTGATTTGTGTG
TGTGCCATCACTGATCCAAGGAATATGCAAGTTCCCAAGGCTCTCGTTCCTTTCTTTGTT
GGAATGACGGTTCTAAATATCGGCATCTGCTTTGGATTTAACTGTGGCTATGCCATCAAC
CCCGCTCGAGATCTCGGTCCAAGAATATTCACCCTCATCGCTGGATGGGGCGACCACCCA
TTCACTGCCATGACCGTTGACAGTGTAGCATGGTGGTGGATCCCAATCATTGGGCCACAC
ATTGGGGCTATCATTGGAGTTTTTGTTTACCTGGTCTTC---ATAGAGCTACATCACACA
GAGGCAGCGGAGTTCAATCTACCACAAGTTCAG------
>GEQI01055161_Hyalellopsis_setosa_Glp
TTCAACGTGCTGGGCAAGCTCGGGTCCTTGGTCGTG---CTCCGCAACGCCACCGTCAAG
GAGTTCCTGGCAGAGATGCTGGGCACCTTCATTCTCGTGTTCATGGGCAACGGCGCGGTG
GCCCAGACCGTCATGTCGGGCGGT---------------GAACGTGGGGACTTCTTCTCC
ATCAACATGGGCTATGCCGTGGCCGTCTTCCTGGGGGTCATGGTTTCGGGGGGCATCAGC
GGGGGGCACCTCAACCCTGCAGTCAGTGTGTCGCTGGCTGTGTTTGGCAAGTTC---TCG
TGGCTTAAGGTGCCGGTGTACCTCGTGGCTCAGTATATGGGCGCCTTCACAGCCTCCCTT
ATGCTCTTCCTCGTCTACAGAAATGCCCTGGACGCGTATGAC---CCTCAG---------
CGC------------------------------ACCGTGGCATCAGCGGGCATCTTCGCC
ACGTACCCCGGGTCTGTG------ATCAATGGCACAGGC---GTCCACGTGGACTTCCTC
AGCAGCGGCAACGGGATGGGCGACCAGATCATGGGCACGCTGGTGCTGTTGCTGTGTGTG
AGCGCCATCACCGACCCCCGTAACATGGCCATCCCCAAGCCTCAGATCCCCGCCGCTGTC
GCCGTCGTCGTGCTGGGCATCGGCCTCAGTCTGGGCCTCAACTGCGGCTATGCCATCAAC
CCAGCACGAGACCTGGGCCCCAGGCTGTTCACGCTCATCGCTGGCTGGGGCGCCGACACC
TTCACCACCAGCACTGTTGACGGCGTGGCCTGGTGGTGGGTGCCTGTGGTGGGGACCCAC
CTGGGCGGCATCCTGGGAGCGGGGCTCTACTGCCTCCTG---GTGGAGCTGCACCACGAG

GAGGACGAGGATCAGCACGACCTCGACCTT---------
>GEQF01044437_Hyalellopsis_carinata_Glp
TTCAACGTGCTGGGCAAGCTCGGGTCCGTGGTCGTA---CTCCGCAACGCCACCGTCAAG
GAGTTCCTGGCAGAGATGCTGGGCACCTTCATTCTCGTGTTCATGGGCAACGGCGCGGTG
GCCCAGACCGTCATGTCGGGCGGC---------------GAACGAGGGGACTTCTTCTCC
ATCAACATGGGCTATGCCGTGGCCGTCTTCCTGGGGGTCATGGTTTCGGGGGGCATCAGC
GGGGGGCACCTCAACCCTGCAGTCAGTGTGTCGCTGGCTGTGTTTGGCAAGTTC---TCG
TGGCTTAAGGTGCCGGTGTACCTCGTGGCTCAGTATATGGGCGCCTTCACAGCCTCCCTT
ATGCTCTTCCTCGTCTACAGAAATGCCCTGGACGCGTATGAC---CCTCAG---------
CGC------------------------------ACCGTGGCATCAGCGGGCATCTTCGCC
ACGTACCCCGGGTCTGTG------ATCAATGGCACAGGC---GTCCACGTGGACTTCCTC
AGCAGCGGCAACGGGATGGGCGACCAGATCATGGGCACGCTGGTGCTGTTGCTGTGCGTG
AGCGCCATCACCGACCCCCGTAACATGGCCATCCCCAAGCCTCAGATCCCCGCCGCTGTC
GCCGTCGTCGTGCTGGGCATCGGCCTCAGTCTGGGCCTCAACTGCGGCTATGCCATCAAC
CCAGCACGAGACCTGGGCCCCAGGCTGTTCACGCTCATCGCTGGCTGGGGCGCCGACACC
TTCACCACCAGCACTGTTGACGGCGTGGCCTGGTGGTGGGTGCCTGTGGTGGGGACCCAC
CTGGGCGGCATCCTGGGAGCGGGGCTCTACTGCCTGCTG---GTGGAGCTGCACCACGAG
GAGGACGAGGATCAGCACGACCTCGACCTT---------
>GHHW01012848_Eulimnogammarus_cyaneus_Glp
TTCAACGTGCTGGGCAAGCTCGGGTCCGTGGCCGTG---CTCCGCAACGCCACCGCCAAG
GAGTTCCTGGCAGAGATGCTGGGCACCTTCATTCTCGTGTTCATGGGCAACGGCGCGGTG
GCCCAGACCGTCATGTCGGGCGGT---------------GAACGAGGGGACTTCTTCTCC
ATCAACATGGGTTATGCCGTGGCCGTGTTCCTGGGGGTCATGGTTTCGGGGGGCATCAGC
GGGGGGCACATCAACCCCGCAGTCAGTGTGTCGCTGGCTGTGTTTGGCAAGTTC---TCG
TGGCTTAAGGTGCCGGTGTACCTCGTGGCTCAGTATATGGGCGCCTTCACAGCCTCCCTC
ATGCTCTTCCTCGTCTACAGAAATGCCCTGGACGCGTATGAC---CCTCAG---------
CGC------------------------------ACCCTGGCATCAGCGGGCATCTTCGCC
ACGTACCCCGGGTCTGTG------GTCAATGGCACAGGC---GTCCACGTGGACTTCCTC
AGCAGCGGCAACGGGATGGGCGATCAGATCATGGGCACGCTGGTGCTGTTGCTGTGTGTG
AGCGCCATCACCGATCCCCGTAACATGGCCGTCCCCAAGCCTCAGATCCCCGCCGCTGTC
GCCGTCGTCGTGCTGGGCATCGGCCTCAGTCTCGGCCTCAACTGCGGCTATGCCATCAAC
CCAGCACGAGACCTGGGGCCCAGGCTGTTCACGCTCATCGCTGGCTGGGGCGCCGACACC
TTCACCACCGGCACTGTTGACGGCGTGGCCTGGTGGTGGGTGCCTGTGGTGGGGACCCAC
CTGGGCGGCATCCTGGGAACGGGCCTCTACTGCCTGCTG---GTGGAGCTGCACCACGAG
GAGGACGAGGATCAGCACGACCTCGACCTT---------
>GEQQ01047519_Odontogammarus_calcaratus_Glp
TTCAACGTGCTGGGCAAGCTCGGGTCCGTGGCAGTG---CTCCGCAACACCACCGTCAAG
GAGTTCCTGGCAGAGATGCTGGGCACCTTCATTCTCGTGTTTATGGGCAACGGCGCGGTG
GCCCAGACCGTCATGTCGGGCGGT---------------GAACGAGGGGACTTCTTCTCT
ATCAACATGGGCTATGCCGTGGCCGTGTTCCTGGGGGTCATGGTTTCGGGGGGCATCAGC
GGGGGGCACCTCAACCCCGCAGTCAGTGTGTCGCTGGCTGTGTTTGGCAAGTTC---TCG
TGGCTTAAGGTGCCGGTGTACCTCGTGGCTCAGTATATGGGCGCCTTCGCAGGCTCCCTC
GTGCTCTTCCTCGTCTACAGAAATGCCCTGGACGCGTATGAC---CCTCAG---------
CGC------------------------------ACCCTGGCATCAGCGGGCATCTTCGCC
ACGTACCCCGGGTCTGTG------ATCAATGGTACAGGC---GTCCACGTGGACTTCCTC
AGCAGCGGCAACGGGATGGGCGACCAGATCATGGGCACGCTGGTGCTGTTGCTGTGTGTG
AGCGCCATCACCGACCCCCGTAACATGGCCGTCCCCAAGCCTCAGATCCCCGCCGCTGTC
GCCGCCGTCGTGCTGGGCATCGGCCTCAGTCTCGGCCTCAACTGCGGCTATGCCATCAAC
CCAGCACGAGACCTGGGGCCCAGGCTGTTCACGCTCATCGCTGGCTGGGGCGCCGACACC
TTCACCACCGGCACTGTGGACGGCGTGGCCTGGTGGTGGGTGCCTGTGGTGGGGACCCAC
CTGGGCGGCATCCTGGGAGCAGGGCTCTACTGCCTGCTG---GTGGAGCTGCACCACGAG
GAGGACGAGGATCAGCACGACCTCGACCTT---------
>GEQS01030819_Ommatogammarus_flavus_Glp
TTCAACGTGCTGGGCAAGCTCGGGTCAGTGGCCGTG---CTCCGCAACACCACCGTCAAG
GAGTTCCTGGCAGAGATGCTGGGCACCTTCATTCTCGTGTTCATGGGCAACAGCGCGGTG
GCCCAGACCGTCATGTCGGGCGGT---------------GAACGGGGGGACTTCTTCTCC
ATCAACATGGGCTACGCCGTGGCCGTGTTCCTGGGGGTCATGGTTTCGGGGGGCATCAGC
GGGGGGCACATCAACCCCGCCGTCAGTGTGTCGCTGGCTGTGTTTGGCAAGTTC---TCG
TGGCTTAAGGTGCCGGTGTACCTCGTGGCTCAGTATATGGGCGCCTTCACAGGCTCCCTC
ATGCTCTTCCTCGTCTACAGAAATGCCCTGGACGCGTATGAC---CCTCAG---------
CGA------------------------------ACCCTGGCATCAGCGGGCATCTTCGCC
ACGTACCCCGGGTCTGTG------ATCAATGGCACAGGC---GTCCACGTGGACTTCCTC
AGCAGCGGCAACGGGATGGGCGACCAGATCATGGGCACGCTGGTGCTGTTGCTGTGTGTG
AGCGCCATCACCGACCCCCGTAACATGGCCGTGCCCAAGCCTCAGATCCCCGCCGCTGTC
GCCGTCGTCGTGTTGGGCATCGGCCTCAGTCTCGGCCTCAACTGCGGCTACGCCATCAAC
CCAGCACGAGACCTGGGGCCCAGGCTGTTCACGCTCATCGCTGGCTGGGGCGCCGACACC
TTCACCACCGGCACTGTTGACGGCGTGGCCTGGTGGTGGGTGCCTGTGGTGGGGACCCAC
CTGGGCGGCATCCTGGGAGCGGGGCTCTACTGCCTGCTG---GTGGAGCTGCACCACGAG

GAGGACGAGGATCAGCACGACCTCGACCTT---------
>GEQN01037202_Micruropus_glaber_Glp
TTCAACGTGCTGGGCAAGCTTGGGTCCGTGGCGGTG---ATTCGTCACCCCCTCGTCAGG
GAGTTCCTGGCTGAGATGCTGGGCACCTTCATCCTCGTGTTCATGGGCAACGCAGCGGTG
GCTCAGACCGTCATGTCGGGCGGT---------------GAACGGGGCGACTTCTTCTCA
ATCAACGTGGGCTACGCCGTGGCTGTGTTCCTGGGGGTCATGGTTTCGGGGGGCGTTAGT
GGAGGGCAACTAAACCCCGCCGTCAGTGTGTCGCTGGCTGTGTTTGGCAAGTTC---TCG
TGGGCAAAAGTGCCGGTGTACCTCGTGGCTCAGTATTTGGGAGCCTTCACAGGCGCCGCT
ACGCTCTTCCTCGTCTACAGAAATGCCCTGGACGCGTATGAC---CCTCAG---------
CGC------------------------------ACCCTGGCATCAGCGGGCATCTTCGCC
ACGTACCCGGGGTCTGTG------GTCAATGGCACAGGC---GTCCATGTGGACTTCCTC
AGCAGCGGCAACGGGATTGGTGATCAGATCATGGGCACGTTTGTGCTGCTGCTGTGTGTG
TGCGCCATCACCGACCCGCGTAACATGGCCATCCCCAAGCCGCAGATACCCGCCGCTGTC
GGCGTCGTCGTGCTGGGCATCGGCATCAGTCTCGGCCTCAACTGTGGCTATGCCATCAAT
CCTGCACGAGACCTCGGCCCCAGGCTGTTCACGCTCATCGCGGGCTGGGGCGCCGAGACC
TTTACCACCGGTACTGTTGACGGCGTGGCCTGGTGGTGGGTGCCTGTGGTGGGCCCCCAC
CTGGGTGGCATCCTGGGAGCGGGGCTCTACAGTCTGGTG---GTGGAGCTGCACCACGGG
AACGACGAGGATCAGCCCGAACGCGCCTCT---------
>GEQP01069426_Micruropus_wahlii_Glp
TTCAACGTGTTCGCCAAGCTTATGCCCTTCATGGTG---ATTCGCAACTCCCTCGTCAAG
GAGTTCCTGGCTGAGTTCCTGGGCACCTTCATCCTCGTGTTTATGGGCAACGCAGCGGTG
GCCCAAACCGTCATGTCCGGCGGT---------------GAACGGGGCGACTTCTTCTCC
ATCAACGTGGGCTACGCCGTGGCTGTGTTCCTGGGGGTCATGGTGTCGGGGGGCGTTAGT
GGGGGGCACATAAACCCCGCCGTCAGTGTGTCGCTGGCCGTGTTCGGCAAGTTC---CCG
TGGGCCAAAGTGCCGGTGTACCTCGTGGCTCAGTATCTAGGAGCCTTCACAGGTGCCGCC
GCGCTCTTCCTCACCTACAGAAATGCCCTGGACGCGTATGAC---CCTCAG---------
CGC------------------------------ACCCTGGCGTCAGCGGGTATCTTCGCC
ACGTACCCGGGGTCTGTG------GTCAATGGCACAGGC---ACCCACGTGGACTTCCTC
AGCAGCGGCAACGGGTTAGGTGATCAGATCATGGGCACGCTGGTGCTGGTGCTGTGTGTG
TGCGCCATCACCGACCCCCGTAACATGGCCCTCCCCAAGTCGCAGATACCCGCCGCTGTC
GCCATCGTCGTGCTGGGCATCGGCATCAGTCTCGGCCTCAACTGCGGCTATGCCATCAAT
CCTGCACGAGACCTCGGGCCCAGGCTGTTCACGCTCATCGCGGGCTGGGGCTCCGAGACC
TTTACCACCGGTACTGTTGACGGCGTGGCCTGGTGGTGGGTGCCTTTGGTGGGCGACCAC
GTGGGTGGCATCCTGGGAACGGGGATCTACCATCTGGGG---GTGGAGGTGCACCACAAG
AACGACGAGGATCTGCCCGTACGCATCACT---------
>GEQO01051209_Micruropus_parvulus_Glp
TTCAACGTGCTGAGCAAGCTCGGGTCCGTGGTGGTG---CTCAAGAACATCCTCGTCAAG
GAGTTCCTGGCAGAGATGCTCGGCACCTTCATACTCGTGTTTATGGGCAACGCGGCGGTC
GCCCAGACCGTCATGTCGGGGGGT---------------GAACGGGGCGACTTCTTCTCC
ATCAACATGGGCTACGCGGCGGCGGTGTTCCTGGGGGTCATGGTTTCGGGGGGCATTAGT
GGGGGGCACCTCAACCCCGCCGTCAGTGTGTCGCTGGCCGTGTTTGGCAAGTTC---CCG
TGGACCAAGGTGCCGGTGTACCTCGTGGCCCAGTATACGGGCGCCTTCGCAAGCTCCGCC
ATGCTCTTCCTCGTCTACAGGAATGCCCTGGACGCGTATGAC---CCTCAG---------
CGC------------------------------ACCCTGGCGTCAGCGGGTATCTTCGCC
ACGTACCCGGGGTCGGTG------ATCAACGGCACAGGC---GTCCATGTGGACTTCCTC
AACAGCGGCAACGGGATGGGTGACCAGATCATGGGCACGCTGGTGCTGATGGTGTGCGTG
TGCGCCATCACCGACCCCCGCAACATGGCCGTCCCCAAGCATCAGATCCCCGCCGCTGTC
GCCGCCGTCGTGCTGGGCATCGGCATCAGTCTGGGCCTCAACTGCGGCTATGCCATCAAC
CCAGCACGCGACCTCGGGCCCAGGCTGTTCACCCTCATCGCTGGCTGGGGCGCCGAGACC
TTTACCTCCGGCACTGTTGACGGCGTGGCCTGGTGGTGGGTGCCTGTGGTGGGTACCCAC
CTGGGCGGCATCCTGGGAGTGGGGCTCTACTGGCTGCTG---GTGGAGCTGCACCACACG
CACGACGAGGCCCAGCACGACCTCGCCCAGGACCTCGCC
>GHDB01297872_Gammarus_fossarum_Glp
TTCAACGTGCTCGGCAAGCTCGGGTCCGTGGCCGTG---CTTCGCAATACCATCGTCAAG
GAGTTCCTGGCGGAGATGCTGGGCACCTTCATACTCGTGTTCATGGGCAACGGCGCCGTG
GCCCAGACCGTCATGTCGGGCGGT---------------GCACGTGGCGATTTCTTCTCA
ATCAACATGGGCTACGCCGTGGCTGTGTTCCTGGGTGTCATGGCTTCGGGGGGCGTTAGT
GGGGGACACATCAACCCCGCTGTCAGTGTTTCGCTGGCTGTGTTTGGCAAGTTC---TCG
TGGGCTAAGGTGCCAGTGTACCTCGTGGCTCAGTATATGGGCGCCTTCACAGGCTCCCTC
ATGCTCTTCCTCGTCTACAGAAATGCCCTGGACGCCTATGAC---CCTCAG---------
CGC------------------------------ACCCTGGCAACAGCGGGCATCTTCGCC
ACGTATCCCGGATCTGTG------GTCAATGGCACAGGC---GTCCATGTGGACTACCTC
AGCAGCGGCAACGGGTTGGGCGACCAGATCATGGGCACGATGGTGCTGTTGCTGTGCGTG
TGCGCCATCACCGACCCCCGTAACATGGCCGTCCCCAAGCCTCAGATCCCCGCCGCTGTC
GCTGCCGTCGTACTAGGCATCGGCCTCAGTCTCGGCCTCAACTGCGGCTATGCCATCAAC
CCAGCACGAGACCTGGGGCCCAGGCTGTTCACGCTCATCGCTGGCTGGGGCGCCGAGACC
TTCACAAGCGGTACTGTTGACGGCGTGGCCTGGTGGTGGGTGCCTGTGGTGGGCACCCAC
CTGGGCGGCATCCTGGGAGCGGGGCTCTACTGCCTGCTG---GTGGAGCTGCACCACGCT

GAGGACGAGGACCAGCACGACCTCGACATG---------
>GHCP01013510_Gammarus_pulex_Glp
TTCAACGTGCTGGGCAAGCTTGGGTCCGTGGTTATG---CTTCGCAACACCACCGTCAAG
GAGTTTCTCGCAGAGATGCTGGGCACCTTCATACTCGTGTTTATGGGCAACGGCGCGGTG
GCCCAGACCGTCATGTCGGGCGGT---------------GCTCGGGGCGATTTCTTCTCC
ATCAACATGGGCTACGCCGTGGCTGTGTTCCTGGGGGTCATGGCTTCGGGGGGCGTTAGT
GGGGGGCACATCAACCCCGCCGTCAGTGTGTCGCTGGCTGTGTTTGGCAAGTTC---TCG
TGGGCTAAGGTGCCAGTGTACCTCGTGGCTCAGTATACGGGCGCCTTCACAGGCTCTCTC
ATGCTCTTCCTCGTCTACAGAAATGCCTTGGACGCGTATGAC---CCTCAG---------
CGC------------------------------ACCCTGGCAACAGCGGGTATCTTCGCC
ACATACCCCGGGTCTGTG------GTCAACGGCACAGGC---GTTCATGTGGACTACCTC
AGCAGCGGCAACGGGTTGGGCGACCAGATCATGGGCACGCTGGTGCTGTTGCTGTGCGTG
TGCGCCATCACCGACCCCCGTAACATGGGTGTCCCCAAGCCTCAGATCCCCGCCGCTGTC
GCGGCCGTCGTGCTGGGCATCGGCCTCAGTCTCGGCCTCAACTGCGGCTATGCCATCAAC
CCAGCACGAGACCTGGGGCCCAGGTTGTTCACGCTCATCGCGGGATGGGGCGCCGAGACA
TTCACAACCGGTACTGTTGACGGCGTGGCCTGGTGGTGGGTGCCTGTGGTGGGCACCCAC
CTGGGCGGCATCCTGGGAGCGGGGCTCTACTGCCTGCTG---GTAGAGCTGCACCATGAT
GAGGACGAGGACCACCACGACCTCGACAAG---------
>IACT01002934_Hirondellea_gigas_Glp
CAGAACGTGCTTGAGAAGCTGACGGTCTACACCGCC---CTGCGCAACACCACAGCCAGA
GAGTTCCTGGCGGAGATGCTGGGCACATTCATCCTCGTCGTGATGGGGAACGGAGCTGTG
GCGCAGGTGGTGCTCTCTGGAGGA---------------GGGAAGGGCGACTTCTTCTCC
ATCAACGTAGGGTACGCCATGGCCGTGTACCTGGGGGTCATGGTCTCTGGTGGCATCAGT
GGGGGACACATCAACCCTGCCGTCAGTTTGTCCATGGCCCTGTTTGGCAAGTTC---CCG
TGGGTGAAGGTGCCGGTTTACATGGTCGCACAGTATTCGGGAGCCTTCATTGGGTCTATC
GTGATCTTCTTTGTCTACAGAAATGCTCTTGACACCTACGAT---CCGGGT---------
CGA------------------------------ACACTGGCATCTGCGGGAGTGTTCGCC
ACCTACCCAGGCTCAGTT------GTCAACAGCACCGGGGCAGTGATCTCGGACTTCCTC
ACCAGCGGCAACGGCTTGGGTGACCAGATCTTAGCAACCTGCATCCTACTTTTGTGCGTC
TGCGCCATCACCGATTCACGCAACATGGGAATCCCTAAAGCCCAGATCCCCGCGTGTGTG
GCCGGGGTTGTGCTGTCCATCGGGTTGAGCTTGGGGCTCAACTGTGGGTACGCCATCAAT
CCCGCCAGGGATTTGGGTCCCAGGCTCTTCACCCTCATAGCCGGCTGGGGAGCAGAAACT
TTCACTGCCAGTACGAGTGATGGCGTTGCGTGGTGGTGGGTTCCGATAGTTGGTCCTCAC
ATCGGAGGCATACTAGGTGTGGGCATCTACTACATGGTC---ATCGAGCAGCACTACGGA
GCAGAACCTGAGAACCTCGACCTCGAGAAA---------
>VCRD01002769_Trinorchestia_longiramus_Glp1
TTCAACGTGCTGGACAAGCTGGGCTCCGTGGCTGTG---ATACGGAGCCCCGTCGTGAAG
GAGTTTCTTGCTGAGATGCTGGGTACTTTCATCCTGGTGCTGATGGGGAACTGCGCGGTG
GCGCAGGCGGTGATGGCCGAGGAT---------------------GGCAGTTTCCTCTCC
ATCAACATCGGCTACGCGGTGGGGGTAACGCTAGGAGTCCTGGTCTCGGGGGGCATCAGC
GGAGGTCACATCAATCCGGCCGTCAGCGTAGCCATGGCTGTGTTCGGCAAGTTT---TCG
TGGGCAAAAGTACCAGTGTACATAGTAGCTCAGTACACTGGGGCCTTTGCTGGTGCCCTC
ATGGTCTTCCTCGTCTACAGGGACGCCCTGGATGTGTATGAC---CCTGAA---------
AGA------------------------------ACGCTGTCGAGCGCCGGTATCTTTGCT
ACGTACCCTGGAGCTGTG------ATCAACTCTACGGGAGACACGGTTGACAACTTTCTG
TACAGCGGCAACGGCATGGCTGACCAGGTGCTGGGCACAGCTGTACTGCTGGTGTGCGTG
TGTGCCATCACTGACCCACGCAACATGGGCATCACCAAGCCCATGATCCCCGTCGCTGTG
GGCACCGTCGTGCTGGGGATTGGGCTGAGCTTGGGGCTCAACTGTGGCTATGCGATTAAC
CCCGCGAGGGACTTGGGGCCCCGTCTTTTCACCCTCGTTGGGGGCTGGGGAGCTGAACCC
TTCACGGCGAGCACTGCTGACGGTGTGGCGTGGTGGTGGATCCCTGTTGTAGGTCCCCAT
TTGGGCGCCATCTTGGGTGTCTTCTTCTACTCAATAATC---ATCGAACTACATCACGGA
TCAGAAATGCCTGATGGAAAAGTTGACCCT---------
>GDUJ01066534_Talitrus_saltator_Glp
TTCAACGTACTGGACAAGCTGGGCTCTGTAGCTGTG---ATACGCAACTCCACCGTGAAG
GAGTTCTTAGCAGAGATGATCGGCACATTCATCCTGGTGCTGATGGGGAACTGCGCGGTG
GCGCAGGGGGTGATGTCAGAAGAC---------------------GGGAGCTTCCTGTCC
ATCAACTTCGGGTACGCGGTGGGGGTGACTCTCGGAGTGATGGTGTCGGGGGGCGTCAGT
GGGGGACACATCAACCCCGCCGTCAGCGTGGCTTTGGCTGTGTTCGGAAAGTTC---TCA
TGGGTCAAAGTGCCCATCTACATCGTGGCGCAGTATACGGGGGCCTTCGCGGGTGCCCTC
ATGGTGTTCCTCGTGTACAGAAACGCTTTGGATACATTTGAC---CCTGAG---------
AGG------------------------------ACACTTGCGAGCGCCGGTATCTTTGCT
ACGTACCCGGGCGCTGTG------ATTAACTCGACCGGCGATGTGGTCGACGACTACCTC
TACAGCGGCAATGGAATGGCTGACCAGGTGTTGGGCACGGCAGTGTTGCTGGTGTGCGTT
TGCGCCATCACCGATCCCCGCAACATGGGGATCACGAAGCCCATGATCCCCGTTGCCGTG
GGGACCGTCGTGCTGGGGATTGGGCTTAGCCTGGGGCTCAACTGCGGCTATGCCATCAAC
CCCGCCAGAGACCTCGGGCCTCGTCTCTTCACCCTCGTAGGAGGGTGGGGCGCCGAGCCC
TTCACGGCCAGCACTGCTGACGGAGTAGCGTGGTGGTGGATCCCTGTGGTCGGACCTCAT
GTGGGCGCCATCTTGGGAGTCTTCTTCTACTCTATCATC---ATCGAACTCCACCACGGA

TCAGACACGCCCGACGGAAAAGTTGATCCT---------
>VCRD01000483_Trinorchestia_longiramus_Glp2
ATCAAC---ATGACCAAATTTGGCTCTAAGTCCATC---ATCCGTAGCACTGCAGGCAAG
GAGTTCTTGGCCGAGATGCTCGGCACTTTCATGCTTGTGTTCCTGGGCGACTGCGCGGTG
GCGCAATCTGTCATGTCCCAG---------------------AGAGGTAGCCTTCTTTCT
ATCGACCTGGGTGTTGCTGTTGGAGTGGCTGCTGGAATTTTGGTGTCCGGAGGTGTAAGT
GGAGGACACATCAACCCGGCTGTCAGCGTGGCGTTGGCGGTGTTTGGAAAGTTC---TCG
TGGTCGAAAGTAGCGGTGTATCTCGCTGCTCAGTACACAGGGGCTTTTCTGGCTGCCCTC
ATGGTGTTTCTTGTCTACAAGGATGCTTTAGACGTGTATGAC---CCAGAG---------
AGG------------------------------ACCATGGCCAGCGCAGGTATCTTTGCT
ACCTACCCTGGTAGCGTG------ATGGACGAAAACGGAATGTTATCCAGAACTTTCTC
TCCACCGGTAACGGATTTGCAGACCAGGTTTTAGGAGCAATGATATTGCTACTGGGTGTT
TGCGCTATAACGGACATACGGAATATGAAGTTCACGAAACCCATGATACCAGCATCTGTG
AGCATTGTCGTGCTTGGCATCGGCATCAGCCTGGGCATGAACTGTGGGAACGCCATTAAC
CCTGCAAGAGATTTCGGACCCCGGCTTTTCACATCCGTTGCTGGCTGGGGTGTGGAGCCA
TTCACGTACACCACAGTTGATGGCGCTGCCTGGTGGTGGATACCCGTGGTGGCCCCTCAC
TTAGGCGCAATTTTAGGGGTCTTCTTGTACGCTATCATG---GTCGAACAGCACCAT---

>NW_017252273_Hyalella_azteca_Glp
TTTGGCGTGATGGAAAAGTTCAGCGCGGTGGCGGCG---ATCAAGAACGAAGCTATTCGC
GAGTTCCTCGCTGAATTGATCGGAACATTCGTGCTCGTGATGATGGGCAACTGTGCGGTG
GCCCAGGTTGTGATGGCCGAAGGT---------------------GGCAGCTTCTTGTCC
ATCAACATGGGCTACGCCGTAGCGGTCACTCTTGGCGTCATGGTGTCAGGAGGCGTGAGT
GGTGGACACATCAACCCGGCCGTCAGTGTGGCGCTGGCCGTGTTCGGCAAGTTC---CCG
TGGGCGAAAGTACCGATCTTTATAGTGGCGCAGTACACAGGTGCCTTCCTCGGTGCCTTC
ATGGTTTTCCTCGTCTACAGAGACGCACTGGACGCGTTCGAC---CCTGGC---------
CGG------------------------------ACACTGGCGAGCGCTGGTATCTTCGCT
ACGTATCCCGGGTCGGTG------GTGAACTCAACCGGCGAAGTGGTAGACAACTTCCTG
TACAGCGGCAACGGCATGGCAGATCAGGTTGTGGGGACTGCCATGTTGCTGCTATGCGTA
TGTGCCATCACTGATCCCCGCAACATGGGGATTCCCAAGCCCATGATCCCCGCCGCCGTG
GGCACAGTAGTGCTGGGCATTGGGCTCAGCCTGGGGCTCAACTGCGGGTACGCAATCAAC
CCCGCCAGGGACCTGGGGCCCCGGCTGTTCACCCTCGTCGCGGGGTGGGGCGCTGAGCCC
TTCACGGCTATGACAGCCGATGGGGTGACATGGTGGTGGATCCCTGTGGTCGGACCACAC
TTGGGT--

>LQNS02278070_Parhyale_hawaiensis_Glp
TTGAACATTATGGAGAAGCTCCAGTCTGTTTGCTCA---ATCAGAAATCAAGCAGCAAGA
GAGTTCTTTGCCGAGTTCCTGGGCACGTTTCTGCTGGTGCTGATGGGCAACTGCGCGGTG
GCGCAAGTGGTGATGGCAGAGGGT---------------------GGCAGCTTTCTGTCC
ATCAACATGGGCTACGCGGTCGGAGTGACGCTGGGTGTGATGGCCTCCGGGGGCATCAGC
GGTGGCCACCTAAACCCTGCCGTCAGCGTGGCGATGGCAGTGTTCGGGAAGTTC---TCG
TGGGCCAAAGTTCCCGTGTACATCGTGGCGCAATACGTCGGGGCTTTCTTTGGCGCTCTC
ATGGTCTTCCTTGTGTATAGAGACGCCCTGGATGCATTTGAC---CCTGAC---------
CGA------------------------------ACCCTCGCCAGTGCCGGCATATTCGCC
ACCTACCCTGGCTCAGTC------ATCAATGCCACGGGTGGTCTTGTGGACAACTTCCTG
TACAGTGGGAACGGTATGGCTGACCAGGTGCTGGGCACATGTGTATTACTGGTGTGCGTG
TGCGCCATCACAGACTCCCGTAACATGGGCCTCACGAAGCCAATGATCCCCGCTGCTGTA
GGCACCGTTGTCCTCGGCATCGGCCTCAGTCTTGGCCTCAACTGTGGCTACGCCATCAAC
CCCGCCAGGGATCTGGGACCTAGGCTTTTCACCCTTATCGCTGGTTGGGGCTCCGACACA
TTCACGCTCAGCACCGCTGACGGCGTCGCCTGGTGGTGGATCCCGGTCGTAGGGCCTCAC
ATTGGCGCTATTTTGGGTGTATTCTTCTACAGCATCCTT---GTGGAGCTGCACTACGGT
GCTGAATCGGAAGACTCCAAACCTGACCCAGAA------
>HAEW01040360_Proasellus_ebrensis_Glp
ATTGATGTTGTGGATTGGTTGCGCAAACACTATGCG---ATACAAAAACAAGTTGTTAAA
GAATTCTTAGCCGAATTTCTGGGGACATTTTTGCTTATCGTTTTCGGTAATGGAGCAGTT
GCCCAAAGCGTGTTGACAGCGGGG---------------ACAGTTAATAACTATTTTTCT
GTAATCTGGGGGTACTCCCTCGGGGTAGTGATGGGCGTTATGGTGTCTGGTAATGTCAGC
GGAGGTCACATCAACCCTGCAGTAACATTCGCTATGGCCCTTTGGGGAAAACTA---CCT
TGGTCAAAGGTTCCAGTCTACATGGTGGCCCAATACTTAGGTGCCTTTGCTGCTTCCGTT
TGTCTCTATGGAGTCTATACAGACGCTATTCATACTTACGAA---CCGACT---------
CAT------------------------------ACTATGGCCACCGCTGGAATATGGGCT
ACTTATCCTGGAACGATG------ATCAACGCCAATGGAACGGAAGTTGAAAGCTATTTG
ACTGCTGGAAATGGATTTGGTGATCAGGTATTTGGAACCATGCTCCTACTGATTTGCGTC
TGCGCTATTAATGACCAAAGAAACATGAATGTCCCTCAATGGATTTCTCCTCTTTTCGTG
GGTTTCGTGGTGATGAACATTGGTCTCAGCTTTGGATTTAACTGTGGGTTCGCCCTCAAC
CCTGCAAGAGATTTGGCCCCTAGATTCTTCACTATGATTGCTGGCTGGGGAAGTGAACCA
TTCACAGCAACTACTGTCGATGGTGTGAGTTGGTGGTGGATCCCAGTGGTGGCCACTCAT
ATTGGAGCAATTCTTGGAATTTTGGTCTACACCATTTTC---ATCGAAATGCATCATAAG

GAAAATAAGGACTCCGAGCTTCCAAAAGCCCAA------
>HAFF01020158_Proasellus_ortizi_Glp
ATTGATGTTGTGGATTGGTTGCGCAAACACTATGCG---ATACAAAAACAAGTTGTTAAA
GAATTCTTAGCCGAATTTCTGGGGACATTTTTGCTTATCGTTTTCGGTAATGGAGCAGTT
GCCCAAAGCGTGTTGACAGCGGGG---------------ACAGTTAACAACTATTTTTCT
GTAATCTGGGGGTACTCCCTTGGGGTAGTGATGGGCGTTATGGTGTCTGGTAATGTCAGC
GGAGGTCACATCAACCCTGCAGTAACATTCGCTATGGCCCTTTGGGGAAAACTA---CCG
TGGTCAAAGGTTCCAGTCTACATGGTGGCTCAATATTTAGGGGCCTTTGCTGCTTCCGCC
TGTCTCTATGGAGTCTATACAGACGCTATTCATACTTACGAA---CCGACT---------
CAT------------------------------ACTATGGCCACCGCTGGAATATGGGCT
ACTTATCCTGGAACGATG------ATCAACGCCAATGGAACGGAAGTTGAAAGTTATTTG
ACTGCAGGAAATGGATTTGGTGATCAGGTATTTGGAACCATGCTTCTACTGATTTGCGTC
TGCGCAATTAACGACCAAAGAAACATGAATGTCCCTCAATGGATTTCTCCTCTTTTCGTG
GGTTTCGTGGTGATGAACATTGGTCTCAGCTTTGGATTTAACTGTGGGTTCGCCCTCAAC
CCTGCAAGAGATTTGGCCCCTAGATTCTTCACTATGATTGCTGGCTGGGGAAGTGAACCA
TTCACAGCAACTACTGCCGATGGTGTGAGTTGGTGGTGGATCCCAGTGGTGGCCACTCAT
ATTGGAGCAATTCTAGGAATTTTGGTCTACACCATTTTC---ATCGAAATGCATCATAAG
GACAATAAGGACTCCGAGCTTCCAAAAACCCAA------
>HAER01073303_Proasellus_cantabricus_Glp
ATTGATGTTGTGGATTGGTTGCGCAAACACTATGCG---ATACAAAAACAAGTTGTTAAA
GAATTCTTAGCCGAATTTCTGGGGACATTTTTGCTTATCGTTTTCGGTAATGGAGCAGTT
GCCCAAAGCGTGTTGACAGCGGGG---------------TCAGTTAACAACTATTTTTCT
GTAATCTGGGGGTACTCCCTTGGTGTAGTGATGGGCGTTATGGTGTCTGGTAATGTCAGC
GGAGGTCACATCAACCCTGCAGTAACATTCGCTATGGCCCTTTGGGGAAAACTA---CCG
TGGTCAAAGGTTCCAGTCTACATGGTGGCTCAATACTTAGGGGCCTTTGCTGCTTCCGTT
TGTCTTTATGGAGTCTATACAGACGCTATTCATGCTTACGAA---CCGACT---------
CAT------------------------------ACTATGGCCACCGCTGGAATATGGGCT
ACTTATCCTGGAACGATG------ATCAACGCCAATGGAACGGAAGTTGAAAGCTATTTG
ACTGCTGGAAATGGATTTGGTGATCAGGTATTTGGAACCATGCTTCTACTGATTTGCGTC
TGCGCAATTAATGACCAAAGAAACATGAATGTCCCTCAATGGATTTCTCCTCTTTTCGTG
GGTTTCGTGGTGATGAACATTGGTCTCAGCTTTGGATTTAACTGTGGGTTCGCCCTCAAC
CCTGCAAGAGATTTGGCCCCTAGATTCTTCACTATGATTGCTGGCTGGGGAAGTGAACCA
TTCACAGCAACTACTGTCGATGGTGTGAGTTGGTGGTGGATCCCAGTGGTGGCCACTCAT
ATTGGAGCAATTCTAGGAATTTTGGTCTACACCATTTTC---ATCGAAATGCATCATAAG
GACAATAAGGACTCCGAGCTTCCAAAAGCCCAA------
>HAEX01018667_Proasellus_grafi_Glp
ATTGATGTTGTGGATTGGTTGCGCAAACACTATGCG---ATACAGAAACAAGTTGTTAAA
GAATTCTTAGCCGAATTTCTGGGGACATTTTTGCTAATCGTTTTCGGTAATGGAGCAGTT
GCCCAAAGCGTGTTAACAGCAGGG---------------TCAGTTAACAACTATTTTTCT
GTAATCTGGGGGTACTCCCTTGGGGTAGTGATGGGCGTTATGGTGTCTGGTAATGTCAGT
GGAGGTCACATCAACCCTGCAGTAACATTCGCTATGGCCCTTTGGGGAAAACTA---CCG
TGGTCAAAGGTTCCAGTCTACATGGTGGCCCAATACTTAGGGGCATTTGCCGCTTCCGTC
TGTCTCTATGGAGTCTATACAGACGCTATTCATGCTTACGAA---CCGACT---------
CAT------------------------------ACTATGGCCACCGCTGGAATATGGGCT
ACTTATCCTGGAACGATG------ATCAACGCCAACGGAACGGAAGTTGAAAGCTATTTG
ACTGCTGGAAATGGATTTGGTGATCAGGTATTTGGAACCATGCTTCTACTGATTTGTGTC
TGCGCAATTAATGACCAAAGAAACATGAATGTCCCTCAATGGATTTCTCCTCTTTTCGTG
GGTTTCGTGGTGATGAACATTGGTCTCAGCTTTGGATTTAACTGTGGGTTCGCCCTCAAC
CCTGCAAGAGATTTAGCCCCTAGATTCTTCACAATGATTGCTGGCTGGGGAAGCGATCCA
TTCACAGCAACAACTGTCGATGGTGTCAGTTGGTGGTGGATCCCAGTGGTGGCCCCTCAT
ATTGGAGCAATTCTAGGAATTTTGGTCTACACCATTTTC---ATCGAAATGCATCATAAG
GAAAATAAGGATTCCGAGCTTCCAAAAACCCAACAA---
>HAFH01036875_Proasellus_racovitzai_Glp
ATTGATGTTGTGGATTGGTTGCGCAAACACTATGCG---ATACAGAAGCAAGTTGTTAAA
GAATTCTTAGCCGAATTTCTGGGGACATTTTTGCTTGTCGTTTTCGGTAATGGAGCAGTT
GCCCAAAGCGTGTTGACAGCGGGG---------------TCAGTTAACAACTATTTTTCT
GTAATCTGGGGGTACTCCCTCGGGGTAGTGATGGGCATTATGGTGTCTGGCAATGTCAGC
GGAGGTCACATCAACCCTGCAGTAACATTCGCTATGGCTCTTTGGGGTAAATTA---CCC
TGGTCAAAAGTTCCAGTCTACATGGTGGCCCAGTACTTAGGGGCCTTTGCTGCTTCCGTC
TGTCTCTATGGAGTCTATACAGACGCTATTAACACTTACGAA---CCGACG---------
CAT------------------------------ACTATGGCCACAGCTGGAATTTGGGCT
ACTTATCCTGGAACGATG------ATCAACGCCAACGGAACGGAAGTTGAAAGTTATTTG
ACTGCTGGAAATGGATTTGGTGATCAGGTATTTGGAACCATGCTTCTACTGATTTGCGTC
TGCGCTATTAACGACCGAAGAAACATGAATGTCCCTCAATGGATTAGTCCTCTTTTCGTT
GGTTTCGTGGTCATGAACATTGGTCTCAGCTTTGGATTTAACTGCGGTTTCGCCCTCAAC
CCTGCAAGAGATTTGGCTCCTAGATTTTTCACTATGATTGCTGGCTGGGGCAGCGAACCA
TTCACAGCAACAACTGACGATGGTGTGAGTTGGTGGTGGATCCCAGTGGTGGCCCCTCAT
ATTGGAGCAATTCTAGGAATTTTGGTCTACTCCATTTTC---ATAGAAATGCATCATAAG

GACAATAAGGACTCCGAACTTCCCAAAGCCCAA------
>HAFL01016789_Proasellus_beticus_Glp
ATCGATGTTGTGGATTGGTTGCGTAAACACTACGCA---ATACAGAAACAAGTAGTCAAA
GAATTCTTAGCCGAATTTCTGGGAACATTTTTGCTTGTCGTTTTCGGTAATGGAGCAGTT
GCCCAAAACGTGTTGACAGCAGGG---------------TCAGCTAACGACTATTTTTCT
GTAATCTGGGGGTACTCAATCGGGGCTATGATGGGCGTTATGGTGTCTGGCAACGTCAGC
GGAGGCCACATCAACCCTGCAGTAACATTCGCTATGGCTCTATGGGGAAAATTA---CCC
TGGTCAAAGGTTCCAGTCTACATGGTGGCCCAATACTTAGGGGCCTTTGCTGCTTCCGTC
TGTCTCTATGGAGTATATACAGATGCTATTAACACTTTCGAA---CCGACA---------
CAT------------------------------ACCATGTCCACCGCTGGAATATTTGCT
ACTTATCCTGGTACGATG------GTTAACGCTACCGGAACGGAAGTCGAAAGCTTTTTG
ACTGCTGGAAATGGGTTTGGTGATCAGGTATTTGGAACGATGCTTCTACTGATCTGCGTC
TGCGCAATTAACGACCGAAGGAACATGAATATTCCTCAATGGAGTTGTCCTCTTTTCGTT
GGTTTCGTGGTCATGAACATTGGTCTCAGCTTTGGATTTAACTGTGGGTTTGCCATCAAC
CCTGCAAGGGATTTGGCCCCTAGATTTTTCACTATGATTGCTGGCTGGGGCAGTGAACCC
TTTACAGCAACTACTGACGATGGTGTGAGTTGGTGGTGGATCCCGTTGCTGGGCCCTCAT
GTTGGAGCAATTCTAGGAATTTTGGTCTACTCCATTTTC---ATCGAAATGCATCACAAG
GACAATAAGGACTCTGAGCTTCCAAAAGCCCAA------
>HAEP01031015_Proasellus_assaforensis_Glp
ATTAATGTTGTGGATTGGTTGCGCAAACACTATGCA---ATACAGAAACAAGTTGTCAAA
GAATTCTTAGCTGAATTTCTGGGGACATTTTTGCTTATCGTTTTCGGTAATGGAGCAGTT
GCCCAAAGCGTGTTGACAGCGGGG---------------TCAGTTAACGACTATTTTTCT
GTAATCTGGGGGTACTCCCTTGGGGTAATGATGGGCATTATGGTGTCTGGTAATGTCAGC
GGAGGTCACATCAATCCTGCAGTAACATTCGCTATGGCCCTTTGGGGAAAACTA---CCT
TGGTCAAAGGTTCCAGTCTACATGGTGGCACAATACTTAGGGGCCTTTGCTGCTTCTGTC
TGCCTCTATGGCGTCTACACAGACGCTATTAACACTTACGAA---CCGACC---------
CAT------------------------------ACTATGGCCACCGCTGGAATATGGGCT
ACTTATCCTGGAACGATG------ATCAATGCCAATGGAACGGAAGTTGAAAGCTATTTG
ACTGCTGGAAATGGATTTGGTGATCAGGTATTTGGAACCATGCTTCTACTAATCTGCGTC
TGCGCAATTAACGACCGAAGAAACATGAATGTCCCTCAATGGATTTGTCCCCTTTTCGTT
GGTTTCGTGGTCATGAACATTGGTCTAAGCTTTGGATTTAACTGTGGGTTCGCCCTCAAC
CCTGCAAGAGATTTGGCCCCAAGATTCTTCACTATGATTGCTGGCTGGGGCAGCGAACCA
TTCACAGCAACAACTGACGATGGTGTGAGTTGGTGGTGGATCCCAGTGGTGGCCCCTCAT
GTTGGAGCAATTCTAGGAATCTTGGTCTACACCATTTTC---ATCGAGATGCATCATAAG
GACAATAAGGACTCCGAGCTTCCAAAAGCCCAA------
>HAFG01098721_Proasellus_parvulus_Glp
ATAGACGTTGTAGAATGGTTACGAAAAAGATACGCT---ATAACGAACCAAATTGTCAAA
GAATTTTTAGCCGAATTTCTTGGAACATTTTTACTAATCGTTTTTGGTAACGGAGCAGTC
GCCCAAAGCGTGTTGACGGCGGGA---------------TCGGTTAACAACTATTTTTCT
GTAGTATGGGGGTACTCTCTAGGGGTAATGATGGGCGTTCTTGTGTCTGGTAATGTCAGC
GGAGGTCACATCAACCCTGCAGTAACATTCGCTATGGCCCTTTGGGGAAAACTG---CCG
TGGTCAAAAGTTCCAGTTTACATGGTAGCCCAATACTTAGGGGCCTTTGCGGCCTCAGTC
TGTTTATACGGAGTCTATACAGATGCTCTTCATGCTTACGAG---CCGACC---------
CAT------------------------------ACTATGGAATCCGCTGGAATATGGGCC
ACTTATCCTGGAACGATG------GTCAACGTCAACGGAACAGAAGTTGAAAGTTATTTG
ACTGCCGGGAATGGATTTGGAGATCAGGTATTTGGAACCATGCTGCTACTGATCTGTGTA
TGCGCCATTAATGACAGAAGGAACATGAATGTTCCTCAATGGATTTGTCCCCTTTTCGTT
GGTTTCGCAGTCATGAACATCGGTCTGAGCTTCGGATTTAACTGCGGGTTCGCCCTCAAT
CCTGCAAGAGATTTGGCACCTCGATTCTTCACTATGATTGCTGGCTGGGGGAGTGACCCA
TTCACGGCAACAACTTTCGATGGTGTGAGTTGGTGGTGGATTCCAGTGGTGGCCCCTCAC
CTTGGAGCAATTCTGGGAATTTTGGTCTACACCATTTTC---ATCGATATGCATCACAAG
GACACTAAAGAGTCTGAACTTCCAAAAGTTATT------
>HAEN01065588_Bragasellus_peltatus_Glp
ATTGATGTGGCGAATTATTTACGTGAAAAATACAGT---ATCAAAAACCCTGTCGCCAAA
GAATTTTTGGCCGAATTTCTTGGGACTTTCATCCTTATAGTTTTCGGCAATGGAGCAGTG
GCCCAGAGCGTCTTGACAGGGGGA---------------TCGGTAAACGACTACTTCTCC
GTGGTATGGGGGTATTCCCTCGGGGTAATGATGGGCATTTTGGTGTCTGGCAACGTCAGT
GGAGGGCACATCAATCCAGCAGTCACTTTTGCCATGGCCCTTTGGGGTAAATTG---CCA
TGGCCGAAGGTTCCAGTTTACATGACTGCTCAGTATTTAGGGGCCTTTTCTGCGTCGGCG
TGCCTATATGGAGTTTATACAGATGCAATTTCCACTTTCGAG---CCAACG---------
CAT------------------------------ACCTTGGCCACAGCCGGAATATGGGCA
ACATACCCTGGAACTATG------GTCAATGCCAATGGAACAGAAATTGAAAGTTACTTA
ACAGCTGGAAATGGATTTGGAGATCAGGTCTTCGGAACAATGCTTCTGTTGGTGTGCGTC
TGCGCCATTAACGATTCGAGAAACATGAAGGTTCCTCAGTGGATCTGTCCCCTGTTCGTC
GGTTTCGTCGTGATGAACATCGGTCTCAGTTTCGGATTCAATTGCGGTTTCGCCCTCAAC
CCAGCGAGGGATTTGGCACCCAGATTCTTCACTATGATTGCTGGATGGGGAAGCGACCCC
TTCACAGCAACGACGTATGACGGAGTCAGTTGGTGGTGGATACCAGTAGTGGCTCCCCAC
ATTGGGGCAGTCCTGGGGATGCTCGTGTACACCGTTTTC---GTCGACATGCACCACCAA

GACCCTACGGAGGTCGAGCTTCCAAAACCTCAA------
>SAUD01000707_Armadillidium_vulgare_Glp
ATAGATGTTACAAAAGCTTTAAGAAAAAAATTTCGC---TTAAAAAATGAAGCAGGCAGA
GAATTTTTAGCAGAATTTTTAGGAACATTTATACTAACCGTATTGGGTGATGGTTCAGTT
GCTCAAAAAACTCTAACTGGTGGA---------------ACATCAAATGATTTCTTCAGT
GTTATTTGGGGATGGGGTTTAGCAGTAACATTTGGGGTTATTGCCTCTGGAAATATAAGC
GGTGGACACATCAATCCTGCCGTAACTTTAGCTATGGCTCTTTGGGGAAGACTT---TCA
TGGCAGAAAGTTCCAATTTATATGGTGGCGCAGTATCTAGGCGCTTTTTCGGCTTCTGTT
TTTGTCTACGCAGTTTACACAGGCGCCCTCAATAGTTATGAA---ACAACG---------
CGA------------------------------TCTTTGAGTACAGCTGGAATATGGGCA
ACATATCCTGGAACGATG------GTTAATGCCAATGGAACAGTTGTCAATAATTTTCTT
ACAACTGGAAATGGAATTGGAGATCAAGTTTTTTCCACCATGATACTTCTACTCCTCATT
TGCGCAATAGTTGACCAGAAGAATATGCAAATCCCCAAATGGATTTCTCCACTATTTGTG
GGTTTTGCTGTTTTAAGCATCGGTATTTGCTACGGATTCAACTGCGGATTTGCCATCAAC
CCTGCACGAGATCTTGCCCCGAGGATTTTTACGCTCATCGCCGGATGGGGGGATGCACCT
TTTACGGCATCGACCGTGGAAGGAGTTGTGTGGTGGTGGGTGCCAATTATAGCTCCTCAT
ATCGGAGCTGTCCTCGGGATGCTCGTTTACACTCTCTTC---ATTGAAATGCATCACGAA
GATGATTCAGAAGTCATCACCATAACCAAAGAA------
>SEYY01000912_Armadillidium_nasatum_Glp
ATAGATGTTACAAAAGCTTTAAGAAAAAAATTTCGC---TTAAAAAATGAAGCAGGCAGG
GAATTTTTAGCAGAATTTTTAGGAACATTTATACTAATCGCATTGGGTGATGGTTCAGTT
GCTCAAAAAACTCTAACTGGTGGA---------------ACATCAAATGATTTCTTCAGT
GTTATTTGGGGATGGGGTTTAGCAGTAACATTTGGGGTTATTGCCTCTGGAAATATAAGC
GGTGGACACATCAATCCTGCCGTAACTTTAGCTATGGCCCTTTGGGGAAGACTT---TCA
TGGCAAAAGGTTCCAATTTATATGGTGGCGCAGTATCTAGGCGCTTTTTCGGCTTCTGTT
TTTGTCTACGCAGTTTACACAGGCGCCCTCAATAGTTATGAA---ACGACG---------
CGA------------------------------TCTTTGAGTACAGCTGGAATATGGGCA
ACATATCCTGGAACGATG------GTTAATGCCAATGGAACAGTTGTCAATAATTTTCTT
ACAACTGGAAATGGAATTGGAGATCAAGTTTTTTCCACCATGATACTTCTACTCCTCATT
TGCGCAATAGTTGACCAGAAGAATATGCAAATCCCCAAATGGATTTCTCCTCTATTTGTT
GGTTTTGCAGTTTTAAGCATCGGTATCTGCTACGGCTTCAACTGCGGATTTGCCATCAAC
CCTGCAAGAGATCTTGCCCCGAGGATTTTTACGCTCATAGCCGGATGGGGGGATGCACCT
TTTAAGGCATCGACCGTGGAAGGAGTGGTGTGGTGGTGGGTACCAATAATAGCTCCTCAT
ATCGGAGCTGTCCTCGGGATGCTCGTCTACACTCTCTTT---ATTGAAATGCATCACGAA
GACGATTCAGAAGTCATCACCATAACCAAAGAA------
>IABZ01085732_Ligia_exotica_Glp
GTAGATGTTTCTCTGACTCTTCGAAAGAAATACGGT---TTACAAAATGAATCTGCAAGA
GTTTTTATGGCAGAATTTTTAGGCACATTCATACTTATT---------AATGGTGCTGTT
GCGCAAAAAGTATTCACAGGGGAC---------------ACAGTTAACGATTTTTTCTCA
GTAGTATGGGGCTACTCCCTTGGAGTAATGATGGGGATTCTAGCATCTGGCAACATTAGT
GGAGGTCACATAAATCCAGCCGTTACTTTGGCGATGGCGCTGTGGGGCAGACTA---CCT
TGGCATAAAGTGCCTGTCTATATGGTCGCCCAATATTTGGGAGCGTTCGTAGGTTCGATC
GCCGTATTCGGAGTTTATCAGAGTGCTCTCAATAATTATGAG---ACAGTT---------
CCT------------------------------TCAGTTGCTACTGCAGGCATCTTTGCG
ACATATCCAGGCTCTATG------GTCAATACTAATGGAACTACAATTGAAAGTTTTCTC
ACTAGCGGAAATGGATTTGGTGACCAGATTCTAGGAACAATGTTGCTGCTGATCTGCGTC
TGCGCAATCACCGATCAAAAGAATATGAAAATACCTCAATGGCTCTCTCCCATTTTGGTA
GGATTCGCCGTAATGAATATCGGTCTCAGTTTCGGATTTAACTGTGGGTTCGCCATTAAT
CCTGCCAGAGATTTGGGTCCCAGGATTTTCACGCTTTTAGCTGGATGGGGAGAACTGCCC
TTTACAACGAGCACCGTAGATGGGACCGCGTGGTGGTGGATACCAGTTATCGCATCCCAC
ATTGGAGCAATCATGGGTATGTTAGTTTACACGCTATTG---ATTGAGATGCACCACCAA
GAGGAATCGGAA---CACTTACCGCAAATTACA------
>GGMN01017858_Idotea_baltica_Glp
TTGGACGTGGCTGCGGAAATGCGGCGAAGGTTCGGT---CTGAAGAGCAGCGCGGCAAAG
GAATTTCTCGCAGAATTCATCGGCACGTTTCTGCTTATCCTCTTCGGTGACGCCGCCGTG
GCCCAGAGTGTCCTCACAGGCAAC---------------ACGACCAACGACTACTTCTCG
GTCGTGTGGGGATACTCCCTCGGGGTGACGATGGGCATCTTGGCCTCCGGGGGCGTGAGT
GGGGGCCACCTCAACCCCGCCGTCACACTGGCCATGGCCCTTTGGGGCAGGCTC---GAG
TGGCCTAAGGTCCCCATCTACATGGTGGCCCAGTACCTCGGGGCCTTCACTGCTTCCGTT
GTCCTCTTCGGTGTCTACTCAAGTGCCCTGAGCAGCTTCGAG---ACTGTC---------
CCT------------------------------TCCCTGGCGACGGCGGGCATCTGGGCC
ACATACCCGGGCTCCATG------GTCAACGCCAATGGCACAGCCGTATCAAGCTTCCTC
ACCAGTGGAAATGGATTCGCTGATCAGGTGCTTGGAACGATGCTGCTGCTGGTGTGCGTG
TGCGCCATCTCGGACCAGAGGAACATGAAGGTCCCGCAGTGGCTGACGCCGCTCTTCGTC
GGCTTCGTCGTGATGAACATCGGCCTCAGCTTCGGATTCAACTGCGGATTCGCCATCAAC
CCCGCCAGGGACTTGGCGCCAAGGATTTTCACGCTCATCGCAGGATGGGGAGATCTCACT
TTCACAGCGAGCACCATCGACGGGGTGTCCTGGTGGTGGATCCCCATTCTGGCGCCCCAC
GTCGGGGCCGTGCTGGGGATGCTCGTCTACACCGTCTTC---GTCGAGATGCACCACGAC

GTGCCCTCCGAG---GACCTTCCGAAGGTGCAG------
>GFFH01028051_Oratosquilla_oratoria_Glp
AATCGCATCCAGGAGCTTCTGAGGAGTAAATTGGCC---ATTCGCAACCAAATGGTCAAA
GAGTTCTTGGCGGAGTTCTTGGGAACCTTCATTCTAATCGTCTTTGGCGATGGGAGCGTA
GCCCAGAGTGTTCTGACCAATGGA---------------CAGAAGGCGGACTTCTTCTCC
ATCAACTGGGGTTGGGGCATCGCTGTCACTCTTGGGGTTCTGGTGTCCGGCGGCATCTCT
GGAGGACACCTCAACCCCGCCGTGTCGCTGGCCATGGCCGTCTGGGGGAAGTTC---TCC
TGGGTGAAAGTTCCTGTGTATATGGTAGCCCAGTATCTGGGGAGTTTCACGGCCGCTCTC
GTCCTCCTGGGTGTTTACGCAGACGCATTGGACGCCTTCGACGGAGGCAAC---------
CGG------------------------------ACGATGGCCACGGCCGGCATCTTCGCC
ACTTACCCTGGAAGCATC------GTCGACGCCAATCAAGTCACAGTCGGTTCGTTCTTG
ACCAACGCTAACGGCGTCTCCGATCAGATCGTCGGCACCATGTTGCTCCTGATTTGTGTG
TGTGCCATCACGGACCCTCGTAACATGGAGGTCCCGAAGGCGCTCATCCCTCTGTTCGTG
GGTTTCGCCGTCCTCAATATTGGCATCTGCTTCGGATTCAATAGCGGCTACGCTATCAAC
CCGGCTCGAGACTTGGCTCCCAGGCTGGTGACTCTCATCGCGGGCTGGGGCGACGCAACA
TTTACGGCCGGCACCGTGGACGGCATCGCGTGGTGGTGGGTCCCCATCGTGGCGACGCAC
ATCGGGGGCATCCTCGGGGTCCTCGTGTACCTCTTCTTC---ATCGAACTGCACCACCCA
GACACTGAGGAGTACCAGCTGCCACAGGTCCAG------
>GCDB01023983_Nebalia_bipes_Glp
AATCGAATCATGAGCATGGTCCGTAGTAAGGTTGGA---ATAAAGAATGAATTCACTAGA
ATATTTCTCGCTGAGTTCCTGGGGACGTTTATACTAATGATGTTCGGCGACGGCTCGGTG
GCCCAGAGCGTGTTGTCGGGTGAG---------------GCGAAGGGAGGCTTCCTCTCC
GTCAACCTGGGCTGGGGGCTGGGAGTCACGATGGGC---CTAGTGTCGGGGGGCATATCC
GGGGGGCACATCAACCCCGCCGTCACGGTCGCCATGGCCTGCTTCGGCAAGCTG---CAG
TGGATCAAGGTGCCCATCTACATGGTCGCCCAGTACCTCGGAGCCTTCCTGGCCTCGGGG
GTCCTATTCGGGGTCTACAAGAATGCCCTGGACGCGTTCGAGGGCGGGAAC---------
CTG------------------------------ACTAGCGCCACGGCGGGCATCTTCGCG
ACATACCCCGGCACCATC------CAGAACGCGGAGGGG---GAG---ATGGCCTTCCTC
TATAATGGCAACGGCTTTGGCGACCAGATCGTGGGCACGATGATGCTGCTGATCTGCGTG
TGCGCCATCACGGACTCGCGCAACATGAAGGTGCCTAACGGTCTCGTTCCGCTCTTCGTC
GGCCTCGCCGTCGTCAACATCGGCATCTGCTTCGGCCTCAACTGTGGATACGCCATCAAC
CCTGCCAGGGACCTCGCGCCTAGACTCTTCACCCTCATTGCAGGATGGGGCGACTACCCC
TTCACAGCCACCACCCTGGACGATATCGCCTGGTGGTGGGTTCCCGTAGTGGCACCCCAC
GTGGGCGCCATCATGGGGGTGGCCATCTACCTGTTCCTG---GTCGAGCTGCACCACCCG
TCCCAGGAGGAGTTCAGCATGGGCCAGGTCCAC------
>KZS09273_Daphnia_magna_GlpB2
AACCAACGAGACGACAAATTGCCTTCTAATTGCAAT------ATGCCCAGTCTGGCTCGG
GCGGTTTTGGCAGAGTTTATTGGCACCTTTATTCTTGTCGCTATCGGCAGTGGCAGCGTA
GCTCAATCGCAATTGACGAACGGC---------------AAAAAAGGCGACTACTTCACC
ATCAATTGGGGGTGGGCTTTGGGGTGCTCGTTGGGAATCCTCGTTTCGGGCAAAGCATCA
GGTGGTCACTTGAATCCGGCCGTGACGATGGCCCTAGCCGTAGCCAAGGATTTC---TCC
TGGAAGAGGCTACCTGCGTATTGGTTAGCGCAGTACTCTGGAGCTCTGGTCGCCTCTGGC
GCTGTTCTAGGCATCTACTACGAAGCCATAGCTGTTAGAAAGGTTGACGGC---------
GAGTTTCGAATTAATTCCAATTCATCTGAACCGGAGCCCGGCTCGGCAGCCATTTTCGCC
AATTATCCG---------------------------------------ACCCCTTATTCG
TCTGTTCTTACCGGCTTAATGGAAGAGATTTTATCGACCATGATCTTCATGCTGATCATT
TGTGTGGCGACCAACTCCAAGTATTCCAAAGTGCCGACTTTCTTGCAACCGCTCTACATT
GGATTCACGTTGCTGGCCATCGGCGTGGCCTACGGATCCAACGGAGGCTACGCTCTTAAC
CCGGCTCGTGATTTGGCTCCCAGGTTAGTCACGTATTTCGGAGGATGGGGACCGCGCGTA
TTTAGCTTCCGC---------AGCTACAATTGGTTCTGGATTTTCCTGGTCGGGCCACAC
GTGGGAGCTCTTCTCGGCGTCGGCATTTTCCATGCCGTT---TTCTTGCGATTGGAGCAG
ACAGCGGTCGCAACGGATTCGCAGCTACAAATGGTGGAC
>CM023192_Daphnia_carinata_GlpB2
CAGCAAAGAGGCGACAAACTCACTCCGAATTGCAAC------TTCCCCAGTCTGGCTCGA
GCCGTGTTGGCGGAGTTCATCGGCACCTTCATCCTTGTCACTATCGGAAGTGGCAGCGTA
GCTCAATCGCAGCTAACCAATGGC---------------AAAAAAGGCGATTACTTCACC
ATCAACTGGGGATGGGCTCTAGGATGTTCGCTGGGCATCCTCGTTGCGTCCAAAGCCTCA
GGCGGCCACTTGAATCCGGCCGTGACGATGGCCCTGGCCGTGGCCAAAGATTTC---TCC
TGGAAGAGGCTGCCCGCCTATTGGTTAGCGCAGTACTCTGGAGCTCTGGTCGCTTCCGGC
ACCGTCCTCGGCATCTACTACGAAGCCATAGCTGACAAGAAAGTGGACGGT---------
GAGTTTCGAATCAATGCCAATTCGTCGGATCCGGAGCCCGGCTCGGCGGCCATTTTCGCC
AACTATCCG---------------------------------------ACGCCTTATTCG
TCTGTTGTTACCGGCTTAATGGAAGAGATTTTGTCGACCATGATCTTCATGCTGATCATT
TGCGTCGTGACCAATTCGAAGTATTCGAAAGTGCCGAATTTCTTGCAGCCGCTCTACATT
GGCTTCACGTTGCTGGCCATCGGCGTGGCCTACGGATCCAACGGAGGCTACGCTCTGAAC
CCGGCTCGGGATTTGGCCCCCCGATTAGTCTCTTACTTCGGAGGATGGGGACCGCGCGTA
TTCAGCTTTCGC---------GGCTACAACTGGTTCTGGATTTTCCTGGTCGGGCCACAC
GTGGGAGCTCTGCTCGGCGTCGGCATTTTCCACTCCGTC---TTCTTGCGATTGGAGCAG

TCAGCGGTCGGAACGGATTCGCAGCTGCCGATGGTCGAC
>CM023192_Daphnia_carinata_GlpB3P
---------------------CCTACGAATTGCAGT------TGTCCTTGCCTAACTCGC
GCCGTTTTGGCCGAGTTCATCGGTACGTTCATCCTTGTTGTTATT------GGCAGTGTA
GCTCAATCGCAACCAACCAATGGC---------------CAAAAAGGCGATTACTCTACC
ATCAACTGGGGATGGGCTCTAGGATGTTCGCTGGGAATCCTCGTT------------TCG
GGTGGCCATTTGAATACGGCATTGACGCTGGCCCTGGCCGTGGCCAAAGATTTT---CCC
TGGAAGAGGCTGCTTGCGTATTGGTTAGGCCAGTACCTGGGATCTTTGGCCGCTTCCGGG
---------------------------------------AAAATGAACGGT---------
GAATTT---ATTAACTCCAATTTATCTGAACCGGAGTTGGACTCGGCAGCCATTTTCGCC
AATTATACG---------------------------------------ACGCCCTATTCT
CACGTCGTTACTGGTTTAGTAGAAGAGATTCTATCAACAATGATCTTCATACTGATTATT
TGTGTGGTGACCAATTCCAAATATTCAAAAGTGCCGAGTTTTTTGCAGCCGCTCTACACC
GGTTTCACGCTGTTAGCCCTTGGCGCGGCGTACGGATCCAACGGAGGTTACGCTCTA---
---GCGCGTGATTTGGCTCCCCTGTTAGTCACGCATTTCGGAGGA---------------

>KZS11463_Daphnia_magna_GlpB1
---AGCAAAGGAGAC---------AAGAATTGTCGT---TTGTCCAGCACACTTGTTCGC
GCCGCCTTAGCCGAGTTTATTGGCACCTACATTCTTGTTGTCATTGGCAACGGTAGTGTT
GCCCAGTCGCAACTGACGAATGGC---------------AAACAAGGCGATTATTTCACC
ATCAACTGGGGATGGGCTTTGGGATGCTCGTTGGGAATCCTCATTTCGGCCGAGGCATCA
GGTGGTCACTTGAACCCGGCCGTGACGCTGGCCCTGGCCGTGGCTAAAGATTTC---CCA
TGGAAACGGCTACCCGTGTATTGGTTAGCCCAGTATTTTGGAGCGCTGGTTGCGTCTGGC
AGCGTCCTCGGCGTTTATTACGAAGCCATTCTCCTTGGAAAACGAGATGGA---------
AAGTTTCGAATTAACGCCAATTCGTCCGAACCGGAGCCTGGCTCAGCGGCCATTTTTGCA
AACTATCCG---------------------------------------ACTCCTTATTCG
TCAGTGGTTACCGGTCTAGTAGAAGAGACTTTATGTACAATGATCTTCATGCTGATTATA
TGCGTGGTGACGAATGCCAAATATTCAAAAGTGCCGAGCTTCTTGCAGCCGCTCTACATT
GGTTTCACGTTGTTGGCCATGGGCGTGGCTTACGGATCTAATGGAGGCTACGCGCTAAAT
CCGGCTCGCGATTTGGCTCCCCGATTAGTTACTTATTTCGGAGGATGGGGACCTCGTGTA
TTTAGCTTCCGC---------GGCTACAATTGGTTCTGGATTTTCCTAGTCGGGCCACAT
GTGGGTGCCCTTCTTGGCGTCGGCATTTTTCATGCCGTT---TTCTTTCTACCAGAACAG
AGAGTGGTTGCGACCGATTCACAGCTACCAATAGTGAAT
>CM023192_Daphnia_carinata_GlpB1
---AGCAAAGGAGAC------AATCGTAATTGTCGT---TTGTCCAGCGCACTCGTTCGC
GCCGCCTTAGCCGAGTTTATTGGCACGTACATCCTTGTTGTTATTGGCAATGGTAGTGTG
GCCCAGTCGCAACTGACGAATGGC---------------AAACAAGGCGACTACTTCACC
ATCAACTGGGGATGGGCTTTGGGATGCTCGTTGGGAATCCTCATTTCGGCCGAAGCATCA
GGTGGTCACTTGAACCCGGCCGTGACGCTGGCCCTGGCCGTGGCGAAAGATTTC---CCG
TGGAAAAGGCTACCCGTGTATTGGTTAGCGCAGTATTTTGGAGCGCTGGTGGCGTCTGGC
AGCGTCCTCGGCGTTTATTACGAAGCCATCCTCGTTGGAAAACGAGACGGA---------
AAGTTTCGAATTAACGCGAATTCGTCCGAACCGGAGCCAGGCTCTGCGGCCATATTTGCT
AATTATCCG---------------------------------------ACTCCTTATTCG
TCAGTGATTACTGGTTTAGTAGAAGAGACTTTATGTACAATGATCTTTATGCTGATTATC
TGCGTGGTGACCAATGCGAAATACTCAAAAGTGCCGAGCTTCTTGCAGCCGCTCTACATT
GGTTTCACGTTGTTGGCCATGGGCGTGGCCTACGGATCGAATGGAGGCTACGCGCTAAAT
CCCGCCCGCGATTTAGCTCCCCGATTAGTTACGTATTTCGGAGGATGGGGACCTCGTGTA
TTTAGCTTCCGC---------GGCTACAATTGGTTCTGGATTTTCTTAGTCGGGCCACAT
GTGGGGGCCCTTCTTGGCGTCGGTATTTTTCATACCGTT---TTCTTTCTACCAGAACAG
ACGGTGGTTGCGACCGATTCACAGCTACCAATTGTCAAT
>HAFN01005095_Daphnia_galeata_GlpA8
---------------AAAATGCGCGTGTGTTGTGCCAATGGTGTTCATCCTCTGGTTCGA
GCCGCTTTGGCCGAATTTATTGGCACCTTCATTCTTGTTGCCATCGGCAATGGCAGCGTA
GCCCAATCGCAATTGACCCAAGGT---------------GAAAAAGGCAATTATTTCACT
ATCAATTGGGGATGGTGTTTAGGATGTTGCCTCGGAATTCTCGTTTCAGCTAAAGCATCA
GGTGGCCATTTGAATCCGGCTGTAACAATGGCTTTCGCAGTGGCTAAAGATTTT---CCA
TGGAAAAGGCTTCCAGTGTATTGGTTTGCCCAGTACCTAGGAGCTCTAGCCGCGTCAGCC
ACTGTCCTTGGAGTTTACTACGAAGCTATTATCATTAGAAAAGTTGATGGC---------
CATTTCAGAATTCATGATAATGCTTCT------GAACCCGGGTCGGCGGCCATTTTTGCC
AATTATCCG---------------------------------------GCTCCTTATTCG
TCGGTTTGTACTGGTTTAGTCGAGGAGATTTTATGTACAACAATATTTATGATTGTTATC
TGTTCGGTTACGAATCCCAAGTATTCAAAAGTCCCTAGCTCCTTGCAGCCGCTCTACATT
GGTTTCACTCTTTTGGCTGTCGGAGTAGCTTACGGTTCCAACGGAGGATACGCTCTTAAC
CCGGCTCGTGATTTGGCTCCTCGACTAGTTTCTTACTTCGGTGGATTTGGATCACGCGTA
TTTAGTTTCCGT---------GGTTACAATTGGTTTTGGATCTTTTTGGTTGGACCGCAC
GTGGGAGCCATTTTAGGTGTTTTTATCTTTCACTTTGTT---CTTCTGGAAACGAATCAA

CCGGCGAAAGTAAATAATTCGCAGTTGCCAATCGTGGAT
>JAAVJA010000055_Daphnia_dubai_GlpA8

---------GCCGAATTTATTGGCACCTTCATCCTTGTCGTCATAGGTAATGGCAGCGTA
GCCCAATCGCAATTGACTCAAGGT---------------GAAAAAGGCAATTATTTCACG
ATTAACTGGGGGTGGTGTTTAGGTTGTTGTCTCGGAATTCTCGTTTCAGCTAAAGCATCA
GGTGGCCATTTAAATCCTGCTGTTACAATGGCTCTCGCAGTGGCAAAAGATTTT---CCG
TGGAAAAGGCTTCCAGTGTATTGGTTGGCGCAGTATCTAGGAGCCCTAGCTGCGTCTGGT
ACTGTTCTTGGCGTTTATTACGAAGGAATTATCGTCAGGAAAGTTGATGGT---------
CATTTCAGGATTCATGCCAATGCTTCT------GATCCCGGATCGGCGGCCATTTTTGCC
AATTATCCG---------------------------------------GCTCCTTATTCG
TCAGTTTGTACTGGTTTAATAGAAGAGATTTTATGTACAACAATATTTATGCTTGTTATA
TGCGCAGTAACGAATCCTAAATATTCAAAAGTCCCTAGTTCCTTGCAGCCAATCTACATT
GGATTCACGCTTTTGGCCGTCGGAGTAGCTTACGGATCTAACGGAGGATACGCTCTTAAC
CCGGCTCGTGATTTGGCTCCTCGTCTAGTTTCTTATATCGGTGGATTTGGTCCACGCGTC
TTCAGTTTCCGT---------GGCTACAATTGGTTTTGGATCTTTTTGGTTGGACCGCAC
GTGGGAGCCATTTTAGGTGTTTTCATCTTTCACTTCATA---CTCTTGGAAACGGACCAA
CCAGCAAAGGACAATAATTCCCAATTGCCAATCGTGGAG
>EFX66203_Daphnia_pulex_GlpA8
------------------ATGCGTCTGTGCTGTCGT------GTTCATCCCCTGGTTCGA
GCCGCTTTTGCCGAATTTATTGGCACCTTCATTCTTGTTGCCATCGGAAATGGAAGCGTA
GCCCAATCACAATTGACCCAAGGC---------------GAAAGGGCAATTATTTCACT
ATCAACTGGGGATGGTGTATTGGATGTTGCCTTGGAATTCTCGTTTCAGCTAAAGCATCA
GGTGGCCATCTGAATCCAGCAGTAACAATGGCTCTCGCAGTGGCAAAAGATTTC---CCG
TGGAAAAGGCTTCCAGTGTACTGGTTTGCACAGTACTTGGGAGCTCTGGCTGCGTCTGGT
ACTGTCCTTGGAGTTTATTACGAAGCAATTATCGTTAGAAAAATTGATGGC---------
AAATTCAGGATTCATGCCAATGCTTCT------GAGCCCGGATCGGCTGCCATTTTTGCC
AATTATCCG---------------------------------------GCCCCTTATTCG
TCGGTTGTTACCGGTTTAGTCGAGGAGATTTTATGTACAACGATATTTATGCTGGTTATA
TGCGTGGTAACAAATCCAAAGTATTCAAAAGTCCCCAGTTTTTTGCAGCCGTTCTACATT
GGTTTCACCCTTTTGGCCGTCGGTGTGGCTTACGGATCTAATGGAGGATACGCTCTTAAC
CCGGCTCGTGATTTAGCTCCTCGACTTGTTTCTTATTTCGGTGGATTTGGATCACGCGTT
TTCAGTTTCCGT---------GGATACAATTGGTTTTGGATCTTTTTGGTTGGGCCGCAT
GTTGGAGCCATTCTGGGTGTTTTTATCTTTCATTTTATT---CTCATGGAAACGGACAAA
CCAGCGAGCGTAAATAGTTCGCAGTTGCCAATCGTGGAT
>KZS03555_Daphnia_magna_GlpA8
------------------ATGCGATCTAGGTGTACC------ATTCCTCCCTTGGTTCGA
GCCGCCGTTGCCGAATTTATTGGCACCTTCGTCCTCGTAACTATTGGCAGCGGTAGTGTA
GCTCAATCGCAATTGACCGACGGT---------------GCAAAGGGAAACTACTTCACC
ATCAACTGGGGATGGGCTATAGGATGTTCTTTAGGAATCCTCGTATCGGCTAAAGCATCA
GGTGGCCACTTAAATCCGGCTGTGACAATGGCTCTTGCAGTGGGCAAAGATTTC---CCG
TGGAAAAGACTACCCGTTTATTGGTGTGCCCAATACTTGGGGGCTCTGGCCGCGTCCGGG
ACAGTCCTAGGCGTTTATTACGAAGCCATAGCCATGCACAAGGTGGATGGT---------
CAATTCCGCATAAACGCAAATGCTTCC------GAGCCAGGCTCAGCCGCCATTTTCGCC
AACTATCCA---------------------------------------GCTGCTTATTCC
TCGGTTATCACTGGCCTTATCGAAGAGGTTTTTTGTACGACGATATTCATGCTTGTCATT
TGCGTCGTGACCAATCCGAAACACTTGAAAGTACCAAGTTATTTGCAGCCAATTTACATT
GGTTTCACCTTGTTGGCAGTTGGCGTAGCTTATGGATCTAATGGAGGCTACGCTTTGAAC
CCGGCTCGTGATTTGGCTCCACGATTGGTTTCTTATCTTGGTGGATGGGGAAAACGAGTG
TTCAGCTTCCGT---------GGCTACAATTGGTTTTGGATTTTTCTTGTTGGACCCCAT
GTCGGAGCCATTATTGGTGTCGGTATTTTCCACTTTCTG---ATCTTGGAAGAAGACCAG
ACTCCGGAAACAGAAAATTCACAGGCGCCAATCATCGAA
>CM023188_Daphnia_carinata_GlpA8
------------------ATGCGATCGAGGTGTACC------ATTCCTCCTCTGGCTCGA
GCCGCCGTTGCCGAATTTATTGGCACCTTCGTTCTCGTTACGATTGGCAGTGGTAGTGTA
GCTCAATCGCAATTGACCGACGGT---------------GCAAAGGGTAATTACTTCACC
ATCAACTGGGGATGGGCCATAGGATGTTCCTTAGGAATCCTCGTATCGGCTAAAGCATCA
GGTGGCCACTTAAATCCGGCTGTGACAATGGCTCTTGCAGTGGGCAAAGATTTC---CCG
TGGAAAAGATTACCTGTGTATTGGTGTGCCCAATACTTGGGATCTCTGGCCGCATCCGGG
ACAGTCCTAGGAGTTTATTACGAGGCCATAGCCATGTACAAAATCGACGGC---------
CAATTCCGCATTAATGCAAATGCTTCC------GAGCCAGGCTCAGCCGCCATTTTCGCC
AACTATCCA---------------------------------------GCTGCTTATTCC
TCCGTTATCACCGGCCTCATCGAAGAGATTTTGTGTACCACGATATTCATGATTGTTATC
TGCGTCGTGACTAATCCGAAACACCTGAAAGTACCCAGTTACTTGCAGCCACTTTACATC
GGTTTCACCTTGTTGGCAGTTGGCGTTGCTTATGGATCTAATGGAGGCTACGCTTTGAAC
CCGGCTCGTGATTTGGCTCCACGGTTGGTTTCGTACCTTGGTGGATGGGGAACACGAGTG
TTTAGCTTCCGC---------AACTACAATTGGTTTTGGATTTTTCTTGTCGGACCACAT
GTGGGAGCCATTCTTGGTGTTGGTATTTTTCATTTCCTC---ATCTTGGAAAAAGACCAG

ATCCCGGAAACAAACAGTTCACAAGCGCCAATTCTCGAA
>KZS03556_Daphnia_magna_GlpA9
TCGACATTGTCGTCCCAGACTATGTTATCCTGTAGTGGGACTGATCCCTCCCTCACTCGG
GCGGCGTTTGCAGAATTCATAGGGACCTTCGTTCTCGTTGCTATAGGCAATGGCAGTGTG
GCTCAGTCGCAGTTAACGAATGGC---------------GAAAAGGGCAACTACTTCACC
ATCAACTGGGGATGGGCTTTAGGATGTTCCTTGGGCATTCTCATATCCGCTAAAACATCA
GGGGGTCACATAAATCCGGCCGTGACGATGGCTCTGGCGGTAGCCAAAGAATTT---CCA
TGGAAACGGCTACCTGCATATTGGTGTGCACAGTATTTGGGAGCTCTCGCAGCGTCTAGC
ACGGTTCTAGGAGTTTATTACGAGGCCATCATGAACGGCAAAGTCAACGGG---------
GAGTTCAAAATCCAAAAGGATTCTTCC------GATCCGGGCTCAGCGGCTATTTTTGCT
AATTATCCA---------------------------------------GCTCCATATTCA
TCAGCTAGCACTGGCCTAATGGAAGAGATTTTGTCCACTGTGGTCTTCATGTTGGTTATC
CGTGTGGTAACCGAT---AGGCAATCAAAAGTACCCAGTTTTCTGCAACCGCTTTACATT
GGGTTCACTTTACTGGCAATCGGCGTGTCCTATGGATCGAACGGCGGCTATGCTCTGAAT
CCGGCTAGAGATTTAGCTCCTCGAGTTGTTACACTTTTCGCTGGATGGGGAAGCCATGTT
TTCAGCTTCCGT---------GGATACAATTGGTTCTGGATTTATCTGCTGGGTCCTCAC
GTTGGAGCCATTCTAGGCATAGGCATCTTCAATCTCTTC---CTGAAGTCGGATGGAACA
ATGGACGAC---ACAAGATCAGAATCTCCTTGTATCGAT
>CM023188_Daphnia_carinata_GlpA9
TCGACATTGTCGGCC---GAGATGCTGTCTTGCAGTCGGAATGATCCTTCCCTGACAAGG
GCGGCTTTTGCGGAATTCATTGGAACCTTCGTTCTCGTCGCTATCGGCAATGGAAGTGTT
GCCCAGTCACTATTAACTAATGGC---------------GAAAAGGGCAACTATTTCACC
ATCAACTGGGGATGGGGTTTAGGATGTTTCTTAGGCATTCTCGTATCCGCTAAAACATCA
GGGGGTCACATAAATCCGGCCGTGACGATGGCTCTGGCAGTGACGAAAGAGTTC---CCA
TGGAACCGGCTACCGGCATATTGGTGTGCACAATATCTGGGAGCTCTTGCAGCATCTAGC
ACAGTTCTGTCTGTGTATTACGAGGCCATCATGAATGGTAAAGTGAACGGT---------
GAATTCAAAATCCACAAGGATCCTTCC------GATCCGGGCTCAGCGGCTATTTTTGCT
AATTATCCA---------------------------------------GCTCCATATTCA
TCAGCTAGTGCTGGCCTGATGGAAGAGATTTTGTCCACTGTCATCTTCATGTTGGTTATC
CGTGTGGTAACTGAT---AGGCAATCAAAAGTACCCAGTTTTCAGCAACCGCTTTACATC
GGTTTCACTTTATTGGCAATCGGCTTGTCTTACGGATCAAACGGCGGCTATGCACTGAAT
CCGGCTAGAGATATTGCTCCTCGAGTTGTTACACTTTTTGCTGGATGGGGAATCCATGTT
TTCAGCATCCGT---------GGATACAATTGGTTCTGGATTTATCTGGTGGGTCCGCAC
GTTGGAGCCATCCTAGGCGTTGTCATCTTCCATCTCTTC---GTAAGACAAGATGGGACA
ATGGACGAC---ACAAGATCAGAATCTCCTTCAATCGAT
>JAAVJA010002980_Daphnia_dubai_GlpA7
---------------------TGTCTC---TGCAGC------GTCCCACAAATAGTTCGT
GCAGCA---GCCGAATTTATTGGCACCTTCGTCTTCGTCGTCATCGGAAATGGAAGCATT
GCTCAGTCCCATTTGACTAAAGGG---------------AAAAAATTNGATTACTTCACG
ATTAACTGGGGTTGGGCTTTAGGATGTTGTCTTGGAACCCTAATTTCAGCTAAAATATCA
GGCGGCCACTTGAACCCGGCGGTGACGATGGCTCTCTCTCTCGTCCGCGATTTT---CCT
TGGAAAAGGTTGCCGGTTTATTGGTTC---------------------------------
------------------TCAGAAGCGCTAGTTCAAGGCAAACTTAACGGT---------
GAGTTC--

---------------------------ATTTTATCTACGATGATTTTCATGATAGTCATT
TGCGTGACCACGAATGCAAAATATTCGAATGTTCCGTCTTTCTTGCAGCCGATCTACATT
GGTTTCACCTTACTGGCCTGTGGCATATCTTACGGCNNNNNNNGAGGTTACGCTCTCAAC
CCGGCACGCGATTTATCTCTTCGTCTTGTTTTTTATCTCGCTGGATGGGGCGAAGGCGCC
TTC--GGACCGCAC
ATTGGAGCTTTTTTGGGAGTATATATCTTTCACATCTTT---TTGAGGACTGAGCTGAAT
ACGGATTGGGAAAAACCTTCACCGGCAGCACCAACAGTC
>JAAVJA010002980_Daphnia_dubai_GlpA6
------------------ATGTGTAAAAAGTTTCAA------GTTCCATCGTTGTTTAGA
GCAGCATTCGCTGAATTTATTGGTACCTACATCTTGGTTGTCATCGGGAACGGGAGCATA
GCGCAGTCACAGTTAACCAATGGC---------------GAAAAAGGNGATTACTTCACG
ATTAACTGGGGATGGGCTTTAGGATGTTCGTTGGGAATTCTCATTTCTGCTAATATATCA
GGAGGTCATTTGAATCCAGCAGTGACACTGGCGCTGGCACTAGTTCGTCATTTC---CCG
TGGAAAAAGTTACCTGTTTATTGGTGTGCCCAATATCTCGGAGCGATGGCAGCTTCTGGA
ACCGTCCTTGGAGTCTATTACGAGGGAATACTGGAAGGAAAAGTCGAAGGT---------
GAATTTCGGATCAGGAAGAACATTACC------GAACCGGGAATGGCATCTATTTTTGCA
AACTATCCA---------------------------------------GCTCCATATTCA
TCAGCTAGTATTGGACTGGTTGAGGAAATTTTATCGACTATGATCTTCATGCTGGTTATA
TGCGTTGTGACTGACAAAAGGTATTCAAACGTN---------------------TACATA
GGCTTCGCCCTGTTGGCCATTGGTGTTGCATACGGA------------------------
---GCTCGTGATTTAGCTCCTAGGTTGGTCTCTTTTCTGGGTGGATGGGGACCAGGTGTA
TTTAGTTTTCGG---------GATTACAATTGGTTCTGGATATTTTTGGTCGGTCCTCAC
GTTGGAGCCGTTCTGGGCGTTTCCATTTTTCATCTGGTT---TTGAAAAAGGGCTCAGCA

GATAGCAGCGAGACAGCCTCTTCAATTGATTCACGTCAA
>HAFN01017572_Daphnia_galeata_GlpA6

------------------------TGTGCTCAGTATTTGGGAGCGATGGCCGCTTCTGGA
ACTGTTCTTGGAGTTTATTATGAGGGAATCTTGGAAGGAAAAGTCGGCGGT---------
GAATTTCGAATCAGGAAGAACATTACC------GAACCGGGTATGGCATCTATTTTTGCC
AACTATCCA--GCCCCATATTCG
TCGGCTAGCATTGGACTTGTTGAGGAGATTTTATCGACTATGATATTCATGCTCGTTATA
TGCGTTGTGACTGACAAAAGGTATTCATGCGTGCCGAATTTCTTGCAACCACTCTACATC
GGCTTTGCCCTGTTGGCCATCGGCGTTGCGTACGGATCGAATGGAGGTTACGCTCTCAAC
CCGGCTCGTGATTTGGCTCCTCGGTTGGTCTCATTTCTTGCCGGATGGGGACCAGGTGTA
TTTAGTTTTCGG---------GATTACAATTGGTTCTGGATATTTTTGGTCGGCCCGCAT
GTTGGAGCCATTTTGGGCGTTTGCATTTTTCACTTGATT---CTTAAAAAAGGTTCAGCA
GAAACCAGCGAGACAAGCTCATCTATGGATTCACGCCAA
>EFX79826_Daphnia_pulex_GlpA6
------------------ATGTGTAAAAGATTTCGT------GTCCCGTCTTTGTTTCGA
GCAGCATTCGCTGAATTCATTGGCACCTACATCTTGGTTGTCATTGGGAACGGGAGCATA
GCGCAATCACAGTTAACCAATGGC---------------GAAAAAGGTGATTACTTCACC
ATTAACTGGGGATGGGCTTTAGGGTGTTCGCTGGGAATTCTTATTTCAGCTAATATATCA
GGGGGCCATTTGAATCCAGCCGTGACACTGGCCCTTGCCCTAGTTCGTCATTTC---CCG
TGGAAAAAGTTGCCGGTGTATTGGTGTGCCCAGTATCTAGGAGCGATGGCCGCTTCTGGG
ACCGTCCTTGGAGTCTATCACGAA------------GGAAAAGTCGACGGG---------
GAATTTCGGATCAGAAAGAACATTACC------GAACCCGGCATGGCTTCTATTTTCGCC
AATTACCCA--GCCCCATATTCA
TCGGCTAGCATTGGACTAGTCGAAGAGATCTTATCGACCATGATCTTCATGCTCGTTATC
TGCGTGGTGACAGACAAAAGGTATTCGAACGTGCCGAATTTCTTGCAGCCACTCTACATC
GGCTTCACCTTGTTGGCCATCGGGGTCGCGTACGGATCGAATGGAGGTTACGCTCTCAAC
CCGGCTCGTGATTTGGCTCCCCGTTTGGTCACCTTTCTTGGTGGGTGGGGGCTAGGTGTA
TTTAGTTTCCGG---------GGCTACAATTGGTTCTGGATCTTTCTGGTGGGACCTCAC
GTCGGAGCACTCTTGGGAGTAGGAGTCTTTCACATCTTT---CTAAAGACTGAGCTCACG
GACTGGGCAGAAAAACGTCCAGAAAAGCCAATCGGTGCC
>CM023188_Daphnia_carinata_GlpA6
------------------ATGTGTAAGAGATTCCAC------GTTCCTTCTGTATTTCGA
GCAGCATTCGCCGAATTTTTAGGCACCTATATTTTGGTAGTCATTGGGAACGGAAGTATA
GCTCAGTCGCAGTTGACCAACGGC---------------GAAAAAGGGGACTATTTTACT
ATCAACTGGGGATGGGCTTTGGGTTGTTCTTTAGGAATGCTCGTGTCAGCCAATGTATCA
GGAGGTCATCTGAATCCCGCGGTGACAATGGCGCTAGCATTAGTTCGTCGTTTT---CCA
TGGAAAAGGCTGCCAGTGTATTGGTGTGCCCAATACATGGGAGCTCTTGCCGCTTCTGGA
ACGGTTCTCGGAGTATATCACGAAGGCATTTTGGAAGGAAAAGTCAATGGC---------
AAATTCCGCATTGAGAAGAACATTACA------GAACCTGGTATGGCCTCCATTTTTGCT
AACTATCCA--GCACATTACTCG
TCGGCTAGTATTGGTTTAATCGAAGAAATTTTATCGACCATGATCTTCACGCTCGTCATA
TGCGTGGTAACCGACAAACGGTATTCAAACGTGCCAAACTTCTTGCAGCCACTCTATGTC
GGATTCACGTTGTTGGCCATCGGTGTCGCCTACGGATCCAATGGAGGTTACGCCCTCAAT
CCGGCGCGGGATTTGGCTCCCCGGTTGGTTTCCTTCCTTGGTGGATGGGGACTAGGTGTA
TTCAGTTTTCGA---------GACTACAACTGGTTCTGGATATTCTTGGTCGGCCCGCAT
GTTGGAGCCATTCTTGGTGTCTGTATCTTCCACCTGATC---TTGAAGAATGGCTCGAAA
GATATCAGTGAGGACGTTTCCATGATCAATCCACGTCTA
>HAFN01012924_Daphnia_galeata_GlpA1
------------------ATGCGTTCGAGGTTTTCT------ATTCCACTTATAGTGCGC
ATAGCTTTCGCCGAGTTCGTCGGTACTTACGTTTTGGTTGTCATTGGCAACGGAAGTGTA
GCGCAGGCCCAACTGACTCATGGT---------------CAGAAAGGTGACTATTTCGCA
ATCAATTGGGGTTGGGCACTCGGTTGTTTTCTTGGCGTGTTGATTTCGGCTAACGTATCA
GGCGGCCACTTAAATCCAGCAGTCACCCTGGCCCTGGCCATCACTCGCAGGTTC---GAA
TGGAAAAAACTCCCCGTTTACTGGTTTGCGCAATACCTGGGAGCTCTGACTGCTTCTGGA
ACGGTTCTAGGGGTTTATTATGAGGCAATTATGCAAGGCAAAGTAAATGGC---------
GAATTCCGTATTCATAAGGATTCTTCT------GATCCAGGTTCGGCTTCAATTTTTGCA
AATTATCCA--GCTCCTTACTCG
TCGGCTGGCCTTTGTTTGGTCGAAGAGATATTAGCCACGATGGTATTTATGTTGGTCATA
TGCGTTACAACGGATAAAAGATATTCGAAAATACCTCCGTTCTTGCAGCCCTTATTTATT
GGCTTTGCCTTACTGGCTCTTGGCATTTCCTATGGAGCTAACAGTGGCTACGCTTTGAAT
CCGGCTCGCGATCTTGGTCCTCGCATAACATCTTACTTTGGAGGATGGGGAACTGGAGTT
TTCAGCTTTAGG---------GACTATAACTGGTTTTGGATCTTTATTTTGGGGCCACAC
ATCGGTGCCATTTTGGGAGTTTGTATCTTCCAACTTCTC---TTGAAGCTGCATCAATCC

AATGATCTAGATCATGATCAGCAACTGCAACGGAAGGAA
>JAAVJA010009739_Daphnia_dubai_GlpA1
------------------ATGCGTCCGAGGTTTTCT------ATTCCGCTTATGGTACGC
ACAGCTTTCGCTGAGTTCGTCGGCACATATGTTTTGGTTGTTATTGGCAACGGAAGTGTA
GCGCAAGCCCAATTGACTCACGGT---------------CAGAAAGGTGACTAT------

GGTGGCCACTTAAATCCAGCAGTTACCCTGGCCCTGGCCATAACCCGTAGGTTC---GAA
TGGAAAAAACTACCCGTTTACTGGCTCGCGCAATACCTAGGAGCTCTGGCGGCGTCTGGA
ACGGTTTTAGGGGTTTATTACGAGGCAATCATG---GGCAAAGTAAATGGC---------
GAATTCCGTATTCATAAGGATTCTTCT------GACCCAGGATCGGCATCAATTTTTGCA
AATTATCCG---------------------------------------GCTCCTTACTCG
TCGGCTGGTCTCTGTTTGGTCGAAGAGATTTTGGCTACCATGATTTTCATGTTAGTCATT
TGCGCAACAACGGATAAAAGATATTCGAAAATACCTGTTTTCTTGCAGCCCTTATTTATT
GGATTTGCTTTACTGGCTATTGGAATTTCCTATGGAGCTAACAGCGGCTACGCTTTGAAT
CCAGCTCGCGATCTTGGTCCTCGTATAACATCTTACTTTGGAGGATGGGGAACTGGAGTT
TTTAGCTTCAGG---------GACTATAACTGGTTTTGGATCTTTATTGTGGGCCACAT
ATCGGTGCCATTTTGGGAGTTTGTGTCTTTCAACTTCTC---TTGAAGTTGCATAACTCC
AATGATCTAGATCACGATCATCAAATGCAACGGAAGGAA
>EFX88619_Daphnia_pulex_GlpA1
------------------ATGCGTCCGAGGTTTTCT------ATTCCACTCATGGTGCGC
ACAGCTTTCGCTGAGTTCGTCGGAACATACGTTTTGGTTGTTATTGGCAACGGAAGTGTA
GCGCAAGCCCAATTGACTCACGGC---------------CAGAAAGGTGACTATTTCGCG
ATCAATTGGGGTTGGGCTCTCGGCTGTGTTCTCGGCATTTTAATTTCGGCCAATGTATCA
GGTGGCCACTTAAATCCAGCAGTTACACTGGCCCTGGCCATTACTCGTAGATTC---GAA
TGGAAAAAACTACCCGTTTACTGGCTCGCGCAATATCTAGGAGCTCTGACTGCGTCTGGA
ACGGTTTTAGGGGTTTATTACGAGGCAATCATGCAAGGGAAAGTGAATGGC---------
GAATTCCGCATTCATAAAGACTCGTCT------GACCCAGGATCGGCTTCAATTTTTGCA
AATTATCCG---------------------------------------GCTCCTTACTCG
TCGGCTGGTCTCTGTTTGGTCGAAGAGATATTAGCTACCATGATTTTCATGCTGGTCATT
TGCGTAACAACGGATAAAAGATATTCGAAAATACCTGCCTTCTTGCAGCCCCTATTTATT
GGTTTCGCGTTACTTGCTATTGGAATTTCATATGGAGCAAACAGCGGCTACGCTTTGAAT
CCGGCTCGAGATCTGGGTCCTCGCATAACATCGTACTTAGGTGGATGGGGGACGGGAGTT
TTCAGCTTCAGG---------GACTACAACTGGTTTTGGATCTTTATTGTGGGCCACAC
ATCGGTGCCATTTTGGGAGTTTGCATCTTTCAACTTCTC---TTGAAGCTCCATCAATCC
AATGCTCTAGATCATGATCAGCAAATTCAACGTAAGGAA
>KZS21285_Daphnia_magna_GlpA1
------------------ATGCGTCCGAGGTTTTGC------ATTCCACTTATGGTACGC
GCTGCTTTTGCAGAGTTTGTTGGGACCTACGTTTTAGTTGTTATTGGCAACGGAAGTGTA
GCGCAAGCTCAGTTAACTCATGGC---------------CAGAAAGGAGACTATTTTGCC
ATCAATTGGGGCTGGGCTTTAGGCTGTATGTTAGGTATCCTGGTATCGGCCAACGTATCA
GGTGGTCACTTAAATCCGGCAGTAACGCTGGCCCTGGCCATCACTCGTAAGTTT---CAA
TGGAAGAAACTTCCCATCTACTGGTTTGCGCAATATCTGGGAGCTCTAGCTGCGTCTGGA
ACAGTTCTAGGCGTTTATTACGAGGCAATAATGGAAGGCAGAGTAAATGGA---------
GAATTTCGCATTCAGAAAAATGCGGCT------GACCCAGGAACGGCGGCAATATTTGCT
AATTATCCA---------------------------------------GCTCCTTATTCG
TCATCGGGTCTCTGTTTAGTTGAAGAGATTTTGGCTACCATGATTTTCATGTTAGTCATT
TGTGCAACAACAGACAAACGATACTCTAAGGTTCCTGCTTTCCTGCAACCTTTCTATATC
GGCTTCGCCTTGTTGGCCATGGGATTATCCTATGGGGCTAACAGTGGTTACGCTTTGAAC
CCGGCCCGCGATTTGGGTCCTCGCATAACATCGTACTTTGGCGGATGGGGCTCTGGAGTC
TTCAGTTTCAGA---------GATTATAACGGGTTTTGGATTTTCATCGCCGGACCCCAT
ATCGGAGCCATTTTAGGAGTCTGCATCTTTGAGCTTCTC---TTAAAGCTCCGCCCTTCC
GACAAAACAGATCATGAACAACAAATACAGCGAAAGGAA
>CM023188_Daphnia_carinata_GlpA1
------------------ATGCGTCCGAGGTTTTGC------ATTCCACTTATGGTACGC
GCTGCTTTTGCAGAGTTTGTTGGGACCTACGTTTTAGTTGTTATCGGCAACGGAAGTGTA
GCGCAAGCTCAGCTTACTCATGGT---------------CAGAAAGGAGACTATTTCGCC
GTCAACTGGGGCTGGGCTTTAGGCTGTATGTTAGGCATCCTGGTATCGGCCAACGTATCA
GGCGGCCACTTAAATCCGGCAGTAACGCTGGCCCTGGCAATCACACGTAAGTTT---CAA
TGGAAGAAACTTCCCATCTACTGGTTAGCGCAATATCTAGGAGCTCTGGCCGCATCTGGA
ACAGTCCTCGGCGTATATTACGAGGCAATAATGGAAGGCAGAGTGAATGGA---------
GAATTTCGCATTCAGAAAAATGCGGCA------GACCCAGGTACGGCGGCAATTTTCGCT
AATTATCCA---------------------------------------GCTCCTTATTCG
TCGTCGGGTCTCTGTTTGGTTGAAGAGATTTTAGCTACGATGATTTTCATGTTAGTCATT
TGTGCGACAACCGACAAACGATACTCTAAGGTTCCCACTTTCCTGCAACCTTTCTATATC
GGCTTCGCCTTGTTGTCCATGGGATTGTCCTATGGGGCCAACAGTGGGTACGCTTTGAAT
CCGGCTCGCGATTTGGGTCCTCGCATTACATCGTACTTTGGCGGATGGGGCTCTGGAGTC
TTCAGTTTCAGA---------GACTATAACGGGTTTTGGATTTTCATCGCCGGACCCCAT
ATCGGTGCTATTTTAGGAGTTTGCATCTTTGAACTTCTT---TTAAAGCTCCGCCCATCC

GACAAAGCAGATCATGAACAACAAATGCAGCGGAAGGAA
>GGQP01091236_Diaphanosoma_celebensis_Glp2
------------------TGTCGCCCGAACTGGTCTCTAACTCCGCCCCTTCTCCTGAAG
CAGGCCTTTGCTGAATTCGTTGGCACTTTCATCCTAGTGCTGATTGGCAATGGCAGCATC
GCCCAGTCTCTGCTGTCCAACGAA---------------GAGAAAGGCGACTTCTTCTCC
ATCAATTGGGGCTGGGCCATGGGAGTCACTCTCGGTGTCCTGATATCCGCCAACATCTCA
GGTGGCCATTTAAATCCAGCCATCACGTTGGCATTAAGTTTGAAGCGTCAATTT---CCC
TGGAAGAGACTGCTCGTCTACTGGGCGGCACAATATCTGGGCGCTCTGGTCGCTTCTGGA
ACAGTCTTTGGACTCTATCGAGAGGCAATATATGGAGGAAAACCCCATCAC---------
AAATTTCGCATCAAAGTGAATCCCAAC------GACCCGGGAACGGCGGTCATTTTTGCC
AACTATCCG---------------------------------------GAACCTTACACG
TCCACAGGAGAAGCTCTAGCCGAAGAGATTGTGGCGACGATGGTGCTCATGCTGGCCATT
TGCGTCGTGACCGATCCTCGATATTCCAAAGTTCCTGCTTACCTGCAGCCATTTTATGCC
GGATTCGCCATGCTGTCCATCGGACTCGCTTACGGAGCCAACTGCGGCTACGCACTGAAT
CCGGCCCGTGATTTAGCTCCGCGACTCGTCTCTCTCATCGGCGGATATGGCGTCGGTGTT
TTCAGTTTCCGA---------GGCTACAACTGGTTCTGGGTGTTCATCGTCGGTCCGCAC
ATTGGCGCCGTTTTGGGCTACATAATCTTCCGGCTGCTG---TTAAAGTCCGTCCCTTCC
GATCCGGCT---CAGTCAACCCAGAAACCAAATAACCCG
>JAAVJA010004895_Daphnia_dubai_GlpA5
ACATCAGGTCTGAAGTTATTGAAACGATCCTCCACTATATATCATCGACTTTATGTTCGA
GCTTTTTTAGCCGAATTTATTAGTACGTTTGCTGTTTTGGCTTTTGTGAATGGTTTGATC
GCTCAGGCCGTCCTTACGGACGGA---------------GAGAAGGGAAGTGGAGTCGCT
CCGAAGTGGGGTGCGTTTGTTGCCGCCATGTCAGGTATCTTAATCGCTTGTGGAGCATCA
GGTGCGCATTCTAATCCGGCGTTCTCTCTCCTTTTTGCCCTGAGAGGTATTCTA---CCG
TGGGCTAAATTGCCGGTTTACTGGTTAGCACAATACCTTGGGGCTTTTTGCGGAGCAGCA
GCCGTTCTTGGAGTCTATTGGGACGCGATAATGTTCAAAGGG---AATGGC---------
GAATTTTACATCACTAACGAA------------GATCCAGGGTTAGCTGCCATTTTCGCT
ACATATCCC---------------------------------------ATGCCTTACGTT
TCGATCACAGGCTGTATTATAGATCAG---TTTGGTAGTGCCTTACTTGCTCTCTGTGTG
GCCGCTTTAACCGATCCCAAAAACATGCGCATTCCACTTCCGCTAGTGGCATTGTATATT
GGATTTCTGGTGCTAGAATTGGCGATTGGATTTTCACTTAATACCGGTGGATCACCGAAT
CCTGCTCGAGATTTATCTCCGAGGATTTTAACTTATTTTGCTGGATGGGGAATAAGGGTC
TTTAGCTTCCGA---------GACTGGACCTGGTTTTGGATTCCAGTCGTGATCCCGCAT
TTTGGTGTGGTGTTTGGTTATTTAATTTATTTTATTCTT---ATTGAAGCGCATTGGCCA
ACACAA---------------------------------
>EFX88760_Daphnia_pulex_GlpA5
ACATCAGCTCTGAAGTTATTGAAAAGTTCCTCCGCTATTTATCATCGGCTTTATGTTCGA
GCTTTTTTAGCCGAATTTATAAGCACATTTGCTATTTTGGCGTTTGTGAATGGCTTAATC
GCCCAGGCTGTCCTTACGGACGGA---------------GAGAAGGGAAGCGGAGTCGCC
CCGAAATGGGGTGCGTTCGTTGCCGCCATGTCTGGTATCTTAATCGCTTGCGGTGCTTCA
GGCGCTCACTCTAATCCGGCATTCTCTCTTCTATTCGCTCTGAGAGGTATTTTA---CCC
TGGGCTAAATTGCCGGTTTACTGGATAGCACAATATCTTGGTGCTTTTTGCGGAGCAGCA
GCTGTTCTTGGAGTTTATTGGGACGCCATAATGTTTAAAGGC---AATGGC---------
GAATTTTACATCACAAACGAA------------GATCCGGGATTGGCTGCCATTTTTGCT
ACATATCCT---------------------------------------ATGCCATACGTC
TCCATCGCAGGATGTATTATAGATCAGCTTTTGGAAGTGCCTTACTTGCACTCTGTGTG
GCCGCTTTGACCGATCCCAAGAACATGCGTATTCCGCTTCCGCTCGTGGCCCTGTATATT
GGATTTTTGGTTTTGGAATTGGCTATTGGATTCTCACTTAATACAGGTGGCTCTCCGAAT
CCTGCTCGCGATTTATCTCCTAGGATTTTGACTTACTTTGCTGGATGGGGAATCCAGGTC
TTT------AGA---------GACTGGACCTGGTTTTGGATTCCCGTAGTAATACCTCAC
ATTGGTGTCGTAATCGGTTATTTAATATATTTTATTCTC---ATTGAAGCGCATTGGCCA
ACACAA---------------------------------
>HAFN01024804_Daphnia_galeata_GlpA4
---GTCGATAAGCGTCGCGTGAGAAGGGCCATCAGCCTGTCTTCCTACCCCCTTATCCGC
GAATTCCTTGGAGAACTGGCCGGGACCTTCGTTCTAGTGTTATGCATTATTGGAACAAAG
GCTCAATCAGTTCTGACCAACGGG---------------AAACTTGCAGAGTTCTTGGCT
GGCAGATTTGGCGATGGATTCGGTGTAATGGCCGGAGTTTGGGTTGCTGGCGGTGTGTCA
GGTGCTCATCTCAACCCTGCAATGTCGTTCGCTCTGGCCTGTATTGGAAGATTT---CAA
TGGCGGAAATTACCGGTTTATTGGACTGCGCAGTATATTGGCGCTTTTGCAGCTTCATCT
TGCGTTTTCGCAGTTTACCTCGAGGCGATTTATTACTTTACT---GGGGGG---------
CAATTTGTGATGACTGAAGAT------------GATCCTGGATTCGCTGCCGTTTTCGCT
CCATATCCT---------------------------------------TCTGCGTACCTT
TCAACGACGGGAGCCATAATCGATTCAGTAGTAGGATCAGCGTTGCTATTACTATGTGCC
TGCGCTATAGTCGACCAACAAAACTGCAGAGTCCCACTCTCTCTCTTTGCCTTATATATG
GGATTCCTCATAATGGCATTGGGCATAAGTTTCAGTATGAACTGCGGTGTACCCGTAAAC
CCGGCTCGAGATTTATCCCCAAGGTTGTTCACTTACCTAGTCGGATGGGGTCCTCAAGTA
TTTAGCTACGAC---------AACTGGATGTGGTTCTGGATCCCTCTTGTGATTCCGCAC
TTCGGCGCAACTCTTGGGGCTTTCATCTACATTATTTTC---ATCGAAGCTCACTGGCCG

AAA-----------------------------------
>JAAVJA010004895_Daphnia_dubai_GlpA4
---GTCGATAAGCGTCGCGTGAGAAGGGCCATCAGCCTGTCTTCCTACCCCCTTATCCGC
GAATTCCTTGGAGAACTGGCCGGGACCTTCGTTCTAGTGTTATGCATTATTGGAACAAAG
GCTCAAGCTGTTCTGACCAACGGG---------------AAACTGGCAGAGTTCCTGGCT
AGCAGATTTGGTGATGGATTCGGTGTCATGGCCGGAGTTTGGGTTGCAGGCGGTGTGTCA
GGTGCTCATTTAAACCCTGCAATGACGTTCGCTCTGGCCTGTGTTGGAAAATGT---CAA
TGGCGTAAATTACCCATCTACTGGACTGCCCAGTATATTGGCGCTTTTGCAGCTTCTTCT
TGCGTCTTCGCTGTTTACCTCGAGGCGATTTACTACTTCACT---GGGGGC---------
CAATTTGACATGACTGAAGAT------------GATCCTGGATTCGCTGCCGTTTTCGCT
CCCTATCCT---------------------------------------TCCGCCTACCTC
TCAACGGCAGGAGCCATTGTCGATTCAATAGTAGGATCAGCGTTGTTATTGCTATGTGCC
TGCGCTATAGTCGATCAACAAAACTGTAAAGTCCCACTCTCTCTCTTTGCCTTGTATATG
GGATTCCTCATAATGGGATTGGGCATAAGTTTCAGTATGAACTGCGGTGTACCCGTAAAC
CCGGCTCGAGATTTATCCCCAAGACTGTTCACTTACTTAGTCGGATGGGGTCCTCAAGTA
TTTAGCTACGAC---------AATTGGATGTGGTTCTGGATCCCTCTTGTGATTCCACAC
TTTGGTGCAACCCTTGGTGCCTTCATTTACATCATTTTC---ATCGAAGCTCACTGGCCG
AAA-----------------------------------
>EFX88758_Daphnia_pulex_GlpA4
---GTCGATAAGCGTCGCGTGAGAAGGGCCATCAGCCTGGCTTCCTACCCCCTTATCCGG
GAATTTCTTGGAGAGCTGGCCGGGACCTTCGTTCTAGTGTTATGCATTATCGGAACAAAG
GCACAAACGGTGTTGACCAACGGG---------------AAACTGGCAGAATTCATGGCC
GGCAGATTCGGCGATGGATTCGGTGTCATGGCCGGTGTTTGGGTTGCAGGCGGTGTGTCA
GGTGCCCATCTGAACCCAGCAATGTCGTTCGGTCTGGCCTGTGTTGGAAGACTT---GAA
TGGCGAAAGTTACCAGTCTATTGGGCTGCTCAGTATATCGGCGCTTTTGCAGCTGCATCT
TGCGTCTTTGCAGTTTACCTCGAGGCGATTTATTATCAAACT---GGGGGC---------
CAATTTGTGATGACAGAAGAT------------GATCCAGGATTCGCTGCCATTTTCGCT
CCTTATCCT---------------------------------------TCTGCCTACCTC
TCAACAGCAGGAGCCATTGTCGATTCGATAGTAGGATCGGCCCTGTTGTTGCTGTGTGCC
TGCGCTATAGTCGATCGACTAAATTGCAAAGTTCCACTTTCTGTCTTTGCCTTGTACATG
GGATTTCTCATCATGGGATTGGGCATCAGTTTCAGTATGAACTGCGGTGTGCCCGTCAAT
CCGGCTCGAGATTTATCTCCAAGACTGTTCACTTACTTAGTCGGATGGGGTCCACAAGTA
TTTAGCTACGAC---------AATTGGATGTGGTTCTGGATCCCTCTTGTGATTCCGCAC
TTTGGTGCAACCCTTGGAGCCTTCATTTACATCATTTTT---ATCGAAGCTCACTGGCCG
GAA-----------------------------------
>KZS15526_Daphnia_magna_GlpA4
---GTCGATAAGCGCCGCGTGCGGAAGGCCATCAGCCTGTCGGCCTACCCTCTTATCCGC
GAATTCTTAGGAGAGCTTGCCGGGACCTTCGTTCTAGTGTTATGCATTATCGGAACGCAG
GCTCAAGCCGTTTTGACCAACGGG---------------AGACTAGCAGAGTTCTTGGCT
AACCGGATGGGTGAAGGATTTGGTGTCATGGCCGGAGTTTGGATTGCTGGCGGCGTGTCA
GGTGCCCATTTGAACCCAGCCCTGTCGTTTGCCTTGGCCTGCACTGGAAAATTG---CAA
TGGCGCAAATTACCGGTTTATTGGATAGCCCAATATCTCGGGGCTTTTGCGGCTGCGGCT
TGTGTCTTTGCAGTTTATCTCGAGGCAATATTTTATCGCACC---GGAGGC---------
CAGTTTGTCATGACCGACGAA------------GACCCGGGATTCGCTGCCATTTTCGCT
CCTTACCCG---------------------------------------TCTGCCTATCTT
TCAATGACGGGCGCTATTGTTGATTCAATAGTTGGATCAGCATTGTTGCTGATATGTGCC
TCCGCCATAGTTGACCAAAGGAATTGCAAAGTCCCTGTTTCTCTTGTTGCCTTTTACATG
GGATTCGCCGTTATGGGATTGGGCATAAGCTTCAGCATGAACTGTGGGGTACCAATCAAT
CCTGCTAGAGATTTATCACCAAGACTTTTCACTTACCTGGTTGGTTGGGGTCAACAAGTA
TTCAGTTACAAC---------AACTGGATGTGGTTTTGGATCCCTCTTGTGATCCCTCAC
TTCGGTGCTGTGCTTGGAGCTTTCATCTATATCGTTTTT---GTTGATGCCCACTGGCCC
GCA-----------------------------------
>CM023188_Daphnia_carinata_GlpA4
---GTCGATAAACGCCGCGTGCGGAAGGCCATTAGCCTGTCGGCCTACCCTCTTATTCGC
GAATTCTTAGGAGAGCTTGCCGGGACCTTCGTTCTAGTGTTATGCATCATCGGAACGCAG
GCTCAAGCCGTTTTGACCAACGGG---------------AGACTAGCAGAGTTCTTGGCT
AACCGGATGGGCGAAGGATTTGGTGTCATGGCCGGAGTCTGGATTGCTGGCGGAGTATCA
GGTGCCCATTTGAACCCAGCCCTGTCATTTGCCTTGGCCTGCACCGGAAAATGT---CAA
TGGCGCAAATTACCGGTTTATTGGATAGCCCAGTATCTCGGGGCTTTCGCAGCTGCAGCT
TGTGTCTTTGCAGTTTATCTCGAGGCAATTTTCTATCGCACC---GGAGGC---------
CAGTTTGTCATGACCGACGAA------------GACCCAGGATTCGCTGCCATTTTCGCT
CCTTACCCG---------------------------------------TCTGACTATCTT
TCAATGACGGGCGCTATTGTCGATTCGATAGTTGGATCAGCATTGTTGCTGATATGTGCC
TCCGCCATAGTTGACCAAAGGAATTGCAAAGTCCCTGTTTCTCTTCTTGCCTTTTACATG
GGATTCGCCGTTATGGGATTGGGCATAAGCTTTAGCATGAACTGTGGCGTACCAATCAAT
CCTGCTAGAGATTTTTCACCAAGGCTTTTCACTTACCTGGTTGGGTGGGGCCAACAAGTA
TTCAGTTACAAC---------AACTGGATGTGGTTTTGGATCCCTCTTGTTATCCCTCAC
TTTGGTGCTTTGCTTGGAGCTTTCATCTATATCGTTTTT---ATTGATGCCCACTGGCCC

GCA------------------------------------
>CM023188_Daphnia_carinata_GlpA3
---GTCGATAAACGCTGCGTGCGGAAGGCCATCAGCCTGTCCGCCTACCCCCTTATCCGC
GAATTCCTGGGAGAGCTTTCCGGAACCTTCGTTCTAGTGTTATGCACCATAGGAACGCAG
GCTCAGGCTGTTCTGACCAAAAGG---------------AAACTGGCAGAGTTCTTGGCT
AACAGGATGGGCGATGGAATAGGTGTTATGGCAGGAATTTGGGTTGCAGGCGGCGTGTCA
GGTGCCCATATGAACCCAGCAATGACGTTTGCTTTGGCCTGCACTGGAAAGGTT---CCA
TGGCGTAAAGTACCGGCTTATTGGGCAGCTCAGTATCTTGGCGCTTTTGCAGCTACATCT
TGTGTTTTCGGAGTTTATTATGATGCAATTTACGCTCAAACT---GGGGGC---------
CAGTTTATTATGACCGATGAA------------GATCCGGGATTCGCTGCCATTTTTGCG
CCCTACCCA---------------------------------------TCTGCCTATCTT
TCAACGGCGGGGGCCGTGGTCGATTCAATAATTGGATCAACATTATTGCTGATATGTGCC
TCCGCCATAGTTGACCAAAGGAATTGCAAAGTTCCAGTTCCTTTTATTGCCTTCTACATG
GGATTTATTGTTATAGGGTTAGGCATAAGCCATAGCATGAACTGCGGCCTACCAATCAAT
CCTGCTCGAGATTTTTCAGCAAGGCTCTTCACGTATCTAGTCGGATGGGGCCCTCAAGTA
TTCAGTTACGAC---------AACTGGATGTGGTTTTGGATACCGCTTGTGATCCCTCAT
TTCGGAGCATTCCTTGGAGCTTTCATATACATAATTTTT---GTTAGTTCCCACTGGCCT
GTAGTA---------------------------------
>JAAVJA010004895_Daphnia_dubai_GlpA2
---------GCCCCCAAACTTATGAAGATGATACGT---GTCGAGGCGCCACTTCTCCGC
GAGATTATGGCAGAATTCCTGGGCACTTTCATTCTTGTGCTTTTTGGTGACGCCAGTGTG
GCCCAGTCTAAACTATCCAACGAA---------------GCCAATGGAGATTTCTTCTCC
ATCAACTGGGGTTGGGCTGTTGGTGTCATGATGGGGGTCTTGGTTGCTGGGGGCGTATCG
GGTGCCCATTTAAATCCGGCTGTCACGTTGGCCATGGCTTGTGCTGGTCGTATT---GCA
CTCATCAAAGTTTTCTTCTACTGGCTCGCACAGTATTTGGGAGCATTCGCCGCTGCTGCC
TCCGTCCTCGGAGTTTACAGCGATGCAATCCAATATGTTAGC---AACGGG---------
ACTTTGACTATGAACAATGATTTGAAC------GATCCGGGCTTGGCTGGTATCTTTGCT
ACTTATCCG---------------------------------------GCCCCTTGGCTC
ACCATTGCTGGAGGGCTTGGCGACCAGGTG---------ATGCTATTACTCCTCTGCATT
TGCGCCATCACGGACAAGAAAAACACGCAAATTCCGTCCGCTCTAGTGCCAATGTACGTC
GGATTCACCATTCTGGGCATCGGCGTTTGCTTTGGAGCCAACTGCGGCTACGCA------

---AGTTGGAAT---------AATTTCAACTGGTTCTGGATACCCATCGTTGGTCCTCAC
ATCGGAGCTATCCTCGGGGTCTTTATTTACATCGCCTTT---GTCGAAGCCCATTGGCCA
GAGGATGGCGACGAAGTGGTGCCAATGACC---GTT---
>HAFN01014029_Daphnia_galeata_GlpA2
---------GCTCCCAAACTTATGAAAATGATACGT---GTCGAGGCGCCACTTCTCCGC
GAGATTATGGCTGAATTCCTGGGCACTTTCATTCTTGTGCTTTTTGGTGACGCCAGTGTA
GCCCAGTCTAAATTATCCAACGAG---------------GCCAATGGAGACTTCTTCTCC
ATCAACTGGGGCTGGGCCGTCGGTGTTATGATGGGGGTCTTGGTTGCTGGGGGCGTATCG
GGTGCCCATTTAAATCCGGCTGTCACGCTTGCAATGGCTTGTGCTGGTCGTATT---GCA
CTCATCAAAGTTTTTTTCTACTGGCTCGCCCAATATTTGGGCGCATTCGCCGCTGCTGCC
TCCGTCCTCGGAGTCTATAGCGATGCAATCCAATATGCCAGC---AACGGG---------
ACTTTGACTATGAACAATGAATTGAAC------GATCCGGGCTTGGCTGGCATCTTTGCT
ACTTATCCG---------------------------------------GCCCATTGGCTC
TCCGTTGCTGGAGGGCTCGGCGACCAGATTTTGGGAACCATGCTGTTACTCCTCTGCATT
TGCGCCATCACGGACAAGAAAAACACGCAAATTCCGTCGGCTCTAGTGCCAATGTACGTC
GGATTCACCATTCTGGGTATCGGCGTTTGCTTTGGGGCCAACTGTGGCTACGCACTTAAC
CCAGCAAGAGATCTTTCACCTCGTCTCATCAGTCTCATCGCTGGATGGGAC---CAATCT
TTCAGCTGGAAC---------AATTACAACTGGTTCTGGATACCTATCGTTGGTCCTCAT
ATTGGAGCTATCCTCGGGGTCTTTATTTACATCGCATTT---GTCGAAGCCCACTGGCCG
GAGGATGGCGACGAAGTGGTGCCAATGACC---GTT---
>CM023188_Daphnia_carinata_GlpA2
---------GCCCCAAAGCTTATGAAGATGATACGT---GTCGAGGCTCCACTCCTTCGC
GAGATCATGGCTGAATTCCTGGGCACTTTTATTCTAGTGCTGTTTGGCGACGCCAGTGTA
GCCCAATCGAAACTATCAAATGAA---------------GCCAATGGTGACTTCTTCTCC
ATAAACTGGGGCTGGGCAATTGGTGTCATGATGGGAGTCTTGGTTGCTGGGGGCGTCTCG
GGTGCCCATTTGAATCCTGCTGTCACGTTGGCAATGGCTTGTGCTGGTCGCCTA---GCA
ATCATCAAAGTTTTCTTTTACATGCTGGCCCAGTATTTGGGAGCGTTCGCTGCCGCCGCC
TCCGTCCTTGGCGTCTACAGCGATGCGATCCAGTATGCAAGC---AACGGG---------
ACTTTGACTATGGACGATGAGGCAAAC------GATCCAGGACTGGCTGGCATTTTTGCC
ACTTATCCA---------------------------------------GCCCCATGGCTC
TCAGTTGCTGGAGGGCTTGGCGACCAGATATTGGGCACCATGTTGTTATTGCTCTGCATC
TGCGCCATTACGGACAAAAAGAACACGCAAATCCCGTCCGCTTTAGTGCCCATGTACGTA
GGATTCACCATTCTGGGCATCGGTGTTTGCTTTGGTGCTAATTGCGGCTATGCGCTCAAT
CCGGCCAGAGATCTTTCTCCCCGACTCATCAGCCTCATCGCCGGATGGGAA---CAATCT
TTCAGCTGGGAC---------AACTACAACTGGTTCTGGATTCCTATTGTTGGCCCTCAT
ATCGGAGCTATCCTTGGAGTCTTTATTTACATCGCATTC---GTTGAAGCTCACTGGCCT

GAGGATGGAGATGAACTGGTACCGGTGAAC---GTG---
>KZS15525_Daphnia_magna_GlpA2
---------GCCCCAAAGCTTATGAAGATGATACGT---GTCGAGGCTCCACTCCTTCGC
GAGATCATGGCTGAATTCCTGGGCACTTTTATTCTAGTGCTGTTTGGCGACGCCAGTGTA
GCCCAATCGAAACTATCAAACGAA---------------GCCAATGGTGACTTCTTCTCC
ATAAACTGGGGTTGGGCAATTGGTGTCATGATGGGAGTCTTGGTTGCTGGGGGCGTCTCG
GGTGCCCATTTGAATCCTGCTGTCACGTTGGCAATGGCTTGTGCTGGTCGTCTA---GCA
ATCATCAAAGTCTTCTTTTACATGCTGGCGCAATATTTGGGCGCGTTCGCTGCAGCCGCG
TCCGTCCTTGGCGTCTACAGCGATGCGATCCAGTATGCAAGC---AACGGG---------
ACTTTGACTATGAACGATGAAGCAAAC------GATCCGGGACTGGCTGGCATTTTCGCT
ACTTATCCA---------------------------------------GCCCCATGGCTC
TCGGTTGCTGGAGGGCTTGGCGACCAGATATTGGGCACCATGTTGTTATTGCTCTGCATC
TGCGCCATTACGGACAAGAAGAACACGCAAATCCCGTCCGCTTTAGTGCCCATGTACGTG
GGATTCACCATTCTAGGCATCGGTGTTTGCTTTGGTGCTAATTGCGGCTATGCGCTCAAT
CCGGCCAGAGATCTTTCCCCACGACTCATCAGTCTCATCGCCGGATGGGAA---CAGTCT
TTCAGCTGGGAC---------AACTACAACTGGTTCTGGATTCCTATTGTTGGCCCTCAT
ATCGGAGCTATTCTCGGAGTCTTTATTTACATCGCATTC---GTTGAAGCGCACTGGCCA
GAGGATGGAGATGAACTGGTACCCGTGGGC---GTG---
>EFX88757_Daphnia_pulex_GlpA2
---------GCCCCAAAACTTATGAAAATGATACGA---GTCGAGGCGCCACTTCTCCGT
GAGATTATGGCGGAATTCCTGGGCACTTTCATTCTTGTGCTTTTTGGTGACGCCAGCGTC
GCCCAGTCTAAACTTTCAAACGAA---------------GCCAATGGAGACTTTTTCTCC
ATCAACTGGGGCTGGGCTGTCGGTGTCATGATGGGGGTCTTGGTTGCTGGGGGCGTATCG
GGTGCTCATTTAAATCCGGCCGTCACGTTGGCAATGGCCTGCGCTGGTCGTATT---GCC
ATCATCAAAGTTTTCTTCTACATGCTGGCCCAGTATCTGGGCGCCTTCGCCGCAGCCGCC
TCCGTCCTCGGAGTCTACAGCGATGCCATCGAATATTACAGCAACAACGGG---------
ACTTTGTACATGAACAATGAAGCCAAC------GATCCGGGCTTGGCCGGCATTTTTTCC
ACCTATCCG---------------------------------------GCCCCTTGGCTC
TCCATTGCTGGAGGGATTGGCGACCAGATTTTGGGAACCATGCTGCTACTCCTCTGCATC
TGCGCCATCACGGACAAGAAAAACACGCAAATTCCGTCGGCCCTAGTGCCAATGTACGTG
GGATTCACCATTCTGGGCATCGGCGTTTGCTTCGGGGCCAACTGCGGCTACGCGCTCAAT
CCGGCCAGAGATCTTTCGCCCCGTCTCATCACTCTCATCGCCGGATGGGAC---CAATCT
TTCAGCTGGAAC---------AATTACAACTGGTTCTGGATACCCATCGTCGGCCCACAT
ATCGGAGCTATCCTCGGAGTCTTCATTTACATCGCCTTT---GTCGAAGCCCATTGGCCG
GAGGAAGGCGACGAAGTGGTGCCCATCACC---GTT---
>GGQP01066089_Diaphanosoma_celebensis_Glp1
---------TCGCCCAAGTTCATGAGCAAGATCCGC---ATCGAGGCGCCGCTGCTCCGC
GAGATCCTCGCCGAGTTCCTCGCCACTTTCATCCTCGTCCTGTTTGGAGATGCCAGCGTT
GCTCAGTCGGTGCTCTCCGGCGAG---------------AAGAACGGCGACTTCTTCTCC
ATCAACTGGGGCTGGGGTCTGGGCGTCATGATGGGCGTCCTGATCGCCGGCCGCGTGTCC
GGTGCTCACCTGAACCCAGCCGTGACGCTGGCCATGGCCATTTTCGGCCGCTTT---CCC
TGGATCAAGGTCCTCTTCTACTGGGCGGCTCAATACCTGGGCGCGTTGGCCGCCGCTGCC
TCCGTACTCGGCGTCTACAGCGAGGCCATTCACAACAAGACG---GGCGGA---------
TCGTTCACGATGCTGCCGGGT------------GATCCGGGCACGGCCGGAATTTTCGCC
ACCTACCCG---------------------------------------GCTCCATATCTG
TCGACCCTCGGCGGCTTGGGCGATCAGATCCTGGGAACGTTCCTGCTGCTCCTGTGCGTG
TGCGCCATTACCGACAAGAAGAACGGCAACGTGTCGCCGGCCCAGGCGCCGATCTACGTC
GGCATGACCATCATCGCCATCGGCGCCTGTTTCGGCGTCAACTGCGGCTACGCCATCAAC
CCGGCGCGCGATCTAGCGCCGCGTGTCTTGACCTGGTTTGCCGGCTGGGGCACCGACGTC
TTCACCTACGGA---------AAC---TACTGGTTCTGGGTACCGATCGTTGGTCCGCAC
ATCGGAGCCGTGCTCGGCGTTGCCGTTTACATTCTGTTC---ATCGAAGCCCACTGGCCC
GACGATGGCGAAGACGCTTACGACGCTTCCAGAATC---
>GEHY01004014_Triops_newberryi_Glp
---------------GAGCTTTTCCAGCGTTACCAT---ATAAAACACCCCTTAATCCGG
GAAATGTTAGCGGAATTCCTGGGTACCTTTTTACTTGTGCTATTCGGTGATGCCAGTGTA
GCTCAGTCGAAGCTCACTAACAAC---------------GCGAACGGTGATTTCTTTTCG
ATTAACTGGGGATGGGGTTTGGGAGTAATGATGGGAATTCTGGTAGCCGGTAACATCTCA
GGTGCACATTTGAATCCTGCCGTTTCACTTGCACTTGCCATGTTCAACAAGTTC---TCC
TGGATGAAATTACTGCCGTATATTTTCGCTCAGTACTTGGGGGCATTAGCGGCTGCTGCT
TGCGTTTTGGGAGTCTATTCAGAAGCCATATGGGACTTCTCG---AATGGT---------
AACCTAACGGTTGACGGT---------------AGTACTGGCACAGCGGGCATCTTCTCC
ACGTACCCT---------------------------------------GCCGAATACGTT
TCAGCAGCAGGAGGTTTCGGCGATCAGGTTATGGGAACGATGTTGCTCCTGCTTTGCATT
TGTGCCATTGTTGATAAGAAGAACGCGAACATCCCGAGCTCGCTGGTGCCATTGTACATC
GGTTTCGTGATATTAGCTATCGGAGTTTGCTTCGGCTCCAACTGTGGTTACGCTATTAAC
CCTGCCAGAGATTTGGCCCCGCGGTTCTTGACGTATATCGGTGGATGGGGTACTGGCGTT
TTCAGTTACCGG---------AATTACAACTGGTTCTGGGTTCCTATTATTGGCCCACAT
ATTGGCGCCATTTTGGGGGTACTCATCTACACCGTATGC---ATCGAATTACACTGGCCG

GAAACTTTCGAACAAGCAGTTGACGATAAACGCACC---
>BAYF01005257_Triops_cancriformis_Glp
---------------GAACTCTTCCAGCGTTACCAT---ATAAGAAATGCCTTGATCCGG
GAAATGTTAGCGGAATTCCTGGGTACATTTTTACTTGTGCTATTCGGAGATGCCAGTGTG
GCGCAGTCGAAGCTCACCAACAAC---------------GTGAACGGCGATTTCTTTTCA
ATTAATTGGGGATGGGGTTTGGGAGTAATGATGGGGATTTTGGTAGCTGGTAACATCTCA
GGTGCACATTTGAATCCTGCCGTTTCCCTCGCACTTGCAATGTTCAACAAGTTC---TCT
TGGATGAAATTACTACCGTATATTTTTGCTCAATACTTGGGGGCGTTGGCAGCTGCCGCA
TGCGTTTTAGGAGTTTATTCAGAAGCCATATGGGAGTTTTCG---AACGGT---------
AACCTAACGGTTGATGGT---------------AGTACTGGCACAGCGGGCATATTCGCT
ACGTACCCT---------------------------------------GCCGAGTACGTT
TCAGCAGCGGGTGGTTTTGGAGATCAAGTAATGGGAACGATGTTGCTTCTGCTTTGCATT
TGTGCCATTGTTGATAAGAAGAACGCGAACATCCCAAGTTCTCTGGTCCCCTTGTACATC
GGTTTCGTGATCTTGGCTATTGGAGTTTGTTTCGGCGCAAACTGTGGTTACGCCATTAAC
CCTGCCCGAGATTTGGCCCCGCGGTTCTTGACCTTTATCGGTGGATGGGGTACTGACGTT
TTCAGGTACCGG---------AATTACAACTGGTTTTGGGTACCTATTATTGGGCCACAT
ATTGGTGCCATTTTGGGAGTTCTGATCTACACCGTTTGC---ATCGAATTGCACTGGCCA
GAAACTTACGAAGCAACTGTTGACGATAAACGGAAC---
>RJJA01000048_Lepidurus_arcticus_Glp
---------------GAGCTGTTCCAGTCTTACCAA---ATTAAAACGCCGCTGATACGC
GAAATGTTGGCGGAATTTTTGGGAACCTTCTTGCTCGTGTTGTTTGGAGATGCCAGCGTG
GCGCAGTCTAAGCTCACCAACAAT---------------GTCAATGGAGATTTCTTTTCG
ATTAATTGGGGTTGGGGGTTAGCAGTCATGATGGGAGTTCTAGTAGCCGGAAAAGTATCA
GGTGCACACTTGAATCCTGCCGTCTCAGTCGCTTTTGCGATATTTGGCAAATTC---CCT
TGGCTGAAGCTACTGCCCTATGTATTAGCGCAATACCTTGGAGCCTTGGCTGCTGCAGCA
TCCGTTTTTGGGGTCTATTCAGAAGCTATATGGGATTTTTCG---GGCGGA---------
AATCTTACTGTTGATGGT---------------ATAACCGGCACCGCAGGTATATTTGCA
ACGTACCCA---------------------------------------GCGGAATACGTC
TCAGCTTCAGGTGGATTTGGGGATCAAGTCTTGGGAACCATGCTTCTCTTGCTCTGTATT
TGCGCCATTGTTGACAAGAAAAATATGTCGGTTCCGAGCGGTTTGGTTCCGTTATATATT
GGTTTCGTTATTTTGGCCATTGGAGTATGTTTCGGCTCCAACTGCGGTTATGCTATTAAC
CCTGCTAGGGATCTAGCGCCTCGCTTCCTTACCTTCATTGCCGGATGGGGTACTGATGTG
TTTAGGTATCGA---------GGCTACAACTGGTTCTGGGTACCTATCGTTGGCCCGCAC
ATTGGCGCCATCTTGGGAGTACTCATATACACTCTGTGC---ATTGAACTGCATTGGCCG
GAAACTCCAGAAGCTGGAAGGGACGATAAGAATGTT---
>RJJB01000217_Lepidurus_apus_Glp
---------------GAGCTGTTCCAGTCTTACCAA---ATTAAAACGCCGTTGATCCGC
GAAATGTTGGCGGAATTTTTGGGAACCTTCATGCTTGTGTTATTTGGAGATGCCAGCGTT
GCGCAGTCTAAACTTACCAACACA---------------GACAATGGTGATTTCTTTTCG
ATTAATTGGGGCTGGGGATTAGGAGTCATGATGGGAGTTCTAGTGGCCGGAAAAGTATCA
GGTGCACACTTGAATCCTGCCGTCTCTGTCGCTTTTGCGATATTTGGTCGATTC---CCT
TGGCTGAAGCTACTCCCCTATGTATTAGCGCAATACCTTGGAGCCTTGGCTGCTGCAGCA
TCCGTTTTCGGAGTCTACTCAGAAGCTATATGGAATTTTTCG---GGCGGG---------
AACCTTACTGTTGATGGT---------------AGTTCCGGCACCGCAGGTATATTTGCA
ACGTACCCT---------------------------------------GCGGAATACGTC
TCGGCTTCAGGTGGATTTGGGGATCAAGTTATGGGAACCATGCTTCTCCTGCTCTGTATT
TGCGCCATTGTTGACAAGAAAAACATGTCGGTTCCGAGCGGATTGGTTCCGTTATACATT
GGTTTCGTTATTTTGGCCATCGGAGTGTGTTTTGGCTCCAACTGTGGTTATGCTATTAAT
CCAGCCAGGGATCTAGCGCCCCGTTTCCTTACCTTCATTGCCGGATGGGGCACTGAAGTG
TTCAGGTATCGG---------GGCTACAACTGGTTTTGGGTGCCTATCGTTGGCCCGCAT
ATTGGCGCCATCTTGGGAGTGCTCATATACACTCTGTGC---ATCGAACTGCATTGGCCG
GAAACACCTGAAGTCGGAAAGGACGATAAGAATGTT---
>GCBP01024579_Eubranchipus_grubii_Glp
---------GAGAAATCAGTACGAAGCAAGTTGAGG---ATCAGTAGTCCCATCTTCAGG
GAGTTTGTGGCGGAGTTTCTAGGAACTTTTATTCTTGTTGTTCTTGGCGACGGCAGTGTT
GCTCAGTCCGTCCTTTCAGACAAA---------------GAGAAGGGAGACTTTTTCTCC
ATCAACTGGGGCTGGGGGATTGCCGTCATGCTGGCAGTCCTTATCTCGGGTGGCGTATCT
GGTGGTCACTTAAACCCTGCGGTGACAGTAGCAATGGCAGTCGTTGGAAAGCAT---CCT
TGGAAGAAAGTTCTTCACTATTGCTTGGCGCAGTACCTCGGCGCCTTCTTGGCCGCTGCA
TGCGTCCTTGGAATTTACAGTGAGGGAATTTACTACTTCGAACTAGATGGA---------
ACATTGAATGTC------------------------ACCAACACAGCTGGCATTTTTGCA
ACATACCCT---------------------------------------TACCTTTGGACG
ACTCTAACAGGTGGACTTGCTGACCAGATTCTTGGTACCATGATCCTCTTGCTCGTGATA
TGTGCCATTTCAGACAATCGAAATATGCAAGTAGCGAAGCCTCTTATCCCGCTGTACGTC
GGATTCACTATTCTTGGAATTGGGGTATGCTTTGGTGCCAACTGCGGCTATGCTGTGAAC
CCCGCGAGAGATTTGGCACCGAGGTTACTGACCCTGATTGCCGGTTGGGGACCTGAAGTT
ATGACAATAAAT---------GACTAC---TGGTTCTGGGTTCCGATCGTTGGCCCCCCAC
ATTGGTGCGATTCTCGGGGTACTCATATATTCCCTTTGC---ATAGAAGTTCACTGGCCA

GAAGATGATGAAACTGAAGCCGAAGCA------------
>LS050154_Acartia_tonsa_Glp1
---------------------GGCTGTTCCCCGCGC---ATCCGGAGCCCCGTGATGCGG
GAGTTTCTTGCGGAGTTTTTAGGAACCTTTGTGCTAGTTTTGTTTGGAAACTGTTCCATA
GCCCAATCTACCCTCTCTCTTAGT---------------AGCAAAGGAGACTTTTTCTCC
ATTAATTGGGGGTGGTGTGTTGGAATTATTCTTGGTATTCTTGTGAGCGGAGGAGTTTCA
GGAGGGCACTTGAATCCCGCAGTATCCGTAGCTGTGGCCACTCTTGGCAAGTTT---CCA
TGGTGGAAGGTTCCCCATTATCTTGGTGCGCAGTACCTTGGGTCCTTTGCCGCGTCTTGT
GTCACCTTCCTGGTCTACTGGGACGCCCTTGTCTGGTATGAG---CATAAC---------
CGTGGCGCGTACCGGTCC---------------ACCCCGGACACAGCGGGCATATTCGCC
ACGTACCCC--------------------------------------GCTCCCCATCTC
TCCTGGCTGGGCGGCATTGGGGACCAGATTTTGAGCACGGCGATGCTAGCGGCATGTGTA
TGCGCTATCACCGACAGCAAGAATATGAATGTAAGTAAGCAACTTGTACCGCTCTACATT
GGCCTGACTGTGCTTGGAATTGGAATTTGTTTTGGAGCTAACTGCGGATATGCCATCAAT
CCTGCCAGGGACCTTGCTCCTAGGCTGTTTACAGCCCTGGCTGGTTGGGGACCAGAGGTG
TTCTCTTTCTAT---------TCTCAG---TGGTGGGTGGTGCCCGTCATAGCCACCCAT
GTCGGAGCTATAGCTGGTGCTTGGCTGTACTTCCTTTGT---ATTGAAATGAACTGGCCA
AGGGATGTTGAATTTTCTGATGAGGAATAT---------
>GFWO01073465_Labidocera_madurae_Glp1
---------------AAGGCCCGCTGCTCCCCGCGG---ATCAAGAGCCCGATGGTGCGG
GAGTTCCTCGCCGAGTTTCTCGGGACCTTCATCTTGGTTCTGTTCGGGAACTCCTCGATC
GCCCAGTCGGTGCTCTCCCTCCAC---------------AACAAGGGGGACTTCTTCGCC
ATCAACTGGGGATGGTGCATCGGAATTCTCCTTGGGATTGTGGTCAGCGGGGGCGTATCA
GGAGGTCACCTGAACCCCGCTGTATCCGTGGCGGTCGCTACCCTGGGAAAATTT---CCC
TGGTGGAAGGTACCGCACTACCTGGCAGCGCAGTATCTTGGCGCATTCGTTGCGTCATGC
GTCACTTTCCTGGTCTACTGGGATGCGCTTGTCTGGTATGAG---CACAAC---------
CGGGGGATGTACCGGTCG---------------ACGCCGGACACCGCAGGGATCTTCGCC
ACGTACCCG--------------------------------------GCGCCGCACCTC
TCCTGGGCGGGCGGTGTCGGCGACCAGTTCCTCGGGACGGCGCTCCTCCTCGCCTGCGTC
TGCGCCATCACCGACAGCAAGAACATGAAGGTGAGCAAGCAGCTGGTCCCCTTGTACATC
GGGTTCACCGTGCTCGGGATCGGAGTCGCCTTCGGGTTCAACTCCGGGTACGCGATCAAC
CCTGCCAGGGACCTTGCACCCAGACTCTTTACAGCTATCGCTGGCTGGGGACCTGAGGTG
TTCAGCTTCTAT---------TCTCAC---TGGTGGATTGTTCCAGTCGTAGCTTGCCAT
CTTGGAGCTATAGCTGGAGCCTGGTTGTACTATCTCTGC---GTTGAAATGAACTGGCCC
AAGGACGTTGATATTGAATCTGAGGAACCC---------
>GGQN01024194_Temora_longicornis_Glp1
---------------AAGGGCGGGTGCTCCTTCAAG---ATTCGTAGCCCGGTGCTAAGG
GAGTTTCTCGCGGAGTTTTTAGGGACTTTTATCTTGGTTCTGTTCGGGAACAGCTCCATC
GCGCAGTCCGTGCTCTCCCTCAAC---------------AACAAGGGGGACTTCTTCGCC
ATCAACTGGGGATGGTGTATCGGGATCATCTTGGGTGTAATGGTGAGCGGCGGAGTCTCA
GGGGGCCACCTGAACCCCGCTGTCTCCGTGGCAGTTGCAACCCTTGGGAAATTC---CCA
TGGTGGAAGGTTCCGCACTACTTGGGGGCTCAGTACCTTGGAGCCTTCGCGGCGTCGTGC
GTCACCTTCCTCGTCTACTGGGACGCACTTGTCTGGTATGAG---CACAAC---------
CGTGGCGTGTACCGGTCC---------------ACCCCGGACACGGCCAGCATCTTCGCG
ACCTACCCC--------------------------------------GCCCACCACCTC
TCCTGGGCCGGGGGCGTGGCGGACCAGGTTCTTGGAACTGCTCTTCTTCTGATTTGTGTG
TGTGCTATCACCGACTCAAGGAATATGAACGTGAGCAAGCAGCTGGTCCCCCTGTACGTG
GGGTTCACCGTG--

>NW_019396719_Eurytemora_affinis_Glp1
---------------AAAGGGGGCTGTTCGCCCCGT---ATCAGGAGTCCGCTTCTTCGG
GAGTTTTTAGCTGAGTTTTTAGGAACATTTATCCTTGTCCTGTTTGGAACTGGATCTATT
GCACAGTCTGTTCTCTCTCTCAAC---------------AACAAGGGGGACTTCTTCGCT
ATAAACTGGGGGTGGTTTATTGGAATACTGCTCGGTGTGCTGGTTAGTGGAGGAGTTTCA
GGAGGACATCTGAACCCTGCAGTTTCTGTAGCTGTTGCTACACTTGGCAAATTC---CCT
TGGTGGAAGGTTCCTCACTACTTGGCAGCTCAGTATCTGGGAGCATTTACTGCTTCAACT
GTAGTATTCTTGGTCTACTGGGATGCTCTTGTCTGGTATGAG---CATGAT---------
CGTGGAGCATACCGTACA---------------ACCCCTGATACGGCCGGTATATTTGCC
ACCTACCCT--------------------------------------GGCAGGCATCTG
AGCTGGGCGGGCGGTGTCGGAGATCAATTCCTTGGTACTGCTATGCTACTGATCTGCCTC
TGTGCTATCACTGACAGAAAGAATATGAATGTAAGTAAGCAGTTGGTTCCCTTGTACGTT
GGATTTACTGTGCTGGGTATTGGAATCTGTTTTGGGTTTAACTGCGGCTATGCGGTCAAC
CCCGCTCGAGATCTTGCACCTAGACTTTTTACAGCACTGGCTGGTTGGGGCCCTGAAGTA
TTCAGTTACTAT---------TCTCAT---TGGTGGATAGTTCCTGTGGTTGCATCTCAT
CTTGGAGCTATAGCTGGAGCATGGATTTACTATCTGTGC---GTCGAGATGAACTGGCCA

AGGGATCTTGAACCTGAATCTGAGAAGGAG---------
>GAXK01165253_Calanus_finmarchicus_Glp1_v1
------------AGAAGCAGCTGCTGTAGTTTCAAG---ATCATCAGCCCTGTAATCAGG
GAGTTTCTTGCAGAGTTCCTCGGCACCTTTATTCTTGTCCTCTTTGGTACTGGCTCAGTG
GCCCAGTCAGTTCTATCTCTGGGA---------------CAGAAGGGAAGCTTCTTCTCC
ATTACCTGGGGGTGGGGTATTGGAGTTATCCTCGGTATTGTGGTAAGTGGCGGGGTGTCA
GGAGGCCATCTTAACCCGGCTGTTACCGTCGCAGTTGCATCTGTTGGAAAATTT---CCT
TGGCGCAAGGTTCCCCACTACCTTGCTGCTCAATATCTTGGAGCATTTGTTGCCTCTTGT
GTTGTTTTCCTTGTGTACTGGGATGCTCTGGTCTGGTATGAA---CATGAC---------
CGAGGAGAATACCGCACC---------------ACTCCTGACACTGCCAGCATTTTCTCC
ACCTACCCC---------------------------------------TCGCCACACCTG
ACCTACATGGGCGGTATAGTGGACCAGGTGGTGGCCACAGCCTTGCTACTCATCTGTGTC
AGTGCCATCACGGACAGGCGCAACATGCAGGTGTCAAAACAGTTGGTCCCTTTCTTCATT
GGCCTCACTGTTCTCGGCATTGGAATCTGTTTTGGCTTCAACTGTGGCTACGCTATCAAC
CCAGCAAGAGACTTGGCCCCACGGTTGTTCACTGCTCTATCCGGGTGGGGGCTGGAAGTT
TTTGCCTTTTCC---------AGCCCC---TGGTGGTTGGTGCCGATACTTGGCACCCAT
GCAGGGGCTGTGCTTGGTGCCTGGGTGTACTATCTAGCC---ATAGAGCTGCATTGGCAC
AAGGATGATACTGAGGATACT------------------
>GBFB01029789_Calanus_finmarchicus_Glp1_v2
------------AGAAGCAGCTGTTGTAGGTTCAAG---ATCAGTAGCCCTGTCATCAGG
GAGTTTCTTGCAGAGTTCCTCGGCACCTTCATTCTTGTCCTCTTTGGTACTGGCTCAGTG
GCCCAGTCAGTTCTCTCTCTGGGA---------------CAGAAGGGAAGCTTCTTCTCC
ATTACCTGGGGGTGGGGTATTGGAGTAATCCTCGGTATTGTGGTAAGTGGCGGGGTGTCA
GGAGGCCATCTTAACCCGGCTGTTACCGTCGCAGTTGCATCTGTTGGAAAATTT---CCT
TGGCGCAAGGTTCCCCACTACCTTGCTGCTCAATATCTTGGAGCATTTGTTGCCTCTGGT
GTTGTTTTCCTTGTGTACTGGGATGCTCTTGTCTGGTACGAA---CATGAC---------
CGAGGAGAATACCGCACC---------------ACTCCTGACACAGCCAGCATTTTCTCC
ACCTACCCC---------------------------------------TCGCCACACCTG
ACCTACATGGGCGGTATAGTGGACCAGGTGGTGGCCACAGCCTTGCTACTCATCTGTGTC
AGTGCCATCACGGACAGGCGCAACATGCAGGTGTCAAAACAGTTGGTCCCTTTCTTCATT
GGCCTCACTGTTCTTGGCATTGGAATCTGTTTTGGCTTCAACTGTGGGTATGCTATCAAC
CCTGCCAGGGACTTGGCCCCAAGGTTGTTCACTGCTCTATCTGGGTGGGGGCTGGAAGTT
TTTGCCTTTTCC---------AGCCCC---TGGTGGTTGGTGCCGATACTTGGCACCCAT
GCAGGGGCTGTGCTTGGTGCCTGGGTGTACTATCTAGCC---ATAGAGCTGCATTGGCAC
AAGGATGATACTGAGGATAGTGAGGAAGGA---------
>GAXK01070720_Calanus_finmarchicus_Glp2
---------AAAGGAGGCTGCTGTGCTTCATGGCGA---ATAACCAGCCCGGCTTTGAGA
GAGTTTTTGTCTGAGTGTTTTGGCACTTTCATCCTGGTTGTGTTTGGTTGTGGCTCCATT
GCCCAGTCAGTCCTCTCGCTTCAG---------------ACAAAAGGAGACTTTTTCTCC
ATCAACTGGGGGTGGTGTCTAGGAGCCACCCTTGGAACTCTAGTCAGTGGAGGAGTGTCA
GGCGGTCACTTGAATCCTGCGGTTACAGTGGCCCTTGCTACCCTGGGAAAAGTT---CCC
TGGAAAAAGGTTCCTCACTACCTGGCAGGTCAGTACTTGGGTGCTTTTATTGCTTCCTTT
GTTGTATTCTTGGTCTACTGGGATGCTCTAGTCTGGTACGAA---CATGAC---------
AGAGGAGGATACCGCATT---------------ACTCCAGACACTGCTGGCATCTTCGGC
ACCTACCCT---------------------------------------TCTCACCATCTC
ACCTATGCTGGAGGTGTTGGGGATCAGGTTCTTGGGACGGCCCTTCTCTTGCTCTGTGTT
TGTGCAATCACGGATAAGCGGAACATGCAGGTGAGCAAGCAACTGGTTCCCTTCTTTGTT
GGTCTGACTGTTCTTGGTATTGGGATCTGCTTTGGTTACAACTGTGGCTATGCTGTCAAC
CCTGCTAGAGATTTTGCACCACGGCTGTTTAGCGCCCTGGCTGGGTGGGGGCCGGATGTG
TTCACCTACTCT---------TACCAC---TGGTGGGTTGTTCCCATATTTGCTACTCAT
GTGGGAGCTGTGCTGGGTGCTTGGCTATACTACATTGCT---ATAGAACTCAACTGGCCA
ACAGAGGAACCTGATGTTGAAAAACACAAT---------
>GFCI01389685_Pleuromamma_xiphias_Glp1v1
---------GAAAAGAAAAGAGGCAAACGCTGGAAA---ATTACAAATAATGTTGTGCGG
GAGTTTTTGGCTGAATTTTTAGGAACATTTGTTTTGGTTCTGTTTGGCGATGCATCCATA
GCTCAAGCCGTCTTGTCTTTGGGA---------------TCTAAGGGAGATTTCTTTGCA
ATAAATTGGGGGTGGGGCATGGCAGTAATGCTCGGCGTGTTGGTGAGCGGCGGAGTATCT
GGAGGTCATCTCAACCCGGCGGTCACCGTGGCCGTCGCCACGCTCGGAAAGTTT---CCT
TGGTGGAAGGTCCCTCACTATCTGGCTGCTCAATACCTTGGAGCATTTACGGGAGCAACT
GTGGTGTTCCTCGTCTACTGGGATGCGCTTGTCTGGTATGAA---CACGAC---------
AGGGGACAATACCGCACG---------------ACCCCCGACACCGCGGGTATTTTCGCC
ACGTACCCC---------------------------------------TCCCCCCACCTC
ACCATCGCGGGCGGGGTGGGCGACCAGTTCCTGGGGACCGCCATGCTGCTTATCTGCGTC
TGCGCCATCACAGACAGGAAGAACATGCAGGTTAGTAAACAAATGGTCCCTCTGCTGATC
GGGTTGACGGTTCTGGCTATCGGAGTCTGCTTCGGCTACAACTGCGGCTACGCCATCAAT
CCGGCGAGAGACCTCGGTCCAAGGCTATTCACTGCCATTGCCGGGTGGGGCGGCAGGTG
TTCAGTTATTAC---------AACCAC---TGGTGGGTGGTGCCCGTGGTGGCGTCTCAC
GTGGGCGCCATTGTGGGCGCGTGGATCTACTTCCTCGCC---ATCGAGCTCAACTGGCCC

ATCGACAACGACGACAGCGACGACACCGGC---------
>GFCI01389683_Pleuromamma_xiphias_Glp1_v2
---------GAAAAGAAAAGAGGCAAACGCTGGAAA---ATTACAAATAATGTTGTGCGG
GAGTTTTTGGCTGAATTTTAGGAACATTTGTTTTGGTTCTGTTTGGCGATGCATCCATA
GCTCAAGCCGTCTTGTCTTTGGGA---------------TCTAAGGGAGATTTCTTTGCA
ATAAATTGGGGGTGGGGCATGGCAGTAATGCTCGGCGTGTTGGTGAGCGGCGGAGTATCT
GGAGGTCATCTCAACCCGGCGGTCACCGTGGCCGTCGCCACGCTCGGAAAGTTT---CCT
TGGTGGAAGGTCCCTCACTATCTGGCTGCTCAATACCTTGGAGCATTTACGGGAGCAACT
GTGGTGTTCCTCGTCTACTGGGATGCGCTTGTCTGGTATGAA---CACGAC---------
AGGGGACAATACCGCACG---------------ACCCCCGACACCGCGGGTATTTTCGCC
ACGTACCCC---------------------------------------TCCCCCCACCTC
ACCATCGCGGGCGGGGTGGGCGACCAGTTCCTGGGGACCGCCATGCTGCTTATCTGCGTC
TGCGCCATCACAGACAGGAAGAACATGCAGGTTAGTAAACAAATGGTCCCTCTGCTGATC
GGGTTGACGGTTCTGGCTATCGGAGTCTGCTTCGGCTACAACTGCGGCTACGCCATCAAT
CCGGCGAGAGACCTCGGTCCAAGGCTATTCACTGCCATTGCCGGGTGGGGCGGGCAGGTG
TTCAGTTATTAC---------AACCAC---TGGTGGGTGGTGCCCGTGGTGGCGTCTCAC
GTGGGCGCCATTGTGGGCGCGTGGATCTACTTCCTCGCC---ATCGAGCTCAACTGGCCC
ATTGACAACGACGACAGCGACGACACAGGC---------
>GHWV01136883_Pseudodiaptomus_annandalei_Glp1
---------------AAATCGCGATGCTCTCTCAGG---ATAGAGAGTGTTGTGATTCGG
GAATTCCTGGCAGAGTTTCTCGGGACCTTTGTCCTGGTGACCTTTGGCGCCGGGTCCGTG
GCCCAGTCCGTGCTCAGTCTCAAC---------------AACAAGGGAGACTTCTTCGGC
ATCAACTTCGGTTGGGGTCTTGGCGTGATGATGGGCATCCTGGTGAGCGGCGGCGTGTCT
GGCGGTCACCTCAACCCTGCCATCACCGTGGCCGTGGCCACCCTCGGCAAGTTC---CCC
TGGTGGAAGGTGCCGCACTACATCGCTGCCCAGTATCTCGGGGCGTTCACCGCCTCCTTC
GTCATCTTCTTTGTCTACTGGGATGCTCTTGTCTGGTATGAG---CACGAC---------
CGCGGGGCGTACCGCTCG---------------ATCCCCGACACGGCGTCCATCTTCGCC
ACCTACCCG---------------------------------------GCGCCGCATCTC
ACCTGGGCTGGAGGAATGGGCGATCAGTTCTTGGGAACTGCTCTGCTGCTCATCTGCGTC
TGTGCCATCACTGATCAGAAGAACATGAATGTGAGCAAGCAGCTGGTTCCTCTCTTCGTC
TTCTTCACGGTCTTGGCTATCGGGGTCTGCTTTGGCTTCAACTGCGGCTATGCCATCAAC
CCCGCTAGGGATCTCGCCCCCAGGTTGTTCACTGCCATTGCTGGTTGGGGCCCTGGAGTC
TTCACCTCCTAC---------AACCAT---TGGTGGGTGGTTCCCGTAGTGGCCTCTCAT
ATTGGAGCCATTGTTGGAGCTTGGGTTTACTACCTTAGC---GTTGAGCTGAACTGGCCC
AGGGAATGTGACATGGATGACGACGTGCCA---------
>CM017381_Tigriopus_californicus_Glp1
AAAAACATTGTCGCGGAAAAGATCCGCAATGTGCGG---GTTACCAACGATCTCGTCCGG
GAGTTCATGGCTGAATTTCTCGGTACCTTCGTTCTCATTCTCTTTGGCAATGCCGTCGTT
GCCCAAGTGGTTTTAAGTAATGAA---------------GAGAAGGGCGATTTCTTCTCC
ATCAACTGGGGATGGGGCGTGGCCGTGACCTTGGGCGTGCTCGTGTCCGGTGGAGTTTCT
GGAGGGCACATCAACCCCGCAGTTACATTGGCCTTGGCGGTCATTGGTAACACA---CCG
TGGAAGAAGGTCCCCTTGTACATGGCCGCCCAATACTTGGGAGGCTTTATCGCCTCTGGC
CTCTTGTACGGAATCTACTATGAGGCTTTGAACGCCTTTGAG---GCTGAG---------
AATGGATTGGATCGAGCC---------------ACCCCCGAAACGGCTGGCATTTTTGCC
ACCTACCCT---------------------------------------TCCCCTTTCCTC
AGCCACCACAGTGGATTGCTAGACCAGGTGGTTAGCACCGGTTTATTGATGCTTCTAATT
TGTGCCATTTGCGACAAGAAGAACATGGGTGTGTCCAAGGGTATGGCTCCACTCTACATT
GGATTCATCGTGCTCAACATCGGCATCTGCTTCGGGCACAATTGCGGCTACGCCATTAAT
CCAGCTCGAGATCTTGCCCCCCGCTTGTTCACACTTGTAGCTGGATGGGGCACTAAACCG
TTTACCATTGAA---------AACCAT---TGGTGGGTCGTCCCGGTCATTGGAACCCAT
CTTGGGGCAGTCTTGGGTGCTCTTATGTATTGCTTCTGC---ATTGAAAATCATTGGAAA
CAAAGCGCTTATGAGCTAGATGTGATCAAGGTG------
>GCHA01010929_Tigriopus_japonicus_Glp1
AAGTATATTGTCGCGGAAAAGATCCGCAATGTGCGG---GTTACCAACGACCTCGTCCGG
GAGTTCATGGCTGAATTTCTCGGCACCTTCATCCTCATCCTCTTTGGCAATGCCGTCGTT
GCCCAAGTGGTATTGAGTAACGAG---------------GAGAAGGGAGATTTCTTCGCT
ATTAACTGGGGTTGGGGTGTGGCTGTGACTTTGGGCGTGCTCGTGTCAGGCGGAGTTTCT
GGCGGTCACATCAATCCCGCAGTTACCTTGGCCTTGGCGGTGATTGGAAATACA---CCC
TGGAAAAAGGTTCCCTTGTATATGGCGGCTCAATACTTGGGAGGCTTCGTCGCTTCCGCT
CTTTTGTACGGAATCTACTATGAAGCTTTGAACGCCTTTGAG---GCTGAG---------
AATGGTTTGGACCGAGCC---------------ACCCCTGAAACCGCTGGAATTTTTGCC
ACCTATCCC---------------------------------------TCCCCTTTCCTC
AGCCATCACAGTGGATTGCTGGACCAAGTGGTTAGCACTGGGTGTTTGATGCTCCTGATC
TGCGCCATTTGCGACAAGAAGAACATGAGTGTGTCTAAGGGAATGGCTCCACTTTACATT
GGATTTATTGTGCTCAACATTGGAATTTGCTTCGGTCACAATTGCGGCTACGCCATCAAT
CCTGCACGCGATCTTGCCCCCCGCTTATTTACACTTGTTGCTGGATGGGGAACCAAGCCA
TTTACCTTCTCA---------AATCAT---TGGTGGGTCGTCCCTGTCATTGGAACTCAT
CTTGGAGCTATTTTGGGCGCTCTTTTGTACTGCCTCTGC---ATTGAAAACCATTGGAAA

AAGAGCACCTATGAGTTGGAAATGATCAAGGTG------
>JABCAI010000014_Tigriopus_kingsejongensis_Glp1
AAGTCTTTTCTGGGACAAAGGATCCGCAACCTGAGG---ATCACCAACGAATGGGTCCGA
GAGTTCTTGGCCGAGTTCCTCGGCACCTTCGTCCTGGTCCTCTTCGGCACTGCCGTCGTG
GCCCAGGTGGTGCTGAGCAATGAG---------------GAGAAAGGCGATTTCTTCGCC
ATCAACTGGGGCTGGGGTGTGGCAGTGACTTTGGGGGTGCTCGTGTCCGGCGGCGTGTCC
GGGGGTCACATCAACCCGGCGGTCACCCTGGGATTCGCGGTCATTGGCAACTTC---CCG
TGGAAGAAGGTGCCCCTGTACATGGCAGCCCAGTACCTGGGGGGATTCGTGGCCTCGGCC
ACGGTCTTTGGCGTCTACTATGAAGCTCTCAACGCCTTCGAG---GAGTTT---------
AACGGCTACAGCCGAGCC---------------ACGCCTCAAACTGCTGGCATCTGGGCC
ACCTATCCC---------------------------------------TCCGAGTTCCTG
AGTCACCCCAGTGGGATCTTCGACCAGGTGGTGAGCACGGCCTTGCTCATGCTCCTCATT
TGCGCCATTATTGATAAGAAGAATATGAATGTCGCCAAAGGCATGTACCCCCTGTACATT
GGCTTCGTCGTCTTGAACATTGGGATTTGCTTCGGGCACAACTGCGGCTACGCCATCAAC
CCCGCTCGTGACCTCGCGCCCAGGATCTTCACGGCCATTGCTGGCTGGGGCACGGAGCCT
TTCACCCATGCC---------AACTAC---TGGTGGGTGGTTCCCATCGTGGGCAGTCAC
ATTGGGGCAGTTCTGGGCGCCCTGATCTACTGCTTCTTC---GTCGAGAACCATTGGAAG
GAAACCTCTTTCGAGCTCGAGATGATTAAAATG------
>LR144051_Tisbe_holothuriae_Glp3
GAAAATCAGTTTTCCCGTGGATTGAAAAGACTGCGA---ATCCGAAACCAGATTGCCAGG
GAGTTCCTATCCGAACTGGTCGGTAAGCTCATCCTCATAACCTATGGCGAGGGCGCCATC
TCCACCGAGAAGATCAGCGCCGGCCTCGGCGAA------CCCAATGGCTCTGCAGTGGCC
ACCAACCTCAACTGGGGCATCGCCGTCATGATCGCGGTCACCGTTGCCATGGGAGCCAGC
GGAGCGCATCTCAACCCTGCCGTGACTCTGGCATTCACTGCAGTTGGCAAATTC---CGC
TGGACAAAAATGTTCCACTACTTATTGGCTCAGTACCTGGGCTCTTTCATTGGTGCCGCT
TTGGTCTACGGGTTCTATTACCAAGCCTTGGAAGAGTGTGAAGACCACGGA---------
GAGTGCGGCGACAAGGAT---------------GTCCCTGGCACTGCCGGAATATTTGCA
ACATACCCC---------------------------------------AAGGAGTATGTC
AGTTTGGGGGCTGCCATGGCAGATCAGATTATGGTGACATGCTTTCTAATGTTCTGTATT
TGTGGCATCACTGATCCGAAAAATATCCGTATGCCAACATATCTGTGGCCTCTCTACATT
GGCTGCTTGGTGTTCAACTCAGGAACCTGTCTCGGGTTCAACTGCGGTGCGGCTATGAAC
CCATCCAGGGATCTGGCGCCGAGGATATTCAGCTCGATTGCAGGTTGGGGAAGTGAAGTG
TGGTCGTATGGA---------AACCAC---TTTTGGTTCGTGCCTATTGTTGGGAGTCAC
ATTGGCGCTATACTTGGCGCGATCTTCTACTCACTAGCC---ATTGAGAACCACTGGCCG
GAATCTGAAAGGACAACCAAAACTGTTTCCGAT------
>LR144051_Tisbe_holothuriae_Glp2
GACAACCCCATCGCCAGAGCCATCCGCTCAGTGAGG---ACGGACAATGTCATCGTAAGG
GAGTTTCTCGGAGAGTTTATAGGTACGGCCCTTCTCATTGTCTTTGGCGAGAGCGCCAAC
TCGATCTTCGAGCTGAGCGCCGGAATGGGAATC------CCAGTG------AACGTGGCG
CTCAACTTCAGCTGGGCCGTGGCCGTCATGCTGGCGCTGTGCGTAGCCGGGGGCGCGAGT
GGCGCACATATTAACCCATGTGTCACTGCAACATTCGTAATTACAGGTCGATTA---CAC
TGGTCCAAGCTGTTCCACTATCTGCTGGGGCAGTACCTCGGCGCGTTTGTCGGCGCGGCC
ATCGTGTACGGCAACTTCTACCTCGCTTTGGACGAGTGTGAGACGAATGGG---------
ACGTGCCCGGACAAGGGGACG------------ATCCCGGGCACGGCCGGGATCTACGCG
ACCTACCCG---------------------------------------AAGGAATACATG
ACCATTGGGGCGGCACTGGGAGATCAGATTCTCGCTTCGAGTCTCTTTCTTCTTTGCGTC
ATGGGAATCCTTGACCAGAGGAATATGAAGATCCCGCTCCGATTGGCGCCGCTCTACATC
GGACTTGTCATCTTCAACATTGGATCTTGTCTGTCGCTCAACTGCGGGGCAGCAATCAAT
CCGGCGAGAGATCTGGGGCCGAGGATTTTCACTGCAGTTGCTGGTTGGGATAATGATATT
TGGACATACGGC---------CACAGC---TTTTGGTTCGTGCCCATAGTGGGCCCGCAT
ATTGGCGGCATCCTCGGGGCAGTCGTCTACCTGCTCTGC---ATTGAGAACCACTGGCCA
AAGATAGAAGAAGAAGAGAGTGAGATAAAA---------
>LR138471_Tisbe_holothuriae_Glp1
GACAATCCTCTGGCTAAAGGGCTTCGATGCCTCAGG---ATACGGCAGAACTTTGTCAGA
GAGTTCTTAGCGGAACTATTTGGCACATTTATCTTGATTGTGTTTGGCGACGGCGTGGTT
GCCCAGGTGGTGCTGAGCGACGGCCTCAACGCC------TCCGTCGGCAGCTTCTTCACC
ATCAACTGGGGCTGGGGCGTGGGCGTCGCACTCGCAGTGCTGGTGGTCGGCGGCGTCAGT
GGGGCGCACATCAACCCCGCCGTCACCACTGCTATGGTGGTTGTAGGTAAGCTC---CAC
TGGTCCAAGATGTTCCACTACCTCCTTGCTCAATACTTGGGCGCATTCCTTGGTGCCAGC
GTTGTCTATGGGGTCTATTACGAGGCTCTGGAAGCGTGTGAGGCTAATGGA---------
ACATGCGGGGCTAGATCT---------------GTCCCTGGCACTGCCGGCATCTATGCC
ACTTACCCC---------------------------------------AAGGACTTCCTG
AGCATTGGTGGAGGGATGACCGATCAGATTGTCGGCACCATGCTGCTGCTCATCTGTGTG
TGCGCTATTGGAGACCAGAAAAACATGCGCATCCCTTCGTATCTTGCCCCGCTCTATGTT
GGCTTCACTGTGCTCAACATTGGAATCTGCTTCGGATTCAACTGCGGATATGCCATCAAC
CCTGCCAGGGATCTGGGACCAAGAATCTTCACAGCCATCTCCGGTTGGGGTAGCGACGTC
TGGAGCTTTGGA---------GATAAC---TGGTGGTACGTGCCTGTGATTGGCCCCCAC
GTGGGCGCCCTCCTCGGCGCCCTCCTCTACATGCTCTTC---ATTGAGAACCACTGGCCC

AAGCACGAGACGCTCACCATTAACAGGTCAAAC------
>GCIT01007364_Tisbe_furcata_Glp1
GACAATCCGATTGCAAAAGGACTTCGGTGTCTCAGG---ATTAGGCAGAACTTTGTCAGG
GAGTTCTTTGCCGAACTATTTGGCACGTTCATCTTGATTGTATTTGGCGATGGCGTTGTC
GCCCAGGTCGTGCTCAGCGACGGCCTCGACGCC------TCCGTCGGCAGCTTCTTCACC
ATCAACTGGGGATGGGGCGTGGGCGTCACACTTGCAGTGCTGGTTGTAGGAGGCGTCAGT
GGGGCGCACATTAACCCG--

------------AGATCT---------------GTCCCTGGCACTGCTGGAATCTATGCC
ACTTACCCC---------------------------------------AAGGACTTCCTG
AGCATTGGTGGAGGAATGGCCGATCAGATTGTGGGCACCATGCTTCTGCTCATCTGCGTG
TGCGCTATTGGAGATCAGAAAAATATGCGCATCCCCTCGTATCTTGCCCCGCTCTACGTT
GGATTCACTGTGCTCAACATTGGGATCTGCTTCGGATTCAACTGTGGCTATGCCATCAAC
CCTGCAAGGGATCTGGGACCAAGGATTTTCACGGCCATCTCAGGTTGGGGCAGTGACGTT
TGGAACTTTGGA---------GACAAC---TGGTGGTACGTTCCTGTGGTTGGCCCTCAT
GTTGGTGCCCTCCTTGGTGCCCTGCTCTACATGCTCTTC---ATTGAGAACCACTGGCCC
AAGCACGAGACGCTCACTATTAACAGG------------
>CM017373_Tigriopus_californicus_Glp2
---------ATGGGTCGAATCCTCAGGACAATCCGA---GTTAGGAATCAAGTGGCTCGG
GAATTCCTAGCGGAGTTCTTAGGAACCATGATCCTCATCCTCTTTGGCGATGGCGTTGTA
GCTCAAGTCACGTTGAGTGAAGGTCTGGGGATG------CCTAAGGGCAATTTCTTCACC
ATCAACTGGGGTTGGGGTTTGGGGGTTACCATGGCTGTGCTCATTGCGGGGGGCGTGTCC
GGAGCCCATATCAATCCTGCCGTCACTTTGGCCATGGCTGTTATCGGTAAGTTG---CCA
TGGATCAAAATGTTCCATTATATGCTCGCTCAATATCTCGGAGCCTTTGTAGGCGCAGCC
TTGGTCCATGCTAATTATTACGAAGCCCTGGTTGCATTCGAG---ACAGCT---------
CAAGGCGAAAGTCTT------------------ATCCCAGGCACAGCCGGGATCTTTGCA
ACCTACCCG---------------------------------------GCCGATTACCTT
AGTAATGGGGGAGGATTTCTAGACCAGATCATCGGCACTGCACTTCTACTCATGGTGGTT
TGTGCTGTGGGTGACCAACGAAACATGAAGATCTCGAGCCAAATGGCTCCTCTCTACGTC
GGGTTCACGGTTCTAGCCATTGGCGTGTGTTTCGGTGTCAATTGTGGATATGCCATCAAT
CCTGCCCGAGATTTGGGACCGAGGCTGTGGACATTGATCGTCCATTGGGGAACGGAACG
TTTAGTTACGGA---------AACTAT---TGGTTCTATGTTCCCATTCTGGCCCCTTAT
TTGGGCGGCATCATTGGGGCTGGACTTTATTTCGTGTGT---ATTGAAAATAATTGGCCA
GAGAAATTTGGA---------------------------
>GCHA01021727_Tigriopus_japonicus_Glp2
---------ATGGGTCGAATCCTTAGAGCTATCCGA---ATCAGGAATGAAATGGCTCGG
GAATTCTTGGCGGAACTCCTTGGAACCATGATTCTTATTCTTTTCGGCGATGGCGTTGTC
GCTCAAGTGACTTTAAGTGAAGGTCTGGGAATC------CCAAAGGGTGATTTCTTCACT
ATCAATTGGGGTTGGGGATTGGGCGTTACCATGGCGATTCTCGTTACTGGAGGCGTGTCT
GGTGCTCATCTTAATCCCGCCGTTACTTTGGCAATGGTGGTTGTTGGAAAAATG---CCA
TGGAAAAAAATGTTCCACTACATGCTTGCACAATACCTTGGAGCCTTCATAGGTGCCGCT
CTCGTCCATGCCAACTACTACGAAGCTCTTTCCGCTTTCGAG---GCTACT---------
CAAGGCGAGAGCCTC------------------ATTCCGGCCACAGCTGGGATCTTTGCT
ACTTATCCA---------------------------------------TCCGATTATCTA
AGTATTGGAGGAGGATTTCTAGACCAAATCATTGGTACAGCCCTCTTACTCTTGGTTGTT
TGTGCGGTGGGTGACCAACGAAATATGAAGATTTCAAGCCAGATGGCGCCTCTCTACGTC
GGGTTCACCGTTCTGGCCATTGGAGTGTGTTTTGGTGTCAATTGTGGATATGCCATTAAT
CCAGCTCGAGATTTGGGACCGAGGCTGTGGACTTTGATTGTTCATTGGGGAACAGGGACG
TTCAGTTATGGG---------AACTAT---TGGTTCTATGTACCTATTCTAGCTCCTTAC
TTGGGAGGGATCATCGGGGCTGGGCTGTATTTCGTATGC---ATTGAAAACAATTGGCTT
GAGAAAGTT------------------------------
>JABCAI010000038_Tigriopus_kingsejongensis_Glp2
GGTGACATCATGAGCAAGCTGCTGAAAAGAATCCGG---ATCCAGAATGAAACGGCCCGC
GAGCTACTGGCCGAGATGCTGGGGACCATGATCCTCATCCTCTTCGGGGATGGCGTAGTG
GCCCAAGTCACCTTAAGCGATGGCCTCAACATG------GACAAGGGCAATTTCTTCACC
ATTAATTGGGGGTGGGGCTTGGGCGTGACCATGGCCATCTTGGTGACAGGGGGCGTGTCT
GGGGCTCACCTCAATCCCGCCGTCACCCTTGCCATGGTAGTAACGGGTAAGATG---CGC
TGGACCAAGATGTTCCACTACATGCTGGCCCAGTATATTGGGGCCTTCCTTGGAGCCGCC
TTGGTCTACGCCAACTACCATGAAGCCCTGGCAGCCTACGAG---AAGTTC---------
AATGGCCCCGGCCTA------------------ATCCCGGAGACGGCGGGCATCTTTGCC
ACCTATCCG---------------------------------------GCCGACTACCTC
AGCATTGGCGGAGGCTTTTTGGACCAGATCCTCGCCACGGCACTCCTTTTGATAGTGGTG
TCTGCCGTTGGGGACCCGAGGAACATGAAGATCTCAAGCCAACTGGCTCCCCTCTACGTG
GGCTTCACCGTCCTTGCCATCGGCGTGTGTTTTGGAGTCAATTGCGGCTACGCCATCAAT
CCGGCCCGGGACTTGGGACCGAGATTGTTCACGCTCATGGTGAATTGGGGCAAGGGCACC
TTCACCTATGGT---------AATTCC---TGGTTTTACGTCCCCATTGTGGGTCCATAT
ATTGGTGGAGTCGTGGGGGCGGTTCTATATGTTGTGTGC---ATTGAAAATAACTGGCCG

GAGAAATTT------------------------------
>GHXK01261110_Platychelipus_littoralis_Glp2
AAACTAAATTTGGAACCTTTACTTAAGAAAATTCGA---ATAAGAAATGATTACGCCAGA
GAATTCTTGGCCGAGTTTTTTGGCACTTGCATCTTGCTCACTTTTGGCAATGGGGTCGTT
GCCCAGGTCACTCTATCAGAAATGCTTGGAGAC---CCAACATGGGGAAATTACTTAACC
ATAAGCTTGGCATGGACAGGAGGCCTCATGATGGGCGTGTTAATTGCTGGAGGGGTGTCA
GGTGCTCATCTCAACCCTGCAGTAACAACAGCTTTAATTTTAGTAGGCAAATTT---CCC
TGGTACAAAATGTTTCACTACATGTTGGGTCAGTACCTTGGGGCTTTTATTGGGGCCTCT
CTTGTTTATTTGACATACTTTGATGCCTTCAGGGCCGTTCAC---GATTTG---------
AATGGTTTC------------------------ACCCAAGACACTGCAGGCATTTTTGCT
ACGTACCCA---------------------------------------TCACCGCATTTA
TCTACGGGAGGAGGCTTTCTAGACCAGGTTGTTGGGACTGCTCTTCTTCTGTTCTGTGTA
CTCGCAATTACTGATAACAAAAACATGAAGGTCGGCTCATGGCTGATTCCATTGTACCTT
GGATTTCTCCTCATCTCCATTGGCCTGGCCTTTGGTTTCAACTCAGGCTTTGCTATTAAC
CCGGCAAGAGACTTGGGACCAAGAGTGTTCACAGCCATGGCCGGATGGGGTCTGGATGTA
TTTAGATGGGGC---------AACCAC---TGGTGGTGGGTTCCTGTGGTGGCTACTCAC
GTTGGCGGGGCCATTGGAGCGTGGTTATATGTCTTGGGT---ATTGAAAATCATTGGCCT
GTCGAGGCTGTGCACGAAAAAGATCAAAAC---------
>GHXK01158147_Platychelipus_littoralis_Glp1
AAATTCAACCTGGAGCCTTTGCTGAAGAAATTTCGA---ATAAGAAATTTGCACTTCCGT
GAGTTTTTGGCCGAGATGTTAGGCACGTTCATCCTGATCACTTTCGGGGACGGGTCGGTC
GCCCAGGTCACTTTGTCCAGCTTGCTAAACGAT------TCGAGGGGCAGCTTCTTCTCA
ATAAACTGGGCGTGGGGCGTGGCCGTAATGTTGGGCGTGTTAACAGCAGGGGGCGTGTCA
GGAGCACACATCAATCCAGCCGTTACCACATCTCTAGTGCTTGTCGGCAAGTTT---CCT
TTGTACAAGATGTTTCACTACATCCTGGCTCAATACATTGGGGCTTTCTTGGGTGCCTCT
TGTGTCTACCTCAACTACTTTGAGGCCTTTGAAGAAGTTCAC---ATGAAG---------
AATTCAAAGTCCAAG---------------------------ACGGCCGGAATATTTGCC
ACTTATCCA---------------------------------------GTTGACCATGTC
TCCATAGGAGGAGGATTTGTGGACCAGACCATAGGGACAAGTCTCCTACTCTTCCTTATC
CTTGCCATCACAGACAGAAGAAACATGAAGTTGACCTCCCTCCAAACGCCTTTGTACCTG
GGCTTTGCCGTCTTGAACATCGGCCTGGCCTTCGGGTACAACTGCGGCTACGCCATCAAC
CCGGCCAGGGATCTGGCACCTCGTCTCTTCACAGCCCTGGCCGGGTGGGGTGTGGACGTG
TTCACGTGGTCC---------AATCAC---TGGTGGTGGGTGCCAGTCGTGGCCTGCCAT
TTGGGTGGAGCAATCGGGGCAATTGTCTACGTTTTGGCC---ATCGAGAATCATTGGCCC
GTTGAAGAACATGAAAAAGAACAAACTAGAAGT------
>ALA27397_Lepeophtheirus_salmonis_Glp1_v1
AAAGAAATCCAATTCGAGAATGACCATCCTTGTTAT---ATTCCTACCCGTCCTCTAAGA
GAATTTTTTTCAGAGTTTATAGGAACTTTGATCTTCATTACAATCGGTATAGGAACTGTA
GCTCAAGTTACACTAAGTGGCATTCTCAAGAGT------CCTGCAGGCACATTTATATCT
ACAAATTGGGGGTTTGGATTCGCTATTATGATTGGAATGATGGTGTCAGGTAAAATATCT
GGAGGTCATATGAATCCAGCTGTGACAATTGCCTTTGCTATCTTTGGTAAACTAAAACCA
TGGCATAAATGTTTTCATTATATGTTCGCCCAATATTTGGGAGCCTTTTTTGGTTCGGCA
CTCGTTTATATTACTTACTATGAAGCTTTTCAAGAAGCC------GGT------------
ATC------------------------------AACATTGACACTGCAAAAATATTCGCA
ACATACCCT---------------------------------------GCTCCTCATATT
TCGGCTCCAGGAATTGTTCTTGACGAATTTGTTGGAACAGCCCTTCTATTAATGTCAGTG
TGTGCTATAATTGATAAAAAAAATCTGAACATTCCATCCTTTTTCCATCCATTTTTAATT
GGACTCGTGGTATTTGTCATAACAATGAGCTTTAGTTTTAATGCAGGTGCCGCTTTGAAC
CCAGCGAGAGATTTAAGACCTCGATTATTTACAGCAGTTTTTGGCTGGGGACTAGATCCC
TTTTCATATGCC---------AAACAT---TTCTGGATGACACCCATTTTAGCGACGCAT
GCAGGGGCTATAATTGGTGTTTTGATATATCAAACTTTT---ATAGGATGGCAATTGTCA
TTAGAAGACCAAAGAACGCCTAATTTGATA---------
>ALA27398_Lepeophtheirus_salmonis_Glp1_v2
AAAGAAATCCAATTCGAGAATGACCATCCTTGTTAT---ATTCCTACCCGTCCTCTAAGA
GAATTTTTTTCAGAGTTTATAGGAACTTTGATCTTCATTACAATCGGTATAGGAACTGTA
GCTCAAGTTACACTAAGTGGCATTCTCAAGAGT------CCTGCAGGCACATTTATATCT
ACAAATTGGGGGTTTGGATTCGCTATTATGATTGGAATGATGGTGTCAGGTAAAATATCT
GGAGGTCATATGAATCCAGCTGTGACAATTGCCTTTGCTATCTTTGGTAAACTAAAACCA
TGGCATAAATGTTTTCATTATATGTTTGCCCAATATTTGGGAGCCTTTTTTGGTTCGGCA
CTCGTTTATATTACTTACTATGAAGCTTTTCAAGAAGCC------GGT------------
ATC------------------------------AACATTGACACTGCAAAAATATTCGCA
ACATACCCT---------------------------------------GCTCCTCATATT
TCGGCTCCAGGAATTGTTCTTGACGAATTTGTTGGAACAGCCCTTCTATTAATGTCAGTG
TGTGCTATAATTGATAAAAAAAATCTGAACATTCCATCCTTTTTCCATCCATTTTTAATT
GGACTCGTGGTATTTGTCATAACAATGAGCTTTAGTTTTAATGCAGGTGCCGCTTTGAAC
CCAGCGAGAGATTTAGGACCTCGATTATTTACAGCAGTTTTTGGCTGGGGACTAGATCCC
TTTTCATATGCC---------AAACAT---TTCTGGATGACACCCATTTTAGCGACGCAT
GCAGGGGCTATAATTGGTGTTTTGATATATCAAACTTTT---ATAGGATGGCAATTGTCA

TTAGAAGACCAAAGAACGCCTAATTTGATA---------
>GAZX01003450_Caligus_rogercresseyi_Glp1
TCAGAGCACTTAAATCGTCTGAAGAAGTACTGTCAC---ATAAGTAGCATACCCATGACA
GAGTTTTTTGCAGAGTTCCTCGGAACTGCTCTACTCATCATCATAGGTGTTGGGACTGTA
GCTCAAGTGACATTGAGCAACAAGATGGACGCT------TCAAATGGAGGGCTTTTATCA
ATCGCTTGGGGATTCGGAATCGGCGTCATGATTGGTGTATTGGTTGCTGGCAAAGCCTCT
GGTGGGCACTTAAACCCAGCAGTGTCCTTGGCTTTTGCTATATTCAGGAAACTAACTCCC
TGGTATAAGTGCCTATACTACATGACAGCTCAGTACTTGGGAGCATTCTTCGGATCGATG
GTCGTATACGCTACATACTACGATGCCTTACACAAAGTT------GGA------------
ATC------------------------------ACCTTCGAGACTTCAAAGATTTTTTCT
ACACATCCC---------------------------------------TCTAATTTCATA
TCCTTATTTGGAGTATTTGTGGACCAAGTCGTATCGACCGCACTTCTACTGATATCAATT
TGTGCTATTACTGACAAGAAGAATCTCAACATCCCTCCATTTATACAGCCTTTTCTCATC
GGATTGGTGGTATTTGCATTGTCTCTGAGCTTTAGCTTCAATTGTGGAGCTGCTTTGAAC
CCTGCAAGAGACTTTGGTCCGAGGATCTTTTCGGCTTTGTTCGGATGGGGATTTGCTCCC
TTCACACATTCG---------CCATAC---TTTTGGATGATCATCATAGGCTCAACACAC
GTCGGAGGAATCCTTGGAGTATTCATTTATCAAATAATT---GTTGGATTTCACCTACGC
TCACGAAAGGAAACCATAGCAAATATGGAAGAA------
>GGQW01003486_Tracheliastes_polycolpus_Glp1
---GAA------CAGATGTTAAGACAAAAATGTCGA---ATTCCTCAACTTTGGATTCGA
GAATTTTTGGCGGAATTTATGGGAACCATGATTCTTTTAATATTAGGGGATGGAAGTGTC
GCACAAGTTGTTCTCAGCGAGTCTTTACAACTT------CCAAAGGGAAGTTTCTTTACA
ATCAACATTGGATATTCTTTAGCCGTCGTTTTTTCCCTATATACATCCGTCAAAATTTCG
GGAGGTCATCTTAATCCGGCTGTATCTCTCTCACTCGCCGTACTTGGTAGAATG---CCA
TTACACAAGTTAGGCCATTATATAATAGCTCAGTATCTTGGAGCCATGATGGGCTCTGTA
TTCGTCGTTCTATTAAATATAGATGCCATCAATGCCGTT------GGATAC---------
ATC------------------------------ACACCAGATACAGCTTCTATTTTAACA
ACATTTCCA------------------------------------CTTAATCCACTACTT
TCGACTCGAGGTCAAATAATTGATACACTCTTTGGAACGGCTTTACTTTTATTTCTCATT
TGTGGTCTAAATGACAATGATGGA---AAT------------------CCCGTTTTGTTT
GGCCTCATTGTTTTGGCTCTAGGAGTTAGTTATGGCTTCAATTGTGGCTATGCTTTAAAT
CCAGCTCGTGATCTTGGTCCTCGAATTGTGGCGACTTTGATGGGATGGAACTTAGAGCCC
TTTACGTATGGT---------AATCAC---TACTGGTTAATACCCTCATTTGTGACGTAT
GTTGGAGCCTTGTTAGGAACATTTTTATACACGATCACA---ATCGGAATTCAATTATCT
GTTAGACATTTTTGTGAG---------------------
>GARW01015498_Eucyclops_serrulatus_Glp1
---GAG------TTTAACAAAATGAAAAAGCTGAAA---ATCAGATCACCCCTAATCCGG
GATTTCCTGGCTGAGTTTGCTGGAACATTTATTCTTTTGATTTTTGGTGAAGGCAGTGTA
GCTCAAACAGTCCTCAGTAAAGGG---------------ACCCATGGCTCCTTTTTCTCA
ATCAACTGGGGATATGGTGTTGGGGTCATGCTTGGAGCATACGTGGCAGGTGGAATCAGC
GGAGCACATTTGAATCCGGCCGTAACTCTATCCATGGCCGTAACTAAGAAGTTT---CCA
TGGAGAAAGGTTCCTGTTTACATGATTGGACAATACCTAGGGGCATTTATGGCGTCAGTC
ATTGTGTACATTGTTTACTATGATGCTCTGCAAGCCTTTACT---GGTGGT---------
AATCTTGAAGTCAGTGGA---------------CCAAATGCAACTGCCGGAATTTGGGCA
ACCTACCCT---------------------------------------GCAGAAGGCATT
AGCAGAGGAGTTGGTTTCATTGATCAGATTGTAGGGACAGCTCTACTACTTTTGTGCGTT
CGTGCCATAACAGACGAAAAGAACATGTCCGTGCCAAAATACCTTATACCAGTCCTGGTT
GGATTTGTGGTCCTCAACATCGGAATAGCATTTGGATACAATGCTGGTTATGCGATCAAT
CCTGCCAGAGATTTTGCACCAAGGTTGTTTACTCTCATGTGTGGCTTTGGAACAAATGTT
TTCACTGAATTC---------AACTAC---TGGTTTTACATTCCGCTCCTTGCTCCACAC
CTTGGAGCCATTTTGGGATCCATTCTATACGAGAGTCTG---ATTGCATTCCATTGGCCG
GACGAGGAGGATGTCGATCTGACACTCCAAAGA------
>LT727939_Oithona_nana_Glp1
---------------------ATG------ATCAGA---ATCAGGTCACAGCCAGCCAGA
GAATTCCTCGCGGAATTGGCAGGAACTTTCATCCTTATCGTATTTGGAGATGCTTCAGTG
GCCCAATCAGTGGTCAGTAAGCAA---------------GCCAATGGCGACTTCTTTTCC
ATCAATTGGGGCTGGGGCGTGGCAGTCATGCTAGGCGTGTTTGTCTCTGGTGGCGTCAGC
GGTGGCCATTTGAACCCCGCGGTCACCTTAGCTATGGCACTCCGCAAAAAACTT---GAG
TGGAAAAAAGTTCCCGTCTATTTCTTGCCCAATACCTTGGAGCTTTCTTAGGAGCTGCC
TTAGTCTACTTTGTTTATTACGCATTACAACACAATTAC------GGAGAT---------
TTTCGAGGCGTAGATGGC---------------GCTAATGCAACTGCCGGAATATTTGCG
ACGTATCCT---------------------------------------ACAGCCGGAATT
AGTACTGGTACCGGTCTGCTGGACCAAATCGTGGGTACAGCTTTGCTCCTGTTTTGTATC
CGCGCCATTACAGACGAATACAACATGAAAGTGCCTAGTTATATGGTCCCAATTGCAGTT
GGATTTGTGGTCTTAAACATCGGTATCTGTTTCGGATACAATTGTGGATACGCTATCAAC
CCTGCGCGAGATTTGGGACCCAGATTGCTCACCGCAGTCGTCGGATACGGAGCGGAAGTA
TTCAGTTCAAAA---------ACGTAC---TGGTTTTGGGTGCCAATTGTAGGTCCGCAT
ATTGGAGCCATAATAGGCTGCTTCCTCTACGATTTGCTG---ATAGGTCTTCATTGGCCC

CATAGCGAAGTCACATCTATTGATTTAAAAAGC------
>GGQN01097685_Temora_longicornis_Glp2
---------------------ATGAGA---ATAGCA---CGAAAGTACCCATGGCTAGGA
GAGTCTTTAGCCGAAGTTATTGGTACAATGGTGCTCCTCGCTTTCGGCACCGGCAGTGTA
GCACAGGCTGTTTTATCAAATAGT---------------GTCAACGGAAATATGCTCTCC
ATTAATATAGGCTGGGGCGCAGGAGTCATATTGGGTGTCATGGTTGCAGGACCTATTTCA
GGAGCACATTTGAACCCTGCTGTGTCCGTAGCATTGTGCGCTGTCGGGAAGTTT---CCG
CTCAAGAAGTTGCCGCACTACATTATCTCGCAGTACATCGGGGCTTTCCTTGGTGCAGGC
GTAACGTTGTTGACGTACCATGAGTCTATTCACGGATTCTCA---GGTGAC---------
GGTTTACAGGTATCTGGG---------------ATCAATGGAACGGCTGGGATATTTGTG
ACTTTCCCT------------------------------------GGACGAGAAGGAATA
TCAAACCTCAGCGGAGCAGTTGATCAGGTTGTTGGTACAGCTCTGCTCCTCATCTGCGTC
TGTGCTATAGGCCTACCTAAGAACAAAGCTGTCTCTGGCACGCTTGGTCCCCTCCTGGTT
GGAGTCACCGTCCTCGCCATCGGGATATGCTTCGGGGACAACGCTGGGTACGCAATCAAC
CCAGCGCGTGACCTGGGGCCGCGTATTCTGCTGTCCATGGCGGGCTGGGGCACGGAGGTC
TTCACCGCCAGC---------TACTAT---TGGTTCTGGATCCCTGTAGTTTGTTGCCAT
ATTGGAGGGATCCTTGGTGCTCTCATTCACTTTCTGTGC---GTAGAGAAACTCATGGAC
CAAGAGGATTTCCACTCTGAAGAGAAACAA---------
>GFWY01001247_Acartia_tonsa_Glp2
---------------------ATGAAG---AAGTGG---AAAGTATCTTTGTGGATAAGA
GAATGTCTGGCAGAAATTCTTGGAACAGGAATACTGCTGGCCTTTGGAACTGGAAGTGTA
GCACAGGCTGTACTATCTGGCCGC---------------CAACCGCTTGATATGCTATCC
ATAAACATTGGATGGGCAGCTGGAGTTATTCTTGGCATTCTCTCATCAGCGGCTGTTTCA
GGAGCGCATTTAAATCCTGCAGTAACCATTGCTCAAGCTGTTATGGGCCAGTTT---CCA
TGGAAAAAGGTGCCACACTATCTGTTGTCTCAGTATCTAGGAGCCTTCTTGGGCGCAGGA
GTTACACTTTTAACCTACAGGGAAAATATTCTTGACTTTGCT---GGCGGT---------
GAATTTCAGGTTTCAGGT---------------GAAAATGGAACAGCTGGCATTTTTGTA
ACTTCACCT---------------------------------------AAACCTGGACTA
TCAAATTTTTCTGGAGCTATTGATCAGGTTGTTGGGACAGCATTACTTACTCTGTGTCTT
GGAATTCTAGGAAATCCACGAAATGAGAAGTTGGTTGGCAATCTTGGACCTTTTCTAGTT
GGCCTTATTGTTCTTGCCATTGGGGTTTGCTTTGGTGATAACGCAGGGTATGCCATAAAT
CCAGCTCGGGACCTTGGACCAAGAATCTTGTTACTCATAACTGGCTATGGAACTGAGGCA
TTCTCTGTCAGT---------TATTAC---TGGTTTTGGATTCCTGTTGTTTGTTGTCAT
ATTGGAGGAATACTTGGTTCTCTCATCCATTTTCTTTTT---ATTGATGGATTTCATCAG
AATGATAGC------------------------------
>GBGO01059541_Eurytemora_affinis_Glp2
---------------------ATGAAA---AAGATT---TACTGTGCAGAATGGATGCGA
GAAAGTTTAGCTGAGATAATCGGAACTATGCTGATATTAAGCTTTGGTACTGGAAGTGTG
GCTCAGGCTGTTTTGTCGCATAAA---------------GAAAATGGAAATATGTTCTCA
ATTAATGTTGGATGGGGGGCAGGGGTAGTTCTTGGTATCATGGCTTCAGCTCCTATATCA
GGTGCACACCTGAACCCTGCAGTAACCATTGCCCTAGCAGTTGTAAATAAATTC---CCC
TGGAAAAAAGTTCCCCACTACATGATCTCCCAATATATTGGAGCTTTTATAGGCGCCGCT
ATTACTTTGTTGACTTATAGAGAGGCAATTGTGAACTTTTCC---GGTTCA---------
GAACTTCAGGTGCTTGGG---------------GAGAACGGGACAGCTGGGATCTTTGTA
ACATTTCCC---------------------------------------AATAATGAGATT
TCAAACTTGTCGGGAGCCATTGATCAGATTGTAGGAACAGCACTACTATTGATCTGTGTA
CTGGCCATAGGTTTGCCTGGTAACAATAATGTATCCAAGTCCCTTGGTCCACTGCTGGTT
GGTTTAACAGTTGTTGCTATCGGAGCATGCTTCGGACACAATGCAGGGTATGCTATAAAC
CCAGCACGTGACTTTGCTCCACGCGTTCTTCTTTCTATTTCCGGTTGGGGAACAGAGATC
TATACAGCTAAA---------CAAACA---TGGTGCTGGATTCCTGTGGTGTTTTGCCAT
ATTGGAGGTATCCTAGGAGCTCTTCTATATTACTGGGGA---ATAGAAGTCTGGCAGAAT
AAAAAGTCTAATAACCATGAAGGACCTGACAGC------
>GFWO01123619_Labidocera_madurae_Glp2
---------------------ATGAAG---AGATAC---AAGTGCTCCCCGTGGATCAGA
GAGAGTCTAGCGGAACTTGTTGGAACCATGATTTTGTTGATGTTCGGTACAGGATCTGTA
GCTCAGGCAGTATTATCAAGACGA---------------GTGAACGGGGATATGTTCTCT
ATCAACTTCGGTTGGGGGATCGGAGTAGTGATGGGAGTGATGGCTGCAGGATCTATCTCA
GGAGCGCATCTGAACCCAGCGGTCAGCCTGTCCCTGGCAGTGGTGGGGAAGTTC---CCC
TGGAAGAAGATCCCCCACTACTTCATCAGCCAATACGTCGGGGCTTTCATAGGAGCAGGG
ATTACTCTACTCACTTACAGAGAAAGCATCGTAACTTCTCT------GAT---------
AACCTGGAGGTGCTGGGG---------------GTCAACGGAACAGCTGGAATATTTGTC
ACATTTCCA---------------------------------------AAAGATGGAGTA
TCTAACTTTGGTGGGGCTCTAGATCAGATTGTTGGCACTGCAATTCTCCTACTCTGCATT
TGTGCAATAGGTTACGAGAAGAATGGTGCAGTTTCAAAGACCATGGGACCCTTCCTAGTC
GGTCTGGTGGTGATCGGTATAGGAGCTTGCTTCGGGGATAATGCAGGTTATGCGATCAAT
CCAGCGAGGGATCTATCTCCAAGAATTCTACTATCTATGACTGGCTGGGGAGCTGAGGTG
TTCATCGCCGCA---------AACTAC---TGGTTTTGGATTCCTGTTGTGTTTTGTCAT
ATTGGAGGAATACTTGGAACTCTTATCTTTTATCTTTCT---GTGGAGATTCTCGAATCA

AAGGAGGATGAGTACAATGTACAAATGGAG---------
>GHWV01136355_Pseudodiaptomus_annandalei_Glp2
---------------------ATGAAG---ATAAGG---AGGTACAAGAATGTAATTAGT
GAATTTCTAGGTGAACTTGTGAGCACATGGATCCTTCTCTCCTTCGGCACGGGTGTTGTG
GCTCAGACCGTGTTGTCGCGGAAC---------------AGCGCCGGCTCCATGCTCTCC
ATCAACATCGGCTGGGGCGCCGGCGTCACTCTGGGCATCCTGGCGGCAGGTCAAATCTCA
GGGGCACACATGAACCCTGCAGTGACCGTGGCTCAGGCCGTCTGGAAAAATTTT---CCA
TGGAAAAAGGTACCTCACTATATTGTAGCCCAGTATCTTGGAGCATTCCTAGGAGGTGCT
ACAACTCTCGTTACCTATTGGGAATCTATTCAACTATATTCG---GGAGAT---------
CATTTACAGGTGTCAGGA---------------GAGAATGGAACAGCCATGATCTTCACC
ACGTTCCCG---------------------------------------GCCGAGGGCGTG
TCCAACGTCGGTGGCGCCGTTGACCAGATTGTCGGAACTGGCCTGATGATGGTTTGTTTG
TCAGCAATAGGCTTGCCAAAGAACAGTCACGTGTCTCGAAGTTTGGCGCCATTTCTGGCC
GGGCTGGTCATCCTCACAATTGGTGTTGCTTTTGGATACAACACAGGGTATGCTTTGAAC
CCAGCAAGGGACTTCTCCCCACGTTTCCTGCTGTTCATCTCTGGTTGGGGAAGTCAAGTC
TTCAAGGAGCAC---------GATTAC---TGGTTCTGGATTCCAATTGTAGCAACCCAT
GTTGGAGCAATCTTAGGCATTGGAGTCTATCGACTGTCA---GTGGAGTTTTGGCTTCAC
GAGGAGGACGTAGAGCCGGAGGCAGATGAC---------
>GAXK01179438_Calanus_finmarchicus_Glp3
------------------AAGCCTAAG---CTGTCT---GCTAGAAGTGTGATGCTGAAG
GAGTTCCTTGCTGAGCTGGTTGGTACTTGTGTGCTGCTCATGTTTGGTTGCGGGTGTGTT
GCTCAGTCAGTGCTGAGTGGAGGG---------------GCTAATGGGGGTATGCTGTCC
ATCAATATTGGGTGGGGGCTGGGGGTGCTTGTGGGGGTGCTCATTGCTGGGCCAGTCTCA
GGAGCCCACCTGAACCCTGCAGTAAGCCTCTCCCTTGCCTGTGTGCGTAAGTTC---CCC
CTGACCTCCCTGGCTCACTACCTGGCAGGGCAGTACCTGGGGGCATTCCTTGGCTCCTCC
CTTGTCCTCTTCACCTACAGGGATGCTCTTCACCATTTCTCT---GGTGGC---------
AAGTATGTGGTGGCCGGG---------------GAGAATGCTACTGCGGGTATTTTTGTA
ACTTCTCCC---------------------------------------GCTGAGGGGGTG
ACTAACTTTGGAGGAGCTGTGGATCAGGTTGTTGGGACAGCCCTTCTTCTGCTCTCTGTA
AGTGCCATAAACTTTCATAAGAACTCCAAGATATCCAACAGTCTTGGGCCTCTTCTGGTG
GCTCTTACAGTGGTGGCTATTGGGGTCTGCTTTGGGCATAATGCAGGGTACGCAATCAAT
CCTGCGCGAGACCTTGGTCCTAGGGTGATGATCTCTCTGTGTGGGTGGGGGTGGAAGGCT
TTCAGCAACCAT---------GGGTGG---TGGTGGTGGATACCTGTGGTGTGTTGCCAT
CTGGGAGGCATTGTGGGG--

>GFCI01505892_Pleuromamma_xiphias_Glp3
---------------------------------------ATCCGCGTGGAGTGGGTGAGG
GAGATGCTGGCTGAGATGATCGGGACCTGGGTCCTGATCTGCTTCGGCTGCGGTTGCGTG
GCCCAGTCCGTCCTCAGCGGCGGC---------------AAAAACGGCGAGATGCTCTCC
ATCAATCTGGGCTGGGGGATCGGCGTCACCATGGCCGGGCTCATTTCGGGCAGCGTATCG
GGTGGCCATTTAAATCCAGCCGTGTCTCTTGCCCTGGTGGTTGTGGGTCAAATG---CCT
GTAACCAAACTGGCCCATTACATGGTGGGCCAATATTTGGGAGCATTTGCTGGCGCTGCG
ACGGTATGGGTGGTTTACCTGGACGCCCTCAACCATTACGCG---GGGTCA---------
GATCGATTGGTGGACGGC---------------GAGAATGCCACTGCCGGCATCTTCGCA
ACGTACCCG---------------------------------------GCGGACGGCGTG
TCCAACCTGAGCGGCGGCGTGGACCAAGTCTTGGGCACGGCCCTGTTGCTGCTCTGCCTC
CTCGCCATCAGGGATCCCGCCAACGGCAGCCTCGCCAAGTCCATGGGTCCTGTCCTCGCC
GGACTCGCAGTCACCAACATCGGTATCAGCTTCGGCCACAACTGCGGCTACGCTATAAAC
CCCGCTAGAGACCTTGGTCCGCGGGTGTTCACGGTGATGGCCGGGTGGGGAGGGCGAGTA
TTCACCTCCCAC---------CACGGC---TGGTGGTGGATCCCCCTGGTCTGCAGCCAC
GTCGGTGGCGTCCTCGGTGCCCTCCTCTACACCGTAGGC---ATCAAGATCAACACTAAC
CACGCGAGGATGCAGAGGCACGAAGACCAAGGA------
>GFCI01085950_Pleuromamma_xiphias_Glp2

------TCCATCCTCAGCAACCGC---------------GTGCACGGCGAGATGCTCTCC
ATCAACCTGGGCTGGGGGATCGGCGTCACCTTGGCCGCGCTCATTTCGGGCAGCGTATCC
GGTGGCCATTTAAATCCCGCGGTGTCTCTGGCGCTGGCGGCTGTGGGTCAAATG---TCT
CCATCCAAACTGGCCCATTACATGGCGGGACAATATCTAGGAGCATTTGCAGGCGCCGCT
ACGGTGTGGCTGGTATACCTGGACGCCCTCAACGAATATGCG---GGGTCT---------
GAGCGGCTAGTGGACGGC---------------CCCAACGCCACGGCCGGCATCTTCGCC
ACGTACCCG---------------------------------------GCGGAAGGCGTG
TCCAACCTGGGCGGCGGCGTGGACCAGGTGGTGGGCACGGCCCTGCTGCTCCTCTGTCTC
CTCGCCATCGGAGACCCCGCCAACGGCAGCCTGGCCAAGTCCATGGGGCCCGTCCTGGCA
GGACTCGCCGTCACAAACATCGGCATCTGCTTCGGCCACAACTGCGGCTACGCCATCAAC
CCCGCCAGAGACCTTGGTCCTCGGGTGTTCACGGCGATGGCTGGGTGGGGAGTTCGAGTG
TTCACCGCCCAC---------AACGGG---TGGTGGTGGATCCCCCTGGTCTGCAGCCAC
GTCGGTGGCCTCCTCGGCGCCCTCCTCTACACCCTCGGC---ATCAAGGTCAACAACGAC

CACGCGATGGTGAACAGGCAAGAGGAGAAG---------
>GAWE01083816_Ramulus_artemis_Glp
------------------------TCGAAGTTAAGA---CTTTCTCGCCCATGGCTGCGG
GAGATGTGTGGAGAGTTTATCGGAACATTTTTAATTGTTTTCGTTGGCAACTGCTCGGTT
GCCCAGTCTGTGCTCAGCAGGACG---------------ACCGCTGGAGCATTCCTGTCC
ATAAACATAGCGTATGCGTTCGCTGTGTCCATGGCCATAATTGTCTCTGGAGGACTGTCA
GGAGCTCACGGGAACCCGGCGGTGACTGTCGCGCTAGCAGTGGTCAAGAAGTTC---CCA
TTAAGAAAGGTCCCACACTATCTCGTAGCACAGTACGTCGGGGCATTTGTCGCTGCTGCG
TGCACGTATGTTACGTATTATGAGGCTCTGAGGAACTTTGAT---GGGGGC---------
AACCGCACCGTGGTTGGG---------------GAGCTGGGCACTGCAGGCATATGGGCG
ACGTATCCA---------------------------------------CAGGACTTCCTC
TCTCTCCATAGTGCCTTTGTGGATCAGGTGTTTGGCACGGGCGTGCTCTTGATGGTGGTG
TGCGCGGTGGGAGACCAGAAGAACATGCAGATACACCGCAGCCTACACGCGCTCTACGTG
GGTATGACGGTGGTCGCTCTGGGAGCCAGCCTGGGCTACAACTGTGGCTACCCCTTGAAC
CCGGCCCGCGACCTCGGTCCCAGGACCTTCCTGCTGCTGGCTGGATGGGGGCGCGAGGCA
TTTGTGTACAGG---------------AACTACTGGTGGGTGCCCATTGTAGGACCTCAT
GTCGGTGCTATTGTCGGCAGTGGACTCTACAGCCTTTTTGAAATACAGTTACCAAGGCCA
CATGAAGAT------CAGGTCGAGCTGAAG---------
>GAWD01050986_Medauroidea_extradentata_Glp
------------------------TCGAAGTTACGA---CTTTGTCGTCCGTGGCTGCGA
GAGACGTGTGGAGAGTTTATTGGAACGTTTTTAATTGTTTTCATTGGTAACTGCTCGGTT
GCACAGTCTGTGCTCAGCAAGACG---------------TCTGCAGGAGCATTCCTGTCC
ATTAACATAGCATATGCATTTGGTGTGTCCATGGCCATACTTGTCTCTGGGGGACTGTCT
GGTGCTCATGGAAACCCAGCGGTAACTGTAGCACTAGCAGTCGTCAAGAAGTTC---CCA
ATAAAAAAGGTTCCACACTATCTCGCAGCCCAGTACGTCGGGGCATTCGTGGCTGCTGCA
TGCACGTATTTTACGTATTATGAAGCTCTGACAAACTTTGAT---GGTGGA---------
AACCGCACTGTAATTGGT---------------GAGCTTGGCACTGCGGGCATATGGGCG
ACGTACCCA---------------------------------------CAGGACTTCCTG
TCTCTTCATAGTGCCTTTGTGGATCAGGTGTTCGGCACGGGCGTGCTCTTGATGGTGGTG
TGTGCGGTGGGAGACCCAAAGAACATGCAGGTCCACCGTAGCCTACACGCCCTCTATGTC
GGCATGACGGTGGTTGCTCTGGGAGCTAGTCTGGGCTACAACTGTGGCTACCCCTTGAAC
CCAGCTCGTGACCTTGCTCCTAGGACTTTCCTCTTGCTGGCTGGTTGGGGGCGCGAGGCA
TTTGTGTACAGG---------------AACTATTGGTGGGTGCCGATTGTAGGACCTCAT
GTTGGTGCTATTATCGGCAGTGGACTTTACAGCCTTTTTGAAATACAGTTACCAAGGCCG
CAGGAAGAA------CAGTTAGAACTGAAG---------
>GAWG01068929_Extatosoma_tiaratum_Glp
------------------------TCGAAGTTAAGGATATTGTCTCGTCCATGGATACGT
GAAATGTGCGGGGAATTTATTGGAACATTTCTGGTTGTTTTCATAGGTGATTGTTCAGTT
GCCCAGTCTGTGCTCAGTAAGACA---------------TCTGCTGGGGCCTTCCTGTCC
ATAAACATAGCATATGCATTTGCAGTGACCATGGCCATACTTGTCGCAGGGGGATTATCA
GGTGCTCATGTGAACCCGGCGGTGACCGTAGCGCTAGCAGTTGTTCAGAAGTTC---CCG
AAAAGCAAGGTTGCACACTACCTCATTGCTCAGTACATCGGGGCATTCATTGCTGCAGCT
TGCACATTTTTGATATATTATGAAGCTCTGTGGAATTTTGAT---GGAGGT---------
AACCGCACTGTGGTAGGT---------------GAGCTTGGCACTGCAGGAATATGGACA
ACATATCCA---------------------------------------AAGGACTACCTG
TCCCTCCATGGTGCCTTCTTGGATCAGGTGCTGGCCACGGGTGTGCTGTTGCTTGTGGTG
TGTGCGGTGGGGGATCCGAAAAACATGGAGGTCCACCGCAGCCTGCACGCCCTGTACATT
GGCATGACAGTGGTGGCTCTGGGTGCCAGCCTGGGCTACAATTGTGGCTACCCTCTGAAT
CCTGCCCGTGACCTTGCCCCTAGGACTTTCCTACTACTGGCAGGCTGGGGGCCTGAGGCA
TTTGTGTACAGG---------------AATTATTGGTGGGTGCCAATCTTGGGACCTCAT
GTCGGTGCAATTGTTGGAAGTGGACTGTACAGCCTCTTTGAAGTACAGTTGCAGAGGCCA
CAGGAAGAA------TGTGTGGAACTGAAG---------
>GAWF01020399_Sipyloidea_sipylus_Glp
---------------------------AAGCTAAGT---GTTTCTCGTCCGTGGATGCGA
GAGATGTTCGGAGAGTTTATTGGGACATTTTTACTTGTTTTCATAGGTGACTGCTCGGTC
GCCCAGTGTGTGCTCAGCAAAACA---------------TCTACAGGGACTTTCCTGTCC
ATAAACATAGCTTATGCTCTTGCAGTGACCATGGCCATATTGGTAGCTGGAGGATTGTCA
GGTGCTCATGTCAATCCGGCGGTGACG------CTGGCTGTCGTTCAGAAGTTT---CCA
AAACGCAAGGTTCCATATTACCTCTTAGCTCAGTACTCTGGGGCATTCATCGCTGCGGCA
TGCACATTTTTTACATATTATGAAGCACTGAGGAACTTTGAT---GGAGGA---------
AATCGCACTGTGGTAGGT---------------GATCATGGTACTGCTGGAATATGGGCG
ACATATCCA---------------------------------------CAGGACTTCTTG
TCCCTGCATGCAGCCTTCCTGGATCAGGTTCTGGGTACGAGTGTGTTGTTACTGGTGGTG
TGTGCTATTGGCGACCAGAACAATATGAAGATACACCAGAGCTTACATGCACTCTACATT
GGTATGACAGTCCTAGCTCTCGGTGCTAGTCTTGGCTTCAATTGTGGCTACCCTTTGAAC
CCTGCACGTGACCTTGGTCCTAGGACTTTCCTACTACTGGCCGGCTGGGGACCAGAAGCT
TTTCTGTACAGG---------------AACTACTGGTGGGTGCCAATCATTGGACCTCAT
GTCGGTGCTATTGTTGGATGGGGTGGTTATAATACAGTGGAACCACAGTTACCAAGACCA

GAACAAAAC------CAGTTAGAGTTCAAG---------
>GAUF01080011_Leuctra_sp_Glp
---ATGGACATCGAACTGTTGTCAAGGAAACTCGGC---ACGCAACGAGCATGGCTGCGC
GAGATGATTGCCGAGTTCATCGGAACTTTCCTCATTGTTTTCATGGGTAACTCCTCAGTG
GCAGCATTGAAGTTCAGCAATACT---------------GGTGAGGTCAACGTGACATCA
GTGAATATTGCGTATGGATTTGCAGTTACTTTGGGAGTTCTTGCAGCAGGGGGAAATTCA
GGAGCACATCTGAATCCAGCAGTGACCTTGGCCGTTGCAATCGTTGGAAAGTTC---AAA
TGGAGGAAAATGTTCCATTATTTCGCCGCCCAATATATAGGCGCATTTGTTGCAGCAGTG
TGCACCTTTGCAATATATTACGAGGCGCTGAACTCTTATGAT---GGAGGT---------
ACACACACTGTACAAGGC---------------ACCAACTCGACAGCAGGGATATGGGCG
ACTTATCCC---------------------------------------AAGGATCACCTC
GGTCTCGCAGGAGCATTCGTCGATCAGTTCCTGGGCACCTTCATCCTGCTGCTGGTGATA
TGTGCCATCACGGACCCGCGCAACAACGAGGTGGCCAAGGGCCTGTACGCGCTCTACGTC
GGCATCCTCGTGATCACCATCGGGATAGCACTCGGCTTCAACTGCGGGTATGCCATCAAC
CCTGCCAGAGATCTCTCTCCGCGGCTATTCCTAATGATGGCTGGGTGGGGGACAGAAGCC
TTCACTATTGGA---------------AACTTCTGGTGGGTACCCATCATCGGCCCACAC
TTGGGTGGCATAGCTGGGACATTGTTCTACGTCTTTGCT---ATCGAGATCCACAATCCT
CCAGCATCATCAGAAGATTTGGGTCAGAGAATC------
>GAUX01277991_Ceuthophilus_sp_Glp
AAAATGGATGTAGAGGACATGGCATTTAAATATGGC---ACTTCTCGTGAGTGGTTAAGA
GAAGTTTTGGCAGAATTCATTGGCACATTTTTACTAGTGTTTATAGGGAACTGCTCTGTG
AGTCAAGCCGTGTTTACAGACCCA---------------GTGTTTCCATCGTATCTCGCA
ATCTCCTTTGGCTACGGGTTTGCTGTTACGGTGGGCGTGTTGGTGGCTGGCGGAGCATCA
GGGGCCCATATCAACCCAGCAGTAACGCTCGCACTGGCGGTGACTGGGAAATTC---AAA
TGGAAGAAAGTTCCATATTATTTCGGTGCACAGTACCTGGGTGGATTCGTAGCTGCAGCC
ATGCAATTCCTAATGTACCACGATGCGCTGTTTGACTATGCT---GGAAAC---------
GAGCGGTCGGTGGATGGA---------------TCGAAAGCCACTGCGTCGCTTTATGCC
ACGTACCCC---------------------------------------AAGCCGTACCTC
AGTCTCGAGAACGCATTTCTCGACCAGGTAATGGGGACGCTTATTCTTTTGCTGGTCGTG
TGTGCTATCACTGACAGGAAGAACATGGAGCTGCACCCCAGTCTCTATGCTCTGTACATA
GGCATCTCAGTTGTAGCCATTGGTGTTTCCTTCGGCCTCAACTGTGGCTATGCCATTAAC
CCCGCACGTGACTTGGGACCCAGAATCTTCACCCTTATAGCAGGATGGGGCACAGACACA
TTCATATACAAA---------------TCTTTCTTTTGGGTTCCAATTGTGGGCCCTCAT
GTCGGAGCCATTCTCGGAGCACTGCTGTACGTGTTCGCC---ATTGAAATCCACTTACCT
CCTATAGAACAT---------------------------
>GABA01000698_Zorotypus_gurneyi_Glp
GACCGGAATTTATCAAAATTATTAAGCAAGTTATCG---ACGGAAAGACAATGGCTGAAA
GAATTTTACTCTGAGCTTATTGGAACATTTTTGCTTGTGTTTCTTGGCAACTGCTCCGTA
GCCCAAACAGTTCTCAGCAGGAAA---------------GACGGAGGCTCTTCGCTCTCC
ATTAACTTCGCTTATGGGTTTGCAGTGACTTTGGCCATTTTGGTTGCTGGAAGACCTTCA
GGTGCGCACTTGAATCCAGCAGTTACAACAGCTCTTGCAGTGGTGGGAAAATTT---CCT
TTGGCGGAAGTCTGGCCACTACCTCTGTGCACAGTACTTGGG---ATCGTTTCTATCTGC
GCCACGTATTTCGTCTACTTCGATGCATTGAACTATTTCGAT---TCTGGG---------
AAAAGAAGTGTAGCTGGT---------------GCGAATGGGAGTGCTTCCATCTGGACC
ACTTATCCG---------------------------------------CAAGGGTATTTG
AGTCTGCAGGATGCTTTTCAAGACCAGGTTTTTGGAACCTTCGTTCTGATGTTTGTTGTG
TGTGCTGTCATCGATGAACACAACATGGAGGTCCCAAGGTATTTGCAACCGTTGTACGTG
GGGATGACGGTGGTGGTGATCGGGATGTCCCTGGGTCTCAACTGTGGGTACGCCATCAAC
CCTGCCAGGGACCTCTCACCCAGAATCTTCCTCCTCATTGCAGGTTGGGGATCTCAAGCA
TTCACCTATCGT---------------TATTATTGGTGGGTTCCATTGTTGGGGCCACAC
ATAGGAGCTGTCCTTGGTGCCATCGCTTACTTGATTCTA---ATTGAAATTCATGGACCG
AAGCCAACTACGATTATTCCGGAAGAAGATTTGATCAAA
>GAYA01037781_Zorotypus_caudelli_Glp
GACTGGAATTTATCAAAAATATTAAGCAGGTTATCG---ACAAAAAGACAATGGCTGAAA
GAATTTTTCTCGGAGTTAATAGGAACTTTTCTGCTTGTATTTCTAGGTAACTGCGCTGTA
GCTCAATCAGTACTTAGTCGCAAA---------------GATGCTGGGTCCACGATTTCA
ATTAACTTTGCGTATGGTTTTGCAGTGACATTAGCCGTTTTAGTTGCTGGAAGACCTTCA
GGTGCACATTTAAATCCGGCTGTGACTACAGCGCTCGCAGTGGTGGGTAAATTC---CCT
TGGAAAAAATTGTTGCCATTACCTTCTCGCACAATATTTGGGATCATTTCTCTCGGCTGTT
GCTACATATTTGGTCTATTTTGATGCTCTGAACTTTTTTGAT---TCCGGA---------
AGAAGAAGTGTAGATGGG---------------TTGAATGGCAGTGCATCGATTTGGGCC
ACTTATCCT---------------------------------------CAAGGATATTTG
ACTCTACAGGATGCTTTTCAAGATCAGATATCTGGAACTTTTGTATTGATGTTTGTTGTG
TGTGCGATTATCGACGAACACAATATGGAGATACCTAGATACCTGCAACCTCTATATGTA
GGGATCACAGTGATTGTAATTGGAATGTCTCTCGGTCTTAACTGTGGATACGCCATTAAT
CCTGCTAGAGATCTCTCTCCAAGAATTTTCCTTACGATATCCGGTTGGGGATCCGAAGCT
TTCACATATCGC---------------CATTATTGGTGGGTTCCGTTGCTGGGACCACAT
TTAGGAGCTGTCCTTGGAGCAGTTGCCTACTTGATTCTA---ATTGAAATTCATGGACCG

AAGCCAACTACGATTACTCCTGAAGAAGATTTCATCAAA
>GAUZ01006460_Stenobothrus_lineatus_Glp
------GATATCGAGAAAGTGGGCGAAAAACTTGGC---ACTAAAAGACAGTGGTTAAAG
GAGTTCCTCTCAGAATTTTTGGGGACTTTTATACTAGTGTTTATTGGCAGCTGCTGTATA
GCACAGAGCGTGCTGCGCTGGCAA------------------GGGGCCTGTGCCATAACT
GCCATGACGGGCTACGGCTTCGGTGTCCTGCTGGGTGTTCTCGTCTCAGGA---CCATCT
GGTTCAAATCTGAATCCAGCAGTGACACTAGGATTTTGTGTCATTGGCAAAAAG---CCA
TGGAAGGATTGTCTGCATTATTTCCCAGCCCAGTACCTTGGTGGCTTTGTTGGTGCAGCT
TTTGCATATCTTGTATACTTGGATGCTTTGTACAATTATGAT---AATGGA---------
AATCGTCAGGTAACAGGT---------------CCTCTTGCCACTGCTTATATTTTTACT
ACTTACCCT---------------------------------------GCACCTTATGAA
TCAATTGTTGCAGGATTGGTAAATGAGATGATGGGTACATTTGTATTAATGCTGGTCCTT
TGTGGAATAATAGATGAAAATAGCTGGGGAGTCACTCCAGCTCTGTTACCTGCATACGTT
GGAATACTTATCGTAACACTTATCTATAGTTTCGCTGCAAACACTAGCTGTGCTTTAAAT
CCAGCTCGTGATTTTAGTCCTCGAGTTTTTGTTACTATCGCTGGCTGGGGCTCAGAAGCA
TTTCTGTACCGT---------------GGTTACTGGTGGGTACCAATAGTTGCGCCACAT
CTTGGAGGTATTTTAGGAGCACTATTTTATGCGTTCTTT---ATTGAAATTCACTACAGC
AAAAGTGGATCGAATCTA---------------------
>GASQ01015062_Tetrix_subulata_Glp
------GATCTGGAGAAGGTTTCGGACAAACTCGGC---ACCGAGAGGAAATGGCTCCGC
GAATTTTTCGCGGAAGTAATCGGAACTTGCGTCCTGGTGTTTGTGGGCAACAGCGCCGTG
GCTCAGACAGTCCTCACCTACGGA---------------CAGTCGGGGACAACCTTGTCC
ATCAACCTCGCCTACGGGATCGCCGTCGTACTGGGAGTCCTCATCGCAGGGAAGCCTTCA
GGTGCCCACCTGAACCCCGCGGTGACTCTGGCGATGGCCGTCATCGGTAAGCAC---CCT
TTCAAGGCTGTCCCTCACTACCTGGCCGGACAGTTCATCGGAGCCTTCCTCTCTGCGGTG
CTCACCTTCCTCGTCTACTACGACCTCCTGACCGCGTACAAG---CTGCCG---------
GCCAACGCGCAGCTGGGG---------------GACCTGGCCACCGCCGGCATCTTCGCC
ACCTTCCCC---------------------------------------AACAACGACGTC
ACCCTCCTGGGCTGCTTCGTGGACCAGGTGATAGGAACGTTCGTCCTGCTGCTGCTGGTG
TGCGCCATCGTGGACCCCAAGAACATGGCGGTGCCCAGGAACCTGATCCCGCTGTACGTC
GGCTTGGTGGTGATGGCCATCGGCATGAGCTTGGGCACCAACTGCGGCTACGCCATCAAC
CCCGCCAGGGACCTTTCGCCCAGGGTCTTTGTCACCCTCGCGGGCTGGGACTCCAAGGTC
TTTATGTACAGG---------------ATGTACTTCTGGGTTCCGATCGTGGGTCCGTGT
ATAGGCGGGGTTCTGGGCGCCCTGGTTTACGTGTTCTTC---GTGGAAATACACCACCAA
AAGGAGGAA------------------------------
>GAYV01015247_Liposcelis_bostrychophila_Glp1
------------GTGACATTTAGTCGATATATTGCT---CCTCATCAGCGATGGGTCGCG
GAATTTCTGGCAGAATTTTTGGGAACGTTCTTGTTGGTGTTTTTGGGGAACAGCTCTGTG
GCTGAAAGCGTTTTAACCTATGGG---------------AAAGGAGGAAATTCGGTGACT
ATTTATTTAGGCTACGGGATTGCCTTGCTTTTGGGCATCTTAGTGTCAGTTGGGGTTTCA
GGGGCACATTTGAATCCAGCGGTGACCATAGCAGGGGCCATAGTAAAAAAATTT---GAT
TCGTCCAAAATTTTTCACTATTTGCTTGGCCAATATTTGGGAGCATTTGTGGCTGCGTGT
GCTACTTACTTCGTATACATCGATAGCATACATGCATTCCAC------------------
GAT---CTTCATGGAATA---------------AACAGAACTACAGCTACCATTTTTCAC
ACATTTCCA---------------------------------------AAGGAGCATTTA
AGTGTCGTCGGCTCGTTTGCTGATCAGATATTGGGAACGATGGTTTTGGTTATCGCAATT
TACTCGGCAACAGATAAATTAAACGGCAAAGTTCCGGCTCATTTAATACCACTTTACATT
TCTATAGCTCTCGTAGGCATAGGATGTTCGTTGGGTATTAATCAAGGTTTTGCCTGCAAT
CCTGCACGAGATTTGGCTCCTAGAATTTTCACTTACATTTCGGATTGGGGAAGTGATGTA
TTTTCGGCAAAA---------------ACATATTGGTATGTACCGATTATTGGGCCTCAC
ATAGGAGCGGTCCTAGGTGCTTTCATTTATGTTGTTTTT---ATCGAAAAATTGGAGATT
GCTAAGGAAAAAAAAGTGGACACGCACATTATTGAC---
>GAYV01015248_Liposcelis_bostrychophila_Glp2
------------GTGACATTTAGTCGATATATTGCT---CCTCATCAGCGATGGGTCGCG
GAATTTCTGGCAGAATTTTTGGGAACGTTCTTGTTGGTGTTTTTGGGGAACAGCTCTGTG
GCTGAAAGCGTTTTAACCTATGGG---------------AAAGGAGGAAATTCGGTGACT
ATTTATTTAGGCTACGGGATTGCCTTGCTTTTGGGCATCTTAGTGTCTGTTGGGGTTTCA
GGGGCGCATTTGAATCCAGCGGTGACCATAGCAGGCGCCATAGTAAAAAAATTT---GAT
TCCTCCAAAATCTTTCACTACTTGCTTGGCCAATATTTGGGAGGATTTGTGGCTGCGTGT
GCTACTTACTTTGTATACATGGACAGCATACATGCATTCCAC------------------
GAT---CTTCATGGAATA---------------AACAGAACTACAGCTACCATTTTTCAC
ACATTTCCA---------------------------------------AAGGAGCATTTA
AGTGTCGTCGGCTCGTTTGCTGATCAGATATTGGGAACGATGGTTTTGGTTATCGCAATT
TACTCGGCAACAGATAAATTAAACGGCAAAGTTCCGGCTCATTTAATACCACTTTACATT
TCTATAGCTCTCGTAGGCATAGGATGTTCGTTGGGTATTAATCAAGGTTTTGCCTGCAAT
CCTGCACGAGATTTGGCTCCTAGAATTTTCACTTACATTTCGGATTGGGGAAGTGATGTA
TTTTCGGCAAAA---------------ACATATTGGTATGTACCGATTATTGGGCCTCAC
ATAGGAGCGGTCCTAGGTGCTTTCATTTATGTTGTTTTT---ATCGAAAAATTGGAGATT

GCTAAGGAAAAAAAAGTGGACACGCACATTATTGAC---
>XP_002430403_Pediculus_humanus_Glp
------------GTGACGTTTGGTAAT---TATAAA---TCAATTAAAAACGTAGCCAGA
GAATTTTTAGCCGAATTTATTGGAACGTTTCTTTTAGTCTTTATGGGAAATAGTGCCGTT
GCTCAAACAACTCTAACGGATGGT---------------ACCGGAGGAAATAACATAACA
ATATATTTTGGATACGGTTTCGGTTTATTTTTGGGAGTGTCTGTCGCAGGAGGAATTTCA
GGTGGACATTTAAATCCGGCTGTTTCCGTTGCGGCGGCAATTGTAAAAAAATTT---AAA
TGGCGTAAAATACCACATTATATTCTTGCACAATATTTGGGAGCTTTTCTCGGTGCCTGC
ATAACTTATTTATTATACATTGATTCGTTTAAAGCTTTTCAT------------------
TCG---GATTATGGAATA---------------AATAAAACCACGGCTGGAATTTTCGCG
ACTTATCCA---------------------------------------AAAAATGATTTA
TCCGTTATTGGATCCTTTATCGATCAGATTTTAGGATCCATGATACTTGTTCTCGGTATA
AACGCCATCACGGATCCATTTAAT---AAAATTCCATCGTATTTGGTACCAATTTACGTA
TCATTAACGTTAATTAGCATCGCATGTTCTTTGGGAATAAATCAAGGTTTTGCCATTAAC
CCAGCTCGTGATTTATCACCGAGATTTTTTACTTACATAACCGATTGGGGATCGGATGTA
TTCGATTCTTCC---------------AGTTATTGGTACGTGACAATTATAGGTTCACAC
GTTGGTGCTATTTTAGGAGCTGTGATTTATATGTTAATT---GTTGAAAAAATGAGATTG
ATACGGGAAGAATCAAATGTTACG---ATAATA------
>GAWR01093456_Menopon_gallinae_Glp
------------------CCCTGCGAT---TTTTCA---AATTGTAAGCAATGGTTGGGG
GAATTCGCCGCGGAGTTTCTCGGCACCTTCCTTTTAGTTTTTATGGGGAATAGTGCGGTG
GCCGAGACAGTTCTGACCAACGGG---------------AAAGGAGGAAACCCGGTGACC
GTTTACGTCGGTTATGGAATCGGCTTGTTCCTGGGAATACTCATATCAGGAGGCGTTTCG
GGAGGACATCTGAATCCGGCCGTGACGGTTGCGGGGGCGATTGTCGGAAAGTTT---AAG
CGAGTAAAGGTCTTCCATTACCTCATCGCCCAATATTTAGGCGCGTTTGTCGCAGCAGCC
TTTACTTACCTCGTTTACATTGATTCGTTCAAAGCATTCCAC------------------
GGT---GACCTGCCCATG---------------AACCAGACGACCGCTTCCATATTCGCT
ACATATCCT---------------------------------------AAAAGTGACTTA
TCAGTAATCGGATCCGTAATCGACCAGATTTTGGGTTCCATGGTATTAATCTTCGCAATT
TACGCAGCAACTGATAAGAAGAACACGAACGTTCCTCCACATTTGGTTTCCGTCTATGTG
ACATTGACATTAATTGGCATTGCTTGCTCGTTAGGTATTAATCAAGGATTTGCGGTGAAT
CCCGCCCGAGATTTATCTCCGAGGATTTTTACCTATTTGGCCGGTTGGGGAAATCAAGTT
TTCTCGTCCGAG---------------ACATACTGGTACGTTCCCATCGTTGGTCCGCAC
ATAGGAGCTCTCATTGGTGCTTTACTCTATATATCGCTG---ATTGAAAAGCTAAGAAAA
AATGAAGATGAATTTACCTACTCA---------------
>GAPT01019852_Ectopsocus_briggsi_Glp
------------GTTACTTTTTACAAATACATCAAC---CCTCACCGGGTTTGGGTGAGG
GAGGCAGTTGCTGAGCTACTAGGAACCTTCTTTCTTGTTTTTATGGGCAACAATGCTGTA
GCTGCGAAGGTGATTTCTAATGGG---------------GAAGGCGGTTCATTTGGCACG
ATCTACACTGGATTTGCTTTGGGTGCGTTCCTTGGGGTTCTCATTGCTGCCGGCGTATCA
GGAGCTCACCTAAATCCTGTGGTGACATTAGCTGGTGCTATCGTGAAAAAGGTG---GAC
TGGTGTAAGGTTCCTCATTACATCATCGCTCAGTACCTGGGAGGATTCCTGGCGGCCTGC
TGCACCTACCTGCTGTACATTGATGGATTCAATCAGATGCAT------------------
ATG---GAAGGAGAAGCT---------------AATTTGACAACCGCTGGAATTTTCGCC
ACGTATCCT---------------------------------------CGGGAGGGTCTT
TCTGTGCATGGAGGACTCATTGACCAGATGTTTGGCTCCATGGTTCTTGTAGTTGCTGTC
TATGCGATTCTGGACAAAAAGAACACAAATGTTCCCTTGCAACTTGTGCCTCTCTACGTT
GCGCTTGTCATCTTGGCCATCGGCTGTTCTATTGGTATCAACGAAGGATATGCTATCAAC
CCTGCTCGAGACTTTGGTCCTAGATTGTTCACTTATTTGACCTGCTGGGGAGATCAAGTT
TTCCAGGATGAG---------------ACGTTTTGGTACATTCCCATCGTAGCCACGCAC
ATCGGCGGGATCATTGGAGCTTACTTATACGTGCTGTGT---ATTGAAAGGATGCGGGAG
CTTTCTGCTGAATTCAATATTCAA---ATTGTG------
>GAZG01101517_Eurylophella_sp_Glp
------------GACCGTCTATTCGCTAAGTGTCAC---ACTGAGAGGTCATGGCTTAGA
GAGATACTAGCAGAATTCGTAGGAACATTCATACTCGTGTTTCTGGGTAACAGTGCGGTA
GCACAGGCCGTATTGAGCAGCCAA---------------AGTCATGGACAATTCTTCACT
ATCAACATTGCTTACGGTTTGGCAGTTACACTCGCGGTGCTCGTCGCCGGGGGAGTTTCA
GGTGCCCATTTGAATCCTGCAGTCAGTCTGTCTCTCGCCCTGGTGGGAAAGTTC---CCG
TTCCGTAAGGTTCCACATTACATGTTAGCGCAGTATCTCGGAGCTTTCGTGTCTGCTGCC
TGCACGCTTCTCGTATATCAAGATGCAATGTTTAACTTTGAT---GGTGGT---------
ATTCGTGAAATAATTGGA---------------CCTCGAGGTACTGGAGGAATATTCGCT
ACATATCCT---------------------------------------CAACAATTTGTG
ACACTAGAAGTCGGCCTTGTTGATCAGATCGTGGGTACAGCAATCTTGCTCCTGCTTATA
TGTGCCATCACTGACCCTCGCAATTGTCGCGTTACATCTGGCCTCGCACCTCTTTTCGTC
GGCCTGACTGTCGTTGTGATTGGAATGTCACTGGGCTTCAACTGCGGCTATGCCCTCAAC
CCTGCACGTGACTTAGGACCAAGATTCTTTACAGCGATTGCCGGATGGAGCTTTGACGTG
TTTTATATTGAT---------------GCTTGGGCCTGGGTACCAATTGTTGGACCTCAC
CTTGGAGGATTATTAGGAGCTCTTATTTATTCTCTATGT---ATTGAGGCTCATTGGCCT

GAAGATAGA---CCAATGCACGAAGGCGAGGATACCAAG
>AYNC01028228_Ephemera_danica_Glp
------------GACAAAGCGTTCGCCAAGTGCCAC---ACCGAAAGGACGTGGGTCCGA
GAACTGCTCGCAGAGTTCATAGGCACCTTCATACTTGTGTTCCTTGGCAACAGTTCGGTG
GCACAAGCCGTACTTAGCAGTCAG---------------AGTCATGGACAGTTCTTCACC
ATCAACATTGCTTATGGCTTGGCCGTCACCTTGGCTGTGCTCGTGGCAGGAGGAGTCACA
GGAGCCCACCTGAATCCTGCTGTCACGCTAGCTCTGGCACTCGTTGGAAAATTC---CCT
TTCAGAAAAATACCTCATTACATGATAGCACAATATTTGGGTGCTTTCGTGTCAGCAGCT
GTCACGCTCTTGGTGTACGTAGACGCGATGTACAACTTTGAT---CAAGGT---------
AATCGTCAAATTCTTGGA---------------CCTCAAGGAACAGGTGGAATTTTCGCA
ACATATCCT---------------------------------------CAAACGTTTGTG
TCTCTCGAAGTTGGATTGATTGATCAGGTAGTGGCTACAGCAATTCTATTGTTGCTGATC
TGCGCCATCACTGACCCTCGCAACTGCCGCGTATCCTCAGGCTTGGCTCCCTTGTTAGTG
GGTCTTACAGTCGTCTGCATCGGCATGGCTTTGGGCTTCAACTGCGGCTATGCAATCAAC
CCTGCTCGTGACTTGGGACCTCGTCTCTTTACTGCCATCGCTGGTTGGGGCTTCGAGGTG
TTCATT---GAG---------------GCTTGGTCATGGGTTCCCATTGTTGGTCCCCAT
CTCGGCGCAATCCTCGGTGCTCTCATCTACTCTCTCTGC---ATAGAAGCCCACTGGCCA
GAGGATAGA---CCCGTTCTGGATGGTAAGGATGCCTCA
>GAXA01113250_Isonychia_bicolor_Glp
------------GATAAAGCGTTCGCCAAGTGTCAC---ACAGAGCGATCTTGGATCAGA
GAGATTTTGGCTGAATTTCTCGGTACCTTCATTTTAGTGTTCATTGGCAACAGTGCTGTG
GCACAGGCAGTGCTGAGTAACCAG---------------AGCCATGGTCAGTTCTTCACC
ATCAACATTGCATATGGCCTGGCCGTCACCATGGCAGTGCTTGTCGCAGGTGGCGCCTCT
GGGGGACACCTCAACCCTGCTGTCTCACTGTCACTGGCCCTCGTAGGCAAGTTC---CCA
TTCCGCAAAGTTCCACACTACATGCTGGCTCAGTACTTGGGAGCATTTGTCGCAGCTGCC
GTGACCCTGCTTGTGTACCAAGATGCAATGTTCAACTTTGAC---GGAGGA---------
GTCAGACAAATCATTGGA---------------CCTCAAGGTACTGGAGGCATTTTTGCT
ACATATCCT---------------------------------------CAGGAGTTTGTT
TCGTTGGAAGTTGGACTCACAGATCAGATTGTGGCAACAGCAATCTTGCTGCTGTTGATT
TGTGCCATCACTGATCCTCGCAACTGTGGGGTGTCATCTGGCCTTGCTCCCCTGTTGGTT
GGACTTACTGTGGTGGCCATTGGCATGTCCATGGGCTTCAACTGTGGCTATGCAATCAAC
CCTGCACGAGACTTTGGTCCTCGCTTCTTCACTGCCATTGGAGGCTGGGGATTTGACGTG
TTTGTCATTGAT---------------GACTGGTCATGGGTGCCCATTGTTGGACCTCAC
CTTGGTGGACTTTTGGGCGTGCTCATCTACTCACTCTTC---ATCGAGGCGCACTGGCCA
GAAGACAGG---CCTGTGCTGTCAGACGCAAACCTGAAG
>GATU01072356_Baetis_sp_Glp
------------CAGCGCGCTTTCGCCAAGTGCCAC---ACGGAACGCACCTGGCTCAGG
GAGTTCTTGGCAGAGCTGCTCGGCACCTTCATCCTCGTGTTCATCGGCAACTGTTCGGTG
GCACAGGCGGTGCTGAGCAACCAG---------------AGCCATGGCCAGTTCATCACA
ATCAACTTCGCCTACGGCTTGGCGGTGATGATGGCCGTGCTGGTGGCCGGAGGTGCCTCC
GGCGGCCACCTCAACCCGGCCGTCACCCTGTCGCTGGCGCTCGTCGGCAAGTTT---CCG
TTCGCCAAGATCCTCCACTACATGCTGGCCCAGTACCTCGGCTCTTTCCTCGCAGCCGCC
TGCACCTACATGGTCTACATCGATGCGCTGAACAACTTTGAT---GGAGGT---------
CTGCGACAGATCATTGGC---------------CCACAAGGAACAGCAGGAATTTTTGCC
ACCTATCCG---------------------------------------CAGGAGTTTGTC
TCTCTTGAAGTTGGATTTGCTGACCAGATTATTGGAACTGCGATATTGCTCGTGGTGGTG
TGCGGAATCACCGACCCCAAGAATTGCGGAGTGTCCCCTGGTGTGATCCCCCTGTTCGTG
GGTTTGACGGTGGTGGCCATCGGCATGGCGCTCGGCTTCAACTGCGGCTACGCCATCAAC
CCAGCCAGAGATTTTGCGCCCCGTCTCTTCACGGCGATCGCTGGCTGGACGTTTGACGTG
TTCTACATTGAG---------------CAGTGGTCTTGGGTGCCGTTGCTC---------

>GASO01018965_Tricholepidion_gertschi_Glp
------------GATCGAGTCGCTAGTGCATGTAAA---ACTGACAGAACTTGGCTGCGT
GAAATGCTGGCCGAGTTCATTGGAACCTTCTTGCTTGTGTTCATTGGCGACTGCGCTGTG
GCGCAGTCTGTGCTTAGCAACAAG---------------TCAGCGGGAGAGTTCATATCC
ATCAACTTCGCTTATGGCTTTGCCGTCACGTTGGCTGTCCTGGTGTCTGGAGGAGCTTCG
GGTGGTCATGTTAACCCAGCTGTTTCAGTGGCAATGGCTCTGGTTGGGAAGTTT---GAA
TGGAGGAAAATCGGGCACTACATGGCAGGCCAGTATCTAGGTGCGTTTGTGGGTGCTGCA
GGAGTGCTTCTGGTGTATCAAGATGCTTTGGAGAATTTCGAT---GGTGGT---------
GAAAGACAAGTTGTAGGA---------------AACTTCAGTACAGCTGGTATTTGGGCA
ACATATCCT---------------------------------------AGCGGATTCCTA
TCTACAGGTGTCGGACTTGCAGATCAGATTGTTGGAACCTTTGTTTTGTTGCTGGTTGTG
AGTGCTATTACCGATTTCAAGAATATGAAAGTATCATCTGAGCTAGTACCTCTGTATGTT
GGAATAACTGTTATTACTATTGGCATGTCTCTGGGATTTAACTGTGGCTATGCCATAAAC
CCGGCACGAGATTTGGGTCCCCGTATTTTCACAGCCATAGCAGGATGGGGACCAGGTGTA
TTTACTGAATAT------------GCAAGATTTTGGTTTGTACCAATTCTTGGACCTCAT
ATTGGTGCAGTTCTGGGTGCAATTGCCTATTTATTTATT---GTGGAATTCCATTGGCCA

ACACGGAACAGTGACCTGACAAATGAAATTGTTGTGAAA
>GAYJ01031386_Atelura_formicaria_Glp
------------GAACGAGTGACCGAGAAATGCAGG---ATACAGCGGACGTGGCTGAGA
GAAATGTTGGCTGAATTTATTGGAACGTTGATGCTAGTGCTGATCGGTGACGCATCCGTG
GCCCAGATGGTGTTGAGTGGAGAA---------------ACTGCTGGCCAGTTCGTGTCC
GTCAACTTTGCTTACGGGTTCGCTGCCATGATTGCCATACTCATTGCCGGCGGAGTTTCA
GGAGCGCACATAAACCCTGCCGTGTCTCTGGCCATGGCGATCATCGGCAAGTTC---CCG
TGGAGGAAGGTACCTCACTACATGGTGGCGCAGTACTTGGGAGCGTTCGTAGGATCCGCA
ATCATCTTGCTCGTCTACCAAGATGCTTTGAACGCCTATCTG---GCAGGA---------
---AAGACG------CCA---------------GATTTGGACACTGCTGGAATTTGGGCA
ACCTATCCA---------------------------------------AAGGACTTTTTG
ACTCTTGGAGTTGGATTTGCTGATCAGGTGGTGGGAACGTTTATCCTACTGATAGCAGTT
TGTGCAATCACTGACACAAAGAACATGAAGGTGCCATCTGGCCTGGTTCCTCTGTTCATT
GGCCTCACCATTGCTGCTATTGGGATGTCCCTGGGGTTCAACTGTGGATATGCCATCAAC
CCTGCCAGAGATCTGGGTCCAAGAGTCTTCACATTGCTCTCGCACTGGGGCACAATTACT
TTCACT---------------------GACTGGGCGTGGGTGCCAATAATTGGCCCTCAT
GTGGGTGCGATATTGGGTGCTGTCTGCTACATGCTCCTT---GTAGAACTTCACTGGCCG
AAACACCTAGACTCTGAAGTTACAATGAGTGACATT---
>GAYO01012540_Cordulegaster_boltonii_Glp
------------GACAAACTGTCCGAAAAATGCCGG---TTGAGGCACGTTTGGCTGAGG
GAGTTTTTTGCGGAGTTCTTGGGAACTTTTGCTCTCGTGTTCATCGGGGACTGCTCAGTG
GCTCAGTCAGTTCTAAGTCATACT---------------GCTAATGGAGGTTTTTTGTCC
ATCAACATAGCATGGGGACTGGCCGTTATGATTGGAGTTCTAATTAGCGGTGGGATATCA
GGTGGTCACATTAATCCTGCAGTCACCCTAGCAATGGCGGTCATCGGAAAATTT---TCA
TGGAAAAAAGTACCTCATTACATTTTTGCCCAGTATCTCGGAGCATTTTTTGCATCAGCC
TGCGTCTATTTAGTTTATCTCGATGCGTTGGATGCATTTGAT---GGTGGA---------
GACAGG---GCTGTTCCT---------------------GCCACTGCAGGGATTTTTGCT
ACTTACCCT---------------------------------------GCAGATCATCTT
TCCCTGTTAATTGGTTTCTCTGATCAGGTGATTGGAACTTTTATGCTACTCCTGTCAGTA
TGTGCAATCACAGATAAGAAAAACATGCAAGTTCCTCAAGGCCTGGTTCCTCTACTAGTT
GGACTTGTAGTCGTTGCTATTGGAGCATCACTAGGATTCAACTGCGGTTATGCGATCAAC
CCAGCTCGTGATTTGTCTCCTAGGCTGTTCACTTTAATTGCTGGATGGGGATTGGAGACA
TTCACTTACAAG---------------GTGTGGTGGTGGGTACCAATCATTGGTCCTCAC
GTGGGAGGAATTCTTGGAGCTGCTTTATATGCATTTATG---ATTGAGTTTCACTGGCCA
CCTGAGGGA------------------------------
>APVN01141545_Ladona_fulva_Glp
------------GACAAGGTAGCGGAAAAATGTCGT---CTTCGGAGAACTTGGCTGAGG
GAGTTTTTTGCGGAGCTGCTGGGGACCTTCGTCCTCGTGTTCATCGGAGATTGCTCGGTG
GCCCAATCTGTTCTAAGTCACACA---------------GCTAACGGAGGCTTTTTGTCC
ATCAATTTTGCTTGGGGATTGGCAGTCATGCTTGGAGCACTTATTAGCGGCGGAGTTTCA
GGTGGTCACATAAACCCTGCAGTGACGCTGGCCATGGCAATTATTGGGAAATTT---TCT
TGGAAGAAAGTGCCTCATTATATTCTTGCCCAGTATATAGGAGCCTTCTTTGCATCAGCC
TGTGTCTACCTTATTTATTTAGATGCATTGGATGCATTTGAT---GGTGGT---------
GTAAGG---GCAGTGCCA---------------------GACACTGCTGGAATTTTTGCC
ACTTATCCA---------------------------------------GCTGATCATCTC
TCCCTCCTCATTGGTTTCTGTGACCAGGTGCTGGGAACATTTATACTGCTATTGGCAGTG
TGTGCAATCACAGACAAGAAAAATATGCAAGTTCCTCAGGGTCTAGTTCCTTTGTTGATT
GGACTTGTAGTTGTTGCTATTGGAGCATCCTTGGGATTTAACTGTGGCTATGCTATAAAT
CCAGCTCGAGACCTTGCTCCAAGACTTTTTACATTAGCAGCTGGGTGGGGATTGAAGACA
TTTACGTATAAG---------------GCATGGTGGTGGGTGCCTATTCTTGGTCCCCAT
GTTGGAGCAATACTGGGAGCTGGATTATATGCATTTATG---GTGGAGTTTCATTGGCCC
CCAGAGGGT------------------------------
>GAVW01126922_Epiophlebia_superstes_Glp
------------GATAAACTGTCTGAGAAATGTCGG---TTAAGGCATGTCTGGCTGAGG
GAGTTTTTTGCGGAATTCTTGGGAACTTTCGCGCTCGTGTTCGTCGGGGACAGTTCGGTG
GCTCAGTCAATTTTAAGCCTTACT---------------AATGCTGGTGGTTTCTTGTCC
ATTAACATCGCATGGGGATTGGCTGTAATGATGGGAGTGCTAATTAGCGGTGGCGTATCA
GGTGGTCACATCAATCCTGCAGTTACTCTAGCAATGGCAATAATTGGAAAGTTC---TCT
TGGAAAAAAGTACCTCACTACATTCTTGCCCAGTATCTTGGGGCATTTTTTGGATCAGCC
TGCACCTATATAGTTTATCTAGATGCGCTGGATGCATTTGAT---GGTGGA---------
AACAGGACAGTAGAAGGT---------------GTACGTGCCACAGCTGGGATTTTTGCC
ACCTACCCA---------------------------------------GGAGACCATGTT
TCCCTTTTAATTGGTTTCTGTGATCAGGTACTTGGTACCTTTATGCTACTCCTGGCAGTA
TGTGCAATCACAGACAAGCGCAATATGCAAGTTCCCCAAAGTCTGATTCCTCTACTGGTG
GGACTTGTAGTTGTAGCTATTGGAGCATCATTAGGATTTAATTGTGGTTATGCTATCAAC
CCAGCCCGTGATTTGTCTCCTAGAATATTCACTGCAATTGCCGGTTGGGGATTGGAGACT
TTCAGGTACAAG---------------GCATGGTGGTGGGTACCAATCCTTGGTCCTCAT
GTGGGAGCAATTCTTGGAGCTTCTATATATGCCTTTTTG---GTCGAGTTTCACTGGCCT

CCTGAAGGA------------------------------
>GAYM01097963_Calopteryx_splendens_Glp
------------GACAAGGTGTCGGAGAAGTGCCGC---CTGAAGAGCGTGCCCGTCCGG
GAGTTCTTCGCGGAGTTCCTGGGAACGTTCGTCCTCGTGTTGGTGGGTGACAGCTCGGTG
GCGCAGTCGGTGCTGAGCCACACC---------------TCGGCGGGTGCCTTCCTCTCC
GTCAACGTCGCCTGGGGCGTGGCGGTCATGATGGGCGTCCTCATCAGCGGGGGCGTCTCC
GGAGGGCACATCAACCCGGCCGTCACCTTGGCAATGGCCCTGATCGGCAAGTGC---CCC
TGGAGGAAGGTGCCCTACTACCTGGCGGCGCAGTACCTCGGTGCTTTCCTGGGTGCCGTG
TGCACATACGTCGTCTACTTGGACGCCCTGGACGCCTTCGAC---GGAGGG---------
AACCGGACGGTGGAGGGC---------------CTCCACGCCACGGCCGGCATCTTCGCC
ACGTACCCG-------------------------------------GCGCCGCACCTC
TCCATCCTCATCGGCTTCTGCGATCAGGTTCTGGGCACGTTCCTTCTGCTGCTGTCGGTG
TGCGCCATCACGGACAAGAGGAACATGCAGGTGCCCTCGGGATTGGTGCCCCTGCTCGTG
GGCTTCGTGGTCATCGCCATCGGCGCCGCCCTCGGCTTCAACTGCGGCTACGCCATCAAC
CCCGCCAGGGACCTCTCGCCCAGGATATTCACGGCCGTCGCCGGGTGGGGCCTGGAAACC
TTCACATACAAG---------------GCGTGGTGGTGGGTGCCTATCGTGGGACCGCAC
GTGGGTGCCGTCCTCGGGGCATCGATCTACGCCTTCATG---GTCGAGTTCCACTGGCCG
CCAGAGGGC------------------------------
>ALA27400_Lepeophtheirus_salmonis_Glp3_v1
AATTTATCAGAAGGCATCATGATGACCAGTGTTCCT---AAAGTCCCAAGTCTTGTACGA
CAATTTATGGCAGAACTATTGGGAACATTTTTAGTGGTATACCTTGGGGATGGAGCTATT
GCACAAGTTGTTGTCGGATCACAAACCTCACAGGCTTTTTTTTTCTCTAATTTCCTTTCA
ATTGCACTCGGCTATGGTTTTGCCCTAACAATAGGGATTTTTGTATCAGGTGGAGTATCT
GGCGGTCATCTCAATCCAGCGGTTACTTTAGCAATGGCCTCAATAAAAAAATGT---AAA
TGGAAATGTGTTCCTGTATATATGTTAGCACAATATATTGGAGCATTTTTTGCATCAGCT
ATTCTTTTTGGGGTTTATAGAGATGGAATTATGTTGGTTGAA---GGGGGC---------
---------TACAATAAA---------------ACTGTCAAGACGCAAGGGATTTTTGCT
TCTTATCCAAGT---------------------------------CCAAACATTAATGCT
AGCATTGCTAGTTTAAGCTTTGATCAAATCCTTGCCACAGCTTTGTTGATTATCATTATT
CTTGCAGTCACAGATGGTAAAAATATGAAAGTCATATCAAGTCTTGTTCCTCTATCAATT
GGATTGGGACTTACTGCCATTCATCTAAGTTTCGGAGTTAATGCAGGAACAGCAATAAAC
CCAGCAAGGGATTTTTCTCCGCGTTTATTTACAATAATTGCTGGCTATGGAAATGAACCA
CTTACAACTGGG---------AAT---TACTTTTTCTGGATACCTTGGATTCTTCCTCAT
GTTGGTGCTCTTCTAGGGGCTTTTATTTACGAGTATATG---ATAGAACTTCATCATCCA
ATCGACGAA------------------------------
>ALA27401_Lepeophtheirus_salmonis_Glp3_v2
AATTTATCAGAAGGCATCATGATGACCAGTGTTCCT---AAAGTCCCAAGTCTTGTACGA
CAATTTATGGCAGAACTATTGGGAACATTTTTAGTGGTATACCTTGGGGATGGAGCTATT
GCACAAGTTGTTGTCGGATCACAAACCTCACAGGCTTTTTTTTTCTCTAATTTCCTTTCA
ATTGCACTCGGCTATGGTTTTGCCCTAACAATAGGGATTTTTGTATCAGGTGGAGTATCT
GGCGGTCATCTCAATCCAGCGGTTACTTTAGCAATGGCCTCAATAAAAAAATGT---AAA
TGGAAATGTGTTCCTGTATATATGTTAGCACAATATATTGGAGCATTTTTTGCATCAGCT
ATTCTTTTTGGGGTTTATAGAGATGGAATTATGTTGGTTGAA---GGGGGC---------
---------TACAATAAA---------------ACTGTCAAGACGCAAGGGATTTTTGCT
TCTTATCCAAGT---------------------------------CCAAACATTAATGCT
AGCATTGCTAGTTTAAGCTTTGATCAAATCCTTGCCACAGCTTTGTTGATTATCATTATT
CTTGCAGTCACAGATGGTAAAAATATGAAAGTCATATCAAGTCTTGTTCCTCTATCAATT
GGATTGGGACTTACTGCCATTCATCTAAGTTTCGGAGTTAATGCAGGAACAGCAATAAAC
CCAGCAAGGGATTTTTCTCCGCGTTTATTTACAATAATTGCTGGCTATGGAAATGAACCA
CTTACAACTGGG---------AAT---TACTTTTTCTGGATACCTTGGATTCTTCCTCAT
GTTGGTGCTCTTCTAGGGGCTTTTATTTACGAGTATATG---ATAGAACTTCATCATCCA
ATCGACGAA------------------------------
>ALA27399_Lepeophtheirus_salmonis_Glp2
------------------ATGATATTCGACGTTCCA---CGAGTTCGTAGCCTTATACGA
GTATTCCTTGCTGAATTATTAGGAACATTCCTACTCGTGTATCTCGGAGATGGATCCATT
GCTCAGACGGTTGTAGGAGGAAAATCATCT---CCATTAATTTTCTCCAACTTCCTTTCC
ATTGCTCTTGGCTATGGGTTTGCCCTCACCATTGGGATATTTGTGTCAGGTGGCGTCTCA
GGTGGACATTTGAATCCTGCCGTCACTCTAGCCATGGCATCCATCAAAAAAATG---AAA
TGGAAACTTGTCCCAATTTACATGATAGCACAATATATTGGAGCGTTCCTGGCATCTGGG
ACTTTATTCGGCGTGTATAGAGATGGGATTATATTTGTGGAG---GGGGGC---------
---------TATAACAAA---------------ACGGAGAAAACACAAGGGATCTTTGCT
TCATATCCGAGT---------------------------------GCGGACATTAATGCA
AGTGTTGCGAGTCTCTGCTTTGATCAAATCCTTGCCACAGCATTATTGATTATAATTATT
CTTGCTGTCACAGATGATAAAAATATGAAAATCATATCAAGTCTTGTTCCTATATCAATT
GGATTGGGACTCACTGCCATTCATCTAAGCTTTGGGGTGAACGCTGGAACGGCTATCAAT
CCAGCTAGGGACTTTGCTCCACGATTATTCACGTTCATTGTTGGGTATAGAAGTGGTCCC
TTTGTGGCTGGA---------AAA---CACTTTTTCTGGATCCCTTGGATCCTTCCTCAT
GTGGGTGGAGTTCTTGGAGCCTTGATTTATGAGTACATG---ATTGAGCTTCATCATCCA

ATAGATGTA------------------------------
>GAZX01018815_Caligus_rogercresseyi_Glp2_v1
------------------ATGATCATCGACGTTCCT---CAGCTACCTAATTTGGTCCGA
CAGTTTATGGCGGAGCTTCTAGGGACCTTTCTTCTGGTGTATATGGGAGATGGAGCGATA
GCTCAAGTCGTCGTTGGATCACAGTCCTCCGCATCTTTTTTCTTCTCAAACTTCCTCTCA
ATCGCCATAGGCTATGGTCTGGCCCTAACAATGGGCATACTCGTATCTGGAGGAGTCTCA
GGAGGACACCTCAATCCCGCAGTGACCCTGGCACTTGCCGCCATTCGAAAGTGC---AAG
CTCATCTGCGTCCCCGTCTACATGCTGGCCCAATACATCGGAGCCTTTCTCGCATCTGCC
GTTCTCTTTGGAGTATACCGGGACGGGATTTCCCTTGTAGAA---GGTGGA---------
---------CTTAACAAA---------------ACCGCAAAGACGCAGGGGATATTCGCC
TCCTATCCCACG---------------------------------GGAGCCATACAAGCC
ACGGGGCTCAGTCTGGCCTTTGATCAAATCCTAGCCACTGCACTCCTCGTCATTATTATT
TTGTCGGTGACGGATGGGAGGAACATGAAGGTCATTTCGGGGCTTGTTCCTATCTCCATA
GGGCTTGGACTCACGGCCATTCATCTCAGCTTCGGCATCAACGCTGGAACGGCTATTAAT
CCAGCAAGAGACTTTTCGCCACGGTTGTTTACGTTACTTGCGGGCTATGGGACTGAGCCT
TTCCGGGCCTAC---------AAC---TACTTCTTCTGGATTCCCTTGATCCTTCCCCAC
ATAGGCGGACTCCTGGGAGCCTTCATTTACGAGTACATG---ATTGAGCTCCATCACCCA
AAGGATGAT------------------------------
>GAZX01018816_Caligus_rogercresseyi_Glp2_v2
AAGGAATCTGCAGTCACAATGATCATCGACGTTCCT---CAGCTACCTAATTTGGTCCGA
CAGTTTATGGCGGAGCTTCTAGGGACCTTTCTTCTGGTGTATATGGGAGATGGAGCGATA
GCTCAAGTCGTCGTTGGATCACAGTCCTCCGCATCTTTTTTCTTCTCAAACTTCCTCTCA
ATCGCCATAGGCTATGGTCTGGCCCTAACAATGGGCATACTCGTATCTGGAGGAGTCTCA
GGAGGACACCTCAATCCCGCAGTGACCCTGGCACTTGCCGCCATTCGAAAGTGC---AAG
CTCATCTGCGTCCCCGTCTACATGCTGGCCCAATACATCGGAGCCTTTCTCGCATCTGCC
GTTCTCTTTGGAGTATACCGGGACGGGATTTCCCTTGTAGAA---GGTGGA---------
---------CTTAACAAA---------------ACCGCAAAGACGCAGGGGATATTCGCC
TCCTATCCCACG---------------------------------GGAGCCATACAAGCC
ACGGGGCTCAGTCTGGCCTTTGATCAAATCCTAGCCACTGCACTCCTCGTCATTATTATT
TTGTCGGTGACGGATGGGAGGAACATGAAGGTCATTTCGGGGCTTGTTCCTATCTCCATA
GGGCTTGGACTCACGGCCATTCATCTCAGCTTCGGCATCAACGCTGGAACGGCTATTAAT
CCAGCAAGAGACTTTTCGCCACGGTTGTTTACGTTACTTGCGGGCTATGGGACTGAGCCT
TTCCGGGCCTAC---------AAC---TACTTCTTCTGGATTCCCTTGATCCTTCCCCAC
ATAGGCGGACTCCTGGGAGCCTTCATTTACGAGTACATG---ATTGAGCTCCATCACCCA
AAGGATGAT------------------------------
>GGQW01003900_Tracheliastes_polycolpus_Glp2
AATATTTCAGATACGAATATCATGCATTCGTCATTA---ACGCTTCCTCTTGCTGTTCGT
GAATTTCTCGCAGAATTTTTAGGAACATTTTTATTAGTCTTATTTGGAGACGGTGCAATT
GCACAAGCCATCATAGGTGGAAGTGTCGAAACTCCTGGATTCTTTTCCAGTTTTCAGTCA
ATTGCTTTTGGTTATGGATTTGGACTCATGATTGGAATCTACGTTTCTGGTGGAGTCTCT
GGTGGTCATCTTAATCCCGCAGTTTCCTTTAGTATGGCTCTTGTCAAAAAATTG---TCA
TGGATCAAAGTGCCCATTTACATGGCAGGGCAATATATTGGAGCATTTTTTGCCTCTGCT
GTTCTCTATGGAATTTATGCTGATGGAATTAATTACGTCGAA---GGAGAT---------
---------TTCAATCGT---------------ACATCAAAAACATTTGGAATATTTGCC
TCTTATCCAAAT---------------------------------GAAACTTTAAATCCG
TCTACAGTGACTTTGTCCTTTGGTCAAATGTTATCAACGGCTCTTCTTCTCATTATTATA
CTTGCTGTCACAGATGACCGCAATATGAAAGCTATTTCTGGATTAATTCCATTATCTATT
GGACTAGGTCTAACAGCTATTCATATTAGCTTTGGACTTAATGCTGGCTGTGCTATTAAT
CCAGCAAGAGATTTTTCACCACGATTATTTACTTTAATGGCAGGATGGGGAACAGAGCCC
TTTACAGCAAAC---------ATG---AATTTCTTTTGGATTCCATGGATATTACCTCAT
ATTGGTGGAAGTGTAGGAGCACTTGTATATCAAGCTATG---ATTAAACATCATTTGAGA
GAAAATGAA------------------------------
>GFCI01485726_Pleuromamma_xiphias_Glp5
--CTGGGACAATACTCCAGG
CAGTTTTTGGCAGAGACTTTGGGCACATTTTTGCTTGTGGTGCTTGGGGATGGGTCTGTG
GCTCAGGTGACTCTTGGAAAGAAACTTAATCTGGATGGA---TTTGGGGGTTTACTAAAT
ATAAGCTTTGGATATGGCCTGGCTGTCATGGTTGGCATATTGGCATCAGGTGGTGTGTCA
GGAGGCCATCTCAACCCAGCTGTCACGGTGGCCATGGCTTCACTAAAGAAACTT---AAA
TTAATCCAGGTTCCAATCTACATGGCCGGACAGTACCTGGGTGCCTTCTTAGGAGCAGCA
GTTGTGTTTGGAATATATGCAGATGGAATTAATAAAGTGGGA------------------
---------------AAG---------------TCAAAAGAGACAATGGGAATATTTTCC
TCCTATCCGGGA------------------------------------TTTTCACAGGCA
AGTGAAACCACATTGGCTTTTGACCAAATTCTTGGTACCAGCCTCCTCATCCTCATCATC
CTCGCCGTCACCGATAACAACAACATGGCCATTGTCAACGGAATGTTTCCGCTCTACATC
GGTCTGGGTCTCACAACCATCATTCTCAGTTTTGGTCTGAACGCGGGAGCAGCTATCAAT
CCTGCCAGGGATTTTTCTCCCAGAATCTTCACCCTCATGGCAGGATGGGGGACAAAAACT
TTTGAGGTTAAT---------GGT---TACTTCTTCTGGATCCCCTGGTTGCTTCCCCAC
ATTGGAGGACTGCTTGGAGCTTTCATCTACCACTTTACC---ATCGCGTACCACCACCCA

AAGTCTACCACAGACAGTCTGGAACTCAAG---------
>GFCI01342623_Pleuromamma_xiphias_Glp4
------------------------AAG---TTC------AAAACTCCTAAACTCGTCAGG
GAATTTCTGGCGGAGGTCCTCGGGACTTTCCTTCTCGTCGTGTTCGGAGATGGCGCCATC
ATGCAGGTTGTCCTTGGAAACGCTGGCAGAAGCCACCAATTCTTCGGAGGATTCCTCAAC
ATTTGCCTGGGCTACGGCCTCGGCCTCATGATCGGGATCCTGGCCAGCGGAGGAGTGTCT
GGGGGCCACCTCAACCCGGCAGTTACCCTCTCCATGGCCGCCATCAAGAAGTTG---AAG
CCGATACAGATCCCGATCTACATGGCTGGCCAATACCTGGGAGCATTTCTAGGGGCAGCG
GTTCTTTATGGAATCTACGCCGATGCGATACATATGGTTGAT------------------
---------CCAAACAAG------------------TTAACCAGCCATACTATGTTTGCT
TCCTACCCCAACACATTCGAA------------AATGGTGTGGACACATTTGAAGCGCCC
AGCGTGGCGACACTGGCGATGGACCAAATGTTCGCCACGGCCCTCCTCGTCCTCATCATC
CTCGCCGTCACGGACGAGAACAACATGAAGGTCATCGGGTCCATGGTGCCCCTACTCATC
GGCCTCGGTCTCTGTGCCATTCATATCAGTTTTGGTTTGAATGCCGGTTGTGCCATCAAT
CCCGCGCGGGATCTTTCTCCTCGGCTGTTCACACTTATGGCGGGATACGGCGACAAAACC
TTCAAGCAAGAT---------GAC---TATTTCTTCTGGATTCCGTGGCTTCTGCCTCAC
CTCGGAGGCGTCATCGGAGCCCTGGTCTACCAGTTCATG---GTGGCGCTACACCACAGA
GATGAA---------------------------------
>LS049409_Acartia_tonsa_Glp3
------------------------------ATGCGG---ATATCCAACGTATATGTTCGT
AATTTCCTGTCCGAGTTCCTTGGAACCTTTGCCCTTGTCATCTTTGGAGACGGAGCCGTC
GCACAGGTGGTCCTTGGTTCAAGTTTGGGTGCT---GCTGATTTTGGAGGTTTTCTGAAT
ATTTGCTTCGGCTATGGTTTTGCTCTCATGATTGGAATTCTCATAAGTGGTGGTGTCAGT
GGTGGTCATCTAAACCCAGCTGTCACTGCCTCTATGGTTGTTCTTAGAAAACTG---AAG
CCTGTCATGGCCCCTGTTTACATTGCTGGACAATTTCTTGGTGCATTTTTTGCAGCTCTG
GTCCTGTGGGGAGTTTATGCCGATGCTATTCACATCGCTGAG---AAAGCCATTCTTGTA
GGTTCTGAAGTGAAGTAC---------------ACAATGGCCACTGCTGGTATTTTTGCT
TCTTATCCTATG------------------------------------AATAATGAGATT
AGCACTGCCTGTCTGGCCTTTGACCAGATCCTTGGAACAGCTCTGCTTGTGATCATCATC
CTGGCGGTCACTGATGGCAACAACATGAAGCCCAACTCTGGTCTTATCCCTCTGCTCATT
GGTCTTGGCCTTGCAGCTATTCATCTTAGCTTTGGATTGAATGCTGGAGCTGCCATTAAT
CCTGCCAGAGACCTGGCTCCCAGAATCCTCTCTATCATGGGCGGGTGG------------

>GBGO01056315_Eurytemora_affinis_Glp3
------------------------AAA---CTAGGA---ATATCAAACAGTTATGTCAGA
AATTTCTTGGCTGAGCTACTGGGAACATTTCTCCTGGTAGTTTTCGGGGATGGGGCAATA
GCTCAGGTCACACTAGACAGAGAT---------------------GGCGGCTTCCTGAAC
ATTTGTATTGGATACGGTTTAGCACTCATGATTGGTATCCTGGCCAGTGGCGGGGTCAGT
GGTGGTCACCTAAACCCAGCAGTGACAGTAGCAATGGCTCTTCTTGGAAAAGTT---AAA
CTACTACAGGTCCCTGTTTACATTCTTGGACAGTTCATTGGAGCATTCCTGGCCGCAGCG
GTAGTGCTGGGTGTCTATGCTGATGCTATAAATACTGCAGAT------------------
---------GTTAATTGG---------------AGTTTAACTACTGCTGGAATATTTGCT
TCCTATCCTAGG------------------------------------TTTAATACGGTG
AGTACAATAACCCTGGTGATGGATCAGATCCTTGGAACAGCTCTCCTTCTTATTATTATC
TTGGCTGTAACAGATTCAAGGAACATGAAGGTAAATCCGGCACTGTTTCCTCTTTTTATT
GGACTTGGTCTGACAGCAATTCACATAAGTTTTGGTCTGAATTCTGGCTGTGCAATTAAT
CCCGCAAGAGATTTTGCTCCTCGTCTTCTTTCATTGATCGCTGGCTTCGGAACAGAAACT
TTCACGGTTGGC---------AAC---TATTTCTTCTGGATTCCTCTCCTGATGCCGTTT
CTCGGAGCTGCGATAGGAGCTGCCATTTATCAACTCATG---GTCGCCCTTCATCATACA
GACGAGGAA------------------------------
>GGQN01116754_Temora_longicornis_Glp4
------------------------------AAAGGA---ATAAAAAACAGTTATGTTCGA
AACTTTTTGCTGAGTTCCTCGGGACCTTTGCCCTCGTGGTCTTCGGGGATGGGGCAATA
GCTCAGGTGGTTCTGGGTAAATCTGTGACTGGTTCTGATTTTTTGGTGGCTTTTTGAAT
ATTTGCTTTGGCTATGGCCTCGCACTAATGATTGGCATCCTCATTTCTGGTGGAGTCAGT
GGAGGACATTTGAATCCAGCCGTTTCTCTGGCCATGGTGTGCGTCAAGAAGCTC---AAG
ATTACCCAGTTGCCCGTTTACATGGCTGCCCAGTTTCTGGGGGCCTTCGTTGCCGCGCTG
GTGCTCTGGGGCAACTACGCAGACGCAATCAGAATACACGAA---AACAATCTCTCAGGC
GAGTTTGTGTCTGATTAT---------------TCTGGCGCCACCATGGGAGTGTTCTCC
TCCTTCCCAAAC------------------------------------AATCAAATGACG
AGCCTCACCACCCTCGTCATGGACCAGATCCTCGGCACCGCCCTCCTCGTCATCATCATC
CTTGCTGCCACAGACTCTAAGAACATGAAGTTGGCGTCTGGATTAGTGCCCCTCACGATT
GGTCTGGGGCTCACAGCTATACATATCAGTTTTGGGCTGAACGCTGGATCTGCGGTCAAC
CCGGCCCGGGACTTCTCCCCGCGGTTGCTGACGGCCATGGCGGGCTGGGGAGAACTGCCG
TGCACGGCCTAC---------GAC---TACTGGTTCTGGGTGCCACTGGTGATGCCTTTC
ATCGGCGGCCCCATCGGGGCGGCCATCTACACCCTCCTC---ATTGAGGTCCACCACCAA

CAAGAATTT------------------------------
>GFWO01129170_Labidocera_madurae_Glp4
---------------------------------------TTAAAAAGCAGTTATCTGAGA
AATTTTTGGCTGAGTTCCTTGGAACCTTTGTTCTAGTCGTGTTCGGAGATGGAGCTATT
GCACAGATTGTCTGTGGTGGTCCC---------------TCATTTGGAAACTTTCTCAAC
ATATGCTTTGCCTACGGCCTGGGACTGACAATGGGAATTCTAGTCAGTGGTGGAGTCAGT
GGCGGTCATCTGAACCCAGCTGTTAGTGTGACAATGGCAGCTTTGCGGAAGTTA---GAC
ATAAAACAGCTTCCGGTGTATGTACTCGGACAGTTTCTGGGGGCATTCTTTGCCGCACTT
GTGATCTGGGGAATCTACGCGGACGGAATCTACTTCAGAGAG------------------
GGAGAGACTTTGGATTAT---------------ACAGCCTCTACCGTGGGAATTTTTGCC
TCATATCCAAGC------------------------------------CTCCTCAAGGCC
AACCTCGGCGTGCTCGCGATGGACCAGATCCTGGGCACGGCGTTGCTGCTATTGATCATC
CTCGCCGTGACGGACGAAAGAAACATGAACGTCAACTCCTCCCTCGTCCCCCTCCTCATC
GGGCTCGGCCTCACCGCCATCCACCTCAGTTTCGGGTTTAATGCTGGCTGTGCGATCAAT
CCAGCCAGAGACTTTGCTCCCAGACTTGTCTCTCTCATGGGAGGATGG---CCCAACACA
TTCACGGGTGAG---GGACTGGGA---GGATGGTGGTGGATTCCAACCTTCCTTCCATTC
GTCGGCGGCCTAATCGGCGGTGCTGCCTACCACTTCATG---ATCGAGATTCATCACAAG
GACGAC---------------------------------
>GCIW01018278_Hemidiaptomus_amblyodon_Glp3
GAAACTGATCCCGCCGCAGCCATCTCGGGCTGGTCA---ACATACCGGAGCTTCCTGCGC
ATCTTCCTGGCCGAGATCCTTGGCACCTTCCTCCTCGTCCTCATCGGCGATGCCGCTGTT
GCCCAGGTAGTTCTGGGCGAACAGCTGAAGAAG---ACGATATTCGGAGGCTTCCTCAGC
ATCTCTGTCGGCTTCGGGCTGGCTCTGATGGTTGGCATCCTCGTCGCGGGCGGTGCCAGC
GGAGGCCACCTAAACCCGGCAGTGACGATGGGCTTGGCGGCCGTGGGACGAGTC---CGC
CCCCTCCTGGTCCCCGTGTACTGGGCAGGCCAGTATCTGGGCGCCTTCCTGGCTTCTCTC
GTCCTTTGGGGTGTCTACAGCGAGACAATCCTGCTTGCTGAG------------------
---CCCACCGGAGTGTAC---------------AGCGCCAATACCCGGGGTATATTTGCC
TCCTACCCCATG------------------------------------ATGGACCAATAC
TCGATGGGGGTGCTGGCCGGGGATCAGATCCTCGGCACCGGCCTCCTCCTCATCATCATC
TGCGCGGTGACGGACCCCGGCAACCTCAGGATCAACCCCTCCCTCGTCCCTCTTTACATT
GGGCTCGGGCTGACCTGCATCCATCTGGGCTTCGGCCTGATCGCTGGCTCGGCCATCAAT
CCGGCACGGGACCTAGCTCCCCGCGTCCTCTCCGCCCTGGCCGGCTGG---CCGGGGACC
TTCACAGCCGGG---------AAC---TACTGGTTCTGGATCCCCTCGCTGCTGCCCCAC
CTGGGCGGACTCCTGGGGGCCGGCCTTTACAAGCTAATG---ATTGAGCTGCCCCACCCT
CCGGAGGAACTG---GCATTCCTGCCCCTC---------
>GCIW01018085_Hemidiaptomus_amblyodon_Glp2
---------CCAGGGCTCCTCTCCGCGGAAATGTGC---CAAACCAACAGTTTTTTGCGG
GTCTTTCTGGCCGAGACACTCGGCACCTTCCTCCTTGTCCTCATCGGAGATGCCGCCGTC
GCCCAGGTTGTCCTTGGCGAACAGTTGAACAAG---GCGATATTTGGGGGATTCCTCAGC
ATCTCCGTCGGATTTGGTCTGGCCCTCATGATCGGCATTCTCGTGGCCGGAGGAGCTAGC
GGTGGTCACCTGAACCCGGCGGTAACGCTGGGCCTGGCGGCGGTGGGACGACTC---CGC
CCCATTTTCATCCCCGTGTACTGGGCGGCCCAGTACCTGGGCGCCTTCCTTGCCGCGGCT
GTGGTCTGGGGCATCTACAGCGATGCCATCCACCTGGCCGAG------------------
---CCTGGTGGCCTCTAC---------------AGCAACAGCACGAGGGGGATTTTTGCC
TCCTACCCCATG------------------------------------GATGATCAGTAC
TCCGCTGGGGTGCTGGTTGGCGACCAGATTCTGGGCACAGGCCTGCTTCTCCTCATCATC
TGCGCCGTGACGGACCCCAGCAACCTCAGGATCAACCCCTCCCTTGTCCCTCTCTACATC
GGGCTCGGGCTCACCGCCATCCACCTCGGGTTTGGCCTGATAGCCGGGTCAGCTATCAAC
CCGGCACGTGACCTCTCCCCCCGCCTCCTCACGGCTATGGCGGGCTGG---CCGGGCACC
TTCACGGCTGGG---------AAC---CACTGGTTCTGGATTCCCTGGCTGATGCCTCAC
CTGGGAGGCCTCCTGGGCGCCGGCCTCTACAAGTTCATG---ATTGAGTTCTACCATCCT
AAGGACAGCTTG---AACAGTCTTCCACTG---------
>GCIW01021548_Hemidiaptomus_amblyodon_Glp1
------------------------------ATGCTT---AAACTACACAGCTATGTGCGG
CAGTTCCTGGCTGAGCTGTTGGGAACGTTTGCCCTTGTCATCTTAGGTGATGGTGCTGTA
GCACAGGTTGTATTGGGGCCCGGAGTGAAGGACGCCGTGTTTTTTGGCGGATTCCTGAAC
ATCTGTTTCGGCTACGGACTCGCACTCATGATTGGAATCCTTATCAGCGGAGGCGTTAGT
GGAGGTCACCTGAACCCTGCAGTTACCCTGGCCATGGTGGCTGTGAGAAAGTTG---AAG
TTAATACAGCTCCCTATATACTGGGCTGGCCAATACCTGGGTGCCTTCCTGGCAGCACTT
GTCCTCTGGGGCAACTATGCTGATGCTATCCACCTGACGGAG------------------
---GTAAATGGAGATTAT---------------TCAGCAGCCACTAGAGGGATTTTTGCT
TCATATCCAATG------------------------------------AATGACAAGATC
TCAACGGTGACGCTGGCCATGGACCAGATGCTGGGCACCGCGCTCCTCGTCCTCATCATC
TCTGCCGTCACCGACTCGAGGAACATGAGGGTCAGCTCCTCCCTCGTCCCCCTCTTCATC
GGACTGGGGCTCACCTCCATTCATATAAGCTTTGGCCTGAACGCGGGAGCTGCCATCAAC
CCGGCACGTGACCTGGGGCCACGGGCCCTGTCCGCTCTGGCGGGCTGG---CCCGGCACC
TTCACGACAAAC---------GAG---AGCTGGTTCTGGATTCCCTGGCTGTTGCCCCAC
CTGGGTGGGGTGCTGGGGGCCCTCCTCTACGTCTTCGTC---ATTGAGCTGCACCATCCA

GCTGAGGAGGAC---GTCGTCCTCAAGCAC---------
>GFUD01027877_Neocalanus_flemingeri_Glp7
------------------------AAGATTGGAAAG---ATTACCAACCAGTATGTAAGG
GAGTTCCTGGCGGAGACCTTGGGCACTTTTGCTTTGGTCATCTTTGGAGATGGTGCTGTG
GCTCAGGTGGTGCTGGGCAATGCTGCCCGAGGCAACTCATTTTTCGGAGGATTTCTGAAT
ATCAGTTTTGGGTACGGACTGGCCCTGATGATTGGTATTTGTATTAGTGGTGGAGTGTCC
GGAGGTCATCTCAACCCTGCTGTCACGCTGGCCATGGCGGTTTTCAAGAATCTG---AAA
TGGATCCAGGTACCTATCTACATGGCTGGCCAGTACCTGGGGGCATTCCTTGCAGCTCTG
GTCCTCTGGGGAGAGTATGCTGATGCTATCAAGATTGTGGAG---CTAGGCCAGGATCCT
GCCAACTCTGGCAGCTAC---------------ACCAGCGCCACACACGGGATATTTGCA
TCTTATCCTTTC---TTT---------------------------GGCACCAGCCAGGTC
ACCACCTTGAGCCTGGCCATGGACCAGGTGCTGGGGACAGCCATGCTGCTCATCATCATC
CTGGCGGTGACCGACAACAAGAACATGAAAATCCAGAGTAGCCTGGTACCCCTCACCATA
GGGCTGGGACTGACTGCTATCCATCTCAGTTTTGGCCTTAATGCCGGGTCTGCAATCAAT
CCTGCCAGGGATTTCGCTCCCCGCGTGCTGACTATCCTTGCTGGGTGG---GAGGAGCCG
TTCAAGGCTGCT---------TCC---TCCTGGTTCTGGATACCTTGGCTGCTTCCTCAT
GTGGGAGGAGTGCTGGGAGCTGGACTGTACAAGGTGATG---GTGGGGATGCATACCAAG
GATGGGGAG------------------------------
>GFUD01005420_Neocalanus_flemingeri_Glp6
------------------------AAGATTGGAGAG---GAAACTAACCAATATGTAAGA
GAGTTCCTGGCGGAGACCCTGGGAACTTTTGCTTTGGTCATCTTTGGAGATGGTGCTGTG
GCTCAGGTGGTGCTGGGCAATGCTGCCCGAGGGGACACATTTTTCGGAGGATTTCTAAAT
ATCAGCTTTGGGTACGGACTGGCCCTGATGATTGGTATTTGTATCAGCGGAGGTGTGTCT
GGAGGTCATCTCAACCCTGCCGTCACGCTGGCCATGGCGGTTTTCAAGAATCTG---AAA
TGGATTCAGGTGCCTGTCTACATGGCTGGGCAGTACCTGGGGGCATTCCTTGCAGCTCTA
GTCCTCTGGGGAGAGTATGCTGATGCTATCAAGATGGTGGAG---CTAGGCCAGGATCCA
ACCAACTCTGGCAGCTAC---------------ACCAGCGCCACACACGGGATATTTGCA
TCTTATCCTTTC---TTT---------------------------GACACCAGCCAGGTC
ACCACCTTGAGCCTGGCCATGGACCAGGTGCTGGGGACAGCCATGCTGCTCATCATCATC
CTGGCGGTGACCGACAACAACAACATGAAAATCCAGAGCAGCCTGGTACCCCTCACCATA
GGGCTGGGACTGACTGCTATCCATCTCAGTTTTGGTCTGACAGCCGGGTCTGCCATCAAC
CCTGCCAGAGACTTCTCACCCCGATTACTGACTCTCCTAGCTGGGTGG---GAGCAACCA
TTCAAGGCTGCC---------CAC---TCCTGGTTCTGGATACCTTGGCTGCTCCCTCAT
GTGGGAGGAGTGGTGGGAGCCCTCCTGTACCAGTTTATG---ATTGGTATTCACCACCCT
ATGGAGGAC------------------------------
>GHLB01002221_Neocalanus_flemingeri_Glp5
------------------------AAGATAAATGCA---AACACCAACCAACATGTGAGA
GAGTTTCTAGCTGAGACTTTGGGTACCTTTGCTTTGGTCATCTTTGGTGATGGTGCTGTG
GCCCAGGTGGTGCTGGGCAATGCTGCCAGACAAGACACGTTTTTGGAGGATTTCTGAAC
ATCAGTGTTGGGTACGGGCTGGCTCTGATGATTGGTATTTGTATCAGTGGAGGAGTGTCA
GGGGGGCATCTCAACCCTGCCGTCACGCTAGCCATGGCGGCTATTAAGAATCTG---AAA
CTAATCCAGGTACCTGTCTACATGGCTGGACAGTACCTGGGGGCATTCCTAGCAGCTCTA
GTCCTGTGGGGAGAGTATGCTGATGCTATCAAGATGGTGGAG---GTGGTCGTCTCTCCC
ACCAACTCCAGCAGCTAC---------------ACGAGCACCACACACGGAATATTTGCA
TCTTATCCTTTC---TTT---------------------------GATACAAATAAGGTC
ACCACCCTGAGCCTGGCCATGGACCAGGTGCTGGGGACAGCCATGCTGCTCATCATCATC
CTGGCAGTCACTGACAGCAAGAACATGAAGATAGAGAGCAGCCTGGTACCCCTGACTATT
GGGCTTGGCCTGACTGCTATCCATCTCAGCTTTGGTCTGACAGCCGGGTCTGCCATCAAC
CCTGCCAGAGACTTCTCACCCCGGTTACTGACTCTCCTAGCTGGGTGG---GAGCAACCA
TTCAAGGCTGCC---------CAC---TCCTGGTTCTGGATACCTTGGCTGCTCCCTCAT
GTGGGAGGAGTGGTGGGAGCCCTCCTGTACCAGTTTATG---ATTGGTATTCACCACCCT
ATGGAGGAC------------------------------
>GAXK01155593_Calanus_finmarchicus_Glp6
---------------------GATAGGTTTCGGACT---GCTGGCCACTGCTTCCTGCAG
AAGTTCCTGGCGGAGGCCCTGGGCACCTTCACCCTGGTGCTGCTTGGTGATGGAGCTGTA
GCCCAGGTGGTGCTGGGCAATGCTGCCAGACACGACCAGTTCTTTGGAGGATTCCTCAAC
ATCAGTGTTGGGTATGGCCTGGCACTGATGGTTGGTATCTGCATCAGTGGAGGAGTGTCA
GGGGGACATCTCAACCCTGCTGTCACCCTTGCCATGGCTGTACTGGGCAAGCTC---AAG
CCTGTGCAGGTTCCTGTGTACTGGGCTGGCCAGTACCTTGGGGCTTTTCTAGCAGCAGCA
GTACTTTGGGGAGAGTATGCTGATGCTATCAAGATGGTGGAG------------------
---ACATCTGGCAGTTAC---------------ACCAGCGCTACTCATGGAATATTTGCA
TCTTATCCATTC---TTT---------------------------GGGACAGACCAGGTC
ACCACCTTGAGCCTGGCCATGGACCAGGTGCTGGGGACAGCCATGCTCCTCATCATCATC
CTGGCAGTCACTGACAACAACAACATGAAGATAGAGAGCAGCCTTGTGCCCATCACAATT
GGGCTTGGACTGACCGCTATTCATCTCAGTTTTGGTCTGAATGCCGGGTCTGCCATCAAT
CCTGCAAGGGACTTCTCCCCCCGCTTGCTGTCCCTCTTGGCTGGGTGG---AAGGAGCCA
TTCAAGGCTGCT---------GAC---TCCTGGTTCTGGATCCCTTGGCTGCTCCCCCAT
GTAGGAGGAGTGCTGGGGGCTGGGCTGTACAAGGTCATG---GTGGGCTGCCATATCAAG

GAGCAGGAA------------------------------
>GAXK01155595_Calanus_finmarchicus_Glp5
---------------------GATAGGTTTCGGACT---GCTGGCCACTGCTTCCTGCAG
AAGTTCCTGGCGGAGGCCCTGGGCACCTTCACCCTGGTGCTGCTTGGTGATGGAGCTGTA
GCCCAGGTGGTGCTGGGCAATGCTGCCAGACACGACCAGTTCTTTGGAGGATTCCTCAAC
ATCAGTGTTGGGTATGGCCTGGCACTGATGGTTGGTATCTGCATCAGTGGAGGAGTGTCA
GGGGGACATCTCAACCCTGCTGTCACCCTTGCCATGGCTGTACTGGGCAAGCTC---AAG
CCTGTGCAGGTTCCTGTGTACTGGGCTGGCCAGTACCTTGGGGCTTTTCTAGCAGCAGCA
GTACTTTGGGGAGAGTATGCTGATGCTATCAAGATGGTGGAG------------------
---ACATCTGGCAGTTAC---------------ACCAGCGCTACTCATGGAATATTTGCA
TCTTATCCATTC---TTT--------------------------GGGACAGACCAGGTG
ACCACCTTGAGCCTGGCCTTGGACCAGATGCTGGGGACAGCCATGCTTCTGATCATCATC
CTGGCAGTGACTGACAAGAAGAACATGAAGATAGAGAGCAGCCTGGTCCCCCTGACTATA
GGACTTGGACTGACTGCCATCCACTTGAGTTTTGGTCTGAATGCCGGGTCAGCCATCAAC
CCTGCCAGGGACTTCGCCCCTCGCCTGTTGACCAGCATGGCCGGCTGG---AAACAGCCA
TTTAAGGCTGCT---------CAG---TCCTGGTTCTGGATCCCCTGGTTGCTGCCCCAC
GTGGGCGGGGTGGTGGGGGCTCTCATCTACCAGTTCATG---ATTGGCATACACCATCCT
TTGGAGGAG------------------------------
>GAXK01155600_Calanus_finmarchicus_Glp4
------------------------AAGATTGGGGAG---AATACCAACCAGTATGTAAGA
GAGTTCTTGGCTGAGACTCTGGGTACTTTTGCTCTGGTCATCTTTGGTGATGGGGCTGTG
GCTCAGGTGGTGTTGGGCAATGCTGCCAGAGGGGACACCTTCTTTGGAGGATTTCTGAAC
ATCAGCTTTGGGTACGGGCTTGCCCTGATGATTGGTATTTGTATCAGCGGAGGAGTGTCG
GGAGGCCATCTCAACCCTGCAGTCACGCTAGCCATGGCGGCTGTCAAGAATCTG---AAA
CCACTGCAGGTCCCTGTCTACATGGCTGGTCAGTACCTGGGGGCTTTCCTTGCAGCTCTT
GTGCTGTGGGGAGAGTATGCTGATGCTATCAAGATGGTGGAG------------------
---ACATCTGGCAGTTAC---------------ACCAGCGCTACTCATGGAATATTTGCA
TCTTATCCATTC---TTT--------------------------GGGACAGACCAGGTG
ACCACCTTGAGCCTGGCCTTGGACCAGATGCTGGGGACAGCCATGCTTCTGATCATCATC
CTGGCAGTGACTGACAAGAAGAACATGAAGATAGAGAGCAGCCTGGTCCCCCTGACTATA
GGACTTGGACTGACTGCCATCCACTTGAGTTTTGGTCTGAATGCCGGGTCAGCCATCAAC
CCTGCCAGGGACTTCGCCCCTCGCCTGTTGACCAGCATGGCCGGCTGG---AAACAGCCA
TTTAAGGCTGCT---------CAG---TCCTGGTTCTGGATCCCCTGGTTGCTGCCCCAC
GTGGGCGGGGTGGTGGGGGCTCTCATCTACCAGTTCATG---ATTGGCATACACCATCCT
TTGGAGGAG------------------------------
>GFWO01072462_Labidocera_madurae_Glp3
TCAACCGTCGAAGAGGAAAGAAGAAAAATGCGGTTC---AAGATTCCCAAATTAGCGAGA
TCATTTCTGGCCGAACTTATTGGAACATTTGCTTTGGTTTTGCTTGGTGATGGAGCTGTG
GCCCAGGTCGTCTTAGGACAGGGTATAAAGGGAGATTATTTTTTTGGCGGTTTCCTGAAT
ATTGCTATTGGATATGGGCTAGCTTTGATGGTTGGAATTCTTATCAGTGGTGGAGTTAGT
GGTGGACATTTAAACCCTGCTGTGACCGTTGCTATGGCATGCTTGAAGAAGCTG---CGA
CTGGTGGAGGTTCCAGTATATATGGTCGCACAGTACGTAGGCGCATTCGTCGCAGCTCTT
GTTCTGCGGGGAGCGTACGCTGACGCAATACGTATAACTGAG------------------
---AAAACTACCAACTAC---------------ACTTCAGCTACATACGGAGTATTTGCA
TCCTACCCTTCC------------------------------------TCGAGTGAGATT
GGCATGGTCAGCTTGGCAATGGATCAGATGTTGGGTACAGCTATCCTGGTGCTGATCATC
CTCGCTGTCACGGATCCCAAGAACATGCAGATTTCCTCCTCCCTGGTCCCCCTCCTCATC
GGACTCGGGTTGACCTCCATCCATCTCAGCTTTGGATTGAATGCTGGGTCAGCGATCAAT
CCAGCTCGAGACTTCTCTCCAAGACTGCTCACAGTTCTATCTGGATGGGGAACTCAACCT
TTAACGGCTAAT---------GAC---TACTGGTTCTGGATTCCCTGGCTTCTGCCTCAC
ATCGGAGGTATAGTCGGAGCACTGACCTATGAGATTCTA---ATCGAACTGAACCATCCT
GAAGACGACTTA------------AAACTA---------
>GGQN01050493_Temora_longicornis_Glp3
------------------------------AGGTTT---CACCTCCCAAAGCTTTTTCGG
GAGTTTTTAGCAGAATTTCTGGGAACTTTTGTACTGGTTCTCCTGGGGGATGGATCGGTA
GCCCAGGTTGTCCTGGGAAGTGCTGTAAAGCCAGATCAGTTCTTTGGCGGATTTTTAAAC
ATTGCGTTTGGGTACGGGCTAGCCCTCATGGTCGGAATCTTAGTCAGTGGCGGCGTAAGT
GGCGGTCACTTGAACCCTGCCGTAACTGTGTCCATGGCTTGCATCAAAAAGCTT---AAG
TGGAAGCAAGTCCCTGTGTACATGGCTGGACAATATCTGGGGGCATTCATAGGAGCTCTA
GTTCTGTGGGGAGTCTACGCAGACGCAATCACCATTGCCGAA------------------
---GGAAGCAATGACTAC---------------ACTTCAAAGACCTATGGGATTTTCGCC
TCTTATCCATTC------------------------------------TCTCCTAAGACA
AGCCTGGTCACCCTTGCCATGGACCAGATGTTGGGGACAGCCATGCTAGTTCTCATCATC
CTGGCGGTCACTGACGACCAAAACATGAAAATATCTGCCTCCCTTGTTCCCCTCCTCATT
GGTTTAGGGCTAACATCAATCCATTTAAGTCTTGGTCTGAATGCTGGGTCTGCTATCAAC
CCTGCTCGCGACTTTTCTCCTCGATTGCTCTCTCTGATGGCAGGATGGGGAGAAGAAACA
TTCTCGGTGGACGTTGATTCTGGA---GGTTGGTTCTGGATCCCGTGGTTGTTGCCGCAC
CTTGGGGGTATAGTCGGGGCCCTGGCCTACCAGATAATG---GTCCAGCTTCATCATCCA

GCTGATGATCTG------------AAGCTG---------
>GHWV01089710_Pseudodiaptomus_annandalei_Glp3
---------------------------------------AAACTGCCGAGTTTGGCGCGA
GAGTTCTTCGCCGAGTTCCTCGGAACTTTTGTCCTTGTCCTTATGGGTGATGGCGCCATT
GCACAGGTCAGCTTGGGTAACAGCCTCGGTGCC------AACATGGGCAACTTCCTCAAC
ATCGCCATTGGCTACGGGTTAGCCCTCATGATTGGAATCCTTGTCAGCGGTGGCGTCAGT
GGAGGTCATTTGAACCCAGCCGTCAGCCTGTCCATGACAATCATCGGCAAGCTG---AGG
CCGATTCAGCTGCCGGTGTACATGATTGCCCAGTATCTGGGAGCATTTGTGGCAGCTTTG
GTGCTATGGGGCAACATTTCCAGTGCACTTTATTTGGTTGAG---GAAGGGCTGGACTTC
---AAGGACTGCAACTAC---------------ACTATGGGCACGGCCGGCATCTTTGCC
TCATACCCGATC------------------------------------ACCGAGGAGCTG
AGCTCTGCCAACCTGGTTGTGGATCAGATCCTTGGCACTGCCATGCTTGTACTCATCATC
CTGGCTGTTACAGACTCACAGAACATGAAAGTGTCGTCGGGCCTTGTGCCTCTGCTCATT
GGACTCGGTCTCACCTCCATCCATCTCAGTCTTGGCCTGAACTCAGGGTCAGCGGTCAAC
CCGGCACGTGACTTTTCGCCCCGACTTGTCACTCTCATT---GGCTGGGGCACTGAGACA
TTCACGGCAACT---------GAC---AGTTTCTTCTGGATTCCGTGGATTTTGCCGCAC
GTCGGCGGCGTTCTGGGCGCCGTCGTGTATTATCTCATG---ATCGGCTTGCATCACCGA
GAAAACGACGAC---------------TTT---------
>GBTC01007239_Tigriopus_californicus_Glp6
------------------------------CGAAGT---AAACTTCCCGTTCTGGCTAAG
CAGTTCTTGGCCGAGGCCTTGGGAACCTTTCTCCTTGTTCTTTTCGGAGACGGGGCCATC
GCTCAATATAAAGCCCAATTGGCC---TCAGTA------ACACCCACAGTATTCCCCTCC
GTAGCTTGGGGCTATGGATTGGCGCTCATGATCGGTATTCTGGCTAGTGGGGGAGTGTCC
GGAGGGCACTTAAACCCAGCAGTCACCTTGGCAATGGCGTGCCTCAAGAAGTGT---TCC
TGGAAGCAACTCGTCGTCTACTGGATAGCCCAATATTTGGGTGCTCTGTTGGGGGCGGCT
CTATTATACGGCCTTTATTTGGACGCAATCAATCTACTACCA------------------
------------ATTAGC---------------GATGCTAGCGCAGCTGGGATATTCGCT
ACTTTTCCCGGC------------------------------------TTGAATAATCTG
AACATTGGTACTTTGATTGCCGATCAACTCGTGGGAACTGCTCTTCTTCTGATTATCATC
CTAGCGGTCACAGATTCGAGAAATATGAATGTGCCATCTGGCTTGGTGCCAATTCTCATT
GGTTTGGGTCTGACAGCAATTCATCTCAGTTTCGCATTCAACGCCGGTTGTGCCATCAAT
CCTGCTCGCGATCTCGCACCACGTCTAATTAGTCTCATT------TACAGCTCCTCTGCT
TTTGGTTCCTAC---------GAC---TACTTTTTCTGGATCCCATTGATCATTCCTCAT
ATCGGGGCTGTGTTTGGAGCCGTTATCTATTATTTTGGT---GTCGAGATGCATCATGAG
CCTCATCCACTA---------------GCT---------
>GHUE01053132_Tigriopus_californicus_Glp5
------------------------------CGAAGT---AAACTTCCCGTTCTGGCTAAG
CAGTTCTTGGCCGAGGCCTTGGGAACCTTTCTCCTTGTTCTTTTCGGAGACGGGGCCATC
GCTCAATATAAAGCCCAATTGGCC---TCAGTA------ACACCCACAGTATTCCCCTCC
GTAGCTTGGGGCTATGGATTGGCGCTCATGATCGGTATTCTGGCTAGTGGGGGAGTGTCC
GGAGGGCACTTAAACCCAGCAGTCACCTTGGCAATGGCGTGCCTCAAGAAGTGT---TCC
TGGAAGCAACTCGTCGTCTACTGGCTAGCCCAATATTTGGGTGCTCTGTTGGGTGCGGCT
CTATTATACGGCCTTTATTTGGACGCAATCAATCTACTACCA------------------
------------ATTAGC---------------GATGCTAGCGCAGCTGGAATATTCGCT
ACTTTTCCCGGC------------------------------------TTGAATGATCTT
AACATTGGTACTTTGATTGCCGATCAACTCGTGGGAACTGCTCTTCTTCTGATTATCATC
CTAGCGGTCACGGATTCGAAAAATATGAATGTGCCATCTGGCTTGGTGCCAATTCTCATT
GGTTTGGGTCTGACAGCAATTCATCTCAGTTTCGCATTCAACGCCGGTTGTGCCATCAAT
CCTGCTCGCGATCTCGCGCCACGTCTTATTAGTCTCATT------TACAGCTCCTCTGCT
TTTGCTTCCTAC---------GAC---TACTTTTTCTGGATCCCATTGATCATTCCTCAT
ATCGGGGCTGTGTTTGGAGCCGTTATCTATTATTTTGGT---GTCGAGATGCATCATGAG
CCTAATACAATA---------------GGC---------
>CM017381_Tigriopus_californicus_Glp4
------------------------------CGAAGT---AAACTTCCCGTTCTGGCTAAG
CAGTTCTTGGCAGAGGCCTTGGGAACCTTTCTGCTTGTTCTTTTCGGAGACGGGGCCATC
GCTCAATATAAAGCCCAATTGGCC---TCAGTA------ACACCCACAGTATTCCCCTCA
GTAGCTTGGGGCTATGGATTGGCGCTCATGATCGGTATTCTGGTTAGTGGGGGAGTGTCC
GGAGGGCACTTGAACCCAGCCGTCACCTTGGCAATGGCGTGCCTAAAGAAGTGT---TCT
TGGAAGCAACTCGTTGTCTACTGGCTAGCTCAATATTTGGGTGCTCTGTTGGGAGCGGCA
CTATTATTCGGGCTTTACTTTGATGCAATCAATGAACTACCA------------------
------------ATTAGT---------------GATGCGAGCGCGGCTGGCATTTTCGCT
TCTTTTCCCGGC------------------------------------ATGAGTGATCTT
AACATTGGTACTCTGATTGCTGATCAGCTCGTGGGAACTGCTCTTCTCCTGATTATCATC
CTAGCGGTCACAGACTCGAGAAATATGAATGTGCCATCTGGCTTGGTGACAATTCTCATT
GGTTTGGGTCTGACAGCTATTCATCTCAGTTTTGCATTCAACTCCGGTTGCGCCATTAAT
CCTGCTCGTGATCTCTCGCCGCGTGTTATTAGTCTTCTT------TACATGTCCTCCGCT
TTTGCTGCCTAC---------GAC---TACTTTTTCTGGATCCCATTGATCATTCCTCAT
GTCGGGGCTGTGTTTGGAGCCGTTATTTATTATTTTGGT---GTCGAGATGCATCATGAG

CCTGATACACTA---------------ACG---------
>GCHA01005546_Tigriopus_japonicus_Glp4
------------------------------CGAAGT---AAACTTCCCGTTCTGGCCAAG
CAGTTTCTGGCCGAGACCTTGGGAACCTTTCTCCTCGTTCTCTTCGGAGATGGAGCCATT
GCCCAATACAAAACCCAAATGGGC---TCAGGC------ACACCCACAATTTTTCCCGCG
GTGGCTTGGGGCTATGGATTGGCACTTATGATCGGCATTTTGGCCAGTGGTGGAGTGTCG
GGAGGGCATTTGAACCCGGCTGTCACCTTGGCAATGGCTTGTCTAAAGAAGTGT---TCA
TGGAAGCAACTCCCCGTTTATTGGGTGGCCCAATATTTGGGTGCTTTCTTGGGGGCCGCT
CTCCTGTACGGGATCTATTATGACGGTTTTAATCACACAATT------------------
---------AATTTGACC---------------GATGCCGATGTGGCTGGGGTGTTCGCT
ACGTACCCTGGA------------------------------------AATGGCAAGTTC
AACATCGGAACTTTGATAGCAGATCAGCTCTTAGGCACTGCACTTCTTCTCATCATCATA
TTGGCGGTCACCGATTCGAGAAATATGAACGTACCATCGGGGATGGTACCGATTCTAATT
GGCTTTGGTCTGACAGCAATCCACCTTAGCTTTGCCTTTAATGCAGGTTGTGCGGTCAAT
CCGGCTCGTGATTTAATGCCACGCATCGTGACTCTGATTTTTGGCTTT---CCTGAGTCT
TTTACGTCATAC---------GAT---TACTTTTTCTGGATTCCATGGATTATTCCACAC
TTTGGAGCTGTGTTTGGGGCTGTAATCTATTTCTTTGGT---ATCGAGATGCACCATGAA
CCTACCTTGCTA---------------AGC---------
>JABCAI010000033_Tigriopus_kingsejongensis_Glp4
------------------------------AGGGTG---AAGATCCCCTTGCTGGTCAAG
CAGTTCTTGGCCGAGGCTCTCGGGACATTTCTTCTGGTGGTTTTTGGAGATGGCGCCATC
GCCCAATTCAATTCTCAGGCCAAG---AGCGCC------ACCGACACCAGTTTCATGGCC
GTGGCCATCGGCTACGGGTTCGCTCTCATGATCGGCATCCTGGTGAGTGGAGGCGTGTCC
GGAGGTCATCTCAACCCGGCTGTCACCCTCGCCATGGCGTGTCTGCGGAAATGC---TCC
TGGAAACAATTGCCCGTGTATTGGCTGGCCCAATACTTGGGTGCCTTGCTCGGAGCCGCC
GTGGTCTATGGGATCTATTGGGATGCCTTCAATTTCCTGGAA------------------
------------ATGTCA---------------TACGCCGAAACACGCGGAATATTCGCC
TCCTATCCGGCC------------------------------------AGTGACCAATTG
AGCGTGACCACGTTGATCATGGATCAGCTCCTGGGCACGGGCCTCCTCCTCATCATCATC
CTAGCCGTCACCGACTCCAGGAATATGAAGGTGTCCTCGGGCTTGGTTCCACTCTTGATT
GGCCTGGGATTGGCTGCCATTCACATTAGTCTGGCTTTCAACGCCGGCTGCGCCATCAAT
CCGGCTCGCGATTTCTCCCCGCGATTACTCACTCTGATAGCTGGTTATGGAACTGAAGTG
TTTTCAGCTTTC---------GAC---TACTTCTTTTGGATCCCCCTGATTATGCCGCAC
ATCGGTGGGGTGATTGGAGCGGGACTCTATTACCTCTGC---GTTGAGATGCACCACGAT
ACCTCGGAAGAA------------TTTGCC---------
>GHXK01120398_Platychelipus_littoralis_Glp4
---------------------------------------AAGGCTAATTTGCTTGTAAGA
CAAGGTCTAGCCGAATTCTTCGGTACCTTTTTCCTCGTTCTCATTGGCGACGGAGCAATA
GCGCAGGCAAATTTGAGAGGTGAT---------------GCCGAGAGGACTTTTAGCACG
ATTGCCTTTGGCTACGGATTTGGTTTGATGATTGGCATCTTGGTAAGTGGAGGAGTGTCT
GGTGGCCACTTGAATCCCGCCGTCACCCTGGCCATGGTCTGCCTGAAAAAGTGC---AAG
CCGATTTGTCTTCCCGTTTACATGGCTGCCCAATACCTTGGAGCATTTGCCGGTGCTTCA
GTTCTGTTTGGAATCTATGCCAACGGCCTGAACGATATTGAT------------------
---------CCCTTCTTA---------------ACTAGCAAAACGATGGGCATCTTTGGC
ACCTTTCCCGCT------------------------------------GGTGATCAGATC
AGAGTTGAGACCCTGATAATGGACCAGATACTTGGAACGGCAGTTCTATTAGTCATCATT
TTGGCAGTGACGGATGAAAATAACATGAAGGTAGCCTCCGGTTTGGTGCCTCTCCTGATT
GGTCTCGGACTTACCGCCATTCACTTGTCGCTGGCCTTCAATGCTGGTTGTGGCATAAAC
CCAGCGAGAGATCTTTCTCCGCGAATTTTGAGTTTGGCAGTGGGGTATGGAACTGAACCT
TTCACAGCCTAC---------GAC---TACTTCTTCTGGATTCCTTGGCTAATGCCCCAC
GTTGGAGCTGTCATTGGAGCCTTCGCTTATGTTCTTTTG---ATTTCAATGCATCACGGA
GAGACTGCAGTG---------------------------
>HAHV01124387_Tisbe_holothuriae_Glp4

---------------------GTGACACTGTCCATGTGCTGCCTGAAGAAGTGC---AAG
TGGATGTGTCTGCCCATCTACTGGGCAGCACAATATCTTGGTGCCCTCCTTGCTGCTGCG
ATTCTCTACGGTATCTATGCTGATGGCATCAACAGCAGATTG------------------
------GAT---GGACCT---------------AATCTTGCCAGTGCTGGGATCTTTGCC
TCCTACCCAGCT---------------------------------------GATAAGATC
AGCACAGTGACCCTTGTATTTGACCAGTGCCTGGGAACAGCTGTCCTGCTCATCATCATC
CTGGCCGTGACTGACGGCAAGAACATGAAGGTTGCGTCTGGTATGGTGCCTCTACTCATA
GGACTTGGACTGGCTGTCATTCACATCAGCTTTGCATTTAATGCTGGATGTGCTATTAAT
CCGGCTAGAGATTTCTCACCGCGCTTGTTGACCCTAATGGCTGGCTTTGGCGGCGAGACC
TTCACCGCTTCG---------GAT---TCCTTCTTTTGGATCCCGTGGATCATGCCCCAC
ATTGGCGGCGTCCTCGGTGCGCTCTCCTACTTCTTCCTC---ATTGAGTGGCATCACCCA

GACACAGAGGAT---------------ATG---------
>GCIT01023304_Tisbe_furcata_Glp4
---------------------------------AGC---AAGGTCAAGGACTACGCGCCC
AAGTTCTCCGCCGAGTTCCTCGGCACTTTCATGCTGGTGCTGATCGGCGATGGTTCCATT
GCACAGCTGGTCATGAAGCGGAGT---GGGGCCACCCCTGAGGGGTCAGACTTCCTCAAT
GTGGCACTTGGATATGGCCTTGGACTCATGGTTGGAATCCTGGTTAGTGGTGGGGTCAGC
GGTGGGCACCTTAACCCGGCCGTGACACTCTCAATGTGCTGTCTGAAGAAGTGC---AAG
TGGATCTGCCTGCCAATCTACTGGGCAGCACAATATCTTGGTGCCCTCCTTGCCGCCGCA
ATCCTTTACGGTATCTATGCTGATGGCATCAACTCTAGATTA------------------
------GAT---GGTCCT---------------AACCTTGCCAGTGCTGGGATCTTTGCT
TCCTACCCAGCT---------------------------------------GATAAGATC
AGCACAGTGACCCTTGTGTTTGATCAGTGCCTTGGAACAGCTGTTCTGCTAATCATCATC
CTTGCTGTGACTGATGGCAAGAACATGAAGGTAGCATCTGGCCTGGTGCCTCTGCTCATT
GGACTTGGACTGGCGGTCATTCACATCAGTTTTGCATTTAATGCTGGATGTGCTATTAAT
CCGGCTAGAGATTTCTCACCCCGTTTGTTGACCCTGATGGCCGGCTTTGGTGGCGATACC
TTCACGGCCGCC---------GAC---TCCTTCTTCTGGATCCCCTGGATCATGCCTCAT
ATTGGCGGCGTCCTTGGTGCTCTCTCCTACTTCTTCCTC---ATTGAGTGGCATCACCCA
GACACAGAGGAC---------------ATG---------
>CM017381_Tigriopus_californicus_Glp3a
GGCTTGCCCGGATCGAACACGAGGAAA---ATGAGG---TGCGCCCCGCCGCTTCTTCGG
AAGTTTCTGGCCGAATTCTTGGGAACCTTTCTCCTGGTGATTTTTGGTGACGGAGCCATT
GCCCAACACGTCTTG------GGT---------------CAGAATCAATCATTCCTATCG
ATTTGCTTAGGATATGGTTTGGGCCTCATGATCGGCATCCTTGTCAGTGGAAACGTGTCA
GGAGGTCATCTCAATCCAGCCGTGACTTTGGCCATGAGCTTGCTCCAAAAGTGC---ACT
TACATCTGTGTGCCCGTGTATTGGGCGGCCCAATACTTGGGCGCCTTCGCTGGCGCCACC
GTTCTTTATGGGGTGTATGCAGATGCTATTGAAGCCCAATTG------------------
------------GGCATG---------------AATATGTCTAGTGCTGGGATTTTTGCC
TCGTATCCC---------------------------------------AAGGATGACATC
GGTGTGGTGACACTGATTGTGGGCCAAACTTTGGGAACAGGGATCCTTTTGATCATCATT
TTAGCAGCGACAGACAAGAAGAATATGAATGTGCCACCCGGTCTCTTGCCATTGACTATT
GGATTGGGCCTCACCGCTATTCACATAAGCTTTGCCTATAACGCAGGGTGTGCAATCAAT
CCCGCTCGAGACTTCTCTCCGCGATTACTCACCTATGTGGCTGGTTTCGGG---AACGTT
TTCTCGGCGGAC---------AAT---TATTTCTTTTGGATCCCTTGGATTATGCCCCAT
ATTGGAGCCGTTATTGGAGCCTTGACTTACTACTACTTT---GTGGAAATTCATCACAAA
GATGAAGACGACGAGGAAGATGAAGATTTC---------
>JABCAI010000033_Tigriopus_kingsejongensis_Glp3
------------------------------ATGAAA---CGAGTGCCACCTCTGGTGCAA
AAGTTCCTGGCCGAGCTCCTCGGCACATTCCTCCTCGTGATCTTCGGGGATGGATCCATC
GCCCAGTTTGTGTTG------GGA---------------CAAAACCAGACGTTCCTCTCC
ATTTGCTTCGGCTATGGATTGGGTCTGATGATTGGCATCCTGGTGAGTGGCAACGTGTCG
GGAGGACATCTGAATCCAGCTGTCACCTTGGCCATGGCCTTGCTCCAAAAGTGC---CCT
TACATCTGCGTGCCAGTGTACTGGGCAGCCCAGTATCTGGGTGCATTTCTGGGAGCGAGT
GCCCTCTATGGCGTGTACGCCGATGCCATTGAGGCCAAGGTG------------------
------------GGCATC---------------AATATGACCAGTGCAGGCATCTTTGCC
TCCTACCCC---------------------------------------AAAGACGATATC
GGCATGAGCACCCTGGTTTTGGGCCAAACCCTGGGCACTGCCATCCTCCTAATCATCATT
TTGGCGGCCACCGACAAGAAGAACATGAACGTGTCGTCTGGATTGGTCCCAGTCGTGATT
GGCTTCGGTCTCACCGCTATCCATATTAGCTTTGCCTACAACGCAGGCTGCGCCATCAAT
CCAGCCCGAGACTTCTCTCCGCGCTTGCTGACTTACATTGTCGGTTTTGGC---AACGTG
TTCACCGCCGAC---------GAT---TACTTCTTCTGGATCCCTTGGCTCATGCCTCAC
GCTGGGGCCATCATTGGAGCCCTCACCTACTATTACCTG---ATTGAGCACCATCACCGA
GATGAGGATGAC---------------TTT---------
>GHXK01158759_Platychelipus_littoralis_Glp3_v1
------------------------------ATGAAG---AAGATACCGAATCTGGCCAGA
CAGTTCCTGGCCGAGTTCCTTGGCACCTTCATGCTGGTCATCTTTGGAGACGGGGCAATA
GCTCAGTACAAGTTTCTCGAAGGT---------------CAACAGAGAAACTTTCTCTCA
GTGGCACTGGGGTATGGATTGGCCTTGATGGTTGGCATCCTGGTAAGTGGAAATGTGTCC
GGTGGCCACTTGAATCCCGCCGTCACCCTGTCCATGGCCTGCATCAAGAAGTGC---AAG
TGGATCTGTGTACCTGTCTACTGGATGGCCCAATACCTAGGCGCACTCTGTGGTGCTGCT
GTCCTCTTCGGAGTCTACGCAGACGGCATC---GCGCTAAAT------------------
------------GGGGGT---------------AAAGCTGCCAATGCGGGCATCTTTGCG
TCATACCCCACC------------------------------------AATGAGAATTTG
AGTCCAGTGACCTTGGTTGTGGACCAGGCCTTGGGCACAGGTCTTCTTCTCATCATCATT
TTGGCAGTGACTGACAAAAAGAACATGAACGTCTCCTCTGGATTGATCCCGTTGTTGATT
GGTCTCGGTCTGGCCGCCATTCACATAAGTTTTGCCTACAACGCTGGCTGCGCCATCAAC
CCGGCCAGGGATTTTTCTCCACGACTTTTGACTTTGATTGCTGGTTTTGGAAACAATGTT
TTCGTCAAAAAC---------AAT---TCGTTCTTTTGGATTCCTTGGATCATCCCACAC
ATTGGTGGGGTCTTGGGAGCCTTGACGTACTTCTTCATG---ATTGAAATGCACCACAGG

GACGATTTGGAC--------------------------
>GHXK01158764_Platychelipus_littoralis_Glp3_v2
------------CACTTGCAGTTTGATACAATGAAG---AAGATACCGAATCTGGCCAGA
CAGTTCCTGGCCGAGTTCCTTGGCACCTTCATGCTGGTCATCTTTGGAGACGGGGCAATA
GCTCAGTACAAGTTTCTCGAAGGT---------------CAACAGAGAAACTTTCTCTCA
GTGGCACTGGGGTATGGATTGGCCTTGATGGTTGGCATCCTGGTAAGTGGAAATGTGTCC
GGTGGCCACTTGAATCCCGCCGTCACCCTGTCCATGGCCTGCATCAAGAAGTGC---AAG
TGGATCTGTGTACCTGTCTACTGGATGGCCCAATACCTAGGCGCACTCTGTGGTGCTGCT
GTCCTCTTCGGAGTCTACGCAGACGGCATC---GCGCTAAAT-----------------
------------GGGGGT---------------AAAGCTGCCAATGCGGGCATCTTTGCG
TCATACCCCACC------------------------------------AATGAGAATTTG
AGTCCAGTGACCTTGGTTGTGGACCAGGCCTTGGGCACAGGTCTTCTTCTCATCATCATT
TTGGCAGTGACTGACAAAAAGAACATGAACGTCTCCTCTGGATTGATCCCGTTGTTGATT
GGTCTCGGTCTGGCCGCCATTCACATAAGTTTTGCCTACAACGCTGGCTGCGCCATCAAC
CCGGCCAGGGATTTTTCTCCACGACTTTTGACTTTGATTGCTGGTTTTGGAAACAATGTT
TTCGTCAAAAAC---------AAT---TCGTTCTTTTGGATTCCTTGGATCATCCCACAC
ATTGGTGGGGTCTTGGGAGCCTTGACGTACTTCTTCATG---ATTGAAATGCACCACAGG
GACGATTTGGAC--------------------------
>VIIS01001790_Amphibalanus_amphitrite_GlpB1
CTGCTCCGTCTGCGCCGCCTGCGAAGCGTCTGTCGC---CTTCGTCACCCGCTGCTGAGG
GAGACGCTGGCGGAACTGCTAGGAACCGCCGTACTGGTGTTTATCGGCAACTCTACGATA
GCGAACAACGTGCGGAAT---------------------CCGCTGGTGGCGGCCGTCAAC
GTCCCGCTGGGTTACGTCGCCGCCGTGATGATCGCCGTGTTCGCCACGGGTGGTGTGTCC
GGCGGCCACATTAACCCAGCCGTGACCCTGGCGCTCTGCCTGTCGGGCCGATGT---GAC
CTGACCCGTCTGCTACCCTTCACCGGTGCCCAGTACATTGGCGGCTTCATCGGCGCGCTC
CTGACGCACATTCTATTCTTTGATTGATTCAGTCCGACACC---GATCGA---------
---------------CTC---------------AACCCGACAATATATGGAATATTCTCC
ACCTATCCG---------------------------------------AACAACAGCATA
TCAATCGGAGGAGCCTTCTTTGACCAGGTGCTCGGAACTGCCATCCTGGTGTTCGGCATC
ATGGCTATATCCGACAAACAGAATATGAACGTGCCGAAGCCGCTGCTGCCCGTGGGCGTG
GGTATCGTTCTGTACGGAGCCATCGCCTCGTCGTGCTCCAACACGGGCGCGGCGCTGAAC
CCCGCCAGGGACCTGGGGCCGAGGATCTACTCCTTTGTCATCGGCTACCGA---CAGGTG
TTCAGGGTGGGT---------CAC---CAGTTCTGGTGGATCCCGGTGGTGGCCGGCTAC
ATTGGCGGCCTTCTCGGTGCCTTCGTCTACCTGTTCTGC---ATTGAGCTGCACCACCCG
ACGGCAGAGGGCGACGAGCAGAGGGAGGGGGCGCCACCA
>KY508296_Amphibalanus_improvisus_GlpB1
CTGCTCCGCCTGGACCGCCTGCGTCGCGCCTGTCGC---CTGCGCCATCCTCTGCTGAGG
GAGACGCTGGCCGAACTGCTGGGGACCACGACTCTGGTGTTCATAGGCAACTCGGTGATA
GCCAACAACGTGAAGAAC---------------------CCGCTGGTCGCCTCGGTCAAT
GTGCCTCTGGGATATGTGTCCGCCGTCATCATCTCAGTGTTTGCAACGGGAGGAGTTTCC
GGGGGCCACGTGAACCCAGCAGTAACGCTAGCGCTGTGTCTGGCCGGCCGCTGT---GAT
CTGAACCGACTTCTACCCTTCTGGGGAGCGCAGTATCTCGGAGGGTTTCTCGGTGCACTG
CTGACACATATTCTGTTCTTTGACTTGATTCAGACCGACCCC---GACAGA---------
---------------ATA---------------AACCCGGCAATGTACGGAATATTTGCC
ACATATCCG---------------------------------------AACGACAACATT
TCCATCGGAGGAGCCTTCTCTGACCAGGTGCTGGGCACTGCCATCCTCGTGTTCGGCTGC
ATGGCCATCTCAGACAAGCGGAACATGAACGTGCCCAAGCCGATGCTTCCCGTGGCCGTG
GGTATTGTCCTGTACGGAGCTATCAGCAGTTCGGGATCCAACACAGGCGCAGCGCTGAAC
CCGGCCCGGGATTTCGGACCGAGGATCTACTCCTATATGATCGGATACCAA---CAGGTG
TTCAGCGCGGGC---------CAC---TGTTTCTGGTGGATCCCGGTTGTGGCGTGTTAC
ATCGGTGGTGTTCTCGGCGCATATGTCTACCTCTTCTGC---GTGGAGCTACACCATCCG
GTAGAAGACGGGCAAGAGACGATGACAATCACAAGTGCC
>GHBL01017805_Semibalanus_balanoides_GlpB2
CTGCTCCGCCTGACCCGCCTGAGGCGCGCCTGCCGC---CTGCGTCAGCCCATAGTGCGC
GAGACGCTAGCCGAGCTGGTCGGCACGGCCACACTCGTGTTTATCGGCAACTCGACCATC
GCCAACAACGTGAAGAAC---------------------CCGGTGGCGGCGGCGGTGAAC
ATCCCGCTGGGCTGTGTCGCCGCCGTCACCATCTCTGTGTTCGCCACCGGCGCGGTGTCA
GGTGGTCACATTAACCCGGCTGTGACACTGGCGCTGTGCCTCACGGGTCGCTGT---GAC
CTGAATCGTCTTCTACCCTTCACCGCCGCTCAGTACATCGGTGGCTTCATCGGTGCCTGG
CTGACTCACGGACTTTTCTTCGATCTGATTCAGTCAGATCCA---CAAAGA---------
---------------ATG---------------AATCCTGCCTTGTATGGAATATTCGCC
ACCTATCCG---------------------------------------AATGACAACATG
TCCACCGGAGGAGCCTTCCTTGATCAGGTCCTTGGCACAGCTATCCTTCTGTTTGGCATC
ATGGCTGTCACCGACAGGCGTAACATTAACGTGCCGAAGGCCTTCACCCCGGTCGCCATC
GGCATCGTGCTGTACGGCGCCATCGCCAGTTCCTGCTCCAACACGGGCGCGGCGCTGAAC
CCTGCCCGTGACCTCGGACCGCGGCTCTACTCATACGTCATCGGCTATGAC---CAGGTG

>GHBL01020718_Semibalanus_balanoides_GlpB1
CTGCTGCGCCTGAGCCGCCTGCGCCGCGCCTGCCGC---CTGCGCCGCCCCATAGTGCGC
GAGTCACTAGCCGAGCTGATCGGGACGGCCACGCTCGTGTTTATCACCACCTCAGTGATC
GCCAACAACGTGAGGAGC---------------------CCAGTGACAGCGGCGGTGAAT
GTTCCGCTGGGCTGTGTGGTCGCCGTCACCATAGCTGTGTTCGCCACCGGGGGTGTGTCA
GGAGGTCAAGTTAACCCGGCTGTGACGCTGGCGCTGTGTCTCATGGGTCGATGC---GAC
CTGAACCGCCTTTTGCCCTTCACCACCGCTCAGTTTATCGGCGGCTTTATCGGCGCCTGG
CTGACCCACGGACTCTTTTTCGATTTGATTCAGTCAGATCAA---CAAAGA---------
---------------ATG---------------AATCCCGCCATGTACGGGATATTCGCC
ACCTTTCCG---------------------------------------AATGATAACATA
TCCACCGGAGGCGCCTTCCTTGATCAGGTCCTCGGCACCGCTATTCTTCTGTTCGGTATC
ATGGCCGTCACGGACCAGCGTAACATGAACGTGCCGAAGGCCTGCCTCCCGGTGGCTATC
GGCATCGTCCTGTACAGCGCCATCGCCAGCTCCAGCTCCAACACCGGGGCAGCGCTGAAC
CCGGCCCGCGACCTCGCACCGCGCCTCTACTCCTACGTCATCGGCTATGAC---CAGGTG
TTCAGTGCCGGA---------GAC---CACTTCTGGTGGATCCCCGTGGTGGCGACGTAC
GTGGGCGGCGTACTCGGCGCGCTCGTCTACGTGCTCTGT---ATCGAACTGCACCACCCC
GAGGAGGAG---------------TCGCTGCCACCGTCA
>VIIS01001374_Amphibalanus_amphitrite_GlpA2
ATAATTCCCATGGACCGAGTGCGGCGGGCGTGTCGG---ATACGCCACCCCATTATCCGA
GAGAGCCTGGCCGAGCTGATTGGAACAGCCACGCTGGTGTTTTTCGGCGATGCGGCCATC
GCCAACAACATGTTCATCGGC------------------ACCACCGCGACCGTGGTGAAC
GTGCCTCTTGGGTACGCCACGGCCCTGGCTCTGGCCGTCTACGTGTCTGGCGGCGTGTCA
GGAGGTCACGTAAACCCCGGCGTGACCCTGGGAATGTGCCTGACCGGTCGCTGT---GAC
CTGAACCGGCTGTTGCCCTTTACGATCGCCCAGTTTCTCGGCGGATTCCTCGGCGCTGCT
CTCACTCACGGACTCTTCTTTGATGTTTTCCAAGATGATTCC---CATCAT---------
---------------------------------ACATCCGGGCTATATGCTGTTTTCGCG
ACATACCCC---------------------------------------AATGGATTCATA
TCTACTGGAGGAGCGTTCCTGGACCAGGTAATGGGCACTGCGATGCTGCTGTTTGGGATC
ATGGCCGTCACCGACCGACGCAACATGGAGGTGCCCAAGGGCGGGGTGGGCGCCGCCATT
GGCCTGGTGCTCTTCGGGGTGATCACTGCCAGCGGCTTCAACACGGGCGCCGCGCTCAAC
CCGGCCCGCGACTTTTCGCCGCGCCTCTACAGCTACATCATCGGCTACGAC---AACGTC
TTCAAGGCAGGC---------GAC---CACTTCTTCTGGATCCCGCTGGTGGCCTGCTAC
GTGGGTGCCTTCATCGGCGCCTACCTCTACTTCTTCTGC---ATCGAGGTGCACCATCCG
GACGAGCTCGAGGAGACTCCCGCCGGCCTGGAGCTTCAG
>VIIS01000517_Amphibalanus_amphitrite_GlpA1
ATAATTCCCATGGACCGAGTGCGGCGGGCGTGTCGG---ATACGCCACCCCATTATCCGA
GAGAGCCTGGCCGAGCTGATTGGAACAGCCACGCTGGTGTTTTTCGGCGATGCGGCCATC
GCCAACAACATGATCCTCGGCGGC---------------ACCACCGCGACCGTGGTGAAC
GTGCCTCTTGGGTACGCCACGGCCCTGGCTCTGGCGGTCTACGTGTCGGGCGGCGTGTCA
GGAGGTCACGTAAACCCCGGCGTGACCCTGGGAATGTGCCTGACCGGTCGCTGT---GAC
CTGAACCGGCTGTTGCCCTTTACGATCGCCCAGTTTCTCGGCGGGTTTCTCGGCGCTGCA
CTCACTCACGGACTCTTTTTCGATGTTTTCCAAGATGATTCC---CATCAT---------
---------------------------------ACATCCGAGCTAGTTGCTGTTTTCGCG
ACATACCCC---------------------------------------AATGGATTCATA
TCTACTGGAGGAGCGTTCCTGGACCAGGTAATGGGCACTGCGATGCTGCTGTTTGGGATC
ATGGCCGTCACCGACCGACGCAACATGGAGGTGCCCAAGGGCGGGGTGGGCGCCGCCATT
GGCCTGGTGCTCTTCGGGGTGATCACTGCCAGCGGCTCCAACACGGGCGCAGCGCTCAAC
CCGGCCCGCGACTTTTCGCCGCGCCTCTACAGCTACATCATCGGCTACGAC---AACGTC
TTCAAGGCAGGC---------GAC---CACTTCTTCTGGATCCCGCTGGTGGCCTGCTAC
GTGGGTGCCTTCATCGGCGCCTACCTCTACTTCTTCTGC---ATCGAGGTGCACCATCCG
GACGAGCTCGAGGAGACTCCCGCCGGCCTGGAGCTTCAG
>KY508295_Amphibalanus_improvisus_GlpA1
ATTCTCACCATGAATCGAGTGAGGCGGGCGTGCGGC---ATAAAACACCCGATCGTGCGC
GAGAGCCTGGCTGAGCTGATCGGCACGGCTACACTGGTGTTTTTCGGTGACGCGGCCATC
GCCAACAACATGGGCAGC---------------------AACGTTGGCAGTACCGTGAAC
GTACCGCTGGGGTACGCCACCGCCCTGGCTCTGGCCGTATACGTGTCCGGAGGCGTGTCC
GGCGGGCACGTGAACCCGTGTGTCACCATGGGCATGTGTCTCACGGGCCGCTGT---GAC
CTGAACCGACTGCTACCGTTCACCATTGCCCAGTTCATCGGTGCCTTCCTCGGTGCAGCT
CTCACACACGGACTGTTCTTCGATGTTTTCCAGCTTGACCTC---GCTCGT---------
---------------------------------TCAAAGTCAATGTATGGTGTCTTTGCG
ACGTACCCG---------------------------------------AACCCCATCATA
TCAACTATGGGAGCATTCCTAGATCAGACGATGGGCACGGCGATGCTGCTGTTCGGGATC
ATGGCTGTAACTGACGCGAAGAACATGAACGTGCCTAAGGGAGGCATCCCCGCTTGCATC
GGCCTCGTCCTCTTCGGTGTCATCACGGCCAGTGGCTCTAACACTGGTGCCGCGCTCAAC
CCGGCCAGGGACTTCTCGCCACGGCTCTACAGCTACATCATCGGATACGAC---AATGTG
TTCACGGAAGAC---------GAT---AACTTCTTCTGGATCCCGATCGTGGCCTGCTAT
GTCGGATGCGTCATCGGCGCCTTCCTCTACTTCTTCTGC---ATCGAGGTCCACCATCCG

GCCGAGCTGGACGGCGAGGAACAGGACGTCACGACCAGG
>GHBL01018125_Semibalanus_balanoides_GlpA1
ATTCTCAGCATGGACAAAGTGCGGCGGGCTTGTCGC---CTGAAGAACCCTATCGTGCGC
GAGACTCTTGCAGAACTGATCGGCACTATCACACTGGTGTTCGTCGGGAACTCGGCCATC
GCCAACAACATAGCCAAT---------------------CCAGTGACCGGTTCCGTGAAC
GTTCCACTGGGGTACGCCACAGCTCTGGCACTGGCTGTGTACGTCGCTGGCGGCGTATCC
GGTGGTCATGTCAACCCGACCGTGACGCTGGCCCTGTGTCTGACTGGCCGCTGC---GAC
CTGAATCGGCTGCTGCCCTTCACCGTGGCTCAGTTCATCGGAGGCTTCATCGGGGCGGCA
CTCACACATGGACTCTTCTTCGACTTATTTCAATTGGATCCC---AACCAT---------
---------------GAA---------------GCGGTCGCCTTGTACAAGGTCTTCGCC
ACATACCCG---------------------------------------AATGGCGAGATA
TCAATAGGAGGTGCCTTCCTGGACCAGGTGATGGGCACCGCACTGCTGCTGTTCGGCATC
ATGGCAGTGACGGATCGGCGCAACATGAAGGTGCCGAAGGGCGCGCTGCCGGCCGCCATC
GGCATGGTGCTGTTCGGCGTCATCACGTCCACCAGCTCCAACACGGGCGCGGCGCTCAAC
CCGGCCCGGGACTTCTCACCGCGACTCTACAGCTACATCATCGGCTACCAC---GAGGTG
TTCAAAGCGGGC---------GAC---CACTTCTTTTGGATCCCGATCGTGGCGTGCTAC
ATAGGCTCCGTTGTCGGCGCGTTCCTCTACTTCTTCTGC---ATCGAGGTGCACCACGCG
CCGGAGAACGAGGGGGAGGACCTGCCGGCCGACTACGAA
>GIJW01000734_Octolasmis_warwickii_Glp
---------ATGGAGGCTCTGAGACGCCACTGTCGG---ATCCGGCGGCCAATGGTGCGC
GAGTGTCTGGCCGAGCTGGTTGGCACCATGACATTGGTGTTTATTGGCAACTCTTCCATC
GCCAACAACATGCGCACA---------------------CCCACTGCAAGTCCCATCAAT
GTGCCTCTGGGCTATGCAGCTGGGCTAGCCCTTTCCGTCTATGCAACTGGAGGTGTCTCA
GGTGGTCACCTAAACCCTGCTGTCACCTTGGCACTGTGTCTGACGGGCCGCTGT---CGC
TGGAACCGCTTGCTGCCGTTCACCCTTGTACAGTTCGTGGGCGCCTTCCTGGGCGCTGCA
CTCACGCACGCACTTTTTTTTGACTTGTTCAAGATCGACTGT------------------
---------------------------------TACCCGCAA---------GTATTCGCA
ACCTACCCA---------------------------------------GTACATGGTCTT
TCCACTGGTGGTGGAGCTGTCGATCAGATTCTGGGCACGGCCGTGCTCCTGTACGGTATT
ATGGCGGTGACGGATCGTGCCAACATGGGCTGCCCGCCGGCGCTGACCCCGCTGGCGGTC
GGTCTGACTCTGTTCGCTGTGATCACGTGCTCGGGTAACAACACCGGCGCAGCGGTCAAC
CCGGCCCGTGACCTCAGTCCCCGGCTCTACTCACTGGCCATCGGCTGCGAC---AAGGCC
TTCACAGACGGT---------GAC---CACTTCTGGTGGATCCCGGTGGTCTGTGGATAC
GTGGGTGGCGCGCTGGGCGCAACGTTCTACCTCTTCACC---ATTGAGCTGCACCACCCC
GGTGACGACGGC---------------------------
>GIJX01038132_Glyptelasma_gigas_Glp
---------ATGGAGGCAGCGCGGAGGCACTGCCGG---ATCAGGAGGCCGCTGCTGCGC
GAGTGTCTGGCAGAGCTGGTCGGCACCACCACCCTTGTGTTCATCGGCAACTCGTGCATC
GCCAACAACGTGGCG------------------------TCGCCGGGGAGTCCCATCAAC
GTGCCGCTGGGTTACGGGGCCGCTCTGGCTGTCTCGGTGTACGCCACCGGCGGCGTCTCA
GGTGGGCACCTGAACCCGGCAGTGACGCTGGCGCTGTGCCTGACCGGCCGCTGT---CGC
TGGAACAAACTGCTGCCTTTCACTCTGGTCCAGTTCCTCGGCGCGTTCATCGGCGCCGCT
CTCACGCATGCGCTCTTCTTCGACATGTTCAAGGCGGATAGC------------------
---------------------------------GCGGGGCAGCTGGCGGGTGTGTTCGCC
ACCTACCCGATGACGCGAGGCACC------------------------ACCTTCACCATC
AGCACCGGTGGCGGCGTCGTCGACCAGGTGCTCGGCACGGCGGTGCTGCTGTACGGCATC
CTGGCTGTGACGGATCGTCGCAACATGGCGGTGCCGCCGGCGCTGACGCCGCTGGCTGTG
GGGCTGACCCTGTTCGCGGTCATCACCTGCTCGGGCAACAACACCGGTGCGGCCGTCAAC
CCGGCCCGCGACCTCAGTCCGCGACTGTACTCACTCGCCATCGGATACAAT---CATGCC
TTCAGTGCCGGG---------GAC---CACTTCTGGTGGGTGCCAGTGGTCAGCACGTAC
GTGGGCGCCGCCGTGGGTGCCATGTTCTACATGTTCACG---ATCGAGCTGCACCACGCG
GACCCCGACAGC---------------------------
>GHFC01061518_Neolepas_marisindica_Glp
CTGTTGAACCTGGAGCCAGTGCGACGGCGGCTGCGC---ATCAGCTGCCCGGTGCTGCGC
GAGTCGCTGGCGGAGCTGCTGGGCACCATGACTCTGGTGTTCATCGGCGACTCAGTCATC
GCTAACAACTGGTCA------------------------AGCGTGGCGAAGTCCGTGAGC
GTGCCTCTTGGATATGGCGCCGCGCTCACAATAGCAGTGTTCGCCACTGGCGGCGTGTCA
GGTGGTCACCTGAACCCGGCGGTGACACTGGCTCTCTGCATGACGGGACGGTGC---GAC
TGGACTCGTCTGCTGCCCTTCTCAGCGGCCCAGTACGTCGGTGGCTTTGTTGGTGCTGCT
CTCACTCATGGGCTGTTCTTCGATCTGTTCAAGCACGATGAG---GCTCGA---------
---------------------------------AAGGTT---TTGTTCCCTGTCTTCGCC
ACCTACCCGGCCTTCAGCGGCACG------------------------TTGCAGAACATC
TCGCTGGGCGGAGCCTTCATGGACCAGGTTATGGGCACAGCTCTGCTTCTCCTCGGCATC
ATGGCCATTACCGACCGCAGAAACATGAACGTGCCCAAGGGGATGATCCCGGTGGCGATC
GGGCTGACCTTGTTCGCGGTGATCACGTGCTCGAGCAGCAACACTGGTACCGCCCTGAAC
CCGGCACGTGACCTGGGGCCGCGCCTCTACTCGCTCGCCATTGGATACACG---GATGTG
TTCAAGTACGCC---------GAC---CACTTCTGGTGGATCCCGGTGGCCGCGTGCTAC
CTGGGCGGCACCATTGGCGCCGGCGTGTATCTGTTCTTC---ATCGAGCTCCACCACCCG

CCCGAGGAGGAGCAGGTCAGCGCCGGCGGC------GAC
>GGCH01050926_Pollicipes_pollicipes_Glp
ACCATCAGCTGGGAGCCGGTGCGGCGGGCGTGCCGG---CTCCGACATCCGATCGTGCGC
GAGACGCTGGCGGAGCTGGTGGGCACCATGACGCTGGTGTTCATCGGCAACTCCGTCATC
GCCAACAACGCAGTGAAC------------------------------GCGATGAACAAT
GAGGCGCTCGGCTACGGCGTGGCGCTCGCCATCTCCGTGTTTGCCACCGGCGGTGTCTCA
GGGGGCCACGTGAACCCGGCGGTGACCGTGGCGCTCAGTCTGGCCGGGCGCTGC---CAG
CTGAACCGCGTGCTGCCGTTCGTCCTGGCGCAGTACATCGGCGGCTTCATCGGTGCCGCT
CTCACGCACGGACTCTATTTCGATTTGTTCAAGAAAGACCCC---GGCAGA---------
---------------------------------TATAGTACACTGTACGGCATCTTCGCC
ACCTACCCCGAGCTGAACTTCAAG------------------------CTGCAGAACATA
TCGCTGGGAGGCGCCTTTCTGGACCAGGTGATGGGCACTGCCCTGCTCATGTATGGCATC
ATGGCCATCACGGATAAGCGCAACACGAACGTGCCCAAGGGTGTGGTACCGCTGGCCATC
GGCGTGACCCTGTTTGGGGTCATCACGTGCTCGAGTTCGAACACCGGCGCGGCGCTGAAT
CCGGCACGTGACTTCGCCCCACGGGTCTACTCCTATATCATCGGCTACAAG---GACGTG
TTCAAGTACTGT---------GAC---CACTTCTGGTGGGTCCCGATCGTGGCCTGCCAC
CTCGGTGCCGTCATCGGCGCGCTGGTCTACCTCCTGGCT---ATCGAGCTGCATCACCCG
ACCGGCCAGCACGAGGATGAGGACAAGGCCGTGCTCGAC
>JW965215_Argulus_siamensis_Glp3
---AAGAAAAGGAAATCGCTTTTAGAAAAAACACAG---CTTTCCAATATTTTGCTTCGG
GAATTCATGTCAGAGTTCCTGGGAACTTTTGTTCTGGTGATGGTTGGAGACGGAACAGTA
GCTGGTGTAGTGCTCAATTTGACT---------------TCTGGCGGAGAAATGTTGTTA
GCTGCATGTGGATATTCGTCAGCTTATGTCTTGGGCACTCTGATAGCTGGTGGTGTGTCA
GGAGCGCACATCAATCCAGCTGTGACTCTGGCTTACTGTATCACGGGTCAAGTC---ACT
TGGAAGCAGTTGCCGATATATTGGCTCGCACAATACACCGGAGCCTTTCTGGCATCATCT
GTGGTTTATGGAATTCATTACGAAGCCATAGTCACATATGAA---GCGATT---------
---------GATAGAAAC---------------AAAATCGACACGGCTGTGATTCTGTCT
ACTTTTCCC---------------------------------------AAGCCAAGTATA
GGTTACCCTACGGCTTATTTCGACCAGGTTCTTGGGTCCATGTTGATGATGATTTGCTTT
GAAGCCATTGCCGACCGCCGAAACATGAAGATCCCCCGTAGGATAGCTCCAGTGATGGTG
GGCTTGACCATGTTTTGTATATTTTTGGGGGTCGGCTATAACTGTGGAACTGGGTGTAAC
CCTGCAAGAGACCTTGCACCACGTATCTTTGGCTTGATTTAT---TACGATTCTGAAATT
ATGAGCCCACGT---------GGATTGGCATATTGGTGGATCCCATTTGTTGCACCTCAT
GTGGGTACGGTTTTGGGTGGGGTGGTGTATTTGGCTTTC---ATTATGTATCACCATGTA
AGAGAAGAAGACATTATATATCGGAGGTAT---CTGGAT
>JW969733_Argulus_siamensis_Glp2
GGGAAAGAACGCAGACCATTATTCACCAGGGGTAAA---GTTTTTAATATCTTCGCTCGT
GAGTTTCTGGCGGAACTTTTGGGAACATGGATGCTAGTGACCATAGGAGACGGCGTGATG
GCTGGTATGGTCCTGAACTTGAGC---------------TCTGGTGGAGAAATGATCTCA
GCTGCTGTTGGCTACGGGGCTGCGTATGCAGTGGCTACAATGGTAACAGGAGGAATCTCT
GGGGCCCATGTGAACCCTGCAGTGACACTCGCCTACTGTGTCACAGGTCATGTT---AAT
TGGGTGCAAGTTCCTATATACTGGTCCGCTCAGTACCTAGGAGCATTCTTAGGAGCTGCC
ACAGTGTATGGAATACATTATGAATGCATTACGGATTATCAA---GTCCAC---------
---------GAGCAAAAC---------------AAGATGGACACAGCCGCTATCTTCACA
ACTTTCCCA---------------------------------------GAACCGGTTGTA
GGCTACCCAACTGCCGCCTTTGACCAGGTTGTCGGATCGATGTTGATGATGATATGTTTC
GGAGCCATTACTGACAGGTACAACATGAAGATATCACAGTTTCTGGCTCCCATGTACAAC
GGCATTGTCATGTTTGCCGTCTTCTTGTCTTTTGGTTTCAACTGTGGTGCATCGTTGAAC
CCAGCCAGAGACCTGGGACCGAGACTGTTCACGCTTATATTT---TGGCCGGAAGATATA
TTGAGGCCTCTT---------GGATTTCATTATTGGTGGATACCCGTGTTGATGCCACAC
TTGGGAACTTTGTTAGGAGGTCTTCTGTATTTAATATTC---GTACTATATCATCATTTG
AGGATCGAAGGAGAT------------------------
>JW965635_Argulus_siamensis_Glp1
GCGCAGTTCAGGTCGAAGATAGGATCGAAATTGCGG---ATCAGAAACAAAGCAACCAGG
AACTTCCTCTCCGAATTTCTAGGGACCTTCATTCTAATGATATTTGGTAATGCAAGCGGA
GCTGTTGCTTATTTCAATAAGGAG---------------AATGCAGCTGGCATGGTTTTC
GTCCCTATAGCGTGGGGTCTGGGATTAATGGTGGCGATATGTATTTCTGGTGGTATTTCA
GGGGCACATTTGAACCCTGCTGTCACTTTGACCTACGCAATCCTCGGCAGGGTC---AGT
TGGGTCCAAGTTCCGGTTTACTTTTTAGCTCAATATTTTGGTGCCTTCACGGCTTCAGCG
ACGATATACGGAATATACTACGATGCCTTCAATAAATTTTTA------------------
---------GAGAAA------------------GATTACAAATCTGCTGCCATCTTTGCA
ACATTTCCT---------------------------------------CAACCCTTCGTG
GGAATACCAAATAGTTTTGCAGATGAAGTGTTGAGTACAGCTTTGCTGTTACTCGGAATC
ATGGCTATTACCGACAGTAGCAATATGACAATATTGAAGAATCTTTATCCGCTGTACATT
GGTGCACTGCTGATAGCTATACTCTACACTTTTGGTTACAACTGTGGGGCTGCTCTGAAT
CCCGCTCGAGATATGGGACCAAGAATATTTCTAGTCCTGGCTGGTTGGAATTCCGAAGTT
TTTATGCCTTAT---------GGTGGAAGCTATTGGTGGATTCCCATTATAGGCTCTCAT
ATAGGAGCTATCTTAGGAGCAGGTGTTTATCTCCTATTC---ATTGGTTTACATTTGGAA

CGTGATGAAGACGAGTCTGGTGTTCTTCCCATC------
>JAAFCF010003739_Helicorthomorpha_holstii_GlpA2
AGTTTG---TTAAACATGCTTACTCGACGCTTCCGA---ATACGTAGTTCTTACGTCAGA
GAAACTTTGGCTGAATTTTTAGGCACTTTAGTGTTGGTGGCCCTTGGAGATGCAGCCAAT
GCCCAAAGCACTTTAAGTAAAACA---------------GAAGCTGGGACCTTTCTAAGT
GTCGATTTAGCATGGGGATTTGCAGTGGCAATGGGCGTGTTTGTCAGCGGTGGAGTTAGT
GGTGGACATATTAATCCAGCAGTAACCGTAGCCATGGCATTACTAGGAAAATTT---CAA
TGGCGGAAAGTACCACTTTTTATGCTGGCGCAATACGTCGGCGCATTTGTTGGATCAGCA
ATTGTCTACTGCGTCTACTATGGTGCTCTTGGAAATTTTGAT---GATGGAATT------
GATAACTCAACCATGTAC---------------ACAATGACAACAGCTGGCATTTTTGCT
ACGTATCCT---------------------------------------AAGGATTTCTTA
TCCACCGGAAATGCAGTTGGAGATCAGGTGACTGGTACTGCTATTCTAGTAGTTATTGTT
TTAGCCATAACCGACAGTCGAAACATGGGAACCGCTAAAGGAATGGTTCCATTAATGATT
GGCGTTGGAGTTGCTTGCATCGGCATGTCCTACGGATATAACTGCGGATTTCCACTAAAT
CCAGCCAGAGATTTAGGTCCAAGATTGTTTACCGCAATTGCTGGATGGGGAAACATGGTT
TTCAGTGGTATT---GACGGTGATTTTTGCTATTGGTGGATTCCAATTGTTGCTCCACAT
GTAGGAGCCATCTTGGGTGCTATTGTGTACTGGGGCATC---ATTGAATTGCACTGGCCG
GACGAGGAAGACGAAGGACTCAATGGACCAGTTGGTAAG
>JAAFCF010006680_Helicorthomorpha_holstii_GlpA1
AGTTTG---TTAAAAGCATTAACCAGAGGATTTCGT---GTTCGTAATGTCTATTTTAGA
GAAGCATTGGCTGAGTTTTTGGGAACTTTTGTGCTGGTGACATTAGGATGTGCAGCCAAC
GCCCAAAGTGCCCTTAGTAAAGGA---------------GAAGCTGGGACCTTTTTAAGT
GTTGGTTTAGCATGGGGTTTAGCAATTATGCTCGGTGTATTTGTCTGCGGAGGAGTCAGT
GGTGGCCATATTAACCCAGCTGTATCCGTTGCAATGGCTTTACTTGGAAAATTC---CCA
TGGAGAAAAGTGCCTTTATACATGATTGGACAGTATCTGGGAGCTTTTGTTGGTTCCGCT
GTTGTTTTCTTTGTATATCAA------------------------GGTGGA---------
------------AATTAC---------------ACCATTGAAACAGCAGGCATATTTGGA
ACTTTCCCT---------------------------------------AAAGAATTTCTC
TCCGTTGGAAATGGAATTTCTGATCAGGTGTTAGGTGCGGCTATATTAGTTGCTTGTGTA
TGCGCTATAACAGACAGCAGAAACATGGGAACACCCAAAGGTGTGATTCCTCTTATGGTT
GGACTGGTAGTAGCAGTCATTGGAATGTCTTATGGATACAACTGTGGTTTTGCCATTAAT
CCAGCCAGAGATTTGGGTCCAAGATTGTTTACAGCTGTTGCTGGTTGGGGCATTCAAGTC
TTCAGCTTTAAC---------GACTATAGTTGGTGGTGGATTCCAGTTGTTGGTCCACAT
GTAGGTGCCATTTTAGGAGCCTTGATTTACTGGGTTTTA---GTTGAACTTCATTGGCCA
GAAGATGATGATGATGACATGACTCCACCAGTTGGTAAG
>JAAFCE010006668_Trigoniulus_corallinus_GlpA1
AGTTCA---TTAAGCGCTTTGACGAAAAAGCTTCAA---ATTCGTAACATATACATACGT
GAAATTTTGGCAGAAATGCTCGGAACTTTCATCCTTGTGGCCCTTGGCGACGCATCAGTG
GCTCAAAGTGTCCTGAGCAAGAAC---------------GCACAAGGAAACTTCATCACT
ATTAATTTCGGATGGGGTTTCGCGGTCATGTTCGGAGTGTTCGTTTGTGGTGGTGTTTCT
GGTGGTCATATCAACCCTGCCGTGTCGTTGGCTTTAGCAACAGTAGGAAAATTT---CCG
TGGAGAAAAGTGCCCTTGTTTATGATAGCTCAGTACGTGGGCGCTTTCATCGCTTCCGTC
TTTATTTTCCTCGTCTATAGAGATGCACTGACGGATTTTTCG---GGCGGT---------
------------AACTAT---------------ACCATAACGACGGCCGGTATCTGGGCA
ACGTATCCT---------------------------------------CAGGATTTTTTG
TCAGTTGGAAATGGTCTTGCAGATCAGGTGATGGGCACTGCTATTTTAGTGGCCTGCGTG
TTGGCCATTACAGACAACAAGAACATGAATACTGCAAAAGGATTCGTACCACTGATGGTG
GGCTTTTGTGTGGCAGCCATCGGAATGTCCTATGGCTACAATTGCGGTTATGCCATTAAT
CCAGCACGGGATTTATCTCCCCGTATTTTCACCGCTATTGCCGGTTGGGGTATGCAAGTG
TTCAGTTACGCA---------AATTATAGTTGGTGGTGGGTTCCAGTTGTTGGACCTCAT
ATTGGAGCTGTATTGGGCGCCTGGGTTTATTGGTTGCTA---GTTGAGATGCATTGGCCT
GAAGACGAAGAAGAGACGTTGAACGGTCCTGTTGGTAAG
>AFFK01019422_Strigamia_maritima_Glp
---------ATGGATGCAGTGTTGAGTCGTCTTCGT---GTGAGGATACCCATCGTCAGG
GAAGTGTTTGCCGAGTTTTTAGGAACGTTTGTACTTCTGTTTGTGGGCATTACATCAGTC
GCTCAAGCAGTTCTCAGCGATGGC---------------AAATCTGGAACCATGTTGTCA
ATTAATCTCAGTTGGGGTATCGCAGTGGCCATGGGAATTTTCATCGCTGGAGGTGTTTCA
GGTGCCCATTTAAATCCAGCTGTTACAGTCGCCTTAGCCACTTTGGGCAAATTT---TCG
TGGGTAAAGGTATTACCATACATCATTGCTCAATACATAGGAGCATTTATAGGAGCCGCG
TGTGCCTATTGGGTTTACCTCGACGCAATCGGTAAAATG------GGC------------
------------ATGATG---------------GTCCCCGAAACGGCTGGAATCTTTGCA
ACCTTTCCA---------------------------------------AAAGAATACCTC
TCAAATGGCAACGCTTTTGTTGATCAGGTAATGGGAACTGGAATTCTAATGATGGGTATT
TTGGCCATTGTCGACCCATACAACATGTCTGTGCCCAAATACGCCATACCTTTAGGAGTT
GGTTCGTTGGTTGGAGTCATTGGAATGGCGTTGGGACACAACTGTGGCTACGCAATCAAT
CCCGCGAGGGATTTATCACCGAGACTTTTCACTTTGGCAGCTGGATGGGGGAAAGAGACA
TTTGAG---CAG---------AAT---CATTATTGGTGGATCCCAATACTTGGACCTCAT
CTCGGTGCCATCATGGGCGGATGGCTGTATCAATTGTTG---GTTGGTGTTCATTGGCCG

CTTTTGGATGAAGGTAAACAAAAAAATTATGTGATAAAA
>WP_032330355_Escherichia_coli_GlpF
------------------------------ATGAGT---CAAACATCAACCTTGAAAGGC
CAGTGCATTGCTGAATTCCTCGGTACCGGGTTGTTGATTTTCTTCGGTGTGGGTTGCGTT
GCAGCACTAAAAGTCGCTGGTGCG---------------TCTTTTGGTCAG---TGGGAA
ATCAGTGTCATTTGGGGACTGGGGGTGGCAATGGCCATCTACCTGACCGCAGGGATTTCC
GGCGCGCATCTTAATCCCGCTGTTACCATTGCATTGTGGCTGTTTGCCTGTTTC---GAC
AAGCGCAAAGTTATTCCTTTTATCGTTTCACAAGTTGCCGGCGCTTTCTGTGCTGCGGCT
TTAGTTTACGGGCTTTACTACAATTTATTTTTCGACTTCGAG---CAGACT---------
CATCACATTGTTCGCGGCAGCGTTGAA------AGTGTTGATCTGGCTGGCACTTTCTCT
ACTTACCCT---------------------------------------AATCCTCATATC
AATTTTGTGCAGGCTTTCGCAGTTGAGATGGTGATTACCGCTATTCTGATGGGGCTGATC
CTGGCGTTAACGGACGATGGCAACGGTGTACCACGCGGCCCTTTGGCTCCCTTGCTGATT
GGTCTACTGATTGCGGTCATTGGCGCATCTATGGGCCCATTGACAGGTTTTGCCATGAAC
CCAGCGCGTGACTTCGGTCCGAAAGTCTTTGCCTGGCTGGCGGGCTGGGGCAATGTCGCC
TTTACCGGCGGC------AGAGACATTCCTTACTTCCTGGTGCCGCTTTTCGGCCCTATC
GTTGGCGCGATTGTAGGTGCATTTGCCTACCGCAAACTG---ATTGGTCGCCATTTGCCT
TGCGATATCGAA---------------------------
>CP026786_Shigella_dysenteriae_GlpF
------------------------------ATGAGT---CAAACATCAACCTTGAAAGGC
CAGTGCATTGCTGAATTCCTCGGTACCGGGTTGTTGATTTTTTTCGGTGTGGGTTGCGTT
GCAGCACTAAAAGTCGCTGGTGCG---------------TCTTTTGGTCAG---TGGGAA
ATCAGTGTCATTTGGGGACTGGGGGTGGCAATGGCCATCTACCTGACCGCAGGGGTTTCC
GGCGCGCATCTTAATCCCGCTGTTACCATTGCATTGTGGCTGTTTGCCTGTTTC---GAC
AAGCGCAAAGTTATTCCTTTTATCGTTTCACAAGTTGCCGGCGCTTTCTGCGCTGCGGCT
TTAGTTTACGGGCTTTACTACAATTTATTTTTCGACTTCGAG---CAGACT---------
CATCACATTGTTCGCGGCAGCGTTGAA------AGTGTTGATCTGGCTGGCACTTTCTCT
ACTTACCCT---------------------------------------AATCCTCATATC
AATTTTATGCAGGCTTTCGCAGTTGAGATGGTGATTACCGCTATTCTGATGGGACTGATC
CTGGCGTTAACGGACGATGGCAACGGTGTACCACGCGGCCCTTTGGCTCCCTTGCTGATT
GGTCTACTGATTGCGGTCATTGGCGCATCTATGGGCCCATTGACGGGTTTTGCCATGAAC
CCAGCGCGTGACTTCGGTCCGAAAGTCTTTGCCTGGCTGGCGGGCTGGGGCAATGTCGCC
TTTACCGGCGGC------AGAGACATTCCTTACTTCCTGGTGCCGCTTTTTGGCCCTATC
GTTGGCGCGATTGTAGGTGCATTTGCCTACCGCAAACTG---ATTGGTCGCCATTTGCCT
TGCGATATCGAA---------------------------
>YP_006479893_Enterobacter_cloacae_GlpF
------------------------------ATGAGT---CAGACATCAACCTTAAAAGGC
CAGTGCATTGCCGAGTTCCTTGGTACCGGGTTGTTGATATTCTTCGGAGTGGGCTGTGTT
GCTGCACTGAAAGTGGCGGGTGCC---------------AGTTTTGGTCAG---TGGGAA
ATCAGTATCATCTGGGGTCTGGGTGTGGCAATGGCCATCTACCTGACTGCAGGGGTTTCT
GGCGCACATCTTAACCCGGCGGTGACCATCGCATTGTGGCTGTTCGCGTGCTTC---GAC
GGACGCAAAGTTGTTCCTTTCATTATTTCTCAATTTGCCGGCGCGTTTTGCGCAGCGGCG
TTAGTTTACGGGCTTTATTACAATCTTTTCATCGACTTCGAA---CAGACG---------
CATCATATGGTGCGCGGCAGTGTCGAA------AGTCTGGATCTGGCAGGTATCTTCTCA
ACGTATCCA---------------------------------------AACCCGCATATC
AATTTTGTGCAGGCGTTCGCAGTTGAAATGGTGATTACCGCTATTCTGATGGGCGTTATC
CTGGCGCTGACCGACGACGGAAACGGCATTCCGCGCGGCCCGCTGGCACCACTGCTGATT
GGCCTGCTGATTGCGGTGATCGGCGCATCCATGGGCCCGCTGACTGGATTCGCGATGAAC
CCGGCGCGTGATATCGGACCGAAAGCGTTCGCCTTTATCGCTGGCTGGGGCAACGTGGCC
TTCACCGGCGGC------AAAGATATCCCTTACTTCCTGGTACCGCTGTTTGCACCGGTT
GTCGGGGCTGCGCTGGGTGCGTTTAGCTATCGCAAATTA---ATTGGTCGCCACTTACCG
TGCGACACCGAG---------------------------

File S2: Alignment for Fig. 1C

>ALA27397_Lepeophtheirus_salmonis_Glp1_v1
AAAGAAATCCAATTCGAGAATGAC---CATCCTTGTTATATTCCTACCCGTCCTCTAAGA
GAATTTTTTTCAGAGTTTATAGGAACTTTGATCTTCATTACAATCGGTATAGGAACTGTA
GCTCAAGTTACACTAAGTGGCATTCTCAAGAGT------------CCTGCAGGCACATTT
ATATCTACAAATTGGGGGTTTGGATTCGCTATTATGATTGGAATGATGGTGTCAGGTAAA
ATATCTGGAGGTCATATGAATCCAGCTGTGACAATTGCCTTTGCTATCTTTGGTAAACTA
AAACCATGGCATAAATGTTTTCATTATATGTTCGCCCAATATTTGGGAGCCTTTTTTGGT
TCGGCACTCGTTTATATTACTTACTATGAAGCTTTTCAA---GAAGCCGGT---------
---------------------------ATCAACATTGACACTGCAAAAATATTCGCAACA
TACCCT---------GCTCCTCATATTTCGGCTCCAGGAATTGTTCTTGACGAATTTGTT
GGAACAGCCCTTCTATTAATGTCAGTGTGTGCTATAATTGATAAAAAAAATCTGAACATT
CCATCCTTTTTCCATCCATTTTTAATTGGACTCGTGGTATTTGTCATAACAATGAGCTTT
AGTTTTAATGCAGGTGCCGCTTTGAACCCAGCGAGAGATTTAAGACCTCGATTATTTACA
GCAGTTTTTGGCTGGGGA---------CTAGATCCCTTTTCATATGCCAAACATTTCTGG
ATGACACCCATTTAGCGACGCATGCAGGGGCTATAATTGGTGTTTTGATATATCAAACT
TTTATAGGATGGCAATTGTCATTAGAAGACCAAAGA-
>ALA27398_Lepeophtheirus_salmonis_Glp1_v2
AAAGAAATCCAATTCGAGAATGAC---CATCCTTGTTATATTCCTACCCGTCCTCTAAGA
GAATTTTTTTCAGAGTTTATAGGAACTTTGATCTTCATTACAATCGGTATAGGAACTGTA
GCTCAAGTTACACTAAGTGGCATTCTCAAGAGT------------CCTGCAGGCACATTT
ATATCTACAAATTGGGGGTTTGGATTCGCTATTATGATTGGAATGATGGTGTCAGGTAAA
ATATCTGGAGGTCATATGAATCCAGCTGTGACAATTGCCTTTGCTATCTTTGGTAAACTA
AAACCATGGCATAAATGTTTTCATTATATGTTTGCCCAATATTTGGGAGCCTTTTTTGGT
TCGGCACTCGTTTATATTACTTACTATGAAGCTTTTCAA---GAAGCCGGT---------
---------------------------ATCAACATTGACACTGCAAAAATATTCGCAACA
TACCCT---------GCTCCTCATATTTCGGCTCCAGGAATTGTTCTTGACGAATTTGTT
GGAACAGCCCTTCTATTAATGTCAGTGTGTGCTATAATTGATAAAAAAAATCTGAACATT
CCATCCTTTTTCCATCCATTTTTAATTGGACTCGTGGTATTTGTCATAACAATGAGCTTT
AGTTTTAATGCAGGTGCCGCTTTGAACCCAGCGAGAGATTTAGGACCTCGATTATTTACA
GCAGTTTTTGGCTGGGGA---------CTAGATCCCTTTTCATATGCCAAACATTTCTGG
ATGACACCCATTTAGCGACGCATGCAGGGGCTATAATTGGTGTTTTGATATATCAAACT
TTTATAGGATGGCAATTGTCATTAGAAGACCAAAGA-
>GAZX01003450_Caligus_rogercresseyi_Glp1
---GAGCACTTAAATCGTCTGAAG---AAGTACTGTCACATAAGTAGCATACCCATGACA
GAGTTTTTTGCAGAGTTCCTCGGAACTGCTCTACTCATCATCATAGGTGTTGGGACTGTA
GCTCAAGTGACATTGAGCAACAAGATGGACGCT------------TCAAATGGAGGGCTT
TTATCAATCGCTTGGGGATTCGGAATCGGCGTCATGATTGGTGTATTGGTTGCTGGCAAA
GCCTCTGGTGGGCACTTAAACCCAGCAGTGTCCTTGGCTTTTGCTATATTCAGGAAACTA
ACTCCCTGGTATAAGTGCCTATACTACATGACAGCTCAGTACTTGGGAGCATTCTTCGGA
TCGATGGTCGTATACGCTACATACTACGATGCCTTACAC---AAAGTTGGA---------
---------------------------ATCACCTTCGAGACTTCAAAGATTTTTTCTACA
CATCCC---------TCTAATTTCATATCCTTATTTGGAGTATTTGTGGACCAAGTCGTA
TCGACCGCACTTCTACTGATATCAATTTGTGCTATTACTGACAAGAAGAATCTCAACATC
CCTCCATTTATACAGCCTTTTCTCATCGGATTGGTGGTATTTGCATTGTCTCTGAGCTTT
AGCTTCAATTGTGGAGCTGCTTTGAACCCTGCAAGAGACTTTGGTCCGAGGATCTTTTCG
GCTTTGTTCGGATGGGGA---------TTTGCTCCCTTCACACATTCGCCATACTTTTGG
ATGATCATCATAGGCTCAACACACGTCGGAGGAATCCTTGGAGTATTCATTTATCAAATA
ATTGTTGGATTTCACCTACGCTCACGAAAGGAAACC-
>GGQW01003486_Tracheliastes_polycolpus_Glp1
ATGGAA------CAGATGTTAAGA---CAAAAATGTCGAATTCCTCAACTTTGGATTCGA
GAATTTTTGGCGGAATTTATGGGAACCATGATTCTTTTAATATTAGGGGATGGAAGTGTC
GCACAAGTTGTTCTCAGCGAGTCTTTACAACTT------------CCAAAGGGAAGTTTC
TTTACAATCAACATTGGATATTCTTTAGCCGTCGTTTTTTCCCTATATACATCCGTCAAA
ATTTCGGGAGGTCATCTTAATCCGGCTGTATCTCTCTCACTCGCCGTACTTGGTAGAATG
---CCATTACACAAGTTAGGCCATTATATAATAGCTCAGTATCTTGGAGCCATGATGGGC
TCTGTATTCGTCGTTCTATTAAATATAGATGCCATCAAT---GCCGTTGGA---------
------------------------TACATCACACCAGATACAGCTTCTATTTTAACAACA
TTTCCA------CTTAATCCACTACTTTCGACTCGAGGTCAAATAATTGATACACTCTTT
GGAACGGCTTTACTTTTATTTCTCATTTGTGGTCTAAATGACAATGATGGA---AAT---
---------------CCCGTTTTGTTTGGCCTCATTGTTTTGGCTCTAGGAGTTAGTTAT
GGCTTCAATTGTGGCTATGCTTTAAATCCAGCTCGTGATCTTGGTCCTCGAATTGTGGCG
ACTTTGATGGGATGGAAC---------TTAGAGCCCTTTACGTATGGTAATCACTACTGG
TTAATACCCTCATTTGTGACGTATGTTGGAGCCTTGTTAGGAACATTTTTATACACGATC
ACAATCGGAATTCAATTATCTGTTAGACAT-------
>GHAJ01044454_Apocyclops_royi_Glp2

--------------------------------------GTATTTACGTTGGGCACCACA

GCGCAACATATTTTGAGTCGAGGA---------------------AAACAGGGAGAGTTT
TTTGAACTCAAGTTAGGAAATGGACTTGGAGTCATGCTTGGAATCTGTGTTTCAGGGGGT
GCATGCGGAGCTCACCTTAACCCGGCAGTGACCCTTGCTTTGGCTTGTAGCAAGCAATTC
---CCCTGGCGCAAGGTTCCAGCATATTTTCTGAGCCAATACCTGGCATCTTTTGTCGCT
TCAGCAGTTGTCTACTTTGTTTATCTAGATGGCATATTT---ATTTTCGCC---------
---GAT---TCTGAGCGATCAATTCGCGGAGCTAGAGCGATTGCTTCTATTTGGACGACA
TTTCCC---------TCTGAGGGAATTTCCATATCCACAGGATTTCTCGATCAAGTTGTC
GGAACAGGACTTCTTCTTTTTTGTATTCGAGGAATCATAGACCAAAGAAATTTGAAAATA
CCCCTCTATCTTGGATCGATATTGATGGGTTTGTTAGTCCTGAACATTGGACTTTCATTC
GGTTTTAATTGTGGGAATGCAATCAACCCAGCGAGAGATTTCGGCCCACGAGTATTTCTG
ACTTTAATTGGATACGGA---------ACCGAAGTTTTTACAGCTTACGGATATTGGTTC
TACGTACCTATCGTAGCAACTCATATTGGTGGAGTTGTGGGATCGTTTTTGTATGACCTG
CTCATCTACACTCATTGGAAAAAGAATCT-------
>GHAJ01003507_Apocyclops_royi_Glp1
---------------------ATG------AAGTTCAGAATCAAATCAAAACTTGTCCGC
GAAATCCTCGCGGAGTTCATTGGAACTTTCATTCTAATGGTTTTTGGTTGTGCCCGTGTC
GCCCACTCCGGGCGCCGTGGAAAG---------------------AGGCATGGAGACTTT
TTTTCCTTCTCCTGGGGGGGCGGGGGGGGGGCCATGCTCGGCGTCTTCGTCTCGGGGGCA
GTCAGCGGGGGGCACATCAACCCCGCGGTGACAGTTGCCCTCGCAGTGACAAAAAAATTT
---CCGTGGAAAAAAGTCCCCGCCTACTTCGTAGGTCAATATTTGGGATCATTCACTGCA
TCAGCAATAGTCTACCTCGTTTATTGGGATGCCTTGGAC---AGTTACACC---------
---GGC---GGAAACTTGCTCGTTTCTGGAGAAACCGCCACAGCATCCATATGGGCCACC
TACCCC---------TCAGAGGGGATCAGCAATACCATTGGAGTCGTTGACCAGATTGTC
GGCACAGCATTGCTTCTCATTGCTGTCCGAGCCATCACCGACGAGAAAAACGTGTCCATG
CCCCCCTACTTGGCCCCCTTGTTCGTAGGGTTCACCGTCTTGAACATCGGCATCTCGTTC
GGCTTCAATTGCGGCTACGCCATCAATCCTGCCAGAGACTTCGCGCCGCGGCTTTTCACT
CTCATTGCTGGATGGGGC---------ACT------------------------------
---------TTGCTGGATGGGGCACTGGGTGGAATCCTCGGATCCATCCTCTATGAGGTC
TTCGTTGGTCTTCACTGGCCAGGTGTGATT-------
>GARW01015498_Eucyclops_serrulatus_Glp1
TATGAA---GAGTTTAACAAAATG---AAAAAGCTGAAAATCAGATCACCCCTAATCCGG
GATTTCCTGGCTGAGTTTGCTGGAACATTTATTCTTTTGATTTTTGGTGAAGGCAGTGTA
GCTCAAACAGTCCTCAGTAAAGGG---------------------ACCCATGGCTCCTTT
TTCTCAATCAACTGGGGATATGGTGTTGGGGTCATGCTTGGAGCATACGTGGCAGGTGGA
ATCAGCGGAGCACATTTGAATCCGGCCGTAACTCTATCCATGGCCGTAACTAAGAAGTTT
---CCATGGAGAAAGGTTCCTGTTTACATGATTGGACAATACCTAGGGGCATTTATGGCG
TCAGTCATTGTGTACATTGTTTACTATGATGCTCTGCAA---GCCTTTACT---------
---GGT---GGTAATCTTGAAGTCAGTGGACCAAATGCAACTGCCGGAATTTGGGCAACC
TACCCT---------GCAGAAGGCATTAGCAGAGGAGTTGGTTTCATTGATCAGATTGTA
GGGACAGCTCTACTACTTTTGTGCGTTCGTGCCATAACAGACGAAAAGAACATGTCCGTG
CCAAAATACCTTATACCAGTCCTGGTTGGATTTGTGGTCCTCAACATCGGAATAGCATTT
GGATACAATGCTGGTTATGCGATCAATCCTGCCAGAGATTTTGCACCAAGGTTGTTTACT
CTCATGTGTGGCTTTGGA---------ACAAATGTTTTCACTGAATTCAACTACTGGTTT
TACATTCCGCTCCTTGCTCCACACCTTGGAGCCATTTTGGGATCCATTCTATACGAGAGT
CTGATTGCATTCCATTGGCCGGACGAGGAGGATGTC-
>LT727939_Oithona_nana_Glp1
---------------------ATG---------ATCAGAATCAGGTCACAGCCAGCCAGA
GAATTCCTCGCGGAATTGGCAGGAACTTTCATCCTTATCGTATTTGGAGATGCTTCAGTG
GCCCAATCAGTGGTCAGTAAGCAA---------------------GCCAATGGCGACTTC
TTTTCCATCAATTGGGGCTGGGGCGTGGCAGTCATGCTAGGCGTGTTTGTCTCTGGTGGC
GTCAGCGGTGGCCATTTGAACCCCGCGGTCACCTTAGCTATGGCACTCCGCAAAAAACTT
---GAGTGGAAAAAAGTTCCCGTCTATTTTCTTGCCCAATACCTTGGAGCTTTCTTAGGA
GCTGCCTTAGTCTACTTTGTTTATTACGCATTACAACAC---AATTAC------------
---GGA---GATTTTCGAGGCGTAGATGGCGCTAATGCAACTGCCGGAATATTTGCGACG
TATCCT---------ACAGCCGGAATTAGTACTGGTACCGGTCTGCTGGACCAAATCGTG
GGTACAGCTTTGCTCCTGTTTTGTATCCGCGCCATTACAGACGAATACAACATGAAAGTG
CCTAGTTATATGGTCCCAATTGCAGTTGGATTTGTGGTCTTAAACATCGGTATCTGTTTC
GGATACAATTGTGGATACGCTATCAACCCTGCGCGAGATTTGGGACCCAGATTGCTCACC
GCAGTCGTCGGATACGGA---------GCGGAAGTATTCAGTTCAAAAACGTACTGGTTT
TGGGTGCCAATTGTAGGTCCGCATATTGGAGCCATAATAGGCTGCTTCCTCTACGATTTG
CTGATAGGTCTTCATTGGCCCCATAGCGAA---GTC-
>GFWY01001247_Acartia_tonsa_Glp2
---------------------ATG---AAGAAGTGGAAAGTA---TCTTTGTGGATAAGA
GAATGTCTGGCAGAAATTCTTGGAACAGGAATACTGCTGGCCTTTGGAACTGGAAGTGTA
GCACAGGCTGTACTATCTGGCCGC---------------------CAACCGCTTGATATG
CTATCCATAAACATTGGATGGGCAGCTGGAGTTATTCTTGGCATTCTCTCATCAGCGGCT
GTTTCAGGAGCGCATTTAAATCCTGCAGTAACCATTGCTCAAGCTGTTATGGGCCAGTTT
---CCATGGAAAAAGGTGCCACACTATCTGTTGTCTCAGTATCTAGGAGCCTTCTTGGGC

GCAGGAGTTACACTTTTAACCTACAGGGAAAATATTCTT---GACTTTGCT---------
---GGC---GGTGAATTTCAGGTTTCAGGTGAAAATGGAACAGCTGGCATTTTTGTAACT
TCACCT---------AAACCTGGACTATCAAATTTTTCTGGAGCTATTGATCAGGTTGTT
GGGACAGCATTACTTACTCTGTGTCTTGGAATTCTAGGAAATCCACGAAATGAGAAGTTG
GTTGGCAATCTTGGACCTTTTCTAGTTGGCCTTATTGTTCTTGCCATTGGGGTTTGCTTT
GGTGATAACGCAGGGTATGCCATAAATCCAGCTCGGGACCTTGGACCAAGAATCTTGTTA
CTCATAACTGGCTATGGA---------ACTGAGGCATTCTCTGTCAGTTATTACTGGTTT
TGGATTCCTGTTGTTTGTTGTCATATTGGAGGAATACTTGGTTCTCTCATCCATTTTCTT
TTTATTGATGGATTTCATCAGAATGATAGC-------
>GGQN01097685_Temora_longicornis_Glp2
---------------------ATG---AGAATAGCACGAAAGTAC---CCATGGCTAGGA
GAGTCTTTAGCCGAAGTTATTGGTACAATGGTGCTCCTCGCTTTCGGCACCGGCAGTGTA
GCACAGGCTGTTTTATCAAATAGT---------------------GTCAACGGAAATATG
CTCTCCATTAATATAGGCTGGGGCGCAGGAGTCATATTGGGTGTCATGGTTGCAGGACCT
ATTTCAGGAGCACATTTGAACCCTGCTGTGTCCGTAGCATTGTGCGCTGTCGGGAAGTTT
---CCGCTCAAGAAGTTGCCGCACTACATTATCTCGCAGTACATCGGGGCTTTCCTTGGT
GCAGGCGTAACGTTGTTGACGTACCATGAGTCTATTCAC---GGATTCTCA---------
---GGT---GACGGTTTACAGGTATCTGGGATCAATGGAACGGCTGGGATATTTGTGACT
TTCCCT------GGACGAGAAGGAATATCAAACCTCAGCGGAGCAGTTGATCAGGTTGTT
GGTACAGCTCTGCTCCTCATCTGCGTCTGTGCTATAGGCCTACCTAAGAACAAAGCTGTC
TCTGGCACGCTTGGTCCCCTCCTGGTTGGAGTCACCGTCCTCGCCATCGGGATATGCTTC
GGGGACAACGCTGGGTACGCAATCAACCCAGCGCGTGACCTGGGGCCGCGTATTCTGCTG
TCCATGGCGGGCTGGGGC---------ACGGAGGTCTTCACCGCCAGCTACTATTGGTTC
TGGATCCCTGTAGTTTGTTGCCATATTGGAGGGATCCTTGGTGCTCTCATTCACTTTCTG
TGCGTAGAGAAACTCATGGACCAAGAGGATTTCCAC-
>GBGO01059541_Eurytemora_affinis_Glp2
---------------------ATG---AAAAAGATTTACTGT---GCAGAATGGATGCGA
GAAAGTTTAGCTGAGATAATCGGAACTATGCTGATATTAAGCTTTGGTACTGGAAGTGTG
GCTCAGGCTGTTTTGTCGCATAAA---------------------GAAAATGGAAATATG
TTCTCAATTAATGTTGGATGGGGGGCAGGGGTAGTTCTTGGTATCATGGCTTCAGCTCCT
ATATCAGGTGCACACCTGAACCCTGCAGTAACCATTGCCCTAGCAGTTGTAAATAAATTC
---CCCTGGAAAAAAGTTCCCCACTACATGATCTCCCAATATATTGGAGCTTTTATAGGC
GCCGCTATTACTTTGTTGACTTATAGAGAGGCAATTGTG---AACTTTTCC---------
---GGT---TCAGAACTTCAGGTGCTTGGGGAGAACGGGACAGCTGGGATCTTTGTAACA
TTTCCC---------AATAATGAGATTTCAAACTTGTCGGGAGCCATTGATCAGATTGTA
GGAACAGCACTACTATTGATCTGTGTACTGGCCATAGGTTTGCCTGGTAACAATAATGTA
TCCAAGTCCCTTGGTCCACTGCTGGTTGGTTTAACAGTTGTTGCTATCGGAGCATGCTTC
GGACACAATGCAGGGTATGCTATAAACCCAGCACGTGACTTTGCTCCACGCGTTCTTCTT
TCTATTTCCGGTTGGGGA---------ACAGAGATCTATACAGCTAAACAAACATGGTGC
TGGATTCCTGTGGTGTTTTGCCATATTGGAGGTATCCTAGGAGCTCTTCTATATTACTGG
GGAATAGAAGTCTGGCAGAATAAAAAGTCTAATAAC-
>GFWO01123619_Labidocera_madurae_Glp2
---------------------ATG---AAGAGATACAAGTGC---TCCCCGTGGATCAGA
GAGAGTCTAGCGGAACTTGTTGGAACCATGATTTTGTTGATGTTCGGTACAGGATCTGTA
GCTCAGGCAGTATTATCAAGACGA---------------------GTGAACGGGGATATG
TTCTCTATCAACTTCGGTTGGGGGATCGGAGTAGTGATGGGAGTGATGGCTGCAGGATCT
ATCTCAGGAGCGCATCTGAACCCAGCGGTCAGCCTGTCCCTGGCAGTGGTGGGGAAGTTC
---CCCTGGAAGAAGATCCCCCACTACTTCATCAGCCAATACGTCGGGGCTTTCATAGGA
GCAGGGATTACTCTACTCACTTACAGAGAAAGCATCGTA---AACTTCTCT---------
---------GATAACCTGGAGGTGCTGGGGGTCAACGGAACAGCTGGAATATTTGTCACA
TTTCCA---------AAAGATGGAGTATCTAACTTTGGTGGGGCTCTAGATCAGATTGTT
GGCACTGCAATTCTCCTACTCTGCATTTGTGCAATAGGTTACGAGAAGAATGGTGCAGTT
TCAAAGACCATGGGACCCTTCCTAGTCGGTCTGGTGGTGATCGGTATAGGAGCTTGCTTC
GGGGATAATGCAGGTTATGCGATCAATCCAGCGAGGGATCTATCTCCAAGAATTCTACTA
TCTATGACTGGCTGGGGA---------GCTGAGGTGTTCATCGCCGCAAACTACTGGTTT
TGGATTCCTGTTGTGTTTTGTCATATTGGAGGAATACTTGGAACTCTTATCTTTTATCTT
TCTGTGGAGATTCTCGAATCAAAGGAGGATGAGTAC-
>GHWV01136355_Pseudodiaptomus_annandalei_Glp2
---------------------ATG---AAG---ATAAGGAGGTACAAGAATGTAATTAGT
GAATTTCTAGGTGAACTTGTGAGCACATGGATCCTTCTCTCCTTCGGCACGGGTGTTGTG
GCTCAGACCGTGTTGTCGCGGAAC---------------------AGCGCCGGCTCCATG
CTCTCCATCAACATCGGCTGGGGCGCCGGCGTCACTCTGGGCATCCTGGCGGCAGGTCAA
ATCTCAGGGGCACACATGAACCCTGCAGTGACCGTGGCTCAGGCCGTCTGGAAAAATTTT
---CCATGGAAAAAGGTACCTCACTATATTGTAGCCCAGTATCTTGGAGCATTCCTAGGA
GGTGCTACAACTCTCGTTACCTATTGGGAATCTATTCAA---CTATATTCG---------
---GGA---GATCATTTACAGGTGTCAGGAGAGAATGGAACAGCCATGATCTTCACCACG
TTCCCG---------GCCGAGGGCGTGTCCAACGTCGGTGGCGCCGTTGACCAGATTGTC
GGAACTGGCCTGATGATGGTTTGTTTGTCAGCAATAGGCTTGCCAAAGAACAGTCACGTG

TCTCGAAGTTTGGCGCCATTTCTGGCCGGGCTGGTCATCCTCACAATTGGTGTTGCTTTT
GGATACAACACAGGGTATGCTTTGAACCCAGCAAGGGACTTCTCCCCACGTTTCCTGCTG
TTCATCTCTGGTTGGGGA---------AGTCAAGTCTTCAAGGAGCACGATTACTGGTTC
TGGATTCCAATTGTAGCAACCCATGTTGGAGCAATCTTAGGCATTGGAGTCTATCGACTG
TCAGTGGAGTTTTGGCTTCACGAGGAGGACGTAGAG-
>GFUD01036665_Neocalanus_flemingeri_Glp4

---------------------------------------GGGCTCCTCATAGCTGGGCCT
GTCTCAGGAGGACACCTCAACCCAGCAGTCTCCCTATCTCTGGCCTGTGCTAGAAAGTTC
---CCCCTGTCCTCCCTGGTCCACTACATGGCTGGTCAGTACCTGGGGGCATTCCTGGGC
TCCTCCTTGGTGCTGCTCACCTACAAGGACGCTCTCAAC---CACTTCTCT---------
---GGC---GGGGAGTACGTGGTCAGCGGGGAGAATGCTACTGCGGGTATTTTTGTTACT
TTTCCA---------GCTGAGGGAGTTTCCAACCTGGGAGGTGCTATAGATCAGGTGGTT
GGAACAGCTCTTCTGCTCCTCTCTGTCAGTGCAATAAACTTCCATAAAAACTCCAAGATA
TCAACCAGTCTTGGGCCTCTCCTGGTGGGTCTTGTAGTGGTGGCCATTGGGGTCTGCTTT
GGGCATAATGCAGGATATGCGATAAACCCCGCGAGAGACCTTGGTCCACGTGTGATGATC
TCCTTGTGTGGCTGG---

>GHLB01015685_Neocalanus_flemingeri_Glp3

--CTGGGGGCATTCCTGGGC
TCCTCCTTGGTGCTGCTCACCTACAAGGACGCTCTGCAC---CATTTCTCT---------
---GGT---GGGAAGTATGTGGTCTCTGGGGAGAATGCTACTGCTGGTATCTTTGTAACT
TTCCCA---------GCTGAGGGTGTGTCTAACTTGGGAGGTGCTATAGACCAGGTGGTT
GGAACAGCTCTCATCCTCCTCTCTGTCAGTGCGATAAACTTCCACAAAAACTCCAAGATA
TCCAGCAGTCTTGGACCTCTTATAGTGGCTCTGACAGTTGTGGCCATTGGGGTCTGCTTT
GGGCATAATGCAGGGTACGCCATCAACCCAGCCCGAGACCTGGGTCCACGGGTGATGATC
TCCTTGTGTGGGTGGGGG---------ATGGAGGTGTTCACCAGCCATGGATGGTGGTGG
TGG---

>GAXK01179438_Calanus_finmarchicus_Glp3
---------------------GCT---AGA---AGTTTGGTG---------ATGCTGAAG
GAGTTCCTTGCTGAGCTGGTTGGTACTTGTGTGCTGCTCATGTTTGGTTGCGGGTGTGTT
GCTCAGTCAGTGCTGAGTGGAGGG---------------------GCTAATGGGGGTATG
CTGTCCATCAATATTGGGTGGGGGCTGGGGGTGCTTGTGGGGGTGCTCATTGCTGGGCCA
GTCTCAGGAGCCCACCTGAACCCTGCAGTAAGCCTCTCCCTTGCCTGTGTGCGTAAGTTC
---CCCCTGACCTCCCTGGCTCACTACCTGGCAGGGCAGTACCTGGGGGCATTCCTTGGC
TCCTCCCTTGTCCTCTTCACCTACAGGGATGCTCTTCAC---CATTTCTCT---------
---GGT---GGCAAGTATGTGGTGGCCGGGGAGAATGCTACTGCGGGTATTTTTGTAACT
TCTCCC---------GCTGAGGGGGTGACTAACTTTGGAGGAGCTGTGGATCAGGTTGTT
GGGACAGCCCTTCTTCTGCTCTCTGTAAGTGCCATAAACTTTCATAAGAACTCCAAGATA
TCCAACAGTCTTGGGCCTCTTCTGGTGGCTCTTACAGTGGTGGCTATTGGGGTCTGCTTT
GGGCATAATGCAGGGTACGCAATCAATCCTGCGCGAGACCTTGGTCCTAGGGTGATGATC
TCTCTGTGTGGGTGGGGG---------TGGAAGGCTTTCAGCAACCATGGGTGGTGGTGG
TGGATACCTGTGGTGTGTTGCCATCTGGGAGGCATTGTGGGG------------------

>GIVD01052235_Rhincalanus_gigas_Glp5
---------------------ATG---AAG---TCTGGAATA---AAGGAAGTCATAAGG
GAGTTTGTTGCGGAAATGTTAGGAACATGTGCTCTGTTAATGTTTGGCTGTGGATGCGTA
GCACAGACTGTATTGAGTGGAGGA---------------------ACAAATGGCAACATG
TTCTCCATTAATGTTGGTTGGGGTCTGGGAGTCTTACTTGGTGTAATGGTTGCTGGATCT
GTCTCAGGTGCACATTTGAACCCTGCTGTAAGTGTTGCCTTGACTTGTATTGGAAAGTTT
---AAAGTTACTAAGCTACCTCACTATTTAGCAGGTCAATATTTGGGTGCCTTTCTTGGT
GCCGCTCTGGTTTTCCTTACCTACAGGGATGCTCTTGAC---CATTTTAAT---------
---GGTGATGGACCATATCAGGTTGAAGGTGCTAATGCAACTGCTGGAATTTTTGTGACT
TTTCCC---------AGCCCTGGAGTTTCCAACATTGGAGGATTCATTGATCAGGTGGTA
GGAACAGCATTGTTGCTTCTGGCTATTGGAGCGATCAACTTCCGTCCCAACTCCAGTGTT
TCATCAAGTTTAGGACCTCTCTTAGTGGCATTTACTGTTATGGCTATAGGGATCTGTTTT
GGGCATAATGCTGGGTATGCTATAAACCCAGCCAGAGATCTGGCTCCTAGGATATTCATC
TCTCTTGCAGGCTGGGGT---------CTTGCAGCCTTTACTGCACATAATCACTGGTGG
TGGATACCTGTAGTTGCCTGCCATCTGGGAGGATTGTTGGTGCTGGGATCTTCTACTTT

CTTATTGAGAAGGGA---TATGAAGAGGAGGAGGAT-
>GIVD01030080_Rhincalanus_gigas_Glp4
---------------------ATG---AAG---TCTGGAATA---AAGGATATCATAAGG
GAGTTTGTTGCAGAAATGTTAGGAACATGTGTTCTGTTAATGTTTGGCTGTGGATGCGTC
GCACAGACTGTATTGAGTGAAGGA---------------------ACAAATGGTAACATG
TTCTCCATCAATGTTGGTTGGGGTCTTGGAGTCTTACTTGGTGTAATGGTTGCTGGATCT
GTGTCAGGTGCACATTTGAACCCGGCTGTAAGTGTTGCCTTGACTTGTATTGGAAAGTTT
---AAAGTTACTAAGCTACCTCACTATTTAGCAGGTCAATATCTGGGTGCCTTTCTTGGT
GCCTCTCTTGTTTTCCTCACCTACAGGGATGCTCTAGAC---CATTTCAAT---------
---GGTGATGGACCATATCAGGTTGAAGGTGCCAATGCCACTGCTGGAATTTTTGTCACT
TTTCCT---------AGCCCCGGAGTTTCCAATATTGGAGGATTCATCGATCAGGTGGTA
GGAACAGCATTGTTACTTCTGTGTATTGGAGCAATCAACTTCCGTCCCAACTCTAGTGTT
TCATCAAGTCTAGGACCTCTCTTAGTGGCACTTACTGTTGTGGCTATAGGTATCTGCTTT
GGTCATAATGCTGGGTATGCCATTAACCCAGCCAGAGATCTGGGTCCTAGGATATTCATC
TCTCTTGCAGGCTGGGGT---------CCTGCAGCCTTTACTGCACATAATCACTGGTGG
TGGATACCTGTAGTTGCCTGCCATCTGGGAGGAATTGTTGGTGCTGGGATCTTCTATTTT
CTTATTGAGAAGGGA---CATGAGGAGGAGGAGGAT-
>GFCI01505892_Pleuromamma_xiphias_Glp3
---------------------ATG---------GCCTGGATCCGCGTGGAGTGGGTGAGG
GAGATGCTGGCTGAGATGATCGGGACCTGGGTCCTGATCTGCTTCGGCTGCGGTTGCGTG
GCCCAGTCCGTCCTCAGCGGCGGC---------------------AAAAACGGCGAGATG
CTCTCCATCAATCTGGGCTGGGGGATCGGCGTCACCATGGCCGGGCTCATTTCGGGCAGC
GTATCGGGTGGCCATTTAAATCCAGCCGTGTCTCTTGCCCTGGTGGTTGTGGGTCAAATG
---CCTGTAACCAAACTGGCCCATTACATGGTGGGCCAATATTTGGGAGCATTTGCTGGC
GCTGCGACGGTATGGGTGGTTTACCTGGACGCCCTCAAC---CATTACGCG---------
---GGG---TCAGATCGATTGGTGGACGGCGAGAATGCCACTGCCGGCATCTTCGCAACG
TACCCG---------GCGGACGGCGTGTCCAACCTGAGCGGCGGCGTGGACCAAGTCTTG
GGCACGGCCCTGTTGCTGCTCTGCCTCCTCGCCATCAGGGATCCCGCCAACGGCAGCCTC
GCCAAGTCCATGGGTCCTGTCCTCGCCGGACTCGCAGTCACCAACATCGGTATCAGCTTC
GGCCACAACTGCGGCTACGCTATAAACCCCGCTAGAGACCTTGGTCCGCGGGTGTTCACG
GTGATGGCCGGGTGGGGA---------GGGCGAGTATTCACCTCCCACCACGGCTGGTGG
TGGATCCCCCTGGTCTGCAGCCACGTCGGTGGCGTCCTCGGTGCCCTCCTCTACACCGTA
GGCATCAAGATCAACACTAACCACGCGAGGATGCAG-
>GFCI01085950_Pleuromamma_xiphias_Glp2

------TCCATCCTCAGCAACCGC---------------------GTGCACGGCGAGATG
CTCTCCATCAACCTGGGCTGGGGGATCGGCGTCACCTTGGCCGCGCTCATTTCGGGCAGC
GTATCCGGTGGCCATTTAAATCCCGCGGTGTCTCTGGCGCTGGCGGCTGTGGGTCAAATG
---TCTCCATCCAAACTGGCCCATTACATGGCGGGACAATATCTAGGAGCATTTGCAGGC
GCCGCTACGGTGTGGCTGGTATACCTGGACGCCCTCAAC---GAATATGCG---------
---GGG---TCTGAGCGGCTAGTGGACGGCCCCAACGCCACGGCCGGCATCTTCGCCACG
TACCCG---------GCGGAAGGCGTGTCCAACCTGGGCGGCGGCGTGGACCAGGTGGTG
GGCACGGCCCTGCTGCTCCTCTGTCTCCTCGCCATCGGAGACCCCGCCAACGGCAGCCTG
GCCAAGTCCATGGGGCCCGTCCTGGCAGGACTCGCCGTCACAAACATCGGCATCTGCTTC
GGCCACAACTGCGGCTACGCCATCAACCCCGCCAGAGACCTTGGTCCTCGGGTGTTCACG
GCGATGGCTGGGTGGGGA---------GTTCGAGTGTTCACCGCCCACAACGGGTGGTGG
TGGATCCCCCTGGTCTGCAGCCACGTCGGTGGCCTCCTCGGCGCCCTCCTCTACACCCTC
GGCATCAAGGTCAACAACGACCACGCGATGGTGAAC-
>LS050154_Acartia_tonsa_Glp1
------------ATGGGCTGT---------TCCCCGCGCATCCGGAGCCCCGTGATGCGG
GAGTTTCTTGCGGAGTTTTTAGGAACCTTTGTGCTAGTTTTGTTTGGAAACTGTTCCATA
GCCCAATCTACCCTCTCTCTTAGT---------------------AGCAAAGGAGACTTT
TTCTCCATTAATTGGGGGTGGTGTGTTGGAATTATTCTTGGTATTCTTGTGAGCGGAGGA
GTTTCAGGAGGGCACTTGAATCCCGCAGTATCCGTAGCTGTGGCCACTCTTGGCAAGTTT
---CCATGGTGGAAGGTTCCCCATTATCTTGGTGCGCAGTACCTTGGGTCCTTTGCCGCG
TCTTGTGTCACCTTCCTGGTCTACTGGGACGCCCTTGTC---TGGTATGAG---------
---CAT---AACCGTGGCGCGTACCGGTCCACCCCGGACACAGCGGGCATATTCGCCACG
TACCCC---------GCTCCCCATCTCTCCTGGCTGGGCGGCATTGGGGACCAGATTTTG
AGCACGGCGATGCTAGCGGCATGTGTATGCGCTATCACCGACAGCAAGAATATGAATGTA
AGTAAGCAACTTGTACCGCTCTACATTGGCCTGACTGTGCTTGGAATTGGAATTTGTTTT
GGAGCTAACTGCGGATATGCCATCAATCCTGCCAGGGACCTTGCTCCTAGGCTGTTTACA
GCCCTGGCTGGTTGGGGA---------CCAGAGGTGTTCTCTTTCTATTCTCAGTGGTGG
GTGGTGCCCGTCATAGCCACCCATGTCGGAGCTATAGCTGGTGCTTGGCTGTACTTCCTT
TGTATTGAAATGAACTGGCCAAGGGATGTTGAATTT-
>GFWO01073465_Labidocera_madurae_Glp1
---------AAGGCCCGCTGC---------TCCCCGCGGATCAAGAGCCCGATGGTGCGG
GAGTTCCTCGCCGAGTTTCTCGGGACCTTCATCTTGGTTCTGTTCGGGAACTCCTCGATC

GCCCAGTCGGTGCTCTCCCTCCAC----------------------AACAAGGGGACTTC
TTCGCCATCAACTGGGGATGGTGCATCGGAATTCTCCTTGGGATTGTGGTCAGCGGGGGC
GTATCAGGAGGTCACCTGAACCCCGCTGTATCCGTGGCGGTCGCTACCCTGGGAAAATTT
---CCCTGGTGGAAGGTACCGCACTACCTGGCAGCGCAGTATCTTGGCGCATTCGTTGCG
TCATGCGTCACTTTCCTGGTCTACTGGGATGCGCTTGTC---TGGTATGAG---------
---CAC---AACCGGGGGATGTACCGGTCGACGCCGGACACCGCAGGGATCTTCGCCACG
TACCCG---------GCGCCGCACCTCTCCTGGGCGGGCGGTGTCGGCGACCAGTTCCTC
GGGACGGCGCTCCTCCTCGCCTGCGTCTGCGCCATCACCGACAGCAAGAACATGAAGGTG
AGCAAGCAGCTGGTCCCCTTGTACATCGGGTTCACCGTGCTCGGGATCGGAGTCGCCTTC
GGGTTCAACTCCGGGTACGCGATCAACCCTGCCAGGGACCTTGCACCCAGACTCTTTACA
GCTATCGCTGGCTGGGGA---------CCTGAGGTGTTCAGCTTCTATTCTCACTGGTGG
ATTGTTCCAGTCGTAGCTTGCCATCTTGGAGCTATAGCTGGAGCCTGGTTGTACTATCTC
TGCGTTGAAATGAACTGGCCCAAGGACGTTGATATT-
>GGQN01024194_Temora_longicornis_Glp1
---------AAGGGCGGGTGC---------TCCTTCAAGATTCGTAGCCCGGTGCTAAGG
GAGTTTCTCGCGGAGTTTTTAGGGACTTTTATCTTGGTTCTGTTCGGGAACAGCTCCATC
GCGCAGTCCGTGCTCTCCCTCAAC----------------------AACAAGGGGACTTC
TTCGCCATCAACTGGGGATGGTGTATCGGGATCATCTTGGGTGTAATGGTGAGCGGCGGA
GTCTCAGGGGGCCACCTGAACCCCGCTGTCTCCGTGGCAGTTGCAACCCTTGGGAAATTC
---CCATGGTGGAAGGTTCCGCACTACTTGGGGGCTCAGTACCTTGGAGCCTTCGCGGCG
TCGTGCGTCACCTTCCTCGTCTACTGGGACGCACTTGTC---TGGTATGAG---------
---CAC---AACCGTGGCGTGTACCGGTCCACCCCGGACACGGCCAGCATCTTCGCGACC
TACCCC---------GCCCACCACCTCTCCTGGGCCGGGGGCGTGGCGGACCAGGTTCTT
GGAACTGCTCTTCTTCTGATTTGTGTGTGTGCTATCACCGACTCAAGGAATATGAACGTG
AGCAAGCAGCTGGTCCCCCTGTACGTGGGGTTCACCGTG---------------------

>NW_019396719_Eurytemora_affinis_Glp1
---------AAAGGGGGCTGT---------TCGCCCCGTATCAGGAGTCCGCTTCTTCGG
GAGTTTTTAGCTGAGTTTTTAGGAACATTTATCCTTGTCCTGTTTGGAACTGGATCTATT
GCACAGTCTGTTCTCTCTCTCAAC----------------------AACAAGGGGACTTC
TTCGCTATAAACTGGGGGTGGTTTATTGGAATACTGCTCGGTGTGCTGGTTAGTGGAGGA
GTTTCAGGAGGACATCTGAACCCTGCAGTTTCTGTAGCTGTTGCTACACTTGGCAAATTC
---CCTTGGTGGAAGGTTCCTCACTACTTGGCAGCTCAGTATCTGGGAGCATTTACTGCT
TCAACTGTAGTATTCTTGGTCTACTGGGATGCTCTTGTC---TGGTATGAG---------
---CAT---GATCGTGGAGCATACCGTACAACCCCTGATACGGCCGGTATATTTGCCACC
TACCCT---------GGCAGGCATCTGAGCTGGGCGGGCGGTGTCGGAGATCAATTCCTT
GGTACTGCTATGCTACTGATCTGCCTCTGTGCTATCACTGACAGAAAGAATATGAATGTA
AGTAAGCAGTTGGTTCCCTTGTACGTTGGATTTACTGTGCTGGGTATTGGAATCTGTTTT
GGGTTTAACTGCGGCTATGCGGTCAACCCCGCTCGAGATCTTGCACCTAGACTTTTTACA
GCACTGGCTGGTTGGGGC---------CCTGAAGTATTCAGTTACTATTCTCATTGGTGG
ATAGTTCCTGTGGTTGCATCTCATCTTGGAGCTATAGCTGGAGCATGGATTTACTATCTG
TGCGTCGAGATGAACTGGCCAAGGGATCTTGAACCT-
>GHWV01136883_Pseudodiaptomus_annandalei_Glp1
---------AAATCGCGATGC---------TCTCTCAGGATAGAGAGTGTTGTGATTCGG
GAATTCCTGGCAGAGTTTCTCGGGACCTTTGTCCTGGTGACCTTTGGCGCCGGGTCCGTG
GCCCAGTCCGTGCTCAGTCTCAAC----------------------AACAAGGGAGACTTC
TTCGGCATCAACTTCGGTTGGGGTCTTGGCGTGATGATGGGCATCCTGGTGAGCGGCGGC
GTGTCTGGCGGTCACCTCAACCCTGCCATCACCGTGGCCGTGGCCACCCTCGGCAAGTTC
---CCCTGGTGGAAGGTGCCGCACTACATCGCTGCCCAGTATCTCGGGGCGTTCACCGCC
TCCTTCGTCATCTTCTTTGTCTACTGGGATGCTCTTGTC---TGGTATGAG---------
---CAC---GACCGCGGGGCGTACCGCTCGATCCCCGACACGGCGTCCATCTTCGCCACC
TACCCG---------GCGCCGCATCTCACCTGGGCTGGAGGAATGGGCGATCAGTTCTTG
GGAACTGCTCTGCTGCTCATCTGCGTCTGTGCCATCACTGATCAGAAGAACATGAATGTG
AGCAAGCAGCTGGTTCCTCTCTTCGTCTTCTTCACGGTCTTGGCTATCGGGGTCTGCTTT
GGCTTCAACTGCGGCTATGCCATCAACCCCGCTAGGGATCTCGCCCCCAGGTTGTTCACT
GCCATTGCTGGTTGGGGC---------CCTGGAGTCTTCACCTCCTACAACCATTGGTGG
GTGGTTCCCGTAGTGGCCTCTCATATTGGAGCCATTGTTGGAGCTTGGGTTTACTACCTT
AGCGTTGAGCTGAACTGGCCCAGGGAATGTGACATG-
>GFUD01101212_Neocalanus_flemingeri_Glp2


```
------------------------------------------------------------
------------------------------------------------------------
------------------------------------------------------------
------------------------------GCAATCACAGACAAACAGAACATGGAGGTG
AGCAAGCAGCTGGTCCCCTTCTTTGTTGGCTTAACTGTTCTTGGCATTGGGATCTGCTTC
GGCTACAACTGTGGCTATGCTATCAACCCGGCCAGAGATTTTGCACCCCGGCTGTTTAGT
GCCCTGGCTGGGTGGGGG---------CTGGACGTGTTCACCTACTCTTACCACTGGTGG
GTTGTACCAATATTTGCTACACATGTGGGGGCTGTGCTGGGTGCCTGGGTGTACTATCTA
GCTATAGAACTCAACTGGCCCAAGGAACATTCTGAT-
>GAXK01070720_Calanus_finmarchicus_Glp2
------AAGAAAGGAGGCTGCTGT---GCTTCATGGCGAATAACCAGCCCGGCTTTGAGA
GAGTTTTTGTCTGAGTGTTTTGGCACTTTCATCCTGGTTGTGTTTGGTTGTGGCTCCATT
GCCCAGTCAGTCCTCTCGCTTCAG---------------------ACAAAAGGAGACTTT
TTCTCCATCAACTGGGGGTGGTGTCTAGGAGCCACCCTTGGAACTCTAGTCAGTGGAGGA
GTGTCAGGCGGTCACTTGAATCCTGCGGTTACAGTGGCCCTTGCTACCCTGGGAAAAGTT
---CCCTGGAAAAAGGTTCCTCACTACCTGGCAGGTCAGTACTTGGGTGCTTTTATTGCT
TCCTTTGTTGTATTCTTGGTCTACTGGGATGCTCTAGTC---TGGTACGAA---------
---CAT---GACAGAGGAGGATACCGCATTACTCCAGACACTGCTGGCATCTTCGGCACC
TACCCT---------TCTCACCATCTCACCTATGCTGGAGGTGTTGGGGATCAGGTTCTT
GGGACGGCCCTTCTCTTGCTCTGTGTTTGTGCAATCACGGATAAGCGGAACATGCAGGTG
AGCAAGCAACTGGTTCCCTTCTTTGTTGGTCTGACTGTTCTTGGTATTGGGATCTGCTTT
GGTTACAACTGTGGCTATGCTGTCAACCCTGCTAGAGATTTTGCACCACGGCTGTTTAGC
GCCCTGGCTGGGTGGGGG---------CCGGATGTGTTCACCTACTCTTACCACTGGTGG
GTTGTTCCCATATTTGCTACTCATGTGGGAGCTGTGCTGGGTGCTTGGCTATACTACATT
GCTATAGAACTCAACTGGCCAACAGAGGAACCTGAT-
>GAXK01165253_Calanus_finmarchicus_Glp1_v1
------AAGAGAAGCAGCTGCTGT------AGTTTCAAGATCATCAGCCCTGTAATCAGG
GAGTTTCTTGCAGAGTTCCTCGGCACCTTTATTCTTGTCCTCTTTGGTACTGGCTCAGTG
GCCCAGTCAGTTCTATCTCTGGGA---------------------CAGAAGGGAAGCTTC
TTCTCCATTACCTGGGGGTGGGGTATTGGAGTTATCCTCGGTATTGTGGTAAGTGGCGGG
GTGTCAGGAGGCCATCTTAACCCGGCTGTTACCGTCGCAGTTGCATCTGTTGGAAAATTT
---CCTTGGCGCAAGGTTCCCCACTACCTTGCTGCTCAATATCTTGGAGCATTTGTTGCC
TCTTGTGTTGTTTTCCTTGTGTACTGGGATGCTCTGGTC---TGGTATGAA---------
---CAT---GACCGAGGAGAATACCGCACCACTCCTGACACTGCCAGCATTTTCTCCACC
TACCCC---------TCGCCACACCTGACCTACATGGGCGGTATAGTGGACCAGGTGGTG
GCCACAGCCTTGCTACTCATCTGTGTCAGTGCCATCACGGACAGGCGCAACATGCAGGTG
TCAAAACAGTTGGTCCCTTTCTTCATTGGCCTCACTGTTCTCGGCATTGGAATCTGTTTT
GGCTTCAACTGTGGCTACGCTATCAACCCAGCAAGAGACTTGGCCCCACGGTTGTTCACT
GCTCTATCCGGGTGGGGG---------CTGGAAGTTTTTGCCTTTTCCAGCCCCTGGTGG
TTGGTGCCGATACTTGGCACCCATGCAGGGGCTGTGCTTGGTGCCTGGGTGTACTATCTA
GCCATAGAGCTGCATTGGCACAAGGATGATACTGAG-
>GBFB01029789_Calanus_finmarchicus_Glp1_v2
------AAGAGAAGCAGCTGTTGT------AGGTTCAAGATCAGTAGCCCTGTCATCAGG
GAGTTTCTTGCAGAGTTCCTCGGCACCTTCATTCTTGTCCTCTTTGGTACTGGCTCAGTG
GCCCAGTCAGTTCTCTCTCTGGGA---------------------CAGAAGGGAAGCTTC
TTCTCCATTACCTGGGGGTGGGGTATTGGAGTAATCCTCGGTATTGTGGTAAGTGGCGGG
GTGTCAGGAGGCCATCTTAACCCGGCTGTTACCGTCGCAGTTGCATCTGTTGGAAAATTT
---CCTTGGCGCAAGGTTCCCCACTACCTTGCTGCTCAATATCTTGGAGCATTTGTTGCC
TCTGGTGTTGTTTTCCTTGTGTACTGGGATGCTCTTGTC---TGGTACGAA---------
---CAT---GACCGAGGAGAATACCGCACCACTCCTGACACAGCCAGCATTTTCTCCACC
TACCCC---------TCGCCACACCTGACCTACATGGGCGGTATAGTGGACCAGGTGGTG
GCCACAGCCTTGCTACTCATCTGTGTCAGTGCCATCACGGACAGGCGCAACATGCAGGTG
TCAAAACAGTTGGTCCCTTTCTTCATTGGCCTCACTGTTCTTGGCATTGGAATCTGTTTT
GGCTTCAACTGTGGGTATGCTATCAACCCTGCCAGGGACTTGGCCCCAAGGTTGTTCACT
GCTCTATCTGGGTGGGGG---------CTGGAAGTTTTTGCCTTTTCCAGCCCCTGGTGG
TTGGTGCCGATACTTGGCACCCATGCAGGGGCTGTGCTTGGTGCCTGGGTGTACTATCTA
GCCATAGAGCTGCATTGGCACAAGGATGATACTGAG-
>GIVD01127952_Rhincalanus_gigas_Glp3
------------------------------------------------------------
------------------------------------------------------------
------------------------------------------------------------
------------------------------------------------------------
------------------------------------------------------------
------------------------------------------------------------
------------------------------------------------------------
------------------------------------------------------------
------------------------------------------------------------
---------TTGCTTCTACTTGCTGTCTGCGCTATAACAGACAGAAAAAATATGCAGGTG
```

AGCAAGCAGTTAGTCCCCTTGTTCATTGGTCTCACCGTGCTGGCTATTGGGGTCAGTTTT
GGATTTAACTGTGGATATGCCATCAATCCAGCCAGAGACTTCGCTCCTCGTCTTTTTACA
GCAATGTCCGGTTGGGGC---------CTCAGTGTTTTCACCTTCAGG---CATTGGTGG
TTGGTCCCAATATTCGCCTGTCATCTTGGAGCTATAGTTGGAGCCTGGTTCTACTATCTA
GCTATAGAGATGAACTGGCCTAAGGAAGACACAGAG-
>GIVD01137210_Rhincalanus_gigas_Glp2
------AAGAAAAAAAGATGCGGG------AGCATGAGGGTTCGTAAACCTTGGCTCAGA
GAGTTTATAGCAGAGATGTTAGGAACATTCGTCTTGGTCTTGATCGGTAATGGCTCTGTT
GCTCAGAATATTCTCTCGATTGGA---------------------CACAAGGGAAATTTT
TTCTCCGTCAACTGGGGGTGGGGTCTGGGAGTACTGCTAGGTGTGTGTATCAGTGGTGGA
GTATCTGGAGGTCATATCAATCCTGCTGTTACTGTAGCAGTGGCAACTTTGGGCAAATTC
---CCTTGGAGAAAGGTTCCTCATTACCTTGCTGCTCAATATCTGGGTGGTTTTCTTGCC
TCCTGTGTTCTTTTTCTTGTCTATTGGGATGCTCTTGTG---TGGTACGAG---------
---CAT---GAGCGAGGAGCCTACCGTACCACACCTGACACAGCCCTCATCTTCACTACG
TTCCCCGGT---

>GIVD01066048_Rhincalanus_gigas_Glp1
AAAGTAAAGAAA---GGTTGC---------AGTTTCAAAATCCGGAGCCCAGTTCTCAGG
GAGTTCTTAGCGGAGTTCTTGGGAACATTTGTGCTTGTAACCTTCGGAAGTGGCTCCATA
GCCCAGTCAACGTTGTCTATTGAG---------------------ACCAAGGGCAGCTTC
TTCTCTGTCAACTGGGGATGGGGTCTTGGAATAATGTTGGGCCTGCTGATCAGTGGTGGA
GTTTCTGGGGGTCATCTCAATCCAGCGGTATCGGTGGCTGTGGCTACACTGGGTAAATTT
---CCCTGGTGGAAGGTTCCTCATTACTTGGCTGCTCAATATCTTGGGGCTTTTACTGCT
TCCTGTGCTGTATTCTTAGTCTACTGGGATGCTCTTGTT---TGGTATGAA---------
---CAT---GACCGCGGAGTCTATCGAAGTATTCCGGATACTGCTAAAATTTTTGCTACT
TATCCT---------TCCGATCATTTGTCTTATGCTGGAGGAATATGGACCAGTTCTTG
GGTACAGCTCTCCTTCTCCTTGCAGTCTGTGCCATCACTGACAGGAGAAATATGCAGGTC
AGTAAACAGCTGATTCCCCTGTTTGTTGGTTTGACAGTTCTGGCTCTCGGAGTTGCTTTT
GGATACAATTGTGGATATGCCATCAACCCTGCCAGAGACCTAGCCCCGAGGATATTTTCT
GCCTTGGCTGGATGGGGA---------CTTGGAGTGGGTGTT------GGTGAGTGGTGG
TTGGTGCCAATTTTTGCATGCCATGCTGGAGCTGTGGCTGGAGCCTGGATCTACTATCTA
GCCATAGAGCTCAACTGGCCACAAGAGGAT---GAT-
>GFUD01122245_Neocalanus_flemingeri_Glp1

--ATCTTTGACCAGTTCCTG
GGCACCTTCCTCCTCCTCGTCTGCATCTGCGCCATCACCGACAAGAGCAACATGCAGGTT
GAGAAGCAGCTGATACCCCTCAGTGTAGGCTTCACAGTGCTAGGTCTGGGTATCAGCCTG
GGGTACAACTGTGGGTATGCAGTCAACCCTGCCAGAGACTTGGCGCCAAGAATTTTCTCA
GCTCTGGCGGGGTGGGGA---------CCTGGGGTGTTCACTGAATACAACCACTGGTGG
GTGGTCCCAGTCCTTGCCTGCCATGTGGGTGGTGTTGCTGGTGCCTGGGCTTACTATATT
GCGGTTGAGGTTAACTGGCCGGAGGAGGGAAATGAG-
>GFCI01389685_Pleuromamma_xiphias_Glp1v1
GAAAAGAAAAGAGGCAAA------------CGCTGGAAAATTACAAATAATGTTGTGCGG
GAGTTTTTGGCTGAATTTTTAGGAACATTTGTTTTGGTTCTGTTTGGCGATGCATCCATA
GCTCAAGCCGTCTTGTCTTTGGGA---------------------TCTAAGGGAGATTTC
TTTGCAATAAATTGGGGGTGGGGCATGGCAGTAATGCTCGGCGTGTTGGTGAGCGGCGGA
GTATCTGGAGGTCATCTCAACCCGGCGGTCACCGTGGCCGTCGCCACGCTCGGAAAGTTT
---CCTTGGTGGAAGGTCCCTCACTATCTGGCTGCTCAATACCTTGGAGCATTTACGGGA
GCAACTGTGGTGTTCCTCGTCTACTGGGATGCGCTTGTC---TGGTATGAA---------
---CAC---GACAGGGGACAATACCGCACGACCCCCGACACCGCGGGTATTTTCGCCACG
TACCCC---------TCCCCCACCTCACCATCGCGGGCGGGTGGGCGACCAGTTCCTG
GGGACCGCCATGCTGCTTATCTGCGTCTGCGCCATCACAGACAGGAAGAACATGCAGGTT
AGTAAACAAATGGTCCCTCTGCTGATCGGGTTGACGGTTCTGGCTATCGGAGTCTGCTTC
GGCTACAACTGCGGCTACGCCATCAATCCGGCGAGAGACCTCGGTCCAAGGCTATTCACT
GCCATTGCCGGGTGGGGC---------GGGCAGGTGTTCAGTTATTACAACCACTGGTGG
GTGGTGCCCGTGGTGGCGTCTCACGTGGGCGCCATTGTGGGCGCGTGGATCTACTTCCTC

GCCATCGAGCTCAACTGGCCCATCGACAACGACGAC-
>GFCI01389683_Pleuromamma_xiphias_Glp1_v2
GAAAAGAAAAGAGGCAAA------------CGCTGGAAAATTACAAATAATGTTGTGCGG
GAGTTTTTGGCTGAATTTTTAGGAACATTTGTTTTGGTTCTGTTTGGCGATGCATCCATA
GCTCAAGCCGTCTTGTCTTTGGGA---------------------TCTAAGGGAGATTTC
TTTGCAATAAATTGGGGGTGGGGCATGGCAGTAATGCTCGGCGTGTTGGTGAGCGGCGGA
GTATCTGGAGGTCATCTCAACCCGGCGGTCACCGTGGCCGTCGCCACGCTCGGAAAGTTT
---CCTTGGTGGAAGGTCCCTCACTATCTGGCTGCTCAATACCTTGGAGCATTTACGGGA
GCAACTGTGGTGTTCCTCGTCTACTGGGATGCGCTTGTC---TGGTATGAA---------
---CAC---GACAGGGGACAATACCGCACGACCCCCGACACCGCGGGTATTTTCGCCACG
TACCCC---------TCCCCCACCTCACCATCGCGGGCGGGGTGGGCGACCAGTTCCTG
GGGACCGCCATGCTGCTTATCTGCGTCTGCGCCATCACAGACAGGAAGAACATGCAGGTT
AGTAAACAAATGGTCCCTCTGCTGATCGGGTTGACGGTTCTGGCTATCGGAGTCTGCTTC
GGCTACAACTGCGGCTACGCCATCAATCCGGCGAGAGACCTCGGTCCAAGGCTATTCACT
GCCATTGCCGGGTGGGGC---------GGGCAGGTGTTCAGTTATTACAACCACTGGTGG
GTGGTGCCCGTGGTGGCGTCTCACGTGGGCGCCATTGTGGGCGCGTGGATCTACTTCCTC
GCCATCGAGCTCAACTGGCCCATTGACAACGACGAC-
>GCIT01009149_Tisbe_furcata_Glp3

GCCAGCGGGGCGCACCTCAACCCAGCAGTGACTCTTGCATTTACTGCAGTTGGTAAATTC
---CGATGGACCAAGATGTTCCACTACCTGTTGGCGCAGTACCTGGGCTCCTTTATTGGT
GCCGCCTTGGTCTACGGTTTCTATTACCAGGCCTTGGAA---GAGTGTGAAGAC------
---CACGGCAAGTGCGGCAACAAGGAA---GTCCCTGGCACTGCAGGAATCTTTGCCACA
TACCCC---------AAGGAATATGTCAGTCTGGGAGCGGCAATGGGAGATCAGATCATG
GTCACGTGCTTTCTTATGTTTTGTATTTGTGGAATCACGGAT------------------

>LR144051_Tisbe_holothuriae_Glp3
---AATCAGTTTTCCCGTGGATTG---AAAAGACTGCGAATCCGAAACCAGATTGCCAGG
GAGTTCCTATCCGAACTGGTCGGTAAGCTCATCCTCATAACCTATGGCGAGGGCGCCATC
TCCACCGAGAAGATCAGCGCCGGCCTCGGCGAA------------CCCAATGGCTCTGCA
GTGGCCACCAACCTCAACTGGGGCATCGCCGTCATGATCGCGGTCACCGTTGCCATGGGA
GCCAGCGGAGCGCATCTCAACCCTGCCGTGACTCTGGCATTCACTGCAGTTGGCAAATTC
---CGCTGGACAAAAATGTTCCACTACTTATTGGCTCAGTACCTGGGCTCTTTCATTGGT
GCCGCTTTGGTCTACGGGTTCTATTACCAAGCCTTGGAA---GAGTGTGAAGAC------
---CACGGAGAGTGCGGCGACAAGGAT---GTCCCTGGCACTGCCGGAATATTTGCAACA
TACCCC---------AAGGAGTATGTCAGTTTGGGGGCTGCCATGGCAGATCAGATTATG
GTGACATGCTTTCTAATGTTCTGTATTTGTGGCATCACTGATCCGAAAAATATCCGTATG
CCAACATATCTGTGGCCTCTCTACATTGGCTGCTTGGTGTTCAACTCAGGAACCTGTCTC
GGGTTCAACTGCGGTGCGGCTATGAACCCATCCAGGGATCTGGCGCCGAGGATATTCAGC
TCGATTGCAGGTTGGGGA---------AGTGAAGTGTGGTCGTATGGAAACCACTTTTGG
TTCGTGCCTATTGTTGGGAGTCACATTGGCGCTATACTTGGCGCGATCTTCTACTCACTA
GCCATTGAGAACCACTGGCCGGAATCTGAAAGGACA-
>GCIT01003867_Tisbe_furcata_Glp2

---CTCGGAGCCTTTGTTGGC
GCGGCCATCGTTTACGGAAACTTCTACCTAGCTCTGGAC---GAGTGCGAGACA------
---AATGGAACCTGTGCGGACAGGGGACGATCCCGGGGACGGCTGGAATCTATGCGACC
TACCCC---------AAGGAATATATGACCATAGGAGCGGCATTAGGGGATCAGATTCTA
GCTTCCAGTCTCTTTCTTCTTTGCGTGATGGGAATTCTGGATCAGAGGAATATGAAAATT
CCTCTCCGTCTT--

>LR144051_Tisbe_holothuriae_Glp2
---AACCCCATCGCCAGAGCCATC---CGCTCAGTGAGGACGGACAATGTCATCGTAAGG
GAGTTTCTCGGAGAGTTTATAGGTACGGCCCTTCTCATTGTCTTTGGCGAGAGCGCCAAC

TCGATCTTCGAGCTGAGCGCCGGAATGGGAATC------------CCAGTG---AAC---
GTGGCGCTCAACTTCAGCTGGGCCGTGGCCGTCATGCTGGCGCTGTGCGTAGCCGGGGGC
GCGAGTGGCGCACATATTAACCCATGTGTCACTGCAACATTCGTAATTACAGGTCGATTA
---CACTGGTCCAAGCTGTTCCACTATCTGCTGGGGCAGTACCTCGGCGCGTTTGTCGGC
GCGGCCATCGTGTACGGCAACTTCTACCTCGCTTTGGAC---GAGTGTGAGACG------
---AATGGGACGTGCCCGGACAAGGGGACGATCCCGGGCACGGCCGGGATCTACGCGACC
TACCCG---------AAGGAATACATGACCATTGGGGCGGCACTGGGAGATCAGATTCTC
GCTTCGAGTCTCTTTCTTCTTTGCGTCATGGGAATCCTTGACCAGAGGAATATGAAGATC
CCGCTCCGATTGGCGCCGCTCTACATCGGACTTGTCATCTTCAACATTGGATCTTGTCTG
TCGCTCAACTGCGGGGCAGCAATCAATCCGGCGAGAGATCTGGGGCCGAGGATTTTCACT
GCAGTTGCTGGTTGGGAT---------AATGATATTTGGACATACGGCCACAGCTTTTGG
TTCGTGCCCATAGTGGGCCCGCATATTGGCGGCATCCTCGGGGCAGTCGTCTACCTGCTC
TGCATTGAGAACCACTGGCCAAAGATAGAAGAAGAA-
>GCIT01007364_Tisbe_furcata_Glp1
---AATCCGATTGCAAAAGGACTT---CGGTGTCTCAGGATTAGGCAGAACTTTGTCAGG
GAGTTCTTTGCCGAACTATTTGGCACGTTCATCTTGATTGTATTTGGCGATGGCGTTGTC
GCCCAGGTCGTGCTCAGCGACGGCCTCGACGCC------------TCCGTCGGCAGCTTC
TTCACCATCAACTGGGGATGGGGCGTGGGCGTCACACTTGCAGTGCTGGTTGTAGGAGGC
GTCAGTGGGGCGCACATTAACCCG------------------------------------

---------------------AGATCT---GTCCCTGGCACTGCTGGAATCTATGCCACT
TACCCC---------AAGGACTTCCTGAGCATTGGTGGAGGAATGGCCGATCAGATTGTG
GGCACCATGCTTCTGCTCATCTGCGTGTGCGCTATTGGAGATCAGAAAAATATGCGCATC
CCCTCGTATCTTGCCCCGCTCTACGTTGGATTCACTGTGCTCAACATTGGGATCTGCTTC
GGATTCAACTGTGGCTATGCCATCAACCCTGCAAGGGATCTGGGACCAAGGATTTTCACG
GCCATCTCAGGTTGGGGC---------AGTGACGTTTGGAACTTTGGAGACAACTGGTGG
TACGTTCCTGTGGTTGGCCCTCATGTTGGTGCCCTCCTTGGTGCCCTGCTCTACATGCTC
TTCATTGAGAACCACTGGCCCAAGCACGAGACGCTC-
>LR138471_Tisbe_holothuriae_Glp1
---AATCCTCTGGCTAAAGGGCTT---CGATGCCTCAGGATACGGCAGAACTTTGTCAGA
GAGTTCTTAGCGGAACTATTTGGCACATTTATCTTGATTGTGTTTGGCGACGGCGTGGTT
GCCCAGGTGGTGCTGAGCGACGGCCTCAACGCC------------TCCGTCGGCAGCTTC
TTCACCATCAACTGGGGCTGGGGCGTGGGCGTCGCACTCGCAGTGCTGGTGGTCGGCGGC
GTCAGTGGGGCGCACATCAACCCCGCCGTCACCACTGCTATGGTGGTTGTAGGTAAGCTC
---CACTGGTCCAAGATGTTCCACTACCTCCTTGCTCAATACTTGGGCGCATTCCTTGGT
GCCAGCGTTGTCTATGGGGTCTATTACGAGGCTCTGGAA---GCGTGTGAGGCT------
---AATGGAACATGCGGGGCTAGATCT---GTCCCTGGCACTGCCGGCATCTATGCCACT
TACCCC---------AAGGACTTCCTGAGCATTGGTGGAGGGATGACCGATCAGATTGTC
GGCACCATGCTGCTGCTCATCTGTGTGTGCGCTATTGGAGACCAGAAAAACATGCGCATC
CCTTCGTATCTTGCCCCGCTCTATGTTGGCTTCACTGTGCTCAACATTGGAATCTGCTTC
GGATTCAACTGCGGATATGCCATCAACCCTGCCAGGGATCTGGGACCAAGAATCTTCACA
GCCATCTCCGGTTGGGGT---------AGCGACGTCTGGAGCTTTGGAGATAACTGGTGG
TACGTGCCTGTGATTGGCCCCCACGTGGGCGCCCTCCTCGGCGCCCTCCTCTACATGCTC
TTCATTGAGAACCACTGGCCCAAGCACGAGACGCTC-
>CM017373_Tigriopus_californicus_Glp2
---------ATGGGTCGAATCCTC---AGGACAATCCGAGTTAGGAATCAAGTGGCTCGG
GAATTCCTAGCGGAGTTCTTAGGAACCATGATCCTCATCCTCTTTGGCGATGGCGTTGTA
GCTCAAGTCACGTTGAGTGAAGGTCTGGGGATG------------CCTAAGGGCAATTTC
TTCACCATCAACTGGGGTTGGGGTTTGGGGGTTACCATGGCTGTGCTCATTGCGGGGGGC
GTGTCCGGAGCCCATATCAATCCTGCCGTCACTTTGGCCATGGCTGTTATCGGTAAGTTG
---CCATGGATCAAAATGTTCCATTATATGCTCGCTCAATATCTCGGAGCCTTTGTAGGC
GCAGCCTTGGTCCATGCTAATTATTACGAAGCCCTGGTT---GCATTCGAGACA------
---------GCTCAAGGC---GAAAGTCTTATCCCAGGCACAGCCGGGATCTTTGCAACC
TACCCG---------GCCGATTACCTTAGTAATGGGGGAGGATTTCTAGACCAGATCATC
GGCACTGCACTTCTACTCATGGTGGTTTGTGCTGTGGGTGACCAACGAAACATGAAGATC
TCGAGCCAAATGGCTCCTCTCTACGTCGGGTTCACGGTTCTAGCCATTGGCGTGTGTTTC
GGTGTCAATTGTGGATATGCCATCAATCCTGCCCGAGATTTGGGACCGAGGCTGTGGACA
TTGATCGTCCATTGGGGA---------ACGGGAACGTTTAGTTACGGAAACTATTGGTTC
TATGTTCCCATTCTGGCCCCTTATTTGGGCGGCATCATTGGGGCTGGACTTTATTTCGTG
TGTATTGAAAATAATTGGCCAGAGAAATTTGGA----
>GCHA01021727_Tigriopus_japonicus_Glp2
---------ATGGGTCGAATCCTT---AGAGCTATCCGAATCAGGAATGAAATGGCTCGG
GAATTCTTGGCGGAACTCCTTGGAACCATGATTCTTATTCTTTTCGGCGATGGCGTTGTC
GCTCAAGTGACTTTAAGTGAAGGTCTGGGAATC------------CCAAAGGGTGATTTC
TTCACTATCAATTGGGGTTGGGGATTGGGCGTTACCATGGCGATTCTCGTTACTGGAGGC
GTGTCTGGTGCTCATCTTAATCCCGCCGTTACTTTGGCAATGGTGGTTGTTGGAAAAATG
---CCATGGAAAAAAATGTTCCACTACATGCTTGCACAATACCTTGGAGCCTTCATAGGT

GCCGCTCTCGTCCATGCCAACTACTACGAAGCTCTTTCC---GCTTTCGAGGCT------
---------ACTCAAGGC---GAGAGCCTCATTCCGGCCACAGCTGGGATCTTTGCTACT
TATCCA---------TCCGATTATCTAAGTATTGGAGGAGGATTTCTAGACCAAATCATT
GGTACAGCCCTCTTACTCTTGGTTGTTTGTGCGGTGGGTGACCAACGAAATATGAAGATT
TCAAGCCAGATGGCGCCTCTCTACGTCGGGTTCACCGTTCTGGCCATTGGAGTGTGTTTT
GGTGTCAATTGTGGATATGCCATTAATCCAGCTCGAGATTTGGGACCGAGGCTGTGGACT
TTGATTGTTCATTGGGGA---------ACAGGGACGTTCAGTTATGGGAACTATTGGTTC
TATGTACCTATTCTAGCTCCTTACTTGGGAGGGATCATCGGGGCTGGGCTGTATTTCGTA
TGCATTGAAAACAATTGGCTTGAGAAAGTT-------
>JABCAI010000038_Tigriopus_kingsejongensis_Glp2
GGTGACATCATGAGCAAGCTGCTG---AAAAGAATCCGGATCCAGAATGAAACGGCCCGC
GAGCTACTGGCCGAGATGCTGGGGACCATGATCCTCATCCTCTTCGGGGATGGCGTAGTG
GCCCAAGTCACCTTAAGCGATGGCCTCAACATG------------GACAAGGGCAATTTC
TTCACCATTAATTGGGGGTGGGGCTTGGGCGTGACCATGGCCATCTTGGTGACAGGGGGC
GTGTCTGGGGCTCACCTCAATCCCGCCGTCACCCTTGCCATGGTAGTAACGGGTAAGATG
---CGCTGGACCAAGATGTTCCACTACATGCTGGCCCAGTATATTGGGGCCTTCCTTGGA
GCCGCCTTGGTCTACGCCAACTACCATGAAGCCCTGGCA---GCCTACGAGAAG------
---------TTCAATGGC---CCCGGCCTAATCCCGGAGACGGCGGGCATCTTTGCCACC
TATCCG---------GCCGACTACCTCAGCATTGGCGGAGGCTTTTTGGACCAGATCCTC
GCCACGGCACTCCTTTTGATAGTGGTGTCTGCCGTTGGGGACCCGAGGAACATGAAGATC
TCAAGCCAACTGGCTCCCCTCTACGTGGGCTTCACCGTCCTTGCCATCGGCGTGTGTTTT
GGAGTCAATTGCGGCTACGCCATCAATCCGGCCCGGGACTTGGGACCGAGATTGTTCACG
CTCATGGTGAATTGGGGC---------AAGGGCACCTTCACCTATGGTAATTCCTGGTTT
TACGTCCCCATTGTGGGTCCATATATTGGTGGAGTCGTGGGGGCGGTTCTATATGTTGTG
TGCATTGAAAATAACTGGCCGGAGAAATTT-------
>GHXK01261110_Platychelipus_littoralis_Glp2
AAACTAAATTTGGAACCTTTACTT---AAGAAAATTCGAATAAGAAATGATTACGCCAGA
GAATTCTTGGCCGAGTTTTTTGGCACTTGCATCTTGCTCACTTTTGGCAATGGGGTCGTT
GCCCAGGTCACTCTATCAGAAATGCTTGGAGACCCA---------ACATGGGGAAATTAC
TTAACCATAAGCTTGGCATGGACAGGAGGCCTCATGATGGGCGTGTTAATTGCTGGAGGG
GTGTCAGGTGCTCATCTCAACCCTGCAGTAACAACAGCTTTAATTTTAGTAGGCAAATTT
---CCCTGGTACAAAATGTTTCACTACATGTTGGGTCAGTACCTTGGGGCTTTTATTGGG
GCCTCTCTTGTTTATTTGACATACTTTGATGCCTTCAGG---GCCGTTCACGAT------
---------TTGAATGGT---------TTCACCCAAGACACTGCAGGCATTTTTGCTACG
TACCCA---------TCACCGCATTTATCTACGGGAGGAGGCTTTCTAGACCAGGTTGTT
GGGACTGCTCTTCTTCTGTTCTGTGTACTCGCAATTACTGATAACAAAAACATGAAGGTC
GGCTCATGGCTGATTCCATTGTACCTTGGATTTCTCCTCATCTCCATTGGCCTGGCCTTT
GGTTTCAACTCAGGCTTTGCTATTAACCCGGCAAGAGACTTGGGACCAAGAGTGTTCACA
GCCATGGCCGGATGGGGT---------CTGGATGTATTTAGATGGGGCAACCACTGGTGG
TGGGTTCCTGTGGTGGCTACTCACGTTGGCGGGGCCATTGGAGCGTGGTTATATGTCTTG
GGTATTGAAAATCATTGGCCTGTCGAGGCTGTGCAC-
>GHXK01158147_Platychelipus_littoralis_Glp1
AAATTCAACCTGGAGCCTTTGCTG---AAGAAATTTCGAATAAGAAATTTGCACTTCCGT
GAGTTTTTGGCCGAGATGTTAGGCACGTTCATCCTGATCACTTTCGGGGACGGGTCGGTC
GCCCAGGTCACTTTGTCCAGCTTGCTAAACGAT------------TCGAGGGGCAGCTTC
TTCTCAATAAACTGGGCGTGGGGCGTGGCCGTAATGTTGGGCGTGTTAACAGCAGGGGGC
GTGTCAGGAGCACACATCAATCCAGCCGTTACCACATCTCTAGTGCTTGTCGGCAAGTTT
---CCTTTGTACAAGATGTTTCACTACATCCTGGCTCAATACATTGGGGCTTTCTTGGGT
GCCTCTTGTGTCTACCTCAACTACTTTGAGGCCTTTGAA---GAAGTTCACATG------
---------AAGAAT------------TCAAAGTCCAAGACGGCCGGAATATTTGCCACT
TATCCA---------GTTGACCATGTCTCCATAGGAGGAGGATTTGTGGACCAGACCATA
GGGACAAGTCTCCTACTCTTCCTTATCCTTGCCATCACAGACAGAAGAAACATGAAGTTG
ACCTCCCTCCAAACGCCTTTGTACCTGGGCTTTGCCGTCTTGAACATCGGCCTGGCCTTC
GGGTACAACTGCGGCTACGCCATCAACCCGGCCAGGGATCTGGCACCTCGTCTCTTCACA
GCCCTGGCCGGGTGGGGT---------GTGGACGTGTTCACGTGGTCCAATCACTGGTGG
TGGGTGCCAGTCGTGGCCTGCCATTTGGGTGGAGCAATCGGGGCAATTGTCTACGTTTTG
GCCATCGAGAATCATTGGCCCGTTGAAGAA---CAT-
>CM017381_Tigriopus_californicus_Glp1
AAAAACATTGTCGCGGAAAAGATC---CGCAATGTGCGGGTTACCAACGATCTCGTCCGG
GAGTTCATGGCTGAATTTCTCGGTACCTTCGTTCTCATTCTCTTTGGCAATGCCGTCGTT
GCCCAAGTGGTTTTAAGTAATGAA---------------------GAGAAGGGCGATTTC
TTCTCCATCAACTGGGGATGGGGCGTGGCCGTGACCTTGGGCGTGCTCGTGTCCGGTGGA
GTTTCTGGAGGGCACATCAACCCCGCAGTTACATTGGCCTTGGCGGTCATTGGTAACACA
---CCGTGGAAGAAGGTCCCCTTGTACATGGCCGCCCAATACTTGGGAGGCTTTATCGCC
TCTGGCCTCTTGTACGGAATCTACTATGAGGCTTTGAAC---GCCTTTGAGGCT------
---------GAGAATGGATTGGATCGAGCCACCCCCGAAACGGCTGGCATTTTTGCCACC
TACCCT---------TCCCCTTTCCTCAGCCACCACAGTGGATTGCTAGACCAGGTGGTT
AGCACCGGTTTATTGATGCTTCTAATTTGTGCCATTTGCGACAAGAAGAACATGGGTGTG

TCCAAGGGTATGGCTCCACTCTACATTGGATTCATCGTGCTCAACATCGGCATCTGCTTC
GGGCACAATTGCGGCTACGCCATTAATCCAGCTCGAGATCTTGCCCCCCGCTTGTTCACA
CTTGTAGCTGGATGGGGC---------ACTAAACCGTTTACCATTGAAAACCATTGGTGG
GTCGTCCCGGTCATTGGAACCCATCTTGGGGCAGTCTTGGGTGCTCTTATGTATTGCTTC
TGCATTGAAAATCATTGGAAACAAAGCGCT---TAT-
>GCHA01010929_Tigriopus_japonicus_Glp1
AAGTATATTGTCGCGGAAAAGATC---CGCAATGTGCGGGTTACCAACGACCTCGTCCGG
GAGTTCATGGCTGAATTTCTCGGCACCTTCATCCTCATCCTCTTTGGCAATGCCGTCGTT
GCCCAAGTGGTATTGAGTAACGAG---------------------GAGAAGGGAGATTTC
TTCGCTATTAACTGGGGTTGGGGTGTGGCTGTGACTTTGGGCGTGCTCGTGTCAGGCGGA
GTTTCTGGCGGTCACATCAATCCCGCAGTTACCTTGGCCTTGGCGGTGATTGGAAATACA
---CCCTGGAAAAAGGTTCCCTTGTATATGGCGGCTCAATACTTGGGAGGCTTCGTCGCT
TCCGCTCTTTTGTACGGAATCTACTATGAAGCTTTGAAC---GCCTTTGAGGCT------
---------GAGAATGGTTTGGACCGAGCCACCCCTGAAACCGCTGGAATTTTTGCCACC
TATCCC---------TCCCCTTTCCTCAGCCATCACAGTGGATTGCTGGACCAAGTGGTT
AGCACTGGGTGTTTGATGCTCCTGATCTGCGCCATTTGCGACAAGAAGAACATGAGTGTG
TCTAAGGGAATGGCTCCACTTTACATTGGATTTATTGTGCTCAACATTGGAATTTGCTTC
GGTCACAATTGCGGCTACGCCATCAATCCTGCACGCGATCTTGCCCCCCGCTTATTTACA
CTTGTTGCTGGATGGGGA---------ACCAAGCCATTTACCTTCTCAAATCATTGGTGG
GTCGTCCCTGTCATTGGAACTCATCTTGGAGCTATTTTGGGCGCTCTTTTGTACTGCCTC
TGCATTGAAAACCATTGGAAAAAGAGCACC---TAT-
>JABCAI010000014_Tigriopus_kingsejongensis_Glp1
AAGTCTTTTCTGGGACAAAGGATC---CGCAACCTGAGGATCACCAACGAATGGGTCCGA
GAGTTCTTGGCCGAGTTCCTCGGCACCTTCGTCCTGGTCCTCTTCGGCACTGCCGTCGTG
GCCCAGGTGGTGCTGAGCAATGAG---------------------GAGAAAGGCGATTTC
TTCGCCATCAACTGGGGCTGGGGTGTGGCAGTGACTTTGGGGGTGCTCGTGTCCGGCGGC
GTGTCCGGGGGTCACATCAACCCGGCGGTCACCCTGGGATTCGCGGTCATTGGCAACTTC
---CCGTGGAAGAAGGTGCCCCTGTACATGGCAGCCCAGTACCTGGGGGGATTCGTGGCC
TCGGCCACGGTCTTTGGCGTCTACTATGAAGCTCTCAAC---GCCTTCGAGGAG------
---------TTTAACGGCTACAGCCGAGCCACGCCTCAAACTGCTGGCATCTGGGCCACC
TATCCC---------TCCGAGTTCCTGAGTCACCCCAGTGGGATCTTCGACCAGGTGGTG
AGCACGGCCTTGCTCATGCTCCTCATTTGCGCCATTATTGATAAGAAGAATATGAATGTC
GCCAAAGGCATGTACCCCCTGTACATTGGCTTCGTCGTCTTGAACATTGGGATTTGCTTC
GGGCACAACTGCGGCTACGCCATCAACCCCGCTCGTGACCTCGCGCCCAGGATCTTCACG
GCCATTGCTGGCTGGGGC---------ACGGAGCCTTTCACCCATGCCAACTACTGGTGG
GTGGTTCCCATCGTGGGCAGTCACATTGGGGCAGTTCTGGGCGCCCTGATCTACTGCTTC
TTCGTCGAGAACCATTGGAAGGAAACCTCT---TTC-
>ALA27400_Lepeophtheirus_salmonis_Glp3_v1
AATTTATCAGAAGGCATCATGATG---ACCAGTGTTCCTAAAGTCCCAAGTCTTGTACGA
CAATTTATGGCAGAACTATTGGGAACATTTTTAGTGGTATACCTTGGGGATGGAGCTATT
GCACAAGTTGTTGTCGGATCACAA---ACCTCACAGGCT---TTTTTTTTCTCTAATTTC
CTTTCAATTGCACTCGGCTATGGTTTTGCCCTAACAATAGGGATTTTTGTATCAGGTGGA
GTATCTGGCGGTCATCTCAATCCAGCGGTTACTTTAGCAATGGCCTCAATAAAAAAATGT
---AAATGGAAATGTGTTCCTGTATATATGTTAGCACAATATATTGGAGCATTTTTTGCA
TCAGCTATTCTTTTTGGGGTTTATAGAGATGGAATTATG---TTGGTTGAA---------
------------GGGGGCTACAATAAA---ACTGTCAAGACGCAAGGGATTTTTGCTTCT
TATCCAAGT---CCAAACATTAATGCTAGCATTGCTAGTTTAAGCTTTGATCAAATCCTT
GCCACAGCTTTGTTGATTATCATTATTCTTGCAGTCACAGATGGTAAAAATATGAAAGTC
ATATCAAGTCTTGTTCCTCTATCAATTGGATTGGGACTTACTGCCATTCATCTAAGTTTC
GGAGTTAATGCAGGAACAGCAATAAACCCAGCAAGGGATTTTTCTCCGCGTTTATTTACA
ATAATTGCTGGCTATGGA---------AATGAACCACTTACAACTGGGAATTACTTTTTC
TGGATACCTTGGATTCTTCCTCATGTTGGTGCTCTTCTAGGGGCTTTTATTTACGAGTAT
ATGATAGAACTTCATCATCCAATCGACGAA-------
>ALA27401_Lepeophtheirus_salmonis_Glp3_v2
AATTTATCAGAAGGCATCATGATG---ACCAGTGTTCCTAAAGTCCCAAGTCTTGTACGA
CAATTTATGGCAGAACTATTGGGAACATTTTTAGTGGTATACCTTGGGGATGGAGCTATT
GCACAAGTTGTTGTCGGATCACAA---ACCTCACAGGCT---TTTTTTTTCTCTAATTTC
CTTTCAATTGCACTCGGCTATGGTTTTGCCCTAACAATAGGGATTTTTGTATCAGGTGGA
GTATCTGGCGGTCATCTCAATCCAGCGGTTACTTTAGCAATGGCCTCAATAAAAAAATGT
---AAATGGAAATGTGTTCCTGTATATATGTTAGCACAATATATTGGAGCATTTTTTGCA
TCAGCTATTCTTTTTGGGGTTTATAGAGATGGAATTATG---TTGGTTGAA---------
------------GGGGGCTACAATAAA---ACTGTCAAGACGCAAGGGATTTTTGCTTCT
TATCCAAGT---CCAAACATTAATGCTAGCATTGCTAGTTTAAGCTTTGATCAAATCCTT
GCCACAGCTTTGTTGATTATCATTATTCTTGCAGTCACAGATGGTAAAAATATGAAAGTC
ATATCAAGTCTTGTTCCTCTATCAATTGGATTGGGACTTACTGCCATTCATCTAAGTTTC
GGAGTTAATGCAGGAACAGCAATAAACCCAGCAAGGGATTTTTCTCCGCGTTTATTTACA
ATAATTGCTGGCTATGGA---------AATGAACCACTTACAACTGGGAATTACTTTTTC
TGGATACCTTGGATTCTTCCTCATGTTGGTGCTCTTCTAGGGGCTTTTATTTACGAGTAT

ATGATAGAACTTCATCATCCAATCGACGAA-------
>ALA27399_Lepeophtheirus_salmonis_Glp2
------------------ATGATA---TTCGACGTTCCACGAGTTCGTAGCCTTATACGA
GTATTCCTTGCTGAATTATTAGGAACATTCCTACTCGTGTATCTCGGAGATGGATCCATT
GCTCAGACGGTTGTAGGAGGAAAA------TCATCTCCA---TTAATTTTCTCCAACTTC
CTTTCCATTGCTCTTGGCTATGGGTTTGCCCTCACCATTGGGATATTTGTGTCAGGTGGC
GTCTCAGGTGGACATTTGAATCCTGCCGTCACTCTAGCCATGGCATCCATCAAAAAAATG
---AAATGGAAACTTGTCCCAATTTACATGATAGCACAATATATTGGAGCGTTCCTGGCA
TCTGGGACTTTATTCGGCGTGTATAGAGATGGGATTATA---TTTGTGGAG---------
------------GGGGGCTATAACAAA---ACGGAGAAAACACAAGGGATCTTTGCTTCA
TATCCGAGT---GCGGACATTAATGCAAGTGTTGCGAGTCTCTGCTTTGATCAAATCCTT
GCCACAGCATTATTGATTATAATTATTCTTGCTGTCACAGATGATAAAAATATGAAAATC
ATATCAAGTCTTGTTCCTATATCAATTGGATTGGGACTCACTGCCATTCATCTAAGCTTT
GGGGTGAACGCTGGAACGGCTATCAATCCAGCTAGGGACTTTGCTCCACGATTATTCACG
TTCATTGTTGGGTATAGA---------AGTGGTCCCTTTGTGGCTGGAAAACACTTTTTC
TGGATCCCTTGGATCCTTCCTCATGTGGGTGGAGTTCTTGGAGCCTTGATTTATGAGTAC
ATGATTGAGCTTCATCATCCAATAGATGTA-------
>GAZX01018815_Caligus_rogercresseyi_Glp2_v1
------------------ATGATC---ATCGACGTTCCTCAGCTACCTAATTTGGTCCGA
CAGTTTATGGCGGAGCTTCTAGGGACCTTTCTTCTGGTGTATATGGGAGATGGAGCGATA
GCTCAAGTCGTCGTTGGATCACAGGCATCTTCCTCCGCATCTTTTTTCTTCTCAAACTTC
CTCTCAATCGCCATAGGCTATGGTCTGGCCCTAACAATGGGCATACTCGTATCTGGAGGA
GTCTCAGGAGGACACCTCAATCCCGCAGTGACCCTGGCACTTGCCGCCATTCGAAAGTGC
---AAGCTCATCTGCGTCCCCGTCTACATGCTGGCCCAATACATCGGAGCCTTTCTCGCA
TCTGCCGTTCTCTTTGGAGTATACCGGGACGGGATTTCC---CTTGTAGAA---------
------------GGTGGACTTAACAAA---ACCGCAAAGACGCAGGGGATATTCGCCTCC
TATCCCACG---GGAGCCATACAAGCCACGGGGCTCAGTCTGGCCTTTGATCAAATCCTA
GCCACTGCACTCCTCGTCATTATTATTTTGTCGGTGACGGATGGGAGGAACATGAAGGTC
ATTTCGGGGCTTGTTCCTATCTCCATAGGGCTTGGACTCACGGCCATTCATCTCAGCTTC
GGCATCAACGCTGGAACGGCTATTAATCCAGCAAGAGACTTTTCGCCACGGTTGTTTACG
TTACTTGCGGGCTATGGG---------ACTGAGCCTTTCCGGGCCTACAACTACTTCTTC
TGGATTCCCTTGATCCTTCCCCACATAGGCGGACTCCTGGGAGCCTTCATTTACGAGTAC
ATGATTGAGCTCCATCACCCAAAGGATGAT-------
>GAZX01018816_Caligus_rogercresseyi_Glp2_v2
AAGGAATCTGCAGTCACAATGATC---ATCGACGTTCCTCAGCTACCTAATTTGGTCCGA
CAGTTTATGGCGGAGCTTCTAGGGACCTTTCTTCTGGTGTATATGGGAGATGGAGCGATA
GCTCAAGTCGTCGTTGGATCACAGGCATCTTCCTCCGCATCTTTTTTCTTCTCAAACTTC
CTCTCAATCGCCATAGGCTATGGTCTGGCCCTAACAATGGGCATACTCGTATCTGGAGGA
GTCTCAGGAGGACACCTCAATCCCGCAGTGACCCTGGCACTTGCCGCCATTCGAAAGTGC
---AAGCTCATCTGCGTCCCCGTCTACATGCTGGCCCAATACATCGGAGCCTTTCTCGCA
TCTGCCGTTCTCTTTGGAGTATACCGGGACGGGATTTCC---CTTGTAGAA---------
------------GGTGGACTTAACAAA---ACCGCAAAGACGCAGGGGATATTCGCCTCC
TATCCCACG---GGAGCCATACAAGCCACGGGGCTCAGTCTGGCCTTTGATCAAATCCTA
GCCACTGCACTCCTCGTCATTATTATTTTGTCGGTGACGGATGGGAGGAACATGAAGGTC
ATTTCGGGGCTTGTTCCTATCTCCATAGGGCTTGGACTCACGGCCATTCATCTCAGCTTC
GGCATCAACGCTGGAACGGCTATTAATCCAGCAAGAGACTTTTCGCCACGGTTGTTTACG
TTACTTGCGGGCTATGGG---------ACTGAGCCTTTCCGGGCCTACAACTACTTCTTC
TGGATTCCCTTGATCCTTCCCCACATAGGCGGACTCCTGGGAGCCTTCATTTACGAGTAC
ATGATTGAGCTCCATCACCCAAAGGATGAT-------
>GGQW01003900_Tracheliastes_polycolpus_Glp2
AATATTTCAGATACGAATATCATG---CATTCGTCATTAACGCTTCCTCTTGCTGTTCGT
GAATTTCTCGCAGAATTTTTAGGAACATTTTTATTAGTCTTATTTGGAGACGGTGCAATT
GCACAAGCCATCATAGGTGGAAGTGTCGAAACTCGACCTGGA---TTCTTTTCCAGTTTT
CAGTCAATTGCTTTTGGTTATGGATTTGGACTCATGATTGGAATCTACGTTTCTGGTGGA
GTCTCTGGTGGTCATCTTAATCCCGCAGTTTCCTTTAGTATGGCTCTTGTCAAAAAATTG
---TCATGGATCAAAGTGCCCATTTACATGGCAGGGCAATATATTGGAGCATTTTTTGCC
TCTGCTGTTCTCTATGGAATTTATGCTGATGGAATTAAT---TACGTCGAA---------
------------GGAGATTTCAATCGT---ACATCAAAAACATTTGGAATATTTGCCTCT
TATCCAAAT---GAAACTTTAAATCCGTCTACAGTGACTTTGTCCTTTGGTCAAATGTTA
TCAACGGCTCTTCTTCTCATTATTATACTTGCTGTCACAGATGACCGCAATATGAAAGCT
ATTTCTGGATTAATTCCATTATCTATTGGACTAGGTCTAACAGCTATTCATATTAGCTTT
GGACTTAATGCTGGCTGTGCTATTAATCCAGCAAGAGATTTTTCACCACGATTATTTACT
TTAATGGCAGGATGGGGA---------ACAGAGCCCTTTACAGCAAACATGAATTTCTTT
TGGATTCCATGGATATTACCTCATATTGGTGGAAGTGTAGGAGCACTTGTATATCAAGCT
ATGATTAAACATCATTTGAGAGAAAATGAA-------
>GARW01029074_Eucyclops_serrulatus_Glp2

---TTTTCAGCAGAGCTTTTTGGAACTTTTTTACTGGTTTCACTTGGTTGTGGGTCTATT

GCTCAAGTAACTACAGGAAAACCTCCAGAA---------------TTCTTTTCAAACTTT
CAGTCCATTTGTTTTGGATGGGGATTCGCACTCATGACCAGCATTCTGGTCAGTGGAGGA
GTTTCTGGAGCCCATCTGAATCCGGCCGTCACCCTTTCTCTGGCTCTTCTAAGGCTTTGC
---GAATGGGAATTGGTACCAGCTTACTGGTTGGGGCAATATCTGGGAGCTTTTCTTGGA
GCCGCTAATGTCTATCTCATTTACAATCATTCGATCGAT---CTAGCT------------
------------GGCGGG------AAA---ACCAATGCAACGAGAGCAATTTTTGGAACC
TATCCCAAC---CATGCTTTGGAGCCTTCAACTTTAACTCTGGCTTTTGACGAAATGCTT
GGTACTGCTCTACTTGTAGTCGTCATCATGGCTGTAACTGATAAGCGCAATTTGAACTTA
CCATCGAAGATGGCACCACTGTACATTGGGCTAGGGCTTTGCGGGATCCTGATGAGCTTT
GGAATGAACTCAGATTGTGCTCTCAATCCAGCCAGGGATTTTGGACCGCGATTGTTCACT
TTGATAGCTGGCTGGAGTGCC------GAGGATACCTTTGTTGACGGGAACTACTTCTTC
TGGATTCCGCTCATTCTCCCGCACGTTGGAGGACCAGTTGGAGCGGCTGTGTACTACGGG
ATGATTTCCTACCACCATCCGGAGGACGATGAC----
>LR117534_Apocyclops_royi_Glp4

------------------------TACTGGTTGGCTCAATACGTTGGAGCGTTCTTCGCG
GCTTCATTGGTA---------------GACGCGATATTA---TTTTTGTCT---------
---------ATCAAAGTT---TTGTATTTTTAGATGCAACAGCGCCGATATTCGCAACA
TACCCGAAT---GGCGAATTGCAACCTTCAACCCTGACGGGGGCGTTCGACGAAGTCGTG
GGATCCGCTCTTCTCATCTTGATCATTGCATCCGTCACAGACAAACGGAATATGAAAATT
TCTGAAGAACTTGTTCCGTTGTACATCGGCTTTGGACTGTCTGCCATCCTGATGTCCTTC
GGGATGAACTCGAACTGTGCCCTGAACCCCGCTAGAGATTTTTCACCGCGCTTGTTTTCA
CTCATTGCTGGATGGAGTGCC------AAGGACTCCTTTCAAGCGGGAAGCAACTTTTTC
TGGATTCCCTTGTTCCTACCACACCTCGGGGCAACCATTGGAGCTCTTTTGTATTATGGA
CTCGTCGGAATTTTGTTTCCAAAAGAAGAAATTAAC-
>LR126277_Apocyclops_royi_Glp3
------------------ATGATG---GTTGAT---AGATTA------CTACTTCTCAGA
CAGTTTTTGGCTGAATTTCTCGGAACTTTTGTTTTGGTGCTTATGGGATGTGGTTCCATT
GCTCAGGCCATTACAGGTAAACCAGACTCT---------------TTCTTCCACCACTAC
TTCTTCATCTGCTTCGGGTGGGGCTTCGCGCTCATGATTGCCATCATCATCAGTGGCGGT
GTCTCAGGTGGGCACGTCAATCCAGCGGTTACCTTTGCTATGGCAATCTTCAGAAAATGT
---TCATGGATTCAGGTACCAGTTTATTGGATAGCGCAATATCTTGGCGCGTTTCTGGCC
GCG---

------------------------------------ACACTTGCTTTTGACCAGATTTTA
GGCACTGCCCTATTAGTGATCATCATTCTCGCGGTGACCGACAAGAAGAACATGAACATC
CCATCAAACCTCTATCCACTTTTCATTGGATTGGGTCTTTGTGCAATACATCTCAGCTTT
GGTCTGAACGCAGGTTGTGCGGTCAACCCAGCAAGAGACTTCTCACCGCGTTTATTGACC
CTCATCGCCGGCTGG------------GACAGCTCTCTCACCTTTGGAAACTACTTTTTC
TGGATTCCTTGGCTTCTGCCACACATCGGAGGTGTGGTTGGAGCACTAACCTACTATGTG
ATGGTTGCCATTCACCATTCACCTGTCGACCAA----
>LS049409_Acartia_tonsa_Glp3
------------------------------ATG---CGGATATCCAACGTATATGTTCGT
AATTTCCTGTCCGAGTTCCTTGGAACCTTTGCCCTTGTCATCTTTGGAGACGGAGCCGTC
GCACAGGTGGTCCTTGGTTCAAGTTTGGGTGCTGCT---------GATTTTGGAGGTTTT
CTGAATATTTGCTTCGGCTATGGTTTTGCTCTCATGATTGGAATTCTCATAAGTGGTGGT
GTCAGTGGTGGTCATCTAAACCCAGCTGTCACTGCCTCTATGGTTGTTCTTAGAAAACTG
---AAGCCTGTCATGGCCCCTGTTTACATTGCTGGACAATTTCTTGGTGCATTTTTTGCA
GCTCTGGTCCTGTGGGGAGTTTATGCCGATGCTATTCAC---ATCGCTGAGAAAGCCATT
CTTGTA---GATGGTTCTGAAGTGAAGTACACAATGGCCACTGCTGGTATTTTTGCTTCT
TATCCTATG------AATAATGAGATTAGCACTGCCTGTCTGGCCTTTGACCAGATCCTT
GGAACAGCTCTGCTTGTGATCATCATCCTGGCGGTCACTGATGGCAACAACATGAAGCCC
AACTCTGGTCTTATCCCTCTGCTCATTGGTCTTGGCCTTGCAGCTATTCATCTTAGCTTT
GGATTGAATGCTGGAGCTGCCATTAATCCTGCCAGAGACCTGGCTCCCAGAATCCTCTCT
ATCATGGGCGGGTGG---

>GBGO01056315_Eurytemora_affinis_Glp3
------------------ATGGGG---AAACTA---GGAATATCAAACAGTTATGTCAGA
AATTTCTTGGCTGAGCTACTGGGAACATTTCTCCTGGTAGTTTTCGGGGATGGGGCAATA
GCTCAGGTCACACTAGACAGA------------------------GAT---GGCGGCTTC
CTGAACATTTGTATTGGATACGGTTTAGCACTCATGATTGGTATCCTGGCCAGTGGCGGG
GTCAGTGGTGGTCACCTAAACCCAGCAGTGACAGTAGCAATGGCTCTTCTTGGAAAAGTT
---AAACTACTACAGGTCCCTGTTTACATTCTTGGACAGTTCATTGGAGCATTCCTGGCC

GCAGCGGTAGTGCTGGGTGTCTATGCTGATGCTATAAAT---ACTGCAGAT---------
---------------------GTTAATTGGAGTTTAACTACTGCTGGAATATTTGCTTCC
TATCCTAGG------TTTAATACGGTGAGTACAATAACCCTGGTGATGGATCAGATCCTT
GGAACAGCTCTCCTTCTTATTATTATCTTGGCTGTAACAGATTCAAGGAACATGAAGGTA
AATCCGGCACTGTTTCCTCTTTTTATTGGACTTGGTCTGACAGCAATTCACATAAGTTTT
GGTCTGAATTCTGGCTGTGCAATTAATCCCGCAAGAGATTTTGCTCCTCGTCTTCTTTCA
TTGATCGCTGGCTTCGGA---------ACAGAAACTTTCACGGTTGGCAACTATTTCTTC
TGGATTCCTCTCCTGATGCCGTTTCTCGGAGCTGCGATAGGAGCTGCCATTTATCAACTC
ATGGTCGCCCTTCATCATACAGACGAGGAA-------
>GGQN01116754_Temora_longicornis_Glp4
------------------ATGAAA------------GGAATAAAAAACAGTTATGTTCGA
AACTTTTTGCTGAGTTCCTCGGGACCTTTGCCCTCGTGGTCTTCGGGGATGGGGCAATA
GCTCAGGTGGTTCTGGGTAAATCTGTGACTGGTTCTGAT------TTTTTTGGTGGCTTT
TTGAATATTTGCTTTGGCTATGGCCTCGCACTAATGATTGGCATCCTCATTTCTGGTGGA
GTCAGTGGAGGACATTTGAATCCAGCCGTTTCTCTGGCCATGGTGTGCGTCAAGAAGCTC
---AAGATTACCCAGTTGCCCGTTTACATGGCTGCCCAGTTTCTGGGGGCCTTCGTTGCC
GCGCTGGTGCTCTGGGGCAACTACGCAGACGCAATCAGA---ATACACGAAAACAATCTC
TCAGGCTCAGAGTTTGTG---TCTGATTATTCTGGCGCCACCATGGGAGTGTTCTCCTCC
TTCCCAAAC------AATCAAATGACGAGCCTCACCACCCTCGTCATGGACCAGATCCTC
GGCACCGCCCTCCTCGTCATCATCATCCTTGCTGCCACAGACTCTAAGAACATGAAGTTG
GCGTCTGGATTAGTGCCCCTCACGATTGGTCTGGGGCTCACAGCTATACATATCAGTTTT
GGGCTGAACGCTGGATCTGCGGTCAACCCGGCCCGGGACTTCTCCCCGCGGTTGCTGACG
GCCATGGCGGGCTGGGGA---------GAACTGCCGTGCACGGCCTACGACTACTGGTTC
TGGGTGCCACTGGTGATGCCTTTCATCGGCGGCCCCATCGGGGCGGCCATCTACACCCTC
CTCATTGAGGTCCACCACCAACAAGAATTT-------
>GFWO01129170_Labidocera_madurae_Glp4
---------------------------------------TTAAAAAGCAGTTATCTGAGA
AATTTTTTGGCTGAGTTCCTTGGAACCTTTGTTCTAGTCGTGTTCGGAGATGGAGCTATT
GCACAGATTGTCTGTGGT------------GGTCCCTCA---------TTTGGAAACTTT
CTCAACATATGCTTTGCCTACGGCCTGGGACTGACAATGGGAATTCTAGTCAGTGGTGGA
GTCAGTGGCGGTCATCTGAACCCAGCTGTTAGTGTGACAATGGCAGCTTTGCGGAAGTTA
---GACATAAAACAGCTTCCGGTGTATGTACTCGGACAGTTTCTGGGGGCATTCTTTGCC
GCACTTGTGATCTGGGGAATCTACGCGGACGGAATCTAC---TTCAGAGAG---------
---GGA---GAGACTTTG------GATTATACAGCCTCTACCGTGGGAATTTTTGCCTCA
TATCCAAGC------CTCCTCAAGGCCAACCTCGGCGTGCTCGCGATGGACCAGATCCTG
GGCACGGCGTTGCTGCTATTGATCATCCTCGCCGTGACGGACGAAAGAAACATGAACGTC
AACTCCTCCCTCGTCCCCCTCCTCATCGGGCTCGGCCTCACCGCCATCCACCTCAGTTTC
GGGTTTAATGCTGGCTGTGCGATCAATCCAGCCAGAGACTTTGCTCCCAGACTTGTCTCT
CTCATGGGAGGATGG------------CCCAACACATTCACGGGTGAGGGAGGATGGTGG
TGGATTCCAACCTTCCTTCCATTCGTCGGCGGCCTAATCGGCGGTGCTGCCTACCACTTC
ATGATCGAGATTCATCACAAGGACGAC----------
>GCIW01018278_Hemidiaptomus_amblyodon_Glp3
GAAACTGATCCCGCCGCAGCCATC---TCGGGCTGGTCAACATACCGGAGCTTCCTGCGC
ATCTTCCTGGCCGAGATCCTTGGCACCTTCCTCCTCGTCCTCATCGGCGATGCCGCTGTT
GCCCAGGTAGTTCTGGGCGAACAGCTGAAGAAGACG---------ATATTCGGAGGCTTC
CTCAGCATCTCTGTCGGCTTCGGGCTGGCTCTGATGGTTGGCATCCTCGTCGCGGGCGGT
GCCAGCGGAGGCCACCTAAACCCGGCAGTGACGATGGGCTTGGCGGCCGTGGGACGAGTC
---CGCCCCCTCCTGGTCCCCGTGTACTGGGCAGGCCAGTATCTGGGCGCCTTCCTGGCT
TCTCTCGTCCTTTGGGGTGTCTACAGCGAGACAATCCTG---CTTGCTGAG---------
------------CCCACC---GGAGTGTACAGCGCCAATACCCGGGGTATATTTGCCTCC
TACCCCATG------ATGGACCAATACTCGATGGGGGTGCTGGCCGGGGATCAGATCCTC
GGCACCGGCCTCCTCCTCATCATCATCTGCGCGGTGACGGACCCCGGCAACCTCAGGATC
AACCCCTCCCTCGTCCCTCTTTACATTGGGCTCGGGCTGACCTGCATCCATCTGGGCTTC
GGCCTGATCGCTGGCTCGGCCATCAATCCGGCACGGGACCTAGCTCCCCGCGTCCTCTCC
GCCCTGGCCGGCTGG------------CCGGGGACCTTCACAGCCGGGAACTACTGGTTC
TGGATCCCCTCGCTGCTGCCCCACCTGGGCGGACTCCTGGGGGCCGGCCTTTACAAGCTA
ATGATTGAGCTGCCCCACCCTCCGGAGGAACTGGCA-
>GCIW01018085_Hemidiaptomus_amblyodon_Glp2
------------GGGCTCCTCTCC---GCGGAAATGTGCCAAACCAACAGTTTTTTGCGG
GTCTTTCTGGCCGAGACACTCGGCACCTTCCTCCTTGTCCTCATCGGAGATGCCGCCGTC
GCCCAGGTTGTCCTTGGCGAACAGTTGAACAAGGCG---------ATATTTGGGGGATTC
CTCAGCATCTCCGTCGGATTTGGTCTGGCCCTCATGATCGGCATTCTCGTGGCCGGAGGA
GCTAGCGGTGGTCACCTGAACCCGGCGGTAACGCTGGGCCTGGCGGCGGTGGGACGACTC
---CGCCCCATTTTCATCCCCGTGTACTGGGCGGCCCAGTACCTGGGCGCCTTCCTTGCC
GCGGCTGTGGTCTGGGGCATCTACAGCGATGCCATCCAC---CTGGCCGAG---------
------------CCTGGT---GGCCTCTACAGCAACAGCACGAGGGGGATTTTTGCCTCC
TACCCCATG------GATGATCAGTACTCCGCTGGGGTGCTGGTTGGCGACCAGATTCTG
GGCACAGGCCTGCTTCTCCTCATCATCTGCGCCGTGACGGACCCCAGCAACCTCAGGATC

AACCCCTCCCTTGTCCCTCTCTACATCGGGCTCGGGCTCACCGCCATCCACCTCGGGTTT
GGCCTGATAGCCGGGTCAGCTATCAACCCGGCACGTGACCTCTCCCCCCGCCTCCTCACG
GCTATGGCGGGCTGG------------CCGGGCACCTTCACGGCTGGGAACCACTGGTTC
TGGATTCCCTGGCTGATGCCTCACCTGGGAGGCCTCCTGGGCGCCGGCCTCTACAAGTTC
ATGATTGAGTTCTACCATCCTAAGGACAGCTTGAAC-
>GCIW01021548_Hemidiaptomus_amblyodon_Glp1
---------------------------------ATGCTTAAACTACACAGCTATGTGCGG
CAGTTCCTGGCTGAGCTGTTGGGAACGTTTGCCCTTGTCATCTTAGGTGATGGTGCTGTA
GCACAGGTTGTATTGGGGCCCGGAGTGAAGGACGCCGTG------TTTTTTGGCGGATTC
CTGAACATCTGTTTCGGCTACGGACTCGCACTCATGATTGGAATCCTTATCAGCGGAGGC
GTTAGTGGAGGTCACCTGAACCCTGCAGTTACCCTGGCCATGGTGGCTGTGAGAAAGTTG
---AAGTTAATACAGCTCCCTATATACTGGGCTGGCCAATACCTGGGTGCCTTCCTGGCA
GCACTTGTCCTCTGGGGCAACTATGCTGATGCTATCCAC---CTGACGGAG---------
------------GTAAAT---GGAGATTATTCAGCAGCCACTAGAGGGATTTTTGCTTCA
TATCCAATG------AATGACAAGATCTCAACGGTGACGCTGGCCATGGACCAGATGCTG
GGCACCGCGCTCCTCGTCCTCATCATCTCTGCCGTCACCGACTCGAGGAACATGAGGGTC
AGCTCCTCCCTCGTCCCCCTCTTCATCGGACTGGGGCTCACCTCCATTCATATAAGCTTT
GGCCTGAACGCGGGAGCTGCCATCAACCCGGCACGTGACCTGGGGCCACGGGCCCTGTCC
GCTCTGGCGGGCTGG------------CCCGGCACCTTCACGACAAACGAGAGCTGGTTC
TGGATTCCCTGGCTGTTGCCCCACCTGGGTGGGGTGCTGGGGGCCCTCCTCTACGTCTTC
GTCATTGAGCTGCACCATCCAGCTGAGGAGGACGTC-
>GHWV01089710_Pseudodiaptomus_annandalei_Glp3
------------------ATG------------------AAACTGCCGAGTTTGGCGCGA
GAGTTCTTCGCCGAGTTCCTCGGAACTTTTGTCCTTGTCCTTATGGGTGATGGCGCCATT
GCACAGGTCAGCTTGGGTAACAGCCTC---GGTGCCAAC---------ATGGGCAACTTC
CTCAACATCGCCATTGGCTACGGGTTAGCCCTCATGATTGGAATCCTTGTCAGCGGTGGC
GTCAGTGGAGGTCATTTGAACCCAGCCGTCAGCCTGTCCATGACAATCATCGGCAAGCTG
---AGGCCGATTCAGCTGCCGGTGTACATGATTGCCCAGTATCTGGGAGCATTTGTGGCA
GCTTTGGTGCTATGGGGCAACATTTCCAGTGCACTTTAT---TTGGTTGAG------TGT
GAAGGGCTGGACTTCAAGGACTGCAACTACACTATGGGCACGGCCGGCATCTTTGCCTCA
TACCCGATC------ACCGAGGAGCTGAGCTCTGCCAACCTGGTTGTGGATCAGATCCTT
GGCACTGCCATGCTTGTACTCATCATCCTGGCTGTTACAGACTCACAGAACATGAAAGTG
TCGTCGGGCCTTGTGCCTCTGCTCATTGGACTCGGTCTCACCTCCATCCATCTCAGTCTT
GGCCTGAACTCAGGGTCAGCGGTCAACCCGGCACGTGACTTTTCGCCCCGACTTGTCACT
CTCATT---GGCTGGGGC---------ACTGAGACATTCACGGCAACTGACAGTTTCTTC
TGGATTCCGTGGATTTTGCCGCACGTCGGCGGCGTTCTGGGCGCCGTCGTGTATTATCTC
ATGATCGGCTTGCATCACCGAGAAAACGACGACTTT-
>GGQN01050493_Temora_longicornis_Glp3
------------------------------ATGAGGTTTCACCTCCCAAAGCTTTTTCGG
GAGTTTTTAGCAGAATTTCTGGGAACTTTTGTACTGGTTCTCCTGGGGGATGGATCGGTA
GCCCAGGTTGTCCTGGGAAGTGCTGTAAAGCCAGATCAG------TTCTTTGGCGGATTT
TTAAACATTGCGTTTGGGTACGGGCTAGCCCTCATGGTCGGAATCTTAGTCAGTGGCGGC
GTAAGTGGCGGTCACTTGAACCCTGCCGTAACTGTGTCCATGGCTTGCATCAAAAAGCTT
---AAGTGGAAGCAAGTCCCTGTGTACATGGCTGGACAATATCTGGGGGCATTCATAGGA
GCTCTAGTTCTGTGGGGAGTCTACGCAGACGCAATCACC---ATTGCCGAA---------
------------GGAAGC---AATGACTACACTTCAAAGACCTATGGGATTTTCGCCTCT
TATCCATTC------TCTCCTAAGACAAGCCTGGTCACCCTTGCCATGGACCAGATGTTG
GGGACAGCCATGCTAGTTCTCATCATCCTGGCGGTCACTGACGACCAAAACATGAAAATA
TCTGCCTCCCTTGTTCCCCTCCTCATTGGTTTAGGGCTAACATCAATCCATTTAAGTCTT
GGTCTGAATGCTGGGTCTGCTATCAACCCTGCTCGCGACTTTTCTCCTCGATTGCTCTCT
CTGATGGCAGGATGGGGA---------GAAGAAACATTCTCGGTGGACGGAGGTTGGTTC
TGGATCCCGTGGTTGTTGCCGCACCTTGGGGGTATAGTCGGGGCCCTGGCCTACCAGATA
ATGGTCCAGCTTCATCATCCAGCTGATGATCTGAAG-
>GFWO01072462_Labidocera_madurae_Glp3
TCAACCGTCGAAGAGGAAAGAAGA---AAAATGCGGTTCAAGATTCCCAAATTAGCGAGA
TCATTTCTGGCCGAACTTATTGGAACATTTGCTTTGGTTTTGCTTGGTGATGGAGCTGTG
GCCCAGGTCGTCTTAGGACAGGGTATAAAGGGAGATTAT------TTTTTTGGCGGTTTC
CTGAATATTGCTATTGGATATGGGCTAGCTTTGATGGTTGGAATTCTTATCAGTGGTGGA
GTTAGTGGTGGACATTTAAACCCTGCTGTGACCGTTGCTATGGCATGCTTGAAGAAGCTG
---CGACTGGTGGAGGTTCCAGTATATATGGTCGCACAGTACGTAGGCGCATTCGTCGCA
GCTCTTGTTCTGCGGGGAGCGTACGCTGACGCAATACGT---ATAACTGAG---------
------------AAAACT---ACCAACTACACTTCAGCTACATACGGAGTATTTGCATCC
TACCCTTCC------TCGAGTGAGATTGGCATGGTCAGCTTGGCAATGGATCAGATGTTG
GGTACAGCTATCCTGGTGCTGATCATCCTCGCTGTCACGGATCCCAAGAACATGCAGATT
TCCTCCTCCCTGGTCCCCCTCCTCATCGGACTCGGGTTGACCTCCATCCATCTCAGCTTT
GGATTGAATGCTGGGTCAGCGATCAATCCAGCTCGAGACTTCTCTCCAAGACTGCTCACA
GTTCTATCTGGATGGGGA---------ACTCAACCTTTAACGGCTAATGACTACTGGTTC
TGGATTCCCTGGCTTCTGCCTCACATCGGAGGTATAGTCGGAGCACTGACCTATGAGATT

CTAATCGAACTGAACCATCCTGAAGACGACTTAAAA-
>GFUD01027877_Neocalanus_flemingeri_Glp7
------------------ATGAAG------ATTGGAAAGATTACCAACCAGTATGTAAGG
GAGTTCCTGGCGGAGACCTTGGGCACTTTTGCTTTGGTCATCTTTGGAGATGGTGCTGTG
GCTCAGGTGGTGCTGGGCAATGCTGCCCGAGGCAACTCA------TTTTTCGGAGGATTT
CTGAATATCAGTTTTGGGTACGGACTGGCCCTGATGATTGGTATTTGTATTAGTGGTGGA
GTGTCCGGAGGTCATCTCAACCCTGCTGTCACGCTGGCCATGGCGGTTTTCAAGAATCTG
---AAATGGATCCAGGTACCTATCTACATGGCTGGCCAGTACCTGGGGGCATTCCTTGCA
GCTCTGGTCCTCTGGGGAGAGTATGCTGATGCTATCAAG---ATTGTGGAGCTAGGCCAG
GATCCT------GCCAACTCTGGCAGCTACACCAGCGCCACACACGGGATATTTGCATCT
TATCCTTTCTTTGGCACCAGCCAGGTCACCACCTTGAGCCTGGCCATGGACCAGGTGCTG
GGGACAGCCATGCTGCTCATCATCATCCTGGCGGTGACCGACAACAAGAACATGAAAATC
CAGAGTAGCCTGGTACCCCTCACCATAGGGCTGGGACTGACTGCTATCCATCTCAGTTTT
GGCCTTAATGCCGGGTCTGCAATCAATCCTGCCAGGGATTTCGCTCCCCGCGTGCTGACT
ATCCTTGCTGGGTGG------------GAGGAGCCGTTCAAGGCTGCTTCCTCCTGGTTC
TGGATACCTTGGCTGCTTCCTCATGTGGGAGGAGTGCTGGGAGCTGGACTGTACAAGGTG
ATGGTGGGGATGCATACCAAGGATGGGGAG-------
>GFUD01005420_Neocalanus_flemingeri_Glp6
------------------ATGAAG------ATTGGAGAGGAAACTAACCAATATGTAAGA
GAGTTCCTGGCGGAGACCCTGGGAACTTTTGCTTTGGTCATCTTTGGAGATGGTGCTGTG
GCTCAGGTGGTGCTGGGCAATGCTGCCCGAGGGGACACA------TTTTTCGGAGGATTT
CTAAATATCAGCTTTGGGTACGGACTGGCCCTGATGATTGGTATTTGTATCAGCGGAGGT
GTGTCTGGAGGTCATCTCAACCCTGCCGTCACGCTGGCCATGGCGGTTTTCAAGAATCTG
---AAATGGATTCAGGTGCCTGTCTACATGGCTGGGCAGTACCTGGGGGCATTCCTTGCA
GCTCTAGTCCTCTGGGGAGAGTATGCTGATGCTATCAAG---ATGGTGGAGCTAGGCCAG
GATCCA------ACCAACTCTGGCAGCTACACCAGCGCCACACACGGGATATTTGCATCT
TATCCTTTCTTTGACACCAGCCAGGTCACCACCTTGAGCCTGGCCATGGACCAGGTGCTG
GGGACAGCCATGCTGCTCATCATCATCCTGGCGGTGACCGACAACAACAACATGAAAATC
CAGAGCAGCCTGGTACCCCTCACCATAGGGCTGGGACTGACTGCTATCCATCTCAGTTTT
GGTCTGACAGCCGGGTCTGCCATCAACCCTGCCAGAGACTTCTCACCCCGATTACTGACT
CTCCTAGCTGGGTGG------------GAGCAACCATTCAAGGCTGCCCACTCCTGGTTC
TGGATACCTTGGCTGCTCCCTCATGTGGGAGGAGTGGTGGGAGCCCTCCTGTACCAGTTT
ATGATTGGTATTCACCACCCTATGGAGGAC-------
>GHLB01002221_Neocalanus_flemingeri_Glp5
------------------ATGAAG------ATAAATGCAAACACCAACCAACATGTGAGA
GAGTTTCTAGCTGAGACTTTGGGTACCTTTGCTTTGGTCATCTTTGGTGATGGTGCTGTG
GCCCAGGTGGTGCTGGGCAATGCTGCCAGACAAGACACG------TTTTTTGGAGGATTT
CTGAACATCAGTGTTGGGTACGGGCTGGCTCTGATGATTGGTATTTGTATCAGTGGAGGA
GTGTCAGGGGGGCATCTCAACCCTGCCGTCACGCTAGCCATGGCGGCTATTAAGAATCTG
---AAACTAATCCAGGTACCTGTCTACATGGCTGGACAGTACCTGGGGGCATTCCTAGCA
GCTCTAGTCCTGTGGGGAGAGTATGCTGATGCTATCAAG---ATGGTGGAGGTGGTCGTC
TCTCCC------ACCAACTCCAGCAGCTACACGAGCACCACACACGGAATATTTGCATCT
TATCCTTTCTTTGATACAAATAAGGTCACCACCCTGAGCCTGGCCATGGACCAGGTGCTG
GGGACAGCCATGCTGCTCATCATCATCCTGGCAGTCACTGACAGCAAGAACATGAAGATA
GAGAGCAGCCTGGTACCCCTGACTATTGGGCTTGGCCTGACTGCTATCCATCTCAGCTTT
GGTCTGACAGCCGGGTCTGCCATCAACCCTGCCAGAGACTTCTCACCCCGGTTACTGACT
CTCCTAGCTGGGTGG------------GAGCAACCATTCAAGGCTGCCCACTCCTGGTTC
TGGATACCTTGGCTGCTCCCTCATGTGGGAGGAGTGGTGGGAGCCCTCCTGTACCAGTTT
ATGATTGGTATTCACCACCCTATGGAGGAC-------
>GBXT01029481_Calanus_glacialis_Glp6
ATGAAAAGGCTGAAGATCAGGGATAATAGGTTTCGGACT---GGCAACTGCTTCCTGCAG
AAGTTTCTGGCCGAGTTGCTGGGGACCTTCACCCTGGTCCTGCTTGGTGATGGAGCTGTA
GCCCAGGTGGTGCTGGGCAATGCTGCCAGACAGGACCAG------TTCTTTGGAGGATTC
CTGAACATCAGTGTTGGGTATGGCCTGGCACTGATGATAGGTATCTGCATCAGTGGAGGA
GTGTCAGGGGGGCATCTCAACCCTGCTGTCACCCTCGCCATGGCTGTCCTGGGCAAGCTC
---AAGCCTGTA--
---------------------------------ATCAAG---ATGGTGGAGGTCATCATC
TCCCCC------ACCAACACCAGCACCTACACCACAGCTACACAAGGCATCTTTGCATCT
TATCCTTTCTTCGAAACCAAACAGGTGACCTTACTGACCCTTGCTATGGACCAGATGCTG
GGGACCGCCATGCTGTTGATCATCATCCTCTCAGTAACTGACGAGAAGAACATGAAGATC
CAGAGCAGCATGGTGCCCCTCACCATAGGCCTGGGCCTGACAGCCATCCACCTGAGTTTT
GGTCTGAATGCTGGGTCAGCCATCAACCCTGCCAGGGACTTCTCCCCACGCATGATGAGC
TACTTAGCTGGATGG------------GAGGAACCTTTCAAGGCC------------TTT
TGGATCCCATGGGCG---

>GAXK01155593_Calanus_finmarchicus_Glp6
ATGAAGAGGATGAAGAACATGGAT---AGGTTTCGGACTGCTGGCCACTGCTTCCTGCAG
AAGTTCCTGGCGGAGGCCCTGGGCACCTTCACCCTGGTGCTGCTTGGTGATGGAGCTGTA

GCCCAGGTGGTGCTGGGCAATGCTGCCAGACACGACCAG------TTCTTTGGAGGATTC
CTCAACATCAGTGTTGGGTATGGCCTGGCACTGATGGTTGGTATCTGCATCAGTGGAGGA
GTGTCAGGGGGACATCTCAACCCTGCTGTCACCCTTGCCATGGCTGTACTGGGCAAGCTC
---AAGCCTGTGCAGGTTCCTGTGTACTGGGCTGGCCAGTACCTTGGGGCTTTTCTAGCA
GCAGCAGTACTTTGGGGAGAGTATGCTGATGCTATCAAG---ATGGTGGAG---------
---------------ACATCTGGCAGTTACACCAGCGCTACTCATGGAATATTTGCATCT
TATCCATTCTTTGGGACAGACCAGGTCACCACCTTGAGCCTGGCCATGGACCAGGTGCTG
GGGACAGCCATGCTCCTCATCATCATCCTGGCAGTCACTGACAACAACAACATGAAGATA
GAGAGCAGCCTTGTGCCCATCACAATTGGGCTTGGACTGACCGCTATTCATCTCAGTTTT
GGTCTGAATGCCGGGTCTGCCATCAATCCTGCAAGGGACTTCTCCCCCCGCTTGCTGTCC
CTCTTGGCTGGGTGG------------AAGGAGCCATTCAAGGCTGCTGACTCCTGGTTC
TGGATCCCTTGGCTGCTCCCCCATGTAGGAGGAGTGCTGGGGGCTGGGCTGTACAAGGTC
ATGGTGGGCTGCCATATCAAGGAGCAGGAA-------
>GAXK01155595_Calanus_finmarchicus_Glp5
------------------ATGGAT---AGGTTTCGGACTGCTGGCCACTGCTTCCTGCAG
AAGTTCCTGGCGGAGGCCCTGGGCACCTTCACCCTGGTGCTGCTTGGTGATGGAGCTGTA
GCCCAGGTGGTGCTGGGCAATGCTGCCAGACACGACCAG------TTCTTTGGAGGATTC
CTCAACATCAGTGTTGGGTATGGCCTGGCACTGATGGTTGGTATCTGCATCAGTGGAGGA
GTGTCAGGGGGACATCTCAACCCTGCTGTCACCCTTGCCATGGCTGTACTGGGCAAGCTC
---AAGCCTGTGCAGGTTCCTGTGTACTGGGCTGGCCAGTACCTTGGGGCTTTTCTAGCA
GCAGCAGTACTTTGGGGAGAGTATGCTGATGCTATCAAG---ATGGTGGAG---------
---------------ACATCTGGCAGTTACACCAGCGCTACTCATGGAATATTTGCATCT
TATCCATTCTTTGGGACAGACCAGGTGACCACCTTGAGCCTGGCCTTGGACCAGATGCTG
GGGACAGCCATGCTTCTGATCATCATCCTGGCAGTGACTGACAAGAAGAACATGAAGATA
GAGAGCAGCCTGGTCCCCCTGACTATAGGACTTGGACTGACTGCCATCCACTTGAGTTTT
GGTCTGAATGCCGGGTCAGCCATCAACCCTGCCAGGGACTTCGCCCCTCGCCTGTTGACC
AGCATGGCCGGCTGG------------AAACAGCCATTTAAGGCTGCTCAGTCCTGGTTC
TGGATCCCCTGGTTGCTGCCCCACGTGGGCGGGGTGGTGGGGGCTCTCATCTACCAGTTC
ATGATTGGCATACACCATCCTTTGGAGGAG-------
>GBXT01002924_Calanus_glacialis_Glp4

---AATATCAGTTTTGGGTACGGGCTTGCCCTGATGATTGGTATTTGTATCAGCGGAGGA
GTGTCGGGAGGCCATCTCAACCCTGCAGTCACGCTA---ATGGCGGCTGTCAAGAATCTG
---AAACCACTCCAGGTCCCTGTCTACATGGCTGGTCAGTACCTGGGGGCTTTCCTTGCA
GCTCTTGTGCTGTGGGGAGAGTATGCTGATGCTATCAAG---ATGGTGGAG---------
---------------ACATCTGGGAATATCAGT---------------------------

>GAXK01155600_Calanus_finmarchicus_Glp4
------------------ATGAAG------ATTGGGGAGAATACCAACCAGTATGTAAGA
GAGTTCTTGGCTGAGACTCTGGGTACTTTTGCTCTGGTCATCTTTGGTGATGGGGCTGTG
GCTCAGGTGGTGTTGGGCAATGCTGCCAGAGGGGACACC------TTCTTTGGAGGATTT
CTGAACATCAGCTTTGGGTACGGGCTTGCCCTGATGATTGGTATTTGTATCAGCGGAGGA
GTGTCGGGAGGCCATCTCAACCCTGCAGTCACGCTAGCCATGGCGGCTGTCAAGAATCTG
---AAACCACTGCAGGTCCCTGTCTACATGGCTGGTCAGTACCTGGGGGCTTTCCTTGCA
GCTCTTGTGCTGTGGGGAGAGTATGCTGATGCTATCAAG---ATGGTGGAG---------
---------------ACATCTGGCAGTTACACCAGCGCTACTCATGGAATATTTGCATCT
TATCCATTCTTTGGGACAGACCAGGTGACCACCTTGAGCCTGGCCTTGGACCAGATGCTG
GGGACAGCCATGCTTCTGATCATCATCCTGGCAGTGACTGACAAGAAGAACATGAAGATA
GAGAGCAGCCTGGTCCCCCTGACTATAGGACTTGGACTGACTGCCATCCACTTGAGTTTT
GGTCTGAATGCCGGGTCAGCCATCAACCCTGCCAGGGACTTCGCCCCTCGCCTGTTGACC
AGCATGGCCGGCTGG------------AAACAGCCATTTAAGGCTGCTCAGTCCTGGTTC
TGGATCCCCTGGTTGCTGCCCCACGTGGGCGGGGTGGTGGGGGCTCTCATCTACCAGTTC
ATGATTGGCATACACCATCCTTTGGAGGAG-------
>GIVD01089612_Rhincalanus_gigas_Glp10
------------------ATGAAG------CTTACTGGGGAAAATAACAGATATTTAAGA
GAATTTCTAGCAGAAACATTTGGAACATTTGCTCTGGTTCTGTTCGGCGATGGAGCTGTG
GCGCAGGTTGTTCTAGGCAATGCTGCCAGAGGTGATACT------TTCTTCGGTGGTTTC
CTCAATATAGCAATTGGATATGGATTGGCTCTGATGATAGGTATCTGTATCAGTGGTGGA
GTGTCAGGAGGACATCTTAACCCAGCTGTCACTCTGGCCATGGCTGCAATCAAGAAACTA
---AAACCAATCCAGATCCCGATCTACATGTTGGGACAATATCTTGGAGCCTTTCTAGCA

GCACTGATATTGTGGGGAGAATATGCAGATCTCATCAAA---TTGGCTGAAATAACTAAA
TCCCCA------ACCAACTCCAGCATGTATACCAGTAACACTCATGGTATATTTGCCTCT
TATCCCTTCTTTTCCACCCAGCAGGTAAGTAGTATTAGCCTAGCGATGGATCAAATGTTG
GGAACTGCCTTACTCCTGATTATCATTCTAGCAGTAACGGATCAGGATAATATGAAGATT
AATAATAGTCTGGTCCCACTTCTAATTGGACTGGGTCTTACTGCTATTCATCTCAGTTTT
GGTGTGACCGCTGGATCTGCTATC------------------------------------

>GIVD01135889_Rhincalanus_gigas_Glp9

------------------TTGGGTACATTTGCTCTGGTTCTGTTTGGTGATGGAGCTGTC
GCCCAGGTGGTTCTTGGCAATGCTACAAGAGGTGATCAT------TTCTTTGGTGGTTTT
CTGAATATAGCAATTGGATATGGATTGGCTCTGATGATAGGTATCTGTATCAGTGGTGGA
GTGTCAGGAGGACATCTCAACCCAGCTGTCACCCTGGCCATGGCTGCAACCAAGAAACTA
---AAACCAATTCAGGTCCCAATCTACATGTTGGGACAATATCTTGGAGCCTTTCTAGCA
GCACTGATATTGTGGGGAGAATATGCAGATCTCATCAAA---TTGGCTGAAATAACTAAA
TCCCCA------ACCAACTCCAGCATGTATACCAGTAACACTCATGGTATATTTGCCTCT
TATCCCTTCTTTTCCACCCAGCAGGTAAGTAGTATTAGCCTAGCGATGGATCAAATGTTG
GGAACGGCCTTACTCCTGATTATCATCCTAGCAGTAACGGATCAGGATAACATGAAGATT
AATAATAGTCTG--

>GIVD01128542_Rhincalanus_gigas_Glp8

---------------ATGTTGGGTACCTTCGCCCTGGTCATATTTGGAGATGGAGCTGTA
GCCCAGGTTGTATTAGGGAACAAAGCAAGAGGAGATGAG------TTCTTTGGTGGTTTC
CTCAACATCAGCTTTGGATATGGTCTGGCGTTGATGATAGGTATTTGTATCAGTGGGGGA
GTATCAGGAGGACATCTCAACCCGGCTGTAACACTTACCATGGCTGTACTCAGGAAGATC
---AAACCTATACAAGTTCCTGTCTACATGATAGCTCAATATCTGGGGGCTTTCTTAGCA
GCTTTGGTTCTCTGGGGTGAATATGCAGAGGCCATTTAT---ATGGTCGAT---------
---------------------CCAAATTACGAGAGC---ACACATGGGATATTTGGTTCC
TATCCATTCTTTTCTCAAAAACAGATAGGTCTGGGATCCCTAGCTGTTGATCAAATGCTC
GGCACTGCTCTCCTGATCATCATCATTCTGGCAGTCACTGATGAGAACAATATGAAGATC
AGTGGCAGTCTTGTTCCTCTCATCATTGGACTTGGTCTTACTGCCATCCATCTCAGCTTC
GGA---

>GIVD01060647_Rhincalanus_gigas_Glp7
------------------ATGAAG---AATTTGCGTTTCAACACTCCAGAGCTTGTAAGA
CAATTCCTGGCTGAGATGTTGGGTACCTTCACCCTGGTCATATTTGGGGATGGAGCTGTA
GCACAGGTGGTATTGGGAAATGCTGCAAGAGGAGATCAG------TTTTTTGGTGGATTT
CTCAACATCAGCTTCGGGTATGGCCTGGCATTGATGATAGGTATTTGTATTAGTGGTGGA
GTGTCAGGAGGACATCTCAACCCAGCTGTAACACTCACCATGGCTGTACTCAGGAAGATC
---AAACCTATACAAGTTCCTGTTTACATGATAGCTCAATATCTGGGGGCCTTCTTGGCA
GCTTTGGTTCTCTGGGGTGAATATGCAGAGGCCATTTAT---ATGGTAGAT---------
---------------------CCAAATTATGAAACC---ACACATGGTTTATTTGGAACC
TATCCATTCTTTCCCACAGAGCAGATAGGTTTAGGATCCCTGGCTGTAGATCAAATGCTC
GGCACTGCTTTCCTGATCATCATCATTTTGGCAGTCACTGATGAGAACAATATGAAGATC
AGTGGCAGTCTTGTCCCTCTCATCATCGGACTTGGTCTTACTGCCATCCATCTGAGCTTC
GGACTGAATGCTGGTTCTGCAATAAATCCTGCAAGAGATTTCTCACCCCGTCTTGTGTCA
TTGATGGGTGGTTGG------------GACAACTCATTCCAGAATGCTGGTTCTTGGTGG
TGGATCCCCCTTGCTCTTCCTCATATTGGAGGTTTACTAGGCGGTCTTGTGTACACTGTT
ATGGTGGGGGCTCATCATAGGGAAGAAGAAGAC----
>GIVD01060390_Rhincalanus_gigas_Glp6
------------------ATGAAG---AATCTCCAATTCAACACCCCAGAGCTGGTAAGA
CAATTCCTGGCTGAGCTATTGGGTACCTTCGCCCTGGTCATATTTGGAGATGGAGCTGTA
GCCCAGGTGGTATTGGGAAACAAAGCAAGAGGTGATAAG------TTCTTTGGTGGTTTT
CTCAACATCAGCTTTGGATATGGCCTGGCATTGATGATAGGTATTTGTATCAGTGGTGGA
GTGTCAGGAGGACATCTCAACCCAGCTGTAACACTCACCATGGCTGTACTCAGGAAAATC
---AAACCTATACAAGTTCCTGTCTACATGATAGCTCAATATCTGGGAGCATTCCTGGCC
GCTTTGGTTCTCTGGGGTGAATATGCAGAGGCCATTTAT---ATGGTAGAT---------
---------------------CCAAATTACGAGAGC---ACACATGGTATATTCGGTTCC
TATCCATTCTTTTCTATGGAACAGGTAGGTTTGGGATCCCTGGCAGTTGACCAAATGCTC
GGCACTGCTCTCCTCATCATCATCATTCTGGCAGTCACTGATGAGAACAATATGAAGATC

AGTGGCAGTCTTGTCCCTCTCATCATCGGACTTGGTCTTACTGCCATCCATCTCAGTTTT
GGACTGAACGCTGGTTCTGCAATCAATCCTGCAAGAGATTTTTCACCCCGAGTGTTATCA
TTCATAGCTGGTTGG------------GAGAACTCATTCCAGAATGTTGGAATTTGGTGG
TGGATCCCTCTTGTCCTTCCTCATATCGGAGGTTTACTAGGCGGTCTTGTCTACACTGTC
ATGGTGGGAGCTCATCATAGGGATGAAGAGAC----
>GFCI01485726_Pleuromamma_xiphias_Glp5
------------------ATG------------------GAGCTGGGACAATACTCCAGG
CAGTTTTTGGCAGAGACTTTGGGCACATTTTTGCTTGTGGTGCTTGGGGATGGGTCTGTG
GCTCAGGTGACTCTTGGAAAGAAACTTAATCTGGAT---------GGATTTGGGGGTTTA
CTAAATATAAGCTTTGGATATGGCCTGGCTGTCATGGTTGGCATATTGGCATCAGGTGGT
GTGTCAGGAGGCCATCTCAACCCAGCTGTCACGGTGGCCATGGCTTCACTAAAGAAACTT
---AAATTAATCCAGGTTCCAATCTACATGGCCGGACAGTACCTGGGTGCCTTCTTAGGA
GCAGCAGTTGTGTTTGGAATATATGCAGATGGAATTAAT---AAAGTGGA----------
---------------------------AAGTCAAAAGAGACAATGGGAATATTTTCCTCC
TATCCGGGA------TTTTCACAGGCAAGTGAAACCACATTGGCTTTTGACCAAATTCTT
GGTACCAGCCTCCTCATCCTCATCATCCTCGCCGTCACCGATAACAACAACATGGCCATT
GTCAACGGAATGTTTCCGCTCTACATCGGTCTGGGTCTCACAACCATCATTCTCAGTTTT
GGTCTGAACGCGGGAGCAGCTATCAATCCTGCCAGGGATTTTTCTCCCAGAATCTTCACC
CTCATGGCAGGATGGGGG---------ACAAAAACTTTTGAGGTTAATGGTTACTTCTTC
TGGATCCCCTGGTTGCTTCCCCACATTGGAGGACTGCTTGGAGCTTTCATCTACCACTTT
ACCATCGCGTACCACCACCCAAAGTCTACCACAGAC-
>GFCI01342623_Pleuromamma_xiphias_Glp4
------------------ATGAAG------------TTCAAAACTCCTAAACTCGTCAGG
GAATTTCTGGCGGAGGTCCTCGGGACTTTCCTTCTCGTCGTGTTCGGAGATGGCGCCATC
ATGCAGGTTGTCCTTGGAAACGCTGGGACGGGGGGCGGCAGAAGCTTCTTCGGAGGATTC
CTCAACATTTGCCTGGGCTACGGCCTCGGCCTCATGATCGGGATCCTGGCCAGCGGAGGA
GTGTCTGGGGGCCACCTCAACCCGGCAGTTACCCTCTCCATGGCCGCCATCAAGAAGTTG
---AAGCCGATACAGATCCCGATCTACATGGCTGGCCAATACCTGGGAGCATTTCTAGGG
GCAGCGGTTCTTTATGGAATCTACGCCGATGCGATACAT---ATGGTTGAT---------
---------------------CCAAACAAG---TTAACCAGCCATACTATGTTTGCTTCC
TACCCCAACTTCACATTTGAAGCGCCCAGCGTGGCGACACTGGCGATGGACCAAATGTTC
GCCACGGCCCTCCTCGTCCTCATCATCCTCGCCGTCACGGACGAGAACAACATGAAGGTC
ATCGGGTCCATGGTGCCCCTACTCATCGGCCTCGGTCTCTGTGCCATTCATATCAGTTTT
GGTTTGAATGCCGGTTGTGCCATCAATCCCGCGCGGGATCTTTCTCCTCGGCTGTTCACA
CTTATGGCGGGATACGGC---------GACAAAACCTTCAAGCAAGATGACTATTTCTTC
TGGATTCCGTGGCTTCTGCCTCACCTCGGAGGCGTCATCGGAGCCCTGGTCTACCAGTTC
ATGGTGGCGCTACACCACAGAGATGAA----------
>HAHV01124381_Tisbe_holothuriae_Glp5

---AATGTGGCATTGGGATATGGTCTGGCTCTCATGGTTGGGATCCTGGTTAGTGGTGGG
GTCAGTGGTGGACATCTGAACCCAGCTGTGACACTGACCATGGCTTTCATGGGCAAGTGC
---CGGTGGGTTCAGGTGCCGGTTTACTGGTGTGCACAATATCTTGGTGCCTTCATTGGT
GCTAGCGTACTGTATGGTATCTATGCTGATGGG---------------------------

GGAACAGCTGTCCTGCTCATCATTATCCTTGCCGTGACTGATGGCAAGAACATGAAGGTT
CCATCTGGCCTCATCCCACTGCTCATTGGACTTGGTCTCACTGCAATTCATCTCAGTTTT
GCATTCAATGCTGGATGTGCCATCAACCCTGCCAGAGATTTCTCACCCCGTCTGCTAACC
CTGGCAGCTGGCTTTGGTGCTGAAAGCGACCAAACCTTCACTGCC---------------

>GCIT01012568_Tisbe_furcata_Glp5

--ATCTTTGCCAGC
TACCCAGCC------AATGGAGAGATCACCACAGTGACCCTGGTGTTTGACCAAATCCTG
GGAACTGCAATTCTCCTTGTGATCATCCTTGCTGTGACTGATGGCAAGAATATGAAGGTT
CCCTCTGGTCTCATTCCGCTTCTCATTGGACTTGGTCTCACTGCCATTCATCTCAGCTTT
GCCTTTAATGCTGGATGTGCTATTAACCCTGCCAGAGATTTCTCTCCGCGTCTTCTTACC
CTGGCAGCAGGATTTGGTTCTGAGAGTGATCAAACCTTCACTGCCTACGATTACTTCTTC
TGGATCCCCTGGATGATGCCTCACGTTGGCGGCCTGCTGGGAGCATTCACCTACTACACC

ATGGTGGAGATGCATCATGATGACGAGGATGACGAG-
>HAHV01124387_Tisbe_holothuriae_Glp4
------------------ATGGGC------------AGCAAGGTGAAGGACTACGCACGC
AAGTTCTCCGCTGAGTTCCTCGGCACCTTCATGCTGGTGCTGATCGGCGATGGATCCATT
GCTCAGCTGGTCATGAAGCGGAGTGGGGCTTCCCCG---------GAGGGATCTGACTTC
CTCAATGTGGCACTGGGATACGGCCTCGGACTCATGGTTGGGATCCTGGTTAGTGGTGGG
GTCAGCGGTGGCCACCTCAATCCAGCCGTGACACTGTCCATGTGCTGCCTGAAGAAGTGC
---AAGTGGATGTGTCTGCCCATCTACTGGGCAGCACAATATCTTGGTGCCCTCCTTGCT
GCTGCGATTCTCTACGGTATCTATGCTGATGGCATCAACAGCAGATTGGAT---------
------------------------GGACCTAATCTTGCCAGTGCTGGGATCTTTGCCTCC
TACCCAGCT---------GATAAGATCAGCACAGTGACCCTTGTATTTGACCAGTGCCTG
GGAACAGCTGTCCTGCTCATCATCATCCTGGCCGTGACTGACGGCAAGAACATGAAGGTT
GCGTCTGGTATGGTGCCTCTACTCATAGGACTTGGACTGGCTGTCATTCACATCAGCTTT
GCATTTAATGCTGGATGTGCTATTAATCCGGCTAGAGATTTCTCACCGCGCTTGTTGACC
CTAATGGCTGGCTTTGGC---------GGCGAGACCTTCACCGCTTCGGATTCCTTCTTT
TGGATCCCGTGGATCATGCCCCACATTGGCGGCGTCCTCGGTGCGCTCTCCTACTTCTTC
CTCATTGAGTGGCATCACCCAGACACAGAGGATATG-
>GCIT01023304_Tisbe_furcata_Glp4
------------------ATGGGC------------AGCAAGGTCAAGGACTACGCGCCC
AAGTTCTCCGCCGAGTTCCTCGGCACTTTCATGCTGGTGCTGATCGGCGATGGTTCCATT
GCACAGCTGGTCATGAAGCGGAGTGGGGCCACCCCT---------GAGGGGTCAGACTTC
CTCAATGTGGCACTTGGATATGGCCTTGGACTCATGGTTGGAATCCTGGTTAGTGGTGGG
GTCAGCGGTGGGCACCTTAACCCGGCCGTGACACTCTCAATGTGCTGTCTGAAGAAGTGC
---AAGTGGATCTGCCTGCCAATCTACTGGGCAGCACAATATCTTGGTGCCCTCCTTGCC
GCCGCAATCCTTTACGGTATCTATGCTGATGGCATCAACTCTAGATTAGAT---------
------------------------GGTCCTAACCTTGCCAGTGCTGGGATCTTTGCTTCC
TACCCAGCT---------GATAAGATCAGCACAGTGACCCTTGTGTTTGATCAGTGCCTT
GGAACAGCTGTTCTGCTAATCATCATCCTTGCTGTGACTGATGGCAAGAACATGAAGGTA
GCATCTGGCCTGGTGCCTCTGCTCATTGGACTTGGACTGGCGGTCATTCACATCAGTTTT
GCATTTAATGCTGGATGTGCTATTAATCCGGCTAGAGATTTCTCACCCCGTTTGTTGACC
CTGATGGCCGGCTTTGGT---------GGCGATACCTTCACGGCCGCCGACTCCTTCTTC
TGGATCCCCTGGATCATGCCTCATATTGGCGGCGTCCTTGGTGCTCTCTCCTACTTCTTC
CTCATTGAGTGGCATCACCCAGACACAGAGGACATG-
>CM017381_Tigriopus_californicus_Glp3
------------------ATGAGG---------------TGCGCCCCGCCGCTTCTTCGG
AAGTTTCTGGCCGAATTCTTGGGAACCTTTCTCCTGGTGATTTTTGGTGACGGAGCCATT
GCCCAACACGTCTTGGGT---------------------------CAGAATCAATCATTC
CTATCGATTTGCTTAGGATATGGTTTGGGCCTCATGATCGGCATCCTTGTCAGTGGAAAC
GTGTCAGGAGGTCATCTCAATCCAGCCGTGACTTTGGCCATGAGCTTGCTCCAAAAGTGC
---ACTTACATCTGTGTGCCCGTGTATTGGGCGGCCCAATACTTGGGCGCCTTCGCTGGC
GCCACCGTTCTTTATGGGGTGTATGCAGATGCTATTGAA---GCCCAA------------
---------------------TTGGGCATGAATATGTCTAGTGCTGGGATTTTTGCCTCG
TATCCC---------AAGGATGACATCGGTGTGGTGACACTGATTGTGGGCCAAACTTTG
GGAACAGGGATCCTTTTGATCATCATTTTAGCAGCGACAGACAAGAAGAATATGAATGTG
CCACCCGGTCTCTTGCCATTGACTATTGGATTGGGCCTCACCGCTATTCACATAAGCTTT
GCCTATAACGCAGGGTGTGCAATCAATCCCGCTCGAGACTTCTCTCCGCGATTACTCACC
TATGTGGCTGGTTTCGGG------------AACGTTTTCTCGGCGGACAATTATTTCTTT
TGGATCCCTTGGATTATGCCCCATATTGGAGCCGTTATTGGAGCCTTGACTTACTACTAC
TTTGTGGAAATTCATCACAAAGATGAAGACGACGAG-
>JABCAI010000033_Tigriopus_kingsejongensis_Glp3
------------------ATGAAA---------------CGAGTGCCACCTCTGGTGCAA
AAGTTCCTGGCCGAGCTCCTCGGCACATTCCTCCTCGTGATCTTCGGGGATGGATCCATC
GCCCAGTTTGTGTTGGGA---------------------------CAAAACCAGACGTTC
CTCTCCATTTGCTTCGGCTATGGATTGGGTCTGATGATTGGCATCCTGGTGAGTGGCAAC
GTGTCGGGAGGACATCTGAATCCAGCTGTCACCTTGGCCATGGCCTTGCTCCAAAAGTGC
---CCTTACATCTGCGTGCCAGTGTACTGGGCAGCCCAGTATCTGGGTGCATTTCTGGGA
GCGAGTGCCCTCTATGGCGTGTACGCCGATGCCATTGAG---GCCAAG------------
---------------------GTGGGCATCAATATGACCAGTGCAGGCATCTTTGCCTCC
TACCCC---------AAAGACGATATCGGCATGAGCACCCTGGTTTTGGGCCAAACCCTG
GGCACTGCCATCCTCCTAATCATCATTTTGGCGGCCACCGACAAGAAGAACATGAACGTG
TCGTCTGGATTGGTCCCAGTCGTGATTGGCTTCGGTCTCACCGCTATCCATATTAGCTTT
GCCTACAACGCAGGCTGCGCCATCAATCCAGCCCGAGACTTCTCTCCGCGCTTGCTGACT
TACATTGTCGGTTTTGGC------------AACGTGTTCACCGCCGACGATTACTTCTTC
TGGATCCCTTGGCTCATGCCTCACGCTGGGGCCATCATTGGAGCCCTCACCTACTATTAC
CTGATTGAGCACCATCACCGAGATGAGGATGACTTT-
>GBTC01007239_Tigriopus_californicus_Glp6
------------------ATGCCC---------CGAAGTAAACTTCCCGTTCTGGCTAAG
CAGTTCTTGGCCGAGGCCTTGGGAACCTTTCTCCTTGTTCTTTTCGGAGACGGGGCCATC

GCTCAATATAAAGCCCAATTGGCCTCAGTA---------------ACACCCACAGTATTC
CCCTCCGTAGCTTGGGGCTATGGATTGGCGCTCATGATCGGTATTCTGGCTAGTGGGGGA
GTGTCCGGAGGGCACTTAAACCCAGCAGTCACCTTGGCAATGGCGTGCCTCAAGAAGTGT
---TCCTGGAAGCAACTCGTCGTCTACTGGATAGCCCAATATTTGGGTGCTCTGTTGGGG
GCGGCTCTATTATACGGCCTTTATTTGGACGCAATCAAT---CTACTA------------
---------------------CCAATTAGCGATGCTAGCGCAGCTGGGATATTCGCTACT
TTTCCCGGC------TTGAATAATCTGAACATTGGTACTTTGATTGCCGATCAACTCGTG
GGAACTGCTCTTCTTCTGATTATCATCCTAGCGGTCACAGATTCGAGAAATATGAATGTG
CCATCTGGCTTGGTGCCAATTCTCATTGGTTTGGGTCTGACAGCAATTCATCTCAGTTTC
GCATTCAACGCCGGTTGTGCCATCAATCCTGCTCGCGATCTCGCACCACGTCTAATTAGT
CTCATT------TACAGC---------TCCTCTGCTTTTGGTTCCTACGACTACTTTTTC
TGGATCCCATTGATCATTCCTCATATCGGGGCTGTGTTTGGAGCCGTTATCTATTATTTT
GGTGTCGAGATGCATCATGAGCCTCATCCACTAGCT-
>GHUE01053132_Tigriopus_californicus_Glp5
------------------ATGCCC---------CGAAGTAAACTTCCCGTTCTGGCTAAG
CAGTTCTTGGCCGAGGCCTTGGGAACCTTTCTCCTTGTTCTTTTCGGAGACGGGGCCATC
GCTCAATATAAAGCCCAATTGGCCTCAGTA---------------ACACCCACAGTATTC
CCCTCCGTAGCTTGGGGCTATGGATTGGCGCTCATGATCGGTATTCTGGCTAGTGGGGGA
GTGTCCGGAGGGCACTTAAACCCAGCAGTCACCTTGGCAATGGCGTGCCTCAAGAAGTGT
---TCCTGGAAGCAACTCGTCGTCTACTGGCTAGCCCAATATTTGGGTGCTCTGTTGGGT
GCGGCTCTATTATACGGCCTTTATTTGGACGCAATCAAT---CTACTA------------
---------------------CCAATTAGCGATGCTAGCGCAGCTGGAATATTCGCTACT
TTTCCCGGC------TTGAATGATCTTAACATTGGTACTTTGATTGCCGATCAACTCGTG
GGAACTGCTCTTCTTCTGATTATCATCCTAGCGGTCACGGATTCGAAAAATATGAATGTG
CCATCTGGCTTGGTGCCAATTCTCATTGGTTTGGGTCTGACAGCAATTCATCTCAGTTTC
GCATTCAACGCCGGTTGTGCCATCAATCCTGCTCGCGATCTCGCGCCACGTCTTATTAGT
CTCATT------TACAGC---------TCCTCTGCTTTTGCTTCCTACGACTACTTTTTC
TGGATCCCATTGATCATTCCTCATATCGGGGCTGTGTTTGGAGCCGTTATCTATTATTTT
GGTGTCGAGATGCATCATGAGCCTAATACAATAGGC-
>GHXK01158759_Platychelipus_littoralis_Glp3_v1
------------------ATGAAG---------------AAGATACCGAATCTGGCCAGA
CAGTTCCTGGCCGAGTTCCTTGGCACCTTCATGCTGGTCATCTTTGGAGACGGGGCAATA
GCTCAGTACAAGTTTCTCGAAGGT---------------------CAACAGAGAAACTTT
CTCTCAGTGGCACTGGGGTATGGATTGGCCTTGATGGTTGGCATCCTGGTAAGTGGAAAT
GTGTCCGGTGGCCACTTGAATCCCGCCGTCACCCTGTCCATGGCCTGCATCAAGAAGTGC
---AAGTGGATCTGTGTACCTGTCTACTGGATGGCCCAATACCTAGGCGCACTCTGTGGT
GCTGCTGTCCTCTTCGGAGTCTACGCAGACGGCATCGCG---CTA---------------
---------------------AATGGGGGTAAAGCTGCCAATGCGGGCATCTTTGCGTCA
TACCCCACC------AATGAGAATTTGAGTCCAGTGACCTTGGTTGTGGACCAGGCCTTG
GGCACAGGTCTTCTTCTCATCATCATTTTGGCAGTGACTGACAAAAAGAACATGAACGTC
TCCTCTGGATTGATCCCGTTGTTGATTGGTCTCGGTCTGGCCGCCATTCACATAAGTTTT
GCCTACAACGCTGGCTGCGCCATCAACCCGGCCAGGGATTTTTCTCCACGACTTTTGACT
TTGATTGCTGGTTTTGGA---------AACAATGTTTCGTCAAAAACAATTCGTTCTTT
TGGATTCCTTGGATCATCCCACACATTGGTGGGGTCTTGGGAGCCTTGACGTACTTCTTC
ATGATTGAAATGCACCACAGGGACGATTTGGAC----
>GHXK01158764_Platychelipus_littoralis_Glp3_v2
------------------ATGAAG---------------AAGATACCGAATCTGGCCAGA
CAGTTCCTGGCCGAGTTCCTTGGCACCTTCATGCTGGTCATCTTTGGAGACGGGGCAATA
GCTCAGTACAAGTTTCTCGAAGGT---------------------CAACAGAGAAACTTT
CTCTCAGTGGCACTGGGGTATGGATTGGCCTTGATGGTTGGCATCCTGGTAAGTGGAAAT
GTGTCCGGTGGCCACTTGAATCCCGCCGTCACCCTGTCCATGGCCTGCATCAAGAAGTGC
---AAGTGGATCTGTGTACCTGTCTACTGGATGGCCCAATACCTAGGCGCACTCTGTGGT
GCTGCTGTCCTCTTCGGAGTCTACGCAGACGGCATCGCG---CTA---------------
---------------------AATGGGGGTAAAGCTGCCAATGCGGGCATCTTTGCGTCA
TACCCCACC------AATGAGAATTTGAGTCCAGTGACCTTGGTTGTGGACCAGGCCTTG
GGCACAGGTCTTCTTCTCATCATCATTTTGGCAGTGACTGACAAAAAGAACATGAACGTC
TCCTCTGGATTGATCCCGTTGTTGATTGGTCTCGGTCTGGCCGCCATTCACATAAGTTTT
GCCTACAACGCTGGCTGCGCCATCAACCCGGCCAGGGATTTTTCTCCACGACTTTTGACT
TTGATTGCTGGTTTTGGA---------AACAATGTTTCGTCAAAAACAATTCGTTCTTT
TGGATTCCTTGGATCATCCCACACATTGGTGGGGTCTTGGGAGCCTTGACGTACTTCTTC
ATGATTGAAATGCACCACAGGGACGATTTGGAC----
>CM017381_Tigriopus_californicus_Glp4
------------------ATGGCC---------CGAAGTAAACTTCCCGTTCTGGCTAAG
CAGTTCTTGGCAGAGGCCTTGGGAACCTTTCTGCTTGTTCTTTTCGGAGACGGGGCCATC
GCTCAATATAAAGCCCAATTGGCCTCAGTA---------------ACACCCACAGTATTC
CCCTCAGTAGCTTGGGGCTATGGATTGGCGCTCATGATCGGTATTCTGGTTAGTGGGGGA
GTGTCCGGAGGGCACTTGAACCCAGCCGTCACCTTGGCAATGGCGTGCCTAAAGAAGTGT
---TCTTGGAAGCAACTCGTTGTCTACTGGCTAGCTCAATATTTGGGTGCTCTGTTGGGA

GCGGCACTATTATTCGGGCTTTACTTTGATGCAATCAAT---GAACTA------------
---------------------CCAATTAGTGATGCGAGCGCGGCTGGCATTTTCGCTTCT
TTTCCCGGC------ATGAGTGATCTTAACATTGGTACTCTGATTGCTGATCAGCTCGTG
GGAACTGCTCTTCTCCTGATTATCATCCTAGCGGTCACAGACTCGAGAAATATGAATGTG
CCATCTGGCTTGGTGACAATTCTCATTGGTTTGGGTCTGACAGCTATTCATCTCAGTTTT
GCATTCAACTCCGGTTGCGCCATTAATCCTGCTCGTGATCTCTCGCCGCGTGTTATTAGT
CTTCTT------TACATG---------TCCTCCGCTTTTGCTGCCTACGACTACTTTTTC
TGGATCCCATTGATCATTCCTCATGTCGGGGCTGTGTTTGGAGCCGTTATTTATTATTTT
GGTGTCGAGATGCATCATGAGCCTGATACACTAACG-
>GCHA01005546_Tigriopus_japonicus_Glp4
------------------ATGCCT---------CGAAGTAAACTTCCCGTTCTGGCCAAG
CAGTTTCTGGCCGAGACCTTGGGAACCTTTCTCCTCGTTCTCTTCGGAGATGGAGCCATT
GCCCAATACAAAACCCAAATGGGCTCAGGC---------------ACACCCACAATTTTT
CCCGCGGTGGCTTGGGGCTATGGATTGGCACTTATGATCGGCATTTTGGCCAGTGGTGGA
GTGTCGGGAGGGCATTTGAACCCGGCTGTCACCTTGGCAATGGCTTGTCTAAAGAAGTGT
---TCATGGAAGCAACTCCCCGTTTATTGGGTGGCCCAATATTTGGGTGCTTTCTTGGGG
GCCGCTCTCCTGTACGGGATCTATTATGACGGTTTTAATCACACAATT------------
---------------------AATTTGACCGATGCCGATGTGGCTGGGGTGTTCGCTACG
TACCCTGGA------AATGGCAAGTTCAACATCGGAACTTTGATAGCAGATCAGCTCTTA
GGCACTGCACTTCTTCTCATCATCATATTGGCGGTCACCGATTCGAGAAATATGAACGTA
CCATCGGGGATGGTACCGATTCTAATTGGCTTTGGTCTGACAGCAATCCACCTTAGCTTT
GCCTTTAATGCAGGTTGTGCGGTCAATCCGGCTCGTGATTTAATGCCACGCATCGTGACT
CTGATTTTTGGCTTT------------CCTGAGTCTTTTACGTCATACGATTACTTTTTC
TGGATTCCATGGATTATTCCACACTTTGGAGCTGTGTTTGGGGCTGTAATCTATTTCTTT
GGTATCGAGATGCACCATGAACCTACCTTGCTAAGC-
>JABCAI010000033_Tigriopus_kingsejongensis_Glp4
------------------ATGAGG---------AGGGTGAAGATCCCCTTGCTGGTCAAG
CAGTTCTTGGCCGAGGCTCTCGGGACATTTCTTCTGGTGGTTTTTGGAGATGGCGCCATC
GCCCAATTCAATTCTCAGGCCAAGAGCGCC---------------ACCGACACCAGTTTC
ATGGCCGTGGCCATCGGCTACGGGTTCGCTCTCATGATCGGCATCCTGGTGAGTGGAGGC
GTGTCCGGAGGTCATCTCAACCCGGCTGTCACCCTCGCCATGGCGTGTCTGCGGAAATGC
---TCCTGGAAACAATTGCCCGTGTATTGGCTGGCCCAATACTTGGGTGCCTTGCTCGGA
GCCGCCGTGGTCTATGGGATCTATTGGGATGCCTTCAAT---TTCCTG------------
---------------------GAAATGTCATACGCCGAAACACGCGGAATATTCGCCTCC
TATCCGGCC------AGTGACCAATTGAGCGTGACCACGTTGATCATGGATCAGCTCCTG
GGCACGGGCCTCCTCCTCATCATCATCCTAGCCGTCACCGACTCCAGGAATATGAAGGTG
TCCTCGGGCTTGGTTCCACTCTTGATTGGCCTGGGATTGGCTGCCATTCACATTAGTCTG
GCTTTCAACGCCGGCTGCGCCATCAATCCGGCTCGCGATTTCTCCCCGCGATTACTCACT
CTGATAGCTGGTTATGGA---------ACTGAAGTGTTTTCAGCTTTCGACTACTTCTTT
TGGATCCCCCTGATTATGCCGCACATCGGTGGGGTGATTGGAGCGGGACTCTATTACCTC
TGCGTTGAGATGCACCACGATACCTCGGAAGAATTT-
>GHXK01120398_Platychelipus_littoralis_Glp4
------------------ATGCCC---------------AAGGCTAATTTGCTTGTAAGA
CAAGGTCTAGCCGAATTCTTCGGTACCTTTTTCCTCGTTCTCATTGGCGACGGAGCAATA
GCGCAGGCAAATTTGAGAGGTGAT---------------------GCCGAGAGGACTTTT
AGCACGATTGCCTTTGGCTACGGATTTGGTTTGATGATTGGCATCTTGGTAAGTGGAGGA
GTGTCTGGTGGCCACTTGAATCCCGCCGTCACCCTGGCCATGGTCTGCCTGAAAAAGTGC
---AAGCCGATTTGTCTTCCCGTTTACATGGCTGCCCAATACCTTGGAGCATTTGCCGGT
GCTTCAGTTCTGTTTGGAATCTATGCCAACGGCCTGAAC---GATATTGAT---------
---------------------CCCTTCTTAACTAGCAAAACGATGGGCATCTTTGGCACC
TTTCCCGCT------GGTGATCAGATCAGAGTTGAGACCCTGATAATGGACCAGATACTT
GGAACGGCAGTTCTATTAGTCATCATTTTGGCAGTGACGGATGAAAATAACATGAAGGTA
GCCTCCGGTTTGGTGCCTCTCCTGATTGGTCTCGGACTTACCGCCATTCACTTGTCGCTG
GCCTTCAATGCTGGTTGTGGCATAAACCCAGCGAGAGATCTTTCTCCGCGAATTTTGAGT
TTGGCAGTGGGGTATGGA---------ACTGAACCTTTCACAGCCTACGACTACTTCTTC
TGGATTCCTTGGCTAATGCCCCACGTTGGAGCTGTCATTGGAGCCTTCGCTTATGTTCTT
TTGATTTCAATGCATCACGGAGAGACTGCAGTG----

File S4: Alignment for Fig. S1C

>GESM01001861_Polyxenus_lagurus_GlpB1
GTTCAAGAAAACCCGGACGACGTGCCCTTCAGGTCCCAGATGATCAAAGAATTCGGAGCT
GAAGTCCTGGGGACTTTCTTCCTAACGGTGATGGTGGTCGGCCAAGCCCTGGTAGATAAG
GTAGCAGCTGAAGACCGCGGAGGCCCCATCTGCGCCATGATCTCCAACGGGCTGACAGTC
GCCAGCGCCGTCTACTTGGTCGGCGGAATCTCAGGCGCCCACATTAATCCAGTAGTAACG
GTTGGATTCGCCGTCATCGGCCGGTTGCCCTGGAAAAAGGTCCCGCACTATCTGGCCGCT
CAATACCTTGGCGGATTCTTAGGCGGCGCAGCTGCCTATGGACTGTACAGCGATGCCTTG
TTCGCTTTCGAGGGCGACGCCAAATCGATCAACACAGCCGGAGGATTCATGTCCATGCCC
CACGAATTCCTGGATTACCAAACGGCCTTCTTCGACCCGTTTTTACCAACCGTATACCTG
ATGGCAGGTATCATGGCTCTAAACGATTTCGAAAATTTCGCTCCTCCTAAGGGTCTTATG
CCCTTGATGGTCGGACTACTAGTCGCTGCGTTAGTCGGAACCCACGGTTACAAC---GGT
TTAGCCATCAACCCCGCAAGAGAACTGGGACCCAGAGTATTCATATCGTTAGCAGGATGG
GGCTCGGCCCCTTTCAGTCCCATGAATTACAACTGGTGGTGGCTTCATATCGTGGCGGCT
CACTCCGGATCCATTGTTGGTGTTTACCTTTACCTTATGCTTATCGAATTTCATCACCGT
CCGAAAGAAAACATC
>GCIU01051770_Eudigraphis_takakuwai_GlpB1
GTCAAACAGAATCCGGAAACGGAGCCGTTCGGCAGACAGGTGGTCAAGGAATTCGGCGCC
GAAGTGCTGGGGACGTTTTTTTTAACGTCTATGGTCGTTGGAGAAGCTTTAACTGACAAA
TTGGCCAATGACGAATATGGAGGTCCGGTGAAGGCCATGATTTCAAACGGATTGGTTGTC
GCCGCTGCTGTCTATTTAGTCGGCGGCATTTCCGGGGCCCATATTAATCCTGTGGTGACG
TTAGCATTTGCTTTAATCGGTCGACTGCCGTGGAAAAAAGTGCCTCATTATTTAGTCGCT
CAGTACATCGGCGGATTTTTAGGTGGAGCTGCAGCTTTTGGACTTTACAGTGATGGGATC
TACGCTTTGGAGGGGGATGCTCGATCAGCGAACACGGCTGGTGGTTTCATGTCTATGCCC
CATGAGTTTCTCGATTACGATGTGGCTTTCTTAGATACGTTTTTACCATCTGCGTATTTG
ATGATCGGGATCATGGCGTTAAATGACTTTGAAAATTTTGCTCCGCCCAAGGGTGTGATG
CCCCTGATGGTGGGGATGTTAGTCGCTGCCTTGGTGGGAACCCACGGAATGAAC---GGA
TTGGCCATCAACCCAGCCAGGGAATTGGGACCACGAACCTTCATCTCACTGGCTGGATGG
GGCTCCATCCCTTTCAGTCCTTTAAATTACAACTGGTGGTGGCTGCACATCGTGGCTGCT
CACACTGGCGCCATCCTCGGTACCTACATCTACCTGCTGATGGTCGAGTTCCATCACTCC
TTGAAGAAGGTCCCA
>GESM01001010_Polyxenus_lagurus_GlpB2

---AATTTCCTGGGATACAGCGACGCTTTCCTAGATGTGTTCATTCCATCCATCTATCTC
ATGATTGGCGTCATGGCGCTCACCGATTTCAACAACTTCGCTCCTCCGAAGGGTCTCTTT
CCACTTCTTATAGGGCTGCTTGTCGCCTCGCTGGTTGGATCCCACGGGTACAAC---GGT
TTGGCCATCAATCCTGCCAGGGAACTTGGTCCCAGACTGCTGGCATCCCTCACGGGCTGG
GGCGGCATTCCTTTCAGCCCTTTGAATTTCACCTGGTGGTGGCTCCAAATCATCGCCGTT
CATTTAGGCACCATCCTGGGCGTTTACGTCTACCTCATCTTTATTGAATTTCAGCACCCC
CTCAAACGAAATTCC
>GERY01005807_Glomeridella_minima_GlpB1
AAGCCATGTTTTGGCATGCAGAACAAAATGGTGAGGGATTTACTTAGGGAATCAGCAGCA
GAATGCCTTGGAACCTTCATACTAAATCTGTTCGCAATCGGAGCAAATGCCCAAAGGGTC
CTTAGCAGTGGAACCCATGGAAGTTATGGATCATGTGTAGCAGCAAATACACTAGGAGTA
ATGTCAGCAGTTGCAATTGTTGGTGGAGTCAGTGGTGCCCAAATTAACCCTGCTGTGAGT
TTTGGGATGGCCATGTGCGGAAGGTTACCATTTTATAAACTTCCCTTCTACATGATTGCC
CAATATGTTGGAGCATACTTAGGTGCAATCATCGTCTATCTCAACTACCAAGGGGCTTTG
TGGTATTTTGCTGGAGATGTCAAAGGAATTGAGACTGCTGCCATTTTCACAACTTTTGCC
CCACCTTGGATATCGACTGCAAATGCTTTCTGCGATTCGATGGTCAACTCGGCGCTGCTG
CTCGTGGGTTGCATGGTCCTAGTGGATCACCGTAACACTGCCCCTATAAAGGGTGCTTTC
CCGATCTTCATGGGCCTCATCGTGTGCGCTCTTCAGCTGACGTATAACTACAACACGGGC
AGTCCCAACAATCCCGCAAGGGATCTTGGACCCAGGGTTTTCTGCTTATCTTTTGGCTGG
GGATCCCAAGTCTTCAGTTGGGAAGATTACAGTTGGTGGTGGATCCCATTTGTCGCGCCA
CACGTTGGAGCAGTCATCGGAGTCTGGCTCTATCTTCTAGTCATTGAATTGCATCATCCT
CCTATCAATGATGAT
>JAAFCE010004287_Trigoniulus_corallinus_GlpB1
------AATCTCAACAGAATGGTGAATTTTCGGTGCAAACTTTTTAAAGAAATTTTGGCA
GAATTCTTCGGGACATTTATCCTAGTGAGCTTCACTGTGGGTGCTGCTGCTCAGAGGAGT
TTCCATGATGATCTACCTTTCAACTATATTTCATCCACGTTTTGCGGTGGAGTTGCAGTC
ATGATGGCTGTTTACGTAGCCGGAAATGTTTCTGGAGCTCATGTAAATCCAGCAGTAACC
TTCGCAATGGCTAGCATTGGAAAGTTTAAATGGTATAAAGTACCATACTATATGCTTTCT
CAGTACTTTGGAGCACTGGTTGGAGGAAGTTTTGTGTATGTAGCATATAACGATGCAATA

GAATATCACCATGGAAATAATAGAACATTAGAATTTGCATCAGTCTGGACCTCATTTCCA
TCAAAATATTTAACTATTGAAAACGCATTTTTTGATCAGATGGCAGCTGCCTGCTTATTG
ATGATTTTAAATTTATCAGTGACGGATATGGAGAACATGGGAGTAGAATCTGGCCAGCAG
CCACTCATTTTGGGGTTAGTTGTTACAACAATTGGATTTGGATTTAGCACAAATGCTGGA
AATCCAATGAATCCTGCCAAGGACTTTGGAACTAGAGCTTTCCTTGCTCTTGTTGAACAC
AGCGATTACGTTTTCACGGGTGCAACTTTTTGTTGGTGGTGGATTCCCTTGGTAGCACCT
CACCTTGGAACATTGTGTGGAGGACTACTTTATGAAGTTTTGATTCAGCCCAATAAGCAC
CAAGAAATTACTACT
>GESI01004340_Polydesmus_complanatus_GlpB1
GCTGGGATATTCGGAATCAGAATTAATATT---CCCAACATTTGTAAAGAGATGCTGTCG
GAATTTATAGGAACGTTTATTCTAGTGCTAATGCTCATCGGAATCGGTGCTCAAACGGTA
CTGAGTGGCGGAACTGCGGGAAATACTCTTAGCGGTACTTTGGGAACTGGAATGGCAGTG
ATGATGGGGGTCACTGTGAGTGGAAAACGATCAGGGGCCCACATCAACCCCGTGATGAGT
TTGGGTTTAGCTTGGGCGGATAAATTTGCGTGGAAAAAGGTTCCGGCTTATATGTTATCT
CAATATCTCGGAGCTTTTGTCGCCGCCGGAATCGGATATCTGGTCTACAAAGATGCGTTT
CTGGCTTACGATGGCGACCAATATAGCCTGACAACGGCGGCCATTTACACTTCATTCCCC
GCTCCATTCATTAAATTCTGGAACGCCTTCATTGATGCCATGGTGTCATCGGCGCTGCTG
GTGTTGCTCAATTTGGCCGTTGCAGACCGTAAAAATATGAATGTTCAAACAGGTTTCTCG
CCTATCCTCTACGGTTTCCTGGTAACGTGTATCGCGTCTGGCTATTCATACAACGACGGC
AATCCAATGAACCCGGCTCGCGACTTCAGCCCGAGGGTATTCCTCCTGGCAGCCGGGTGG
GGAAACGAAGTCATGTACGGAAACGATTTTGCTTGGTGGTGGGTGCCCTTAGTGGCTCCG
CATATTGGGGCTATAATCGGAGGAACTGTTTACGTGTTGTTTATCGAAGCGCATCATCCA
ACGGACGAAGAAATA
>GERQ01007761_Callipus_foetidissimus_GlpB1

--TCAACA
CTAAGCAATGGAGGGTACGGAAGCGCTCTGAGCAATGCATTTGGCACAGGCATAGGCGTT
ATGATGGCCGTTCATACAAGTGGAGGGATGTCAGGAGCACACGTTAATCCTGCAGTGTCT
GTCGCAATGGCGTGCATTGGAAAGTTCAGCTGGCTTCGAATGCCATTTTATTTGCTAGCA
CAATATGGTGGAGCATTCGTAGGATCTGCACTAGCGTATTCCGTCTACCAAGGGGCACTC
CACAATTATAATGGAAATGCGCACAATACGACTACAGCTGCAATCTGGACATCTTTCCCT
CAACCGTTTTTGTCTTTTGAAAACGCAATGTTCGATTCTATTGTATCATCAGCTCTTTTA
ATGATGCTTAATTTGGCTGTAAGCGATACCTATAACATGAGCGAATCTCCAAAATATGCC
CCTTTGTATCTAGGGCTTGTTGTCACCACCATTTCACTTGGTTACTCATACAATGCTGCC
AATCCGATGAATCCCGCACGTGACTTGGCACCCCGGTTGTTCTTGGACATGGTCGGATGG
GGTAAAGAGCTTTATCAAGGTTCAAACTACACGTGGTGGTGGATTCCTATTGTAGCTCCT
CACATAGGTTGCATTCTAGGAGGATTTATTTATGTCCTACTCATCGAAATGCATCATCCA
ATCGAGGGAGAGGAT
>GESN01006960_Polyzonium_germanicum_GlpB1
------------------------AATATATGGAAAGAAATTTATCGGGAAATGTTGGCC
GAATTCATCTCGATATTCATTCTAACGGCTATGCAAACAGGCTTAATAGCGACTAACATT
ATCAGCAACAGCAAACCTGGAACCAGCTTTAACGCAGCACTTGGCTGCGGTTTGGTCGTC
ACGATCGCCGTTTACGTGGGTGGAGGCGTTTCCGGAGCACACATCAATCCGGCGGTGACG
TCAACCTTCGCCATGATGGGAAAAATGCCTTGGATCAAAGTGCCCTTTTATTTTCTCGCT
CAATACATCGGTGCCTTCATGGGAGCAGCAGCCACTCACGCACTCTACAATGACGCCATC
CTAAGTTACAATGGCGAA---TTCAGT---GATATGGCTGGCATCTGGACAACATTTCCG
GCCAAATTCTTGACGGCAGGAAACGCCATGCTCGATTCAACTTTCTCTTCAGCCTTGCTC
ATGCTACTGGTATTGGCCATTTGCGATCAAAAAAACATGAACTTGCCGGCAGGAATGTGT
CCATTCTTCATCGGCCTTTTGGTGGCGACCATCGGGCTCGGTTACTCGTCAAACTGTGGC
AATCCAATGAATCCGGCCAGAGATCTTGGACCGAGGCTATTTGCTGTCATTTCGGGTCAC
GGCACAGGCGTTCTCGTAGGTAACGACTACGTCTGGTGGTGGATTCCCGTTGTGTTTCCT
CACGTCGGTTGCCTCATTGGAGCGTTCATCTATGAGGTGCTCATCGAGTTTCACCATCCG
GACGACAACGAAGTC
>GERZ01044073_Hanseniella_nivea_GlpB2
GTGAACAAAGTAGCGGCCAATGTCCGGCTTAGAAATCAGTATCTTCGAGAATTTTGCGCC
GAAACATTCGGCACTTTCGTCTTTATAGCGTTCAACATAGCCTGCGGTATCCAAGATGTT
CTCAGCGGGAGCAAGATGGGCGACATCAACACTCGTGCTATTGGGGGAGGATTGGGAGTT
ACTTTCGGAATATACGCAACAGGCGGAATTTCAGTTGGACTGCTGAATCCAGGTTTGATA
ACCACCTGGGCCAGTCTGGGACTCATCTCCTGGAAGAAAGTTCCTGTCTTAATCGGCGCA
GAATACTTGGGATCCTTCTTAGCTTCCGCGTTCGTGTACGTCGTCTACTACGGCGGAATC
CAAGAAGCAGCGGTTAACGAGTTCACGTTAGAAACCGCGTCAGCTTGGACCAGTTTCCCC
AGAGAGAACTTGACTGGACTCAACGCTTGGATTGACGAGATCGCCGCTCCAGCCTTCCTA
GCACTCTGTGTTCTCTCCGTCTCCAACTGGCAAGCCAAGTACAAGGACAACAACATCACT
CCTCTCGTCCTAGGGCTCATCGTGACGGCTCTTACTTATACCTTCAGCTACAACTGCGGG
GATCCAATGAACGGAGCTCGGGATCTCAGTCCCAGGATCTTCTGTTACCTGGCTGGATGG
GGTCCCAAGGTGTTCAGTCCCTGGAACTTCCAGTGGTGGTTCCTCGTGCCCATTGCGAAT
CATATCGGCGCGCTGCTCGGTGGTTGGGTGTACTACTTGATGGTCGGTTGGCAGCAGGAT
ATAGTCGAGGCATCG

>GAKX01036488_Symphylella_vulgaris_GlpB1

---ATGTTTAGGATGTTTCATCTTGACTGCAGTTGGAACAGCAGTCAGTGCTCAGGTAAT
TTTAAGAAAGGCCAGAATGGAAATATAACGACAATATACGTAGGATGTGCATTAGCCGTC
GGATTAGCACTATACACTTCTCTCGGTATTTCAGGTGGACATGTAAATCCTTCAGTTTCA
ATAGCATTTGCCATAATCAACAAATTTCCATGGAGAAAAGTTCCTGCATACATTTTCGCA
CAGTTTTTAGGATGTTTCATGGGATCTGGAATTGTGTATGTTTTGTACAGAGAACCAATA
TTTCAATTTGAAGCTAACAGCAGTAAACTAGAAACAATGGGTATCTTTTCCACAATTCCA
AGGGAAAACCTGACTATTCTAGAAGGATTGGTTGACCAGATTATTGGGACTGGATTTCTT
CTGTTAGTCGTGTTGGCTATCTCGGACAAGAAAAACTGCAACGTGCCACTTGCGGTACAG
CCTCCTCTTGTCAGTATACTGGTATTTTGTTTATATATAGGATTCGGATTCAATTGTGGG
AATCCGATTAATCCAGCTAGGGATCTGTCTCCAAGATTATTCATGTCGGTGGCCGGTTGG
GGACAACTTCCATTCAGTTTAATGAATTACAACTATTGGTACATACCTGTCGTTGGACCA
ATCTTAGGAGCGATCTTTGGAAGTTGGATATATCTGTTATTTGTAGAATTTCACCACCCT
TACGATGATGATTTC
>GCIH01031045_Symphylella_sp_GlpB1
ATCCAGATGCTGGCCAGGCGATTACGGGTTAAAAATGAGTTAATCAGGCAGGTTTTTGCC
GAATTTTTAGGATGTTTTGTATTAACGGCATTCGGAAGTGCAATTAATGCCCAGGTTGTC
TTAGGCAAAGGCAAGAATGGTAATTTTACGACTCAACAGATGGGAAACTCGCTAGCGGTC
TGCCTTGCCCTCTACACGTCGTTAGGAATATCAGGTGGTCATATTAATCCATCCGTGTCT
ATAGCCATGGCGGTTATAAGCAAGTTTCAATGGTACAAGGTCCCGGCCTACATCGCAGCG
CAGTATTTGGGCTGTTTTATGGGCGCAGGAGCTACATGGATCCTCTACAGAGAACCACTC
CAGGCGTTTGAC------------GTTGCCGATACAGTCGGTGTTTTTGCGACGATGCCC
CGAGAGAATTTATCCATCCAAGAGGGAATGGTTGATCAGATTTTCGGAACTGCCCTTCTG
CTCCTAATGGTCTTAGCCCTCACCGATAAGAAAAATTGCGAAGTTCCAATATCGATTCTG
CCTCCCCTAATCAGCCTCCTAATCTTCTGCTTAAGTACAGCCGTTGGATTTAGCTGCGGT
AATCCGATTAATCCGGCCAGGGATCTATCACCCCGAATTTTTATGGCTGTAGCCGGTTGG
GGAGATTTACCGTTCAGTTTAATGAATTACAACTATTGGTACATCCCGGTAGCAGGGCCT
ATCATAGGAGCCATCCTTGGTAGTTGGATCTATCTACTGCTTATTGAATTCCACCATCCA
---GAAGAAACATTT
>GERZ01044746_Hanseniella_nivea_GlpB1
ATTGAATCCTTCGCAGCAAGAATTCGAATCAAGCATGAACTACTGCGCCAGTGTCTGGCC
GAGTGTCTAGGGACGTTTATACTAACGGCATTCAGTGTCGGAGTTGGAGCTCAAAGTGTT
CTTAGTGGTGGAAACTCAGGAAATATCATCTCAGCTTACATCGGTTCTGGTGTCGCAGTC
GGCTTGGCTGTCTTCGCTTGTCTTGGAGTTTCAGGTGGACACATCAATCCGGCAGCTTCG
GTTGCCTTCGCGTCTATAGGTAACTTCCCTTGGTACAAAGTTCCTGCCTACATCCTAGCT
CAGTTCACCGGCAGCTTCATCGCAGCGGCGGCTCTTCATGGAGTGTACAAGGAGGCGCTG
GATGCTTACGAAGCGGTCGCTGGGAAGAATCTGACCACCGGAGTGTTCGTCAGTGTTCCC
AATGTCTTTCTGTCTCTGGAAGGAGGAATGTTTGACCAGATCTTCGGAACTGGAATGCTG
CTGCTTTGCGTGCTCGCCATCACCGACAAGAAGAACACCAAGTTCCCCATAGAAGCGGCC
CCAATGGCCCTCGCTATGATGGTAACTGGAATCTGCTGCGCCTTCGGACTCAACTGCGGA
AACCCGATCAACCCGGCGCGCGACCTGCCGCCCAGATTGTACATGGCGATTGCTGGCTGG
GGCGATCTTCCTTTCAGGTACCTGGACTTCCAGTACTGGTACGTGCCTGTGGTTGGACCC
ATGGTTGGAGGGATTCTAGGGGCGTGGGTCTACCAACTCTTCGTAGGATTCCACCATCCG
GTAGACGAACCAGAC
>GESA01000764_Haploglomeris_multistriata_GlpA1
TTC---TCATTTGCTCAAAAATTGAGAATCCGCAATGCTTACGTCAGAGAGGCCCTTGCA
GAGTGCTTGGGCACTTTCATATTGGTGACAATCGGATGCGCATCTGTGGCCCAGTCGGTG
TTCAGTAAAGAAAAAGCAGGCACTTTTTTGAGTGTCAATTTCGGATGGGGTTTGGCAGTC
ATGTTTGGAGTTTACGTCAGTGGAGGCATTTCAGGAGGACATATTAACCCAGCTGTGACT
TTAGCCATGGCCACAATCGGCAAATTTCCTTTCAGAAAAGTACCTATCTACATGATAGCT
CAGTATATAGGAGCTTTCTTGGGTTCTGTTGTCGTTTTCTACGTTTACAGAAATGCTCTG
ACTGAGTTTGAAGGAGAGGCTTTTTCACTGACTTCTGCAGGAATCTGGGCAACATATCCC
CAGAGTTTCTTATCAACTGGAAACGGATTTGGAGATCAGGTGTTGGGAACAGCTTTGCTG
GTAGCATGTGCTCTGGCCATCACTGATGCGCGTAACATGGGCACACCGAAAGGTGTTGTT
CCTCTTATGATTGGACTGGTGGTGGCAGTGATCGGCATGACGTATGGATACAACTGTGGA
TATGCCATCAACCCAGCTCGTGATCTGTCTCCAAGAATTTTCACAGCAATTGCTGGATGG
GGCAGCCAGGTTTTCAGCTTCCATGACTACAACTGGTGGTGGGTACCTGTTGTGGGACCT
CACGTGGGTGCCATTGTGGGTGCTTGGGTTTATTTCTGCGTAGTCGAAGCTCATTGGCCA
GATGAGGAAGACGAA
>GERY01013134_Glomeridella_minima_GlpA1
---------------CAAAGATTGAGAATCCGCAATGCATATGTACGAGAGGCCCTTGCG
GAGTGCTTGGGCACTTTCATCTTAGTGACAATCGGATGCGCATCTGTGGCCCAGTCTGTA
TTCAGTAAAGAAAAAGCAGGCACTTTTTTAAGTGTCAATTTTGGATGGGGTTTGGCAGTC
ATGTTTGGAGTTTACGTCAGTGGAGGCATTTCAGGAGGACATATCAACCCAGCTGTTACT
TTAGCCATGGCAACTATCGGCAAATTTCCTTTCAGAAAGGTTCCTCTCTACATGATAGCT
CAGTATATAGGAGCTTTCTTGGGTTCTGTTGTCGTTTTCTACGTTTACAGAAATGCACTG
ACTGAGTTTGAAGGAGAGGCTTTTTCAATCACTTCTGCAGGAATCTGGGCAACATATCCC

CAGAGTTTCTTATCCACTGGAAACGGATTTGGAGATCAGGTGTTGGGAACAGCTTTGCTG
GTAGCATGTGCTCTAGCCATCACTGATGCACGCAACATGGGCACACCGAAGGGTGTTGTT
CCTCTTATGATTGGACTTGTAGTGGCAGTGATCGGCATGACATATGGATTCAACTGTGGA
TATGCCATCAACCCTGCAAGAGATTTATCACCTAGAATTTTCACAGCAATTGCTGGATGG
GGCAGCCAGGTTTTCAGCTTCCATGACTACAACTGGTGGTGGGTTCCTGTTGTGGGACCT
CATGTGGGTGCCATTGTGGGTGCTTGGGTTTATTTCGTAGTAGTCGAAGCCCATTGGCCA
GATGAGGATGACGAA
>GERY01013425_Glomeridella_minima_GlpA2
TTCACTTCGCTTGCTCGCAAAATGCGAATCCGAAATGCATATGTGAGAGAAGCTTTAGCT
GAATGCCTAGGGACATTCATCTTAGTGACAATCGGATGTGCATCAGTGGCCCAGTCTGTG
TTCAGCAAAGAAAAAGCAGGCACTTTCTTGAGTATCAACTTTGGATGGGGTTTGGCAGTG
ATGTTTGGTGTTTACGTCAGTGGCGGCATCTCTGGTGGACACATAAATCCGGCTGTGACT
CTGGCAATGGCTACAATCGGCAAATTTCCGTTCAGAAAAGTTCCTGTCTACATGATTGCC
CAGTATTTGGGAGCTTTCCTTGGTTCTGTCGTCGTTTTCTACGTTTACAGAAATGCTCTG
ACTGAGTTTGAAGGTGATGCTTTCTCAATATCAACCGCTGGCATTTGGGCAACATATCCC
CAATCTTTCTTGTCCACTGGAAATGGATTCGGAGATCAGGTGTTGGGAACTGCTTTACTG
GTGGCATGTGCTTTGGCCATCACCGACGCGCGTAACATGGGCACCCCAAAAGGTGTGGTT
CCTCTGATGATTGGGCTGGTGGTCGCGGTCATCGGTATGACCTACGGTTACAACTGCGGT
TATGCCATCAACCCTGCAAGAGATTTGTCTCCTAGAATCTTTACGGCAATTGCTGGATGG
GGTAGTCAAGTTTTCAGCTTTGACAACTACAACTGGTGGTGGGTACCTGTTGTTGGACCC
CATGTGGGTGCCATTGTCGGTGCGTGGGTGTACTTTGTCGTAGTCGAAGCCCATTGGCCG
GAAGAGGATGAGGAA
>GESM01004697_Polyxenus_lagurus_GlpA1
ATGGACAAATTAAACAAAGCTCTTCATATCAATAACGTATATCTACGGGAAGCCTTCGCG
GAGTTCCTAGGAACCTTCGTTCTAGTGGCAATTGGTGACGCCGGCATTGCGCAGAATGTA
TTTTCCAAAGGCCAGTCTATCGACCCCCTCAGCATCCACTTTGGTTGGGGACTCGGTATT
ATGCTCGGAATATTCATCGCTGGAGGCGTTTCAGGAGGCCATATTAACCCCGCAGTCACA
CTGGCAATGGCAACAGTCGGAAAATTTCCATTTAAGAAAGTGCCCATCTACATCATTTCG
CAATATCTGGGAGGCTTTTTTGCATCGCTTTGCATATTTCTGGTTTATAGGAACGCTATA
ACTCACTTCGAAGGCAGCTCTTACACCTTGGCAACGGCAGGAATATTTGCCTGCTATCCA
CAAGGATGGTTATCTGCTGGAAACGGTTTGGCTGATCAAATTTTTGCTACCGCTATTCTG
GTAGCCGCAGCTTCTGCTATTACCGACAGTCGAAATATGAACGCTCCTAAAGGTGTAGTA
CCATTGATGATTGGACTGGTGGTCGCAGCTATTGGCATGTGCTATGGATACAATTGCGGA
TATCCCTTGAACCCAGCCCGAGATTTGGCCCCTAGGATATTCACCGCGATTGCCGGATGG
GGACAACAAGTGTTTTCAGTCAAGGACTGGAATTGGTGGTGGATTCCCGTTGTAGGACCT
CACATCGGAGCTGTTCTGGGAGCATGGACGTACAGATGTTTCGTCGAAGCCCATTGGCCG
GAAGATGAAGATGAT
>GCIU01049231_Eudigraphis_takakuwai_GlpA1
ACCGATAAGTTGAACAACGCGATTCGCATTGATAATGTGTACCTCCGTGAAGCGTTTGCT
GAATTCCTGGGGACCATGGTGCTGGTGGCGATCGGAGATGCGTCAATCGCGCAATATAAG
TTTGCGGGCGGACAAACAATCGACCCATTGAGCATCCATTTCGGCTGGGGACTGGCAGTT
ATGGTCGGCGTTTTCGTTAGTGGCGGAGTTTCTGGCGGTCATATTAACCCAGCTGTAACA
CTAGCCATGGCTTGCGTCGGGAAATTCCCGTTCAGGAAAGTACCCATCTACATCATCTCG
CAGTATCTGGGCGGATTTTTCGGTGCTCTGCTGATTTATCTGGTCTACAGAAATGCGATC
GTTCACTTTGACGAGGAT---CTAACTTTGGATACTGCTGGAATATTCGCCACTTATCCC
CAGCCTTGGTTGTCTGCTGGTAACGGATTAGGCGATACGATCTTTGGAACAGCCATTCTG
GTAGCAGCAGCATCAGCCATTACAGACAGCAAAAATATGAATGCGCCTAAAGGCTTGGTT
CCTCTGATGATCGGATTCGTGGTTTCGGCAATCGGCATGTGCTATGGCTACAACTGCGGA
TACCCTCTGAACCCCGCTCGGGATTTGTCCCCTAGAATATTCACGTCGATCGCTGGATGG
GGCAAAGAGGTTTTTGCTGTTAACAACTGGAATTACTGGTGGATCCCAGTCGTTGGACCC
CACATCGGTGGTGTGCTGGGTGCCTGGACGTACAAATGTTTCGTTGAATGCCACTGGCCC
GAAGATGAGGAAGAC
>GERS01002433_Craspedosoma_sp_GlpA1
TTCCACTCGTTGACCAGAAAATTGGCGATTCGCAACTCGTACGTGAGGGAAACATTCGCA
GAGTTTCTAGGAACATTCATCCTAGTGGCTCTCGGTGATGCGTCTGTTGCTCAGATGACA
CTTAGTAATCAAGCTTACGGTCAATTCCTCAGCGTGAATCTCGCATGGGGATTTGCTGTC
ATGCTGGGCGTCTTCGTCAGTGGTGGAGTTTCCGGTGGCCACATCAATCCTGCAGTCACC
GTCGCCATGGCAACAATCGGCAAGTTTCCATGGAGAAAAGTTCCAATTTTCATGATAGCC
CAATACGTCGGTGCCTTTGTTGCTTCCGTAGCGTTGTTCTACGTATATCAAAATGCTTTG
ACTGACTTCGAGGGTAATAATAGGACGATAACTACAGCAGGAATCTGGGCCACTTATCCT
CAGGATTTCTTATCCATAGGCAACGGATTCGCTGATCAGGTCATGGGTACAGCTCTTCTG
GTAGCATGCGTATTGGCCATTACTGACGTCAAAAACATGAACACACCGAAAGGCATGGTT
CCACTGATGGTCGGATTGGTTGTGGCCGCCATTGGAATGTGTTACGGCTATAACTGTGGC
TACGCTATCAATCCGGCCCGAGATTTGTCTCCCAGAGTATTCACGTTGATTGCTGGATGG
GGAAGTCAAGTGTTCAGCTACCGAGATTACAACTGGTGGTGGATTCCGGTTGTTGCTCCC
CACGTCGGAGCTGTAGTTGGTGCGTGGGTCTATCGTCTGTTCGTTGAAATTCATTGGCCA
GAAGATGAGGATGAC
>GESN01021765_Polyzonium_germanicum_GlpA1

```
TTGAACGCCATCGCACGCAGATTGCGAATTCGCAATACGTACGTCAGAGAAGCGTTCGCT
GAATTCCTGGGCACCTTCGTCCTTGTGTCTTTCGGCGATGCTTCTGTGGCCCAAAGTGTC
CTAAGCAATCAAGCTGGCGGAACATTCCTGAGCATCAACCTAGGATGGGGATTCGCCGTC
ATGATGGCGATCTTCGTGAGCGGCGGCATTTCCGGTGGCCACGTTAATCCAGCTGTATCG
GTAGCGTTGGCGACGTGCGGCAAATTTCCGTGGCGAAAAGTGCCGATCTACATGGTGGCT
CAATACCTGGGAGCGTTCGTCGCCTCAGCCGCTGTGTTTAGCGTCTATCGCAATGCGCTG
ACTGATTTTTCTGACGGCGAATTCAAGTTGGACACTGCCGGCATTTGGGCCACGTATCCC
AAGGACTTCCTGTCGGTCGGCAACGGATTTGCAGATCAGGTCATGGGTACGGCGCTGTTG
GTTTCCTGCGTGATGGCGATCACGGACTCTAAAAACATGAAGACGCCGAAGGGCGTAGTT
CCATTGATGATCGGCATGCTCGTAGCAGCCATCGGCTTGTGCTTCGGCTACAACTGCGGT
TATGCCATCAATCCGGCCCGCGATTTGGCGCCGCGTTTCTTCACCTTCATCGCCGGATGG
GGACCGCAAGTGTTCAGTTTCCGTGACTACAATTGGTGGTGGGTACCGGTGGTTGCTCCG
CACGTTGGTGGTGTTCTTGGAGCGTGGATTTATCGCATTTTCATTGAGGCACATTGGCCA
GAAGACGAGGACGAC
>JAAFCE010006668_Trigoniulus_corallinus_GlpA1
TTAAGCGCTTTGACGAAAAAGCTTCAAATTCGTAACATATACATACGTGAAATTTTGGCA
GAAATGCTCGGAACTTTCATCCTTGTGGCCCTTGGCGACGCATCAGTGGCTCAAAGTGTC
CTGAGCAAGAACGCACAAGGAAACTTCATCACTATTAATTTCGGATGGGGTTTCGCGGTC
ATGTTCGGAGTGTTCGTTTGTGGTGGTGTTTCTGGTGGTCATATCAACCCTGCCGTGTCG
TTGGCTTTAGCAACAGTAGGAAAATTTCCGTGGAGAAAAGTGCCCTTGTTTATGATAGCT
CAGTACGTGGGCGCTTTCATCGCTTCCGTCTTTATTTTCCTCGTCTATAGAGATGCACTG
ACGGATTTTTCGGGCGGTAACTATACCATAACGACGGCCGGTATCTGGGCAACGTATCCT
CAGGATTTTTTGTCAGTTGGAAATGGTCTTGCAGATCAGGTGATGGGCACTGCTATTTTA
GTGGCCTGCGTGTTGGCCATTACAGACAACAAGAACATGAATACTGCAAAAGGATTCGTA
CCACTGATGGTGGGCTTTTGTGTGGCAGCCATCGGAATGTCCTATGGCTACAATTGCGGT
TATGCCATTAATCCAGCACGGGATTTATCTCCCCGTATTTTCACCGCTATTGCCGGTTGG
GGTATGCAAGTGTTCAGTTACGCAAATTATAGTTGGTGGTGGGTTCCAGTTGTTGGACCT
CATATTGGAGCTGTATTGGGCGCCTGGGTTTATTGGTTGCTAGTTGAGATGCATTGGCCT
GAAGACGAAGAAGAG
>JAAFCF010003739_Helicorthomorpha_holstii_GlpA2
TTAAACATGCTTACTCGACGCTTCCGAATACGTAGTTCTTACGTCAGAGAAACTTTGGCT
GAATTTTTAGGCACTTTAGTGTTGGTGGCCCTTGGAGATGCAGCCAATGCCCAAAGCACT
TTAAGTAAAACAGAAGCTGGGACCTTTCTAAGTGTCGATTTAGCATGGGGATTTGCAGTG
GCAATGGGCGTGTTTGTCAGCGGTGGAGTTAGTGGTGGACATATTAATCCAGCAGTAACC
GTAGCCATGGCATTACTAGGAAAATTTCAATGGCGGAAAGTACCACTTTTTATGCTGGCG
CAATACGTCGGCGCATTTGTTGGATCAGCAATTGTCTACTGCGTCTACTATGGTGCTCTT
GGAAATTTTGATGATGGAATGTACACAATGACAACAGCTGGCATTTTTGCTACGTATCCT
AAGGATTTCTTATCCACCGGAAATGCAGTTGGAGATCAGGTGACTGGTACTGCTATTCTA
GTAGTTATTGTTTTAGCCATAACCGACAGTCGAAACATGGGAACCGCTAAAGGAATGGTT
CCATTAATGATTGGCGTTGGAGTTGCTTGCATCGGCATGTCCTACGGATATAACTGCGGA
TTTCCACTAAATCCAGCCAGAGATTTAGGTCCAAGATTGTTTACCGCAATTGCTGGATGG
GGAAACATGGTTTTCAGTGGTGGTGATTTTTGCTATTGGTGGATTCCAATTGTTGCTCCA
CATGTAGGAGCCATCTTGGGTGCTATTGTGTACTGGGGCATCATTGAATTGCACTGGCCG
GACGAGGAAGACGAA
>JAAFCF010006680_Helicorthomorpha_holstii_GlpA1
TTAAAAGCATTAACCAGAGGATTTCGTGTTCGTAATGTCTATTTTAGAGAAGCATTGGCT
GAGTTTTTGGGAACTTTTGTGCTGGTGACATTAGGATGTGCAGCCAACGCCCAAAGTGCC
CTTAGTAAAGGAGAAGCTGGGACCTTTTTAAGTGTTGGTTTAGCATGGGGTTTAGCAATT
ATGCTCGGTGTATTTGTCTGCGGAGGAGTCAGTGGTGGCCATATTAACCCAGCTGTATCC
GTTGCAATGGCTTTACTTGGAAAATTCCCATGGAGAAAAGTGCCTTTATACATGATTGGA
CAGTATCTGGGAGCTTTTGTTGGTTCCGCTGTTGTTTTCTTTGTATATCAAGGT------
---------------GGAAATTACACCATTGAAACAGCAGGCATATTTGGAACTTTCCCT
AAAGAATTTCTCTCCGTTGGAAATGGAATTTCTGATCAGGTGTTAGGTGCGGCTATATTA
GTTGCTTGTGTATGCGCTATAACAGACAGCAGAAACATGGGAACACCCAAAGGTGTGATT
CCTCTTATGGTTGGACTGGTAGTAGCAGTCATTGGAATGTCTTATGGATACAACTGTGGT
TTTGCCATTAATCCAGCCAGAGATTTGGGTCCAAGATTGTTTACAGCTGTTGCTGGTTGG
GGCATTCAAGTCTTCAGCTTTAACGACTATAGTTGGTGGTGGATTCCAGTTGTTGGTCCA
CATGTAGGTGCCATTTTAGGAGCCTTGATTTACTGGGTTTTAGTTGAACTTCATTGGCCA
GAAGATGATGATGAT
>GESI01021132_Polydesmus_complanatus_GlpA1
TTAAGTATAGTGGGACGTCGTCTGCGAGTGCGCAATAACTACATTAGGGAAGCCCTAGCC
GAGTTTTTAGGAACCTTCGTACTCGTAGCATTAGGCGATGCTTCCAACGCCCAAAGTGTC
CTAAGCAACACGACGGCTGGGACCTTCCTCACCGTGGACTTAGCATGGGGCTTCGCCGTC
ATGTTGGGGGTGTTCGTGAGCGGAGGAGTGAGTGGTGGTCACATCAACCCCGCCGTGTCG
GTGGCCATGGCGCTAGTGGGCAAGTTCCCCTGGCGGAAAGTCCCGCTCTACATGTTGGCC
CAGTACGTGGGAGCCTTCACCGGGGCGGCACTCGTCTTTTTCGTCTACCAAAATGCCTTG
GACAGTTTTGACGACGGCAACTACACGATGACCACAGCCGGGATCTTCGCCACATATCCC
AAAGAATTTTTGTCCGTAGGAAACGGAATCGGAGATCAGATCACGGGTACAGCGATCCTG
```

GTGGCCTGCGTGTGCGCCATCACCGACTCCAAAAACATGGGCACGTTGAAGGGCCTGGTA
CCCTTGATGATCGGTTTTCTGGTGGCGGCTATCGGCATGTCTTACGGCTACAACTGCGGT
TTCGCTATCAACCCGGCTCGTGACTTGGGTCCCCGTTTCTTCACCTACATCGCCGGCTGG
GGCTCGCAAGTCTTCGCATTCAACGACTACTCTTGGTGGTGGATTCCTATCCTAGGGCCC
CATATCGGCGCTTGCGTTGGGGCCATCATCTATCTGCTCAGCGTGGAATTGCATTGGCCC
GACGACGACGAAGAC
>GCHC01029313_Hanseniella_sp_GlpA3

------------------------------------AAGGTTCCGTTCTACCTAATCGCT
CAGTACCTTGGTGCCTTCTTCGGTTCCTGTCTCGTCTACGTTCTGTTCCTAGATAAGCTG
GAAGCGCTACCCGTGCCT---AAGGACCTGTCAACAGCTGGAATATTCGCTACGTATCCG
GGGACTAACGTTACATTAGGAACTGTGTTTATTGATCAGGTTGCAGAAACGGGGTTGATG
GTGTTTCTTGTTATGGCGATATCTGATCCCAGGAACATGGAGGTCCCAAAACATTTTGTT
CCCTTGTGCGTAGGATTTATCATCACCGCCTTGGGCACCGCCTTCAGCCTTAACTGTGGT
GGGCCAATCAACCCGGCCCGTGATTTTGGTCCAAGATTTTTTACCTACATAGCTGGTTGG
GGCAGTGATGTCTTTAGTACCCTAGACGGTAACTACTGGTGGATTCCTATTCTGTGTCCG
CACATTGGAGCAATAATCGGAGCCTGGACCTACATGCTACTGATCGAAGCCCATTGGTCA
AGTTTACCCGAGACC
>GCHC01047146_Hanseniella_sp_GlpA2
TTGGAAAACCTAAAGAAGAAACTGCGGTTTGAAAATGACACACTTCGGGAATCTGTGGCC
GAATTTCTTGGAACCTTTATTCTAATACTAATCGGAAATTCTAGTGTGGCACAAGTCGTA
CTCTCTCCAGTTCCTTCTAGGAGTAATACGACAATCTATTTCGGTTGGGGGTTTGCTGTC
GCCTTGGGAGTTTACACAGCTGGCGGTGTATCTGGTGGTCATCTGAATCCAGCTGTAACA
GTTGCCTTGGCTACGTTAGGACGCACCAAGTGGTTCAAAGTTCTATTCTACATAATCGCC
CAGTATCTTGGTGCCTTCATCGCTTCATGTATCATCTACGTCGTGTTTTTAGATAAGCTA
GAAGCCCTTCCCGAACCT---AAGAATTTATCAACCGCCGGTATATTCGCTACATATCCG
GGCACCAATGTTACGTTATGGACAGCATTTATTGACCAGGTTGTGGTAACGGCGCTGCTG
ATGTTCCTTGTGATGGTCATATCTGATCAAAGGAACATGGAAGTAAGCAAACAAGTGGCT
CCACTCTGTGTAGGTGTTATCGTCACCGCCATTGGTATGTCCTTTGCTCTGAACTGTGGC
TATGCCATCAACCCGGCACGTGATTTTGCTCCAAGGTTCTTCACTTATATTGCTGGTTGG
GGTAACGACGTTTGGAGCGCTCTAGATGGTCATTACTGGTGGGTTCCAATCCTTGGTCCG
CACGTTGGAGCGATAATCGGAGCCTGGACCTACTATCTCCTCATCGAGGTCCATTGGCCC
AGCCGATCCGGATTC
>GCHC01032687_Hanseniella_sp_GlpA1
ATGGAGTCACTTGCCAAGAAGTTGAGGATAAAAAACGATATCGTCCGAGAAGCTCTTGCT
GAATTCTTGGGAACTTTTCTCCTTGTTCTAATTGGAGATGCCAGTGTAGCCCAAGCCAAA
CTTAACCGTGGAGAGGCTGGAAATTACATTACCATATATTTCGGTTGGGGTTTGGCTGTC
GCTATTGCTGTTTATTCAAGTGGCGGAATTTCTGGAGGTCACTTAAATCCGGCGGTGTCT
CTCGCTATGGCGACAGCTGGACGTTTGTCGTGGATCAAATTTCCGGTGTACATGGTAGCC
CAGTATCTAGGAGGATTTGTGGCCTCTTGTGTCCTATATCTTGTTTATATTGATAAAATC
GCCCATGTGGACCCAGAA---AAGACATTGGCCACTGCAGGAATTTGGGCCACTTATCCA
AGTGCTGGAGTTTCTGTAGGGAACGCCTTTGTTGACCAGATTGTTGCCACTGCCTTGCTT
CTTCTCACGGTAATGGCCATCTCAGACTCCAAGAACATGGAGGTTCCCAAACCGCTTGTT
CCTATTTGTGTTGGTGTGGTCATTACCGCCATCGGCATGTGCTTCGGTCTCAACTGCGGC
TACGCTATCAACCCAGCCCGTGATCTGGCACCCAGGTTCTTCACATACATCGCCGGTTGG
GGCGGACAAGTTTGGAGCGCTTTGGACTCTAACTACTGGTGGGTGCCAATCCTGGGTCCT
CACGTTGGAGCGATAATCGGAGTCTGGCTCTATCTGCTTCTGGTTGAATTCCACTGGCCG
CCCACAAATGGAAAC
>GERZ01022232_Hanseniella_nivea_GlpA1
ATGGAATCGTTTGCCAAGAAGTTAGGGATAAAAAACGATGTTGTTCGAGAAGCCCTCGCT
GAATTCCTGGGGACCTTTCTTTTGATGGTATTCGGTGATGCCAGTGTGGCTCAGGCTAAT
TTGCCCCCTGGTGCTGGAGGAAACTTCATTTCCATCTACTTCAGTTGGGGTCTCGCGGTG
GCTATTGGAGTCTATGCTTGCGGAGGAGTTTCAGGAGGTCATGTCAACCCCGCGGTGTCC
CTCGCGATGGCGACTATCGGTCGTCTCGCCTGGTATAAAGTACCAATCTACATGCTGGCC
CAGTACCTCGGGTCCTTCGTAGCGTCCTGCGTCGTGTACGTCGCTTACGTAGATCGCATC
TACGCTGTGGATCCCAAC---AAAACGCTCGCCACTGCTGGAATCTGGGCCACGTATCCT
GATAGCATCCTCACAATCGGAAACGCCTTTTTCGATCTGTTTATTGGTACGGCGCTGCTC
TTGCTCGGAGTGATGGCCGTCTCCGACTCGAGGAACATGGAAGTGCCGAAACACATTGTG
CCAATTTGCGTGGGAGGGATCATCGTGGCCATCGGCATGTGCTTCGGTCTCAACTGCGGC
TACGCTGTCAACCCGGCCAGGGACTTAGCACCCAGGTTCTTCACCTACATCGCTGGCTGG
GGCAGCCAGGTGTGGTCACCACTTAACGGCCACTATTGGTGGATACCGATCATCGCGCCT
CACCTCGGAGCTATCGTCGGAGTCTGGTCGTACCTGCTGCTGATCGAATTCCACTGGCCT
TCCACATCCGGAAAC
>GCIH01022531_Symphylella_sp_GlpA1
ATGGAGGACCTTGCATCAAAGCTGCGAATTAGAAATAGCCTAGTCCGAGAAGGATTAGCC

GAATTTTTGGGAACTACGCTGCTTTTGGCTTTCGGAGATGCGTGCGTGGCCCAGACGGTG
CTGACAAGGGGTGGTTTCGGCAGTTTCGTCACGATTCACCTGGGATGGGCGGCCGCCGTG
GCTATAGGCGTCTACGCTTGCGGAGGAGTCTCAGGTGGTCATCTGAATCCGGCAGTTACG
GTAGCGATGGCGACCATCGGTCGATTCCCGTGGGTTAAGGTTCCAGTCTACTTCCTCGCA
CAATATCTCGGAGCATTCGTCGGATCGTTCATCGTCTACATCACCTACATAGATTCCATA
CACTCGTTTCAAAACGGAAGCCTGACGACGGACACTGCAGGAATCTTCACCACTTTTCCA
AAGGCCGAAATAGGCATCGGAAACGCCTTCGCTGATTTGTTTGTTGGTACGGCGGTCCTG
CTTCTGTTCGTGATGGCCATTTCGGACGCTCGCAACATGGAGGTGCCCAAGTTTGCCGTT
CCCGCCCTGGTCGGTCTCGTCCTGATGGGAATCGGAATGAGTTTCGGAGCCAACTGCGGA
TACGCCGTCAACCCGGCCCGCGATTTCTCGCCGCGACTCTTCACCTTCATTTTCGGATGG
GGATCCAGCGTTTTCCAGCCACTCGATGGCCACTACTGGTGGATCCCCATCATTGCTCCG
CACTTGGGAGGCATTTTCGGCGTCTGGGCCTATCTCCTGCTGATCGAATTCCACTGGCCG
AGCACGCCCTCTCCG
>GERO01036086_Acopauropus_ornatus_GlpA2
TTCGACGACTTCGCGCGAAATCTGAGGATAAAAACGACGATAGTTCGGGAATTTTTGGCG
GAGCTTTTAGGAACGTTCATTTTGCTGGTTATTGGTGATGGGTGCGTGGCGCAATACAAA

--------------------GGCGGAGTCACCGGAGCGCATCTGAATCCGGCGGTGACG
CTGGCGATGGCCACGCTTGGCAAGCTGAAGTGGGTGAAGGTGCTGCCTTACATGATCGCC
CAATACATCGGCGCTTTCTTGGCTTCGATCGTCCTCTACTTCGTCTACAAAAACGCCCTC
GACGTGATGGACCCAGAC---AAGACCCTGGACTCGGCCGGCATCTGGGCCACGTATCCG
CGCAGCTACCTGTCCTCCGGCAATGCGTTCGCCGATCAGCTGTTCGGAACCGGCCTGCTC
CTTGTCGCCGTGATGGCCATCACCGACAATCGCAACATGGAGGTGCCGAAGGGGCTGGCG
GCGCCGCTAGTGGGCGTTACAATCGCCGCCATCGGCATGTCGTTCGGCGTCAACTGCGGC
TACGCCATCAACCCGGCCAGGGACCTCGCCCCGCGTCTCTTCACCTACATCGCCGGATGG
GGCAGCGACGTCTTCACGGCGTTCGACCAC---TTCTCCTGGATTCCGTTCGTGGCGCCG
CACATCGGGGCCGTGCTGGGCGCCTGGGTCTACCAGATGTTCGTCGGCTTTCACTGGCCG

>GERO01000350_Acopauropus_ornatus_Glpa1
TTCGAAGACTTCGCTCGACATATGAGGATAAAAACTTTGATCGTTCGAGAATTACTGGCG
GAATTATTAGGAACTTTCGTCCTAATCGCGATCGGTGACGGCAGCGTAGCTCAGTTCAAA
ATTCTCGCCAATGACGGCGGGAGCGTCTTATCCATATATTTGGGATGGGCGGCCGCGGTC
ACGCTGGCAGTGGCGGTAGCCGGCGGTGTATCAGGTGCGCATTTGAATCCTGCCGTCTCG
CTGGCGATGGCCACGCTGGGAAAGCTGAAGTGGTACAAGGTGTTACCGTACATGATCGCC
CAATACATCGGCGCCTTCCTCGCCTCCGTCGTCGTCTTCTTCGTCTACAAAAATGCACTA
GACAACTTGGATCCGGGA---CGGACACTGGACTCCGCGGGCATCTGGGCGACATATCCC
CGTGGCTACCTATCCACCGGAAACGCCTTTGCCGATCAGCTGTTTGGCACGGGGATGCTA
CTGCTGTGTGTGATGGCCATCGGGGACAACCGCAACATGGAGATTCCGAAGGGCATAGCC
CCGGCTATGGTAGGCGTGACGATCGCGGCCATCGGCATGTCTTTTGGCGCCAACTGCGGG
TACGCCATCAATCCCGCTAGAGATCTTGCTCCGCGATTCTTTACTTTTGTTGCCGGATGG
GGTTCGGACGTATTTACGGCTAACGACGGG---TTTTCTTGGATTCCGTTTGTAGCGCCA
CACATCGGGGCCGTCATAGGCGCGTGGGTGTACCAGATGTTCATTGGATTCCATTGGCCG
GTCGAAGGGGAGATG
>GCAH01036525_Clinopodes_flavidus_Glp1
ATGGAAGCAGCTTTAGGTCGACTTCGAGTCAGGATACCCATAGTCCGAGAGGTGTTTGCT
GAGTTTTTGGGAACGTTTGTTCTTCTATTTATTGGAAATGCATCAGTTGCCCAAGCCTTT
CTCAGTGACTTTAAGGATGGAACTATGCTGTCAATCAATTTTAGTTGGGGAATTGCTGTG
GCCTTTGGAGTGTTCGTCGCTGGAGGTGTATCAGGTGCCCATTTAAATCCAGCTGTTACA
GTAGCCCTTGCCACTTTGGGCAAATTTTCATGGTTGAAAGTGATACCGTACATTATTGCT
CAATACATAGGAGCATTTATTGGAGCTGCCTGTGCTTATTGGGTATATCTAGATGCAATT
ACTGTTGCTGGATATTCA------ACGCCCGGTACGGCTGGGATATTTGCAACCTATCCT
CAAAATTACCTTTCAGCTGGCAATGCCTTTGTTGATCAGATTATGGGAACTGCAATTCTC
ATGATGGGTATATTAGCTTTAATCGACCCGTACAACATGTCAGTACCGAAATACGCGATA
CCCTTGGGAGTTGGGTCTTTAGTTGGCGTTATTGGTATGGCATTAGGACACAACTGTGGT
TACGCAATCAATCCGGCAAGAGATTTATCACCGCGCTTGTTTACGCTGGCAGCTGGATGG
GGAACTATAACCTTCGAGTATCAAAATCAT---TACTGGTGGATTCCAATAGTAGGGCCT
CATCTCGGTGCCATCATGGGGGGTTGGCTTTACCAGTTGTTGGTTGGAGTTCATTGGCCA
CTTTTAGATGAAGAA
>GESB01026876_Henia_illyrica_Glp
ATGGATGGACTTTTGAAACGACTCCGTATCAGGATACCCATTGTACGCGAGGTGTTTGCT
GAATTTTTGGGGACGTTTGTACTTCTATTTTTTGGATGTGCATCAGTTGCCCAAGCTGTA
CTTAGTGATTTTACCTCTGGAACGATGCTTTCGATAAATATAAGCTGGGGTATAGCAGTC
GTGTTTGGAGTTTTGGTAGCTGGAGGAGTTTCAGGTGCCCATATAAATCCGGCAGTCACG
TTAGCGCTTGCCACTTTAGGCAAATTTTCTTGGTGGAAAATATTACCATACATGCTTGCT
CAATATATAGGTGGTTTCATGGGAGCTGCAGGATGTTATTGGGTATACATAGATGCCTTT
CATGCGAAAGGCTATAAG------ACACCCGACACAGCTGGAGTATTTGCGACTTATCCT
AGAGATTATCTCTCAGCTGGCAATGCTTTTGTTGATCAGATTATGGGTACAGGAATTCTT
ATGCTGGGCATATTAGCTTTGACTGATCCAAACAATATGGCAGTTCCAAAATACGCCATT

CCATTTGGTGTTGGATCTTTGGTGGCTGTTATTGGTATGTCACTTGGACATAATTGCGGC
TATGCAATCAATCCTGCAAGAGACTTGTCACCCCGATTATTTACACTCTGTGCTGGATGG
GGAACAGAAACCTTCGAGTATCAAGATTAC---TACTTTTGGATTCCTATAGTCGGACCT
CATCTTGGTGCCATCATGGGAGGTTGGTTATATCAATTGTTGGTCGGTCTTCATTGGCCA
GCTTTGGACGAAGAA
>AFFK01019422_Strigamia_maritima_Glp
ATGGATGCAGTGTTGAGTCGTCTTCGTGTGAGGATACCCATCGTCAGGGAAGTGTTTGCC
GAGTTTTTAGGAACGTTTGTACTTCTGTTTGTGGGCATTACATCAGTCGCTCAAGCAGTT
CTCAGCGATGGCAAATCTGGAACCATGTTGTCAATTAATCTCAGTTGGGGTATCGCAGTG
GCCATGGGAATTTTCATCGCTGGAGGTGTTTCAGGTGCCCATTTAAATCCAGCTGTTACA
GTCGCCTTAGCCACTTTGGGCAAATTTTCGTGGGTAAAGGTATTACCATACATCATTGCT
CAATACATAGGAGCATTTATAGGAGCCGCGTGTGCCTATTGGGTTTACCTCGACGCAATC
GGTAAAATGGGCATGATG------GTCCCCGAAACGGCTGGAATCTTTGCAACCTTTCCA
AAAGAATACCTCTCAAATGGCAACGCTTTTGTTGATCAGGTAATGGGAACTGGAATTCTA
ATGATGGGTATTTTGGCCATTGTCGACCCATACAACATGTCTGTGCCCAAATACGCCATA
CCTTTAGGAGTTGGTTCGTTGGTTGGAGTCATTGGAATGGCGTTGGGACACAACTGTGGC
TACGCAATCAATCCCGCGAGGGATTTATCACCGAGACTTTTCACTTTGGCAGCTGGATGG
GGGAAAGAGACATTTGAG---CAGAATCAT---TATTGGTGGATCCCAATACTTGGACCT
CATCTCGGTGCCATCATGGGCGGATGGCTGTATCAATTGTTGGTTGGTGTTCATTGGCCG
CTTTTGGATGAAGGT
>GESK01021956_Strigamia_acuminata_Glp
ATGGAAGCAGTTTTAAGTCGTCTCCGTGTGAGGATACCCATCGTCAGGGAGGTGTTTGCC
GAGTTTTTAGGAACGTTTGTCCTAATCTTTGTTGGCATATGCTCGGTCGCCCAACTCGTA
CTCAGTGAAAGTCAATCAGGAACATTCTTGTCAGTCAACTTAGGCTGGGGTATTGCGGTG
GCCATGGGCGTTTTCATCGCGGGAGGTGTTTCAGGCGCACACTTAAACCCAGCGGTTACG
GTGGCCTTAGCCACATTGGGCAAATGTTCATGGAAAAAGGTAGTACCATACCTCATTGCA
CAGTACATAGCAGGATTCATAGCAGCCGCATGCGCCTATTGGGTCTACCTAGATGCTTTT
GGTAAACAGGGATTCAGC------ATCCCCACTACGGCTGGAGTGTTTGCAACCTATCCA
AAAGATTACCTTTCAAATGGTAACGCTTTTGTTGATCAGGTTATGGGAACGGGGATTCTA
ATGATGGGCATTTTGGCCATTACCGACCCGTACAACATGGCTGTGCCAAAATATGCGATA
CCTTTAGGAGTTGGTTCGTTGGTCGGAGTTATCGGTATGACGTTGGGAGAGAACTGTGGT
TACGCCATCAATCCTGCGAGAGATTTATCTCCACGACTTTTCACGTTAGCGGCTGGATTT
GGAAAAGAAACATTTGAATACAGCAATCAT---TTTTGGTGGATCCCAATAGTTGGTCCT
CATCTCGGTGCCATCATGGGTGGGTGGATTTATCAACTGTTGATTGGTTTCCATTGGCCA
ATGCTGGATGAGGAA
>GCIL01014214_Himantarium_gabrielis_Glp
ATGGAGACTGTGTTAAAACGTTTACGTGTGAGGATACCAATCGTTCGTGAAGTGTTTGCG
GAGTTTTTGGGCACGTTTGTGTTAGTGATGGTTGGAACTGCGTCGGTTGCACAAAGTGTA
TTGAGTGAAGGCAAATTCGGCGAGTTTGTTTCCATTAATCTAGCTTGGGGTATAGCAGTA
GCTCTAGGTGTTTTTATAGCAGGCGGTGTTACAGGTGCTCATCTCAACCCAGCAGTGACT
CTTGCGTTGGCTACGTTAGGAAAATTTTCATGGAGAAAGGTTGTTCCGTATTTTGTTGCC
CAATACGTCGGTGCGTTTTTAGGAGCTGCTTGCACTTATTGGGTTTATCTAGATGGTTTG
ACATCGCAACCACTGACC------GTTCCTGGCACTGCAGGAATATTTGCCACTTACCCT
AGAGATTATTTATCAGCAGGCAACGCATTCGCTGACCAAATTGTGGGCACTGCAATTCTC
ATGATGAGCATCTTGGCAATAACCGACCCGTACAACATGGCCGTTCCTAAATATGCAATC
CCGTTGAGTGTTGGTTCAGTCGTCGCTGCTATTGGTTTAAGCTTGGGATACAATTGTGGC
TACGCTATCAACCCTGCGAGAGATCTTTCTCCTCGACTTTTCACATTAGCGGCTGGATGG
GGAGAAGAAACTTTCACTTACGAAAATCAT---TTTTGGTGGATTCCAATCATTGGTCCT
CACCTCGGCGCCATTATGGGTGGATGGTTGTACGTTTTACTCGTTGGTGTACACTGGCCA
ATTTTGGATGAATTA
>GESL01011533_Schendyla_carniolensis_Glp1
ATGGACGATCTGCTTGGACGTCTCCGGGTTCGCATCCCGATAGTCAGAGAAGTTTTTGCC
GAATTTCTAGGCACTTTTGTGCTTTTGTTCGTAGGCACTTCTTCTGTGGCGCAGAAAGTC
CTCGGTGGTGAAGAATACGGAACATTCCTCTCTATCAACTTTGGATGGGGTATAGCAGTA
GCACTAGGAATATTTGTATGCGGAGGAGTAACAGGAGGACACTTGAATCCAGCAGTGACA
GTTGCATTGGCAACTCTTGGTAAATTCTCATGGAAAAAAGTTATTCCTTACATATTGGCC
CAGTATTTCGGAGCGTTTATTGGAGCAGCTTGCACTTATTGGATTTACTTAGATGCTCTT
GATCAAAAAACAATGTCA------GTTCCTGGAACTGCAGGAATATTTGCTACGTATCCT
CAGGAGTTTCTCTCTTTGGGCAATGCTTTTGCTGACCAGATTATGGGAACTGCCCTGCTG
ATGATGTGTGTGCTGGCCATCACCGATCCTTACAACATGGCCGTTCCGAAATATGCCATT
CCCATAGCCGTTGGTGGCGTTGTAGGTGTCATTGGAATGACACTCGGCTTTAATTGTGGT
TATGCCATCAACCCTGCAAGAGATCTTGCGCCTAGATTATTTACCTTAGCAGCAGGATGG
GGAACTGAGGTGTTCAGTTACAAAAACTAT---TATTGGTGGATCCCAGTTGTAGGGCCT
CACCTTGGAGCCATTATGGGGGCTTGGTTCTACCAGCTGTTGATTGGTGTCCATTGGCCA
ATGTTGGACGAAGAC
>GCAP01019080_Scolopendra_cingulata_Glp
ATGGAGAAGATTTTTAGCCGCCTGAGGATCAAAAACCTGCTTGTACGGGAGTTGCTGGCC
GAATTCATTGGAACATTTATTCTGATACTATTCGGGACAGCTTCAGTGGCTCAGGCAGTT

```
CTAAGTGGTGGACTCAGTGGAACCTTTTTATCCATCAACTTGTCATGGGGCATTGCTGTG
GCTTTGGGTGTTTATGCCAGCGGTGGAGTTTCAGGCGGTCATTTAAACCCGGCCGTGACA
TTGGCCTTGGCGACAGTCAGGAAATTCCAGTGGAAAAAGTTGTTCCTTTTATGATGGTT
CAGTATCTGGGGGCCTTCGTCGCTTCTGCCTGCACTTTTTGGGTCTATTATGACGGTATA
ATTTCTGAAGGGAAATCG------GTCCCCACCACCGCCGGCATCTTCGCCACTTACCCC
AAAGAATACCTTTCCCTGGGCAACGCTTTAGTCGATCAGATCGTAGGCACGGCTATGTTA
CTCATAGGCATTTTAGCCATCTCAGATCCAAAGAACATGGAAGTGAGCAAGGGCTCGGTC
CCCATGTGCGCTGGCCTCGTCGTCGCCGTCATCGGCATGAGTCTGGGCCACAACTGCGGC
TACGCGATCAACCCTGCTCGGGATTTGAGCCCGAGGTTGTTCACCTTGGCTGCCGGGTGG
GGAGTTGAAGTTTTAACATATGGAAACTAC---TATTGGTGGATTCCCGTTGTAGGACCT
CATCTTGGAGCCATAATTGGCACGTGGACATATCAACTTTTTATTGGTTTCCATTGGGAA
CCAGAAGAAGAGGAG
>GERT01002318_Cryptops_anomalans_Glp
ATGGAACGACTGTTGAGACGGTTACGGATCAAAAACAAGTTTGTCCGAGAAATTATTGCT
GAATTTCTAGGGACATTTGTGCTCATGTTATTTGGAACAGCCTCGGTAGCTCAAGCTGTG
CTTAGCGGTGAAGCGAAAGGTACAATGCTATCCATCAATTTGTCATGGGGCATTGCAGTT
ACCATGGGTATTTATGTTGCTGGTGGTGTCTCAGGTGCACATCTAAACCCTGCAGTGACT
TTGGCACTAGCTACCGTTGGAAAATGCGATTGGATAAAAGTGATTCCATACATGCTAGCG
CAGTATATCGCCGCATTCGTTGCATCTGCAATAACTTATTTTGTTTATTATGATGCTTTC
CAA---TATACGTATGTC------GTACCTGACACTGCCGGTATCTTTGCAACCTATCCA
GGAAAATGGTTAACGCCGGGCATTGCTTTTCTCGACCAGGTGGTTGGTACAGGAATGCTG
ATGCTGTTAGTCTTGGCCATTACGGATGATCGCAACATGGAATGTGCCAAAGGTTACATT
CCCGTTGCTATAGGTCTTGTGGTAGCTGTCATTGGAATGTCCCTTGGTGTCAATTGCGGT
TATGCCATTAATCCTGCTCGTGATTTCAGCCCACGTTTATTCACTTTTTGTGCTGGATGG
GGAATTGAAGTTTTCACATATGGTGACTGGCCATATTGGTGGGTACCAATAGTCGGACCT
CATGTTGGAGCCATCGTTGGTGGATGGACTTATCTGTTATTAATTAGTCTTCACTGGGAG
CCAGAAGAAGAGAAA
>GESL01037829_Schendyla_carniolensis_Glp2
CAAGAGATTCTTCTTACACATCCAAGA---TCTGCTGAGTTATGGAGAAAGATACTTGCA
GAACTCATAGGCACTTTTGTGTTAATGCTTTTTGGAACAGCCAGTGTAGCACAAGCAAAA
CTAAGTAATGGAGAAACTGGAAATATAATCACTATTAATGTAGCATGGGGCCTTGCTGTG
GTCATGGGAGTTTATATAAGTGGAGGAGTGTCAGGTGGTCACTTAAATCCTGCGGTTACT
GTAGCATTAGCATCTGCCAGATTGTTTCCCTTTAGAGATGTTATCCCTTACATTCTTGCA
CAGTATATTGGTTCCTTTTTGGGTGCCTTGACAACATGGTCTCTTTATTATGAGGCAATT
AAATACGTGCATACTGAAGAAATAGTACCTACCACTGCAGGTATATTTGCCACTTATCCT
GGTCCACATCTAGGAATTGCTACTTTATTTTGGGATCAGGTCGTTGGAACTGCATTACTA
CTTTTATGCGTGATGGCCATAACGGATCCCAACAACATGGATTGTCCTAAAGGAGTATTA
CCCATTGCAATAGGGTTGATTGTAATAGTAATAGGAATGACCCTGTGCCACAACTGTGGC
TATGCCATTAATCCAGCGAGAGATTTGAGTCCAAGAATACTAACTGCAATTTGCGGATGG
GGTGCTGAAGTTTTTTCATATCAGAATTAC---TATTTCTGGATACCAGTTATTGGTCCC
CATCTTGGTGGCATAATTGGGATTTGGCTGTATATAGTTACTATTAGATTGTTCTGGCCT
CGAGAACAGCCGCCA
>GCAY01018378_Lithobius_forficatus_Glp
ATGGACGCTGCTCTCAGCCGCCTGCGGATCCGGTCAGAGGTCGTCCGGGCCATGTTGGCC
GAGTTCTTAGGAACCTTTGTCCTTATGGTTTTCGGGACAGCCTCGGTGGCTCAATTCATG
TTGGATCCTTCCCATAATGCCTCGCTCTTGGCATTAAATCTATCATGGGGCTTTGCTGTC
ATGCTCGGAATTTTCGTTAGCGGTGGAATATCAGGTGGCCATTTGAATCCCGCTGTTTCC
GTTGCCTTAGCAACGACTGGAAGTTTTCCTTGGATCCGCGTTGCCCCTTACTTGATTGCT
CAGTATTTGGGTTCTTTCGTTGGCAGCGCCACCACCTACTGGGTCTATTTA---------
------------------------------------------------------------
---------------------------------GATGCTCTCTACCAGACCGCCTTGCTA
CTTCTGGGTGTAATGGCCATCACTGATCCTCGTAACATGGAGACTCCGAAGCATTTTGTT
GCATTTGGTGTTGGTACCCTCGTCATGGTGATTGGTATGACCTTTGGCCTTAACTGTGGC
TATGCTATCAATCCAGCGAGGGATCTGAGTCCCCGTATTATGACGTACCTGGCTGGTTGG
GGACCGGAAGTATTCACGTACGGTGAAAAC---TATTGGTGGATCCCCATTGTAGGCCCC
CATCTTGGAGCAATCGTGGGCGTGTGGATCTACAAACTGCTGGTGTCTGTTCATTGGCCG
CCTCCGCCAGAAGAC
>GERX01006732_Eupolybothrus_tridentinus_Glp
ATGGACGCAGCGCTCAGTCGCCTGAGGATCCGGTCGGAGGTGGTGCGGGCCATGCTGGCC
GAATTTCTGGGTACTTTTGTTCTTATG---------------------------------
------------------------------------------------------------
------------------------------------------------------------
------------------------------------------------------------
------------------------------------------------------------
------------------------------------------------------------
------------ACGTTGCAGGGTGGCTTTGCTGATCAGGTGGTTGGCACAGCCCTATTG
CTGTTAGGAGTGATGGCCATTACTGACCCACGCAATATGGAGACGCCAAAGCACTTTGTG
GCTTTTGGCGTAGGCTGTGTGGTGATGGTGATTGGCATGACGTTTGGACTAAACTGCGGC
```

TACGCCATCAACCCTGCCCGAGATCTCAGCCCCCGAATCTTAACGTACATCGCTGGTTGG
GGCAGTCAAGTATTCACGTACGGTGATAAC---TACTGGTGGATTCCCATTGTGGGCCCT
CATTTAGGAGCCATTGTGGGCGTATGGATCTACAAACTGTTGGTGTCGGTTCACTGGCCG
CCCCCACCCGAAGAT
>GCIY01020397_Scolopocryptops_rubiginosus_Glp
ATGGAAAACGCTTTTAAGCATCTGCGGATCAAGAATACGTTTGTAAGGGAACTGCTGGCG
GAATTCCTCGGGACCTTCGTGCTCGTAATGTTCGGAACGGGCTCCATTGCTCAATTTATG
CTCAGTGAAGGGAGTCACGGCAGCCTTTTGACCGTCGCCTTATCATGGGGTTTCGGAGTA
ACTATGGGTCTTTTTGCGGCTGGAGGAGTGTCA---GGTCATCTTAATCCTGCTGTCACC
TTGGCCATGTTCTCGTGCGGTAAATTAATCTGGTGGAAAGTCGTTCCATATATGTTAGTT
CAATATTTCGGAGCTTTCGTTGCCGCAGCTTGTACGTGCGCGGTATACATTGGTGCTATT
CAGAAGAGAGGATTTGGT------GTTCCCGGTACGGCTGCTATCTTTGTAACATTTCCA
GAAGAGTTCGTTACACATGCAAATGCTTTCTTAGATCATGTTGCAGGGACGGCGTTACTG
TTACTTCTGGTCATGGCTATCACTGATCCCCGGAATATGGAAGTTCCCAAAGGGCTTATT
CCTCTGCCCGTGGGATTTATCGTCACTGTGATCGTGATGGCATTTGGTTACAACTGCGGT
TGCCCCCTCAATCCCGCCCGAGATCTAGGCCCCCGGATATTCACTTCAATTGCGGGATGG
GGCACCGATGTGTTCACCTATCGTTCTAAC---TATTGGTGGATTCCTGTAGTGGGACCT
CACGTTGGAGCTATTGTTGGGGTTTGGACGTACCTATTTTTTGTTGGATTTCACTGGCCA
CCTCCTCCAGAAGTC
>GEAZ01012763_Craterostigmus_tasmanianus_Glp
ATGGCTCTCATGCTTCAAAGACTTCGGATAAAAGTAGACTGGGTTCGAGAATGCATGGCC
GAATTTTTAGGAACTTTCGTGCTTGTAGTAATTGGTGATGCATCTGTGGCTCAATCAATG
CTCAGTAGCGGCACCTCTGGAAATATGCTATCCATTAACCTAAGCTGGGGACTGGCGGTT
ATGCTTGGCGTTTTCGTTAGCGGTGGTGTATCAGGAGGTCATTTAAACCCAGCAGTAACG
CTTGCTATGGCTATCTGTGGGAAGTTACCCTGGAAGAAGGTTCCCCATTATATGGGCGCT
CAGTTTCTTGGTGGATTTGTCGGTGCTGCTTGTGTCTACTGGACGTATTATGATGCAATA
CAGGTACAGGGTTTCCAA---TACACTGTTACGACGGCCGGAATTTTTTCCACCTATCCC
CAGCCGTATCTTAGCCACTTAAACGCAGTGGTTGACCAGATCGTGGGCACAGGCATTCTC
TTATTAGGAATAATGGCTATCACGGACGGTAGAAATATGAATGTGGAGCAGGGAATGGTG
CCTCTACTTGTTGGTCTCTTGGTGGCGGCCATTGGAATGACGTTGGGCCACAACTGCGGT
TATGCTATTAATCCAGCTAGAGATTTGTCGCCCAGATTTTTCTGCTTCATTGCTGGATGG
GGTTCCGAAGTCTTTACATTTGCCAATCAT---TATTGGTGGATCCCAACCTTCGTGCCG
TTCATTGGAGCCATCCTGGGTGCCCTTACATACATATTGTTTATCGGCATTCACATTCCA
GAGTCTGAAGAAGCA
>GCAQ01011625_Scutigera_coleoptrata_Glp
GTTAGCGAGATGCTCAACCGGCTGAAAATTCAGAGTGAAATAATACGGGAAATGTTGGCG
GAATTTGTTGGAATATTTATTCTTATGACTTTCGGAATGGCCTCAGTCGCTCAAGCTATA
TTTTTTCGAGGCGACGATGGTAGCTTCATAGGACTGGATCTCGCATGGGGTTTAGCGGTT
GCGTTTGGAGCTTACGCCAGCGGAAAAGTGTCGGGTGGCCATTTGAATCCAGCAGTTTCT
CTAGCGTTGGCTGTTGTTGGCAAATTCCAATGGAGAAAAGTAATGCCTTATATCGTCGCT
CAGTTCGTTGGTTCCTTTTTAGCTTCTGCCATTATTCACGGTGTCTATTATGATATGATT
CAAGAA------------GCTCCTCTAGAGAATACGATGGGTATCTTTGCTACCTACAAA
CACGCCGGAGTAACAAATCGACAATGCTTTATTGATCAGATCGTGGCAACGGGAATGTTA
TTACTCTGTATACTTTTACTAACTGAT---GAACACACGGGTCTTCCTGGACATATAGTG
CCTTTACTCATTGGACTGAGTGTAACCACCATAGGCTTAACATTTGGATTCAATTGCGGC
TTTCCTATAAATCCTGCAAGAGACTTGGCTCCTCGTTTCTACACGTTCATTGCGGGCTGG
GGATCTTATGTCTTTAGCCACGGAGATTAT---TACTTCTGGATACCTATCGTTGGACCA
GTTTTAGGAGGAATAATTGGCTCATGGTTTTACATCTGTCTTATTAGTTTCCACATCCCT
TCTGCCACAGATACT
>WP_032330355_Escherichia_coli_GlpF
---------------------------------TCAACCTTGAAAGGCCAGTGCATTGCT
GAATTCCTCGGTACCGGGTTGTTGATTTTCTTCGGTGTGGGTTGCGTTGCAGCACTAAAA
GTCGCTGGTGCGTCTTTTGGTCAGTGG---GAAATCAGTGTCATTTGGGGACTGGGGGTG
GCAATGGCCATCTACCTGACCGCAGGGATTTCCGGCGCGCATCTTAATCCCGCTGTTACC
ATTGCATTGTGGCTGTTTGCCTGTTTCGACAAGCGCAAAGTTATTCCTTTTATCGTTTCA
CAAGTTGCCGGCGCTTTCTGTGCTGCGGCTTTAGTTTACGGGCTTTACTACAATTTATTT
TTCGACTTCGAGCAGACTGTTGAAAGTGTTGATCTGGCTGGCACTTTCTCTACTTACCCT
AATCCTCATATCAATTTTGTGCAGGCTTTCGCAGTTGAGATGGTGATTACCGCTATTCTG
ATGGGGCTGATCCTGGCGTTAACGGACGATGGCAACGGTGTACCACGCGGCCCTTTGGCT
CCCTTGCTGATTGGTCTACTGATTGCGGTCATTGGCGCATCTATGGGCCCATTGACAGGT
TTTGCCATGAACCCAGCGCGTGACTTCGGTCCGAAAGTCTTTGCCTGGCTGGCGGGCTGG
GGCAATGTCGCCTTTACCGGCAGAGACATTCCTTACTTCCTGGTGCCGCTTTTCGGCCCT
ATCGTTGGCGCGATTGTAGGTGCATTTGCCTACCGCAAACTGATTGGTCGCCATTTGCCT
TGCGATATCTGTGTT
