# Supplementary material for: Lineage-level divergence of copepod glycerol transporters and the emergence of isoform-specific trafficking regulation
Source: Commun Biol. 2021 May 31;4:643. doi: 10.1038/s42003-021-01921-9 (PMC8167128; doi:10.1038/s42003-021-01921-9)
Supplement: Supplementary file 6 — Reporting Summary [file 42003_2021_1921_MOESM6_ESM.pdf]

nature research

Corresponding author(s): Roderick Nigel Finn & Joan Cerdà

Last updated by author(s): Feb 10, 2021

Reporting Summary

Nature Research wishes to improve the reproducibility of the work that we publish. This form provides structure for consistency and transparency in reporting. For further information on Nature Research policies, see our Editorial Policies and the Editorial Policy Checklist.

Statistics

For all statistical analyses, confirm that the following items are present in the figure legend, table legend, main text, or Methods section.

n/a	Confirmed	
☐	☒	The exact sample size (*n*) for each experimental group/condition, given as a discrete number and unit of measurement
☐	☒	A statement on whether measurements were taken from distinct samples or whether the same sample was measured repeatedly
☐	☒	The statistical test(s) used AND whether they are one- or two-sided<br>*Only common tests should be described solely by name; describe more complex techniques in the Methods section.*
☒	☐	A description of all covariates tested
☒	☐	A description of any assumptions or corrections, such as tests of normality and adjustment for multiple comparisons
☒	☐	A full description of the statistical parameters including central tendency (e.g. means) or other basic estimates (e.g. regression coefficient) AND variation (e.g. standard deviation) or associated estimates of uncertainty (e.g. confidence intervals)
☐	☒	For null hypothesis testing, the test statistic (e.g. *F*, *t*, *r*) with confidence intervals, effect sizes, degrees of freedom and *P* value noted<br>*Give P values as exact values whenever suitable.*
☐	☒	For Bayesian analysis, information on the choice of priors and Markov chain Monte Carlo settings
☒	☐	For hierarchical and complex designs, identification of the appropriate level for tests and full reporting of outcomes
☒	☐	Estimates of effect sizes (e.g. Cohen's *d*, Pearson's *r*), indicating how they were calculated

Our web collection on statistics for biologists contains articles on many of the points above.

Software and code

Policy information about availability of computer code

Data collection	Aquaglyceroporin sequences were retrieved using the full-length deduced proteins of the clones or exon-deduced peptides as tblastn queries on open-source whole genome shotgun (WGS), transcriptome shotgun (TSA) and nucleotide databases (NCBI [blast.ncbi.nlm.nih.gov]).
Data analysis	For the phylogenetic, syntenic and sequence analyses, data sets of the deduced amino acids were aligned using the L-INS-I or G-INS-I algorithms of MAFFT v7.453, and subsequently converted to codon alignments using Pal2Nal prior to Bayesian (Mr Bayes v3.2.2) inference. To detect errors generated by the automated algorithms, alignments were lineage sorted according to the resulting trees and inconsistencies corrected manually using MacVector (MacVector Inc, Cambridge, UK). All trees generated were processed with Archaeopteryx and rendered with Geneious (Biomatters Ltd, New Zealand). Synteny analyses were conducted via tblastn searches of WGS databases. Data on oocyte permeasbility were analyzed using the STATGRAPHICS PLUS 4.1 software (Statistical Graphics, USA).

For manuscripts utilizing custom algorithms or software that are central to the research but not yet described in published literature, software must be made available to editors and reviewers. We strongly encourage code deposition in a community repository (e.g. GitHub). See the Nature Research guidelines for submitting code & software for further information.

Data

Policy information about availability of data

All manuscripts must include a data availability statement. This statement should provide the following information, where applicable:

- Accession codes, unique identifiers, or web links for publicly available datasets
- A list of figures that have associated raw data
- A description of any restrictions on data availability

All data and accession codes are available in the main text and the additional files submitted with the manuscript

Field-specific reporting

Please select the one below that is the best fit for your research. If you are not sure, read the appropriate sections before making your selection.

☒ Life sciences ☐ Behavioural & social sciences ☐ Ecological, evolutionary & environmental sciences

For a reference copy of the document with all sections, see nature.com/documents/nr-reporting-summary-flat.pdf

Life sciences study design

All studies must disclose on these points even when the disclosure is negative.

Sample size	The sample sizes were between 8 to 37 oocytes per treatment.
Data exclusions	No data were excluded
Replication	Replicates were conducted on biologically independent oocytes.
Randomization	Oocytes were selected randomly
Blinding	Blinding was not relevant in this study because the researcher applied the treatments before measuring the effect.

Reporting for specific materials, systems and methods

We require information from authors about some types of materials, experimental systems and methods used in many studies. Here, indicate whether each material, system or method listed is relevant to your study. If you are not sure if a list item applies to your research, read the appropriate section before selecting a response.

Materials & experimental systems

n/a	Involved in the study	
☐	☒	Antibodies
☒	☐	Eukaryotic cell lines
☒	☐	Palaeontology and archaeology
☐	☒	Animals and other organisms
☒	☐	Human research participants
☒	☐	Clinical data
☒	☐	Dual use research of concern

Methods

n/a	Involved in the study	
☒	☐	ChIP-seq
☒	☐	Flow cytometry
☒	☐	MRI-based neuroimaging

Antibodies

Antibodies used	Custom antibodies against synthetic peptides corresponding to intracellular regions of Lepeophtheirus salmonis aquaglyceroporins were generated.
Validation	The specificity of the antisera were confirmed by ELISA, immunoblotting and immunofluorescence microscopy of oocytes expressing the Lepeophtheirus salmonis aquaglyceroporins.

Animals and other organisms

Policy information about studies involving animals; ARRIVE guidelines recommended for reporting animal research

Laboratory animals	A laboratory strain of Lepeophtheirus salmonis was used. Xenopus laevis were purchased from the Centre de Ressources Biologiques Xénopes, Université de Rennes 1 (France)
Wild animals	n/a
Field-collected samples	n/a
Ethics oversight	Procedures relating to the care and use of animals and sample collection were carried out in accordance with the regulations approved by the governmental Norwegian Animal Research Authority (http://www.fdu.no/fdu/), or in accordance with the protocols approved by the Ethics Committee (EC) of the Institut de Recerca i Tecnologia Agroalimentàries (IRTA, Spain) following the European Union Council Guidelines (86/609/EU).

Note that full information on the approval of the study protocol must also be provided in the manuscript.
